# Global and regional seroprevalence, incidence, mortality of, and risk factors for scrub typhus: A systematic review and meta-analysis

**DOI:** 10.1016/j.ijid.2024.107151

**Published:** 2024-09

**Authors:** Qian Wang, Tian Ma, Fangyu Ding, Ahyoung Lim, Saho Takaya, Kartika Saraswati, Benn Sartorius, Nicholas P.J. Day, Richard J. Maude

**Affiliations:** 1 Mahidol Oxford Tropical Medicine Research Unit, Faculty of Tropical Medicine, Mahidol University, Bangkok, Thailand; 2 Centre for Tropical Medicine and Global Health, Nuffield Department of Medicine, University of Oxford, Oxford, UK; 3 Institute of Geographic Sciences and Natural Resources Research, Chinese Academy of Sciences, Beijing, China; 4 College of Resources and Environment, University of Chinese Academy of Sciences, Beijing, China; 5 London School of Hygiene & Tropical Medicine, London, UK; 6 Saw Swee Hock School of Public Health, National University of Singapore, Singapore, Singapore; 7 Oxford University Clinical Research Unit Indonesia, Faculty of Medicine, Universitas Indonesia, Jakarta, Indonesia; 8 Centre for Clinical Research (UQCCR), Faculty of Medicine, University of Queensland, Brisbane, Australia; 9 Department of Health Metric Sciences, School of Medicine, University of Washington, Seattle, USA; 10 The Open University, Milton Keynes, UK

**Keywords:** Scrub typhus, Seroprevalence, Incidence, Mortality, Risk factor, Systematic review

## Abstract

**Objectives:**

Scrub typhus is underdiagnosed and underreported but emerging as a global public health problem. We aimed to provide the first comprehensive review on the seroprevalence, incidence, mortality of and risk factors for scrub typhus.

**Methods:**

We searched PubMed, Scopus, Web of Science, China National Knowledge Infrastructure and other databases. Trended incidence and median mortality were calculated and pooled seroprevalence and risk factors for scrub typhus were evaluated using the random-effects meta-analysis.

**Results:**

We included 663 articles from 29 countries/regions. The pooled seroprevalence was 10.73% (95%CI 9.47-12.13%) among healthy individuals and 22.58% (95%CI: 20.55%-24.76%) among febrile patients. Mainland China reported the highest number of cases and South Korea and Thailand had the highest incidence rates. Median mortalities were 5.00% (range: 0.00-56.00%) among hospital inpatients, 6.70% (range: 0.00-33.33%) among patients without specified admission status and 2.17% (range: 0.00-22.22%) among outpatients. The significant risk factors included agricultural work, specific vegetation exposure, other outdoor activities, risky personal health habits, and proximity to rodents, livestock, or poultry.

**Conclusions:**

Our comprehensive review elucidates the significant yet variable burden of scrub typhus across different regions, underscoring its emergence as a critical public health concern globally.

## Highlights

• The overall pooled seroprevalence of scrub typhus was 10.73% in healthy people.
• The overall pooled seroprevalence of scrub typhus was 22.58% in febrile patients.
• Mainland China reports the most cases; South Korea reports the highest incidence.
• Hospitalized patients showed median fatality rates of 6.70%.
• Agricultural work, vegetation, and outdoor activities raise scrub typhus risk.

## Introduction

Scrub typhus, caused by the bacterium *Orientia tsutsugamushi* along with the closely related and recently described *Candidatus* Orientia chuto [1] and *Candidatus* Orientia chiloensis [2], is a significant but often neglected health concern across various regions of the world. While it predominantly affects parts of South and Southeast Asia, northern Australia, the islands of the western Pacific, and Indian Ocean [3],- an area referred to as the “tsutsugamushi triangle”- there is mounting evidence to suggest that scrub typhus exists and is expanding beyond these traditional boundaries [4]. Climate and environmental changes could be potential catalysts for this expanding geographic distribution, as alterations in temperature and habitat can facilitate the migration and expansion of vectors and hosts [4,5], thereby amplifying its threat globally [6,7].

Estimates from a comment published in 1997 suggested over a billion individuals at risk worldwide and another study published in 2003 suggested approximately one million clinical cases occur annually [8,9]. However, these figures lacks supportive evidence, as is the current state of the disease's seroprevalence across affected geographies. The true extent and burden of scrub typhus has been underreported due to limited awareness, scarce research, and non-distinctive clinical manifestations. The mortality rate has been estimated at approximately 6% for untreated cases with a vast reported range from 0 to 70% [10]. Our understanding of scrub typhus is further obscured by the complexities associated with its primary vectors, the larval trombiculid mites. The geographical distributions, risk factors influencing their behavior, and the dynamics of disease transmission through these mites remain inadequately explored.

While the epidemiology of scrub typhus has been the subject of several systematic reviews, there remains pronounced gaps in our understanding of the global epidemiology. Two seminal reviews, both published in 2017, have provided insights into the disease epidemiology and mortality across various nations [11,12]. Regional systematic reviews, such as one on India, contribute valuable insights on a country level [13]. Untreated scrub typhus mortality were also examined using the data primarily collected in the World War II era [10]. Scientific advances and enriched data provide the opportunity to enhance these analyses and update the knowledge.

Given that robust data and a comprehensive understanding of the epidemiology of this disease are indispensable for targeted detection and informed preventive strategies, further investigation is urgently warranted. In this study, we have two primary objectives. First, we seek to update the current knowledge on the global epidemiology of scrub typhus, including its geographical distribution, seroprevalence including age-specific and sex-specific seroprevalence, incidence, and mortality. Second, we aim to provide further insights into significant risk factors for scrub typhus and offer a systematic examination of the diverse risk factors and how these vary across geography. We hope by providing an up-to-date summary of the evidence, this review will inform the global research agenda and bridge gaps in understanding the burden of scrub typhus.

## Method

This systematic review adhered to PRISMA (Preferred Reporting Items for Systematic Reviews and Meta-Analyses) guidelines [14] (Appendix Table S3), the protocol had been registered with the International Prospective Register of Systematic Reviews (PROSPERO) with registration number CRD42022315209.

### Search strategy and selection criteria

To identify relevant publications, we undertook a systematic literature search without any language, publication date or geographical restrictions, to identify manuscripts that detailed occurrences of scrub typhus and/or reported fatalities or attributable mortality rates, as well as associated risk factors. We searched six databases: PubMed, Scopus, Ovid Medline, Ovid Embase, Web of Science, and China National Knowledge Infrastructure. Additional search to identify gray literature were conducted on NY Academy of Medicine Grey Literature Report, Mednar, WHO Global Index Medicus, ProQuest Dissertations, Theses Global database, Preprints in Europe PMC, WHO International Clinical Registry Platform, ClinicalTrials.gov, and the ProMED website. The first 200 results from Google scholar were used as an addition [15].

We developed and refined an exhaustive search string assisted by an experienced university librarian, incorporating Medical Subject Headings (MeSH) terms and customized for compatibility with each database. The search terms were the combination of scrub typhus synonyms (“*Orientia tsutsugamushi,*” “bush typhus,” “mite typhus,” etc.) and research outcomes of interest (“epidemiology”, “burden”, “prevalence”, etc.). The full search syntax used for each individual database can be found in the Appendix (Table S1). The initial search was done on 17th May 2022. The search results were pooled, and duplicates were removed using EndNote 20, with the source, search strategies, date of search and number of received records tracked and recorded.

All studies containing confirmed human scrub typhus cases were included. The inclusion criteria varied based on the type of study. For seroprevalence studies, the requirement was to report the seroprevalence of test positivity utilizing IgG and/or IgM assays for *Orientia* spp. Incidence studies were included if they reported incidence rates. When considering disease burden, studies that reported fatalities, attributable mortality rates, disability-adjusted life years (DALYs), or the years lived with a disability (YLDs) were included. For risk factors, observational and experimental studies that investigated exposures or protection measures were included. Only case reports and case series published from the year 2000 onwards were included to ensure the relevance and quality of the included data, leveraging the improvements in electronic database indexing that started around this time. We excluded studies with unavailable full text, the reports with the smaller sample sizes in cases of duplicate publication, studies without a clear case definition, studies without information on where participants were infected, and review studies without primary data.

The titles and abstracts of identified studies were independently screened by two reviewers (QW and TM), records in Korean and Japanese were reviewed by native speakers (AL and ST), and studies in other languages were translated and checked using Google Translate. For any potential eligible articles, the full text was screened to make the final decision about inclusion. To ensure that no relevant studies were excluded erroneously, a third reviewer (KS) randomly selected and verified the screening results. Any disagreements were resolved by consensus or following discussions with RJM. The Rayyan web-based platform was used to streamline this process [16].

### Data extraction and data analysis

Three standardized forms were developed using Microsoft Excel (version 2310) based on the review foci: seroprevalence/incidence/burden; case reports and case series; and risk factors. Relevant variables were extracted by two investigators independently (QW and TM), and for articles in Korean and Japanese, QW extracted information using Google Translate, and then AL and ST (native speakers) verified the extraction. The complete description of extracted data can be found in appendix (p3).

For seroprevalence, we differentiated between healthy and febrile populations and conducted random-effects meta-analysis to estimate the pooled seroprevalence and 95% confidence intervals (CI) for each country/region. For incidence, we integrated data from national disease surveillance systems and publications to understand regional and temporal patterns. Mortality data were stratified by patient cohorts, namely: inpatients that is, those which were admitted to hospital), admission not specified (i.e., the study did not state whether patients were admitted to hospital) and outpatients (i.e., those which did not attend or were not admitted to hospital), and we synthesized mortality rates along with DALYs, and economic impacts. A random-effects meta-analysis of risk factors was conducted to identify and quantify the major contributors related to scrub typhus. Each of these analytical components is detailed extensively in the appendix (p3-4). The risk of bias assessment form is in the appendix (Table S11).

## Results

**Figure 1:**
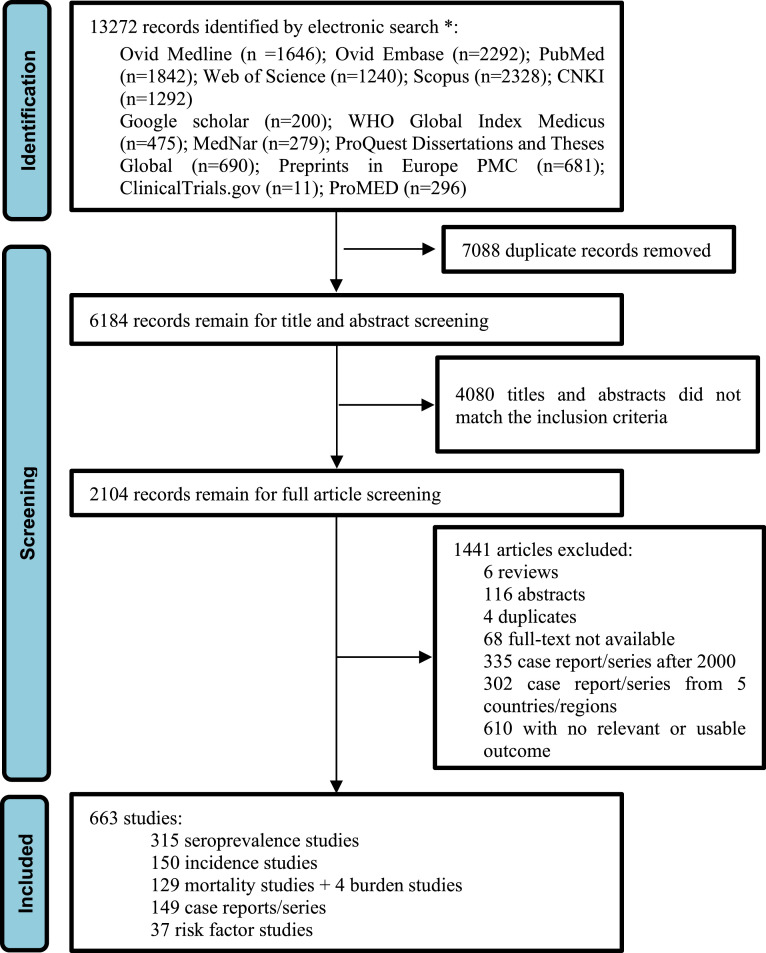
PRISMA diagram of study selection. *We chose the first 200 records from the google scholar search results.

The multi-database literature search identified 13,272 records, with 6184 unique records identified after removal of duplicates. 4080 articles were not considered relevant after title and abstract screening, leaving 2104 articles. Following full text screening, 663 articles were included comprising 315 that reported the seroprevalence of scrub typhus, 150 that reported incidence, 131 that reported mortality, 149 case reports and case series, 37 that investigated risk factors (Figure 1). The results from every database and the publication year of all eligible articles are provided in the appendix (Table S2 and Figure S1)***.*** These included papers had a mean quality score of 7.56 and the results of detailed quality assessments are in the appendix (Table S12).

### Seroprevalence

Reported pooled seroprevalences as the result of a meta-analysis, were 10.73% (95%CI 9.47-12.13%) from 99 521 participants in 109 studies in 22 countries/regions among healthy individuals and 22.58% (95%CI: 20.55-24.76%) among 178,684 febrile patients from 223 studies in 21 countries/regions (Table 1). Locations are shown in Figure S2 and full list of included publications is in appendix (Table S5).

**Table 1:** Reported scrub typhus seroprevalence rates in healthy and febrile cohorts by country/region.

Country/Region	Seroprevalence among healthy populations^a^					Seroprevalence among febrile patients^b^				
	No. studies	n/N	n/N, %	Median, % (range)	Pooled seroprevalence, % (95%CI)	No. studies	n/N	n/N, %	Median, % (range)	Pooled seroprevalence, % (95%CI)
**Total**	109	12,144/99521	12.20%	9.44 (0.00-84.00)	10.73 (9.47-12.13)	223	30,631/178684	17.14%	20.00 (0.00-100.00)	22.58 (20.55-24.76)
Australia	4	62/1438	4.31%	2.90 (0.00-21.77)	3.65 (1.88-6.96)	1	24/920	2.61%	2.60 (2.60-2.60)	2.61 (1.75-3.86)
Bangladesh	-	-	-	-	-	4	212/1529	13.87%	17.00 (0.33-17.50)	8.35 (1.60-33.84)
Bhutan	1	195/864	22.57%	20.35 (4.60-42.60)	19.87 (11.56-32.01)	3	243/1341	18.12%	58.00 (6.70-75.00)	43.40 (12.95-79.81)
Cambodia	1	7/282	2.48%	2.50 (2.50-2.50)	2.48 (1.19-5.11)	3	197/3533	5.58%	4.70 (3.90-7.80)	5.38 (3.49-8.20)
Chile	1	5/1302	0.38%	0.40 (0.00-0.70)	0.45 (0.20-1.04)	-	-	-	-	-
Djibouti	1	3/49	6.12%	6.00 (6.00-6.00)	6.12 (1.99-17.33)	-	-	-	-	-
Honduras	1	56/1000	5.60%	5.60 (5.60-5.60)	5.60 (4.33-7.21)	-	-	-	-	-
India	14	3170/14819	21.39%	20.70 (0.52-72.50)	16.04 (10.55-23.64)	103	12653/68564	18.45%	22.35 (0.00-82.60)	20.98 (18.08-24.20)
Indonesia	6	113/1640	6.89%	7.30 (1.30-20.00)	6.99 (4.15-11.55)	3	46/2298	2.00%	2.90 (0.00-9.30)	3.07 (1.70-5.48)
Iraq	-	-	-	-	-	1	14/91	15.38%	15.38 (15.38-15.38)	15.38 (9.33-24.32)
Japan	5	1777/5950	29.87%	19.40 (2.20-84.00)	22.43 (15.95-30.59)	3	490/1544	31.74%	57.14 (8.30-78.26)	45.32 (26.34-65.77)
Kenya	-	-	-	-	-	2	88/1765	4.99%	5.00 (0.00-12.00)	4.96 (3.06-7.95)
Laos	1	406/2002	20.28%	20.30 (20.30-20.30)	20.28 (18.58-22.10)	8	1778/15184	11.71%	6.00 (2.60-16.00)	6.11 (4.01-9.22)
Mainland China	45	2774/25626	10.82%	6.39 (0.00-76.47)	8.30 (6.93-9.92)	23	2669/18331	14.56%	9.36 (0.00-96.04)	14.97 (10.62-20.69)
Malaysia	7	608/3100	19.61%	16.40 (0.00-81.00)	18.42 (12.10-27.02)	7	1458/5845	24.94%	27.00 (0.00-84.80)	28.47 (16.34-44.78)
Myanmar	-	-	-	-	-	1	134/700	19.14%	11.00 (0.00-59.00)	12.18 (3.62-33.91)
Nepal	1	19/188	10.11%	10.11 (10.11-10.11)	10.11 (6.54-15.30)	14	2780/13420	20.72%	21.80 (1.50-60.00)	20.20 (15.40-26.04)
Palau	1	90/847	10.63%	23.07 (2.02-54.24)	15.67 (2.77-54.76)	-	-	-	-	-
Papua New Guinea	1	39/140	27.86%	25.00 (11.80-70.00)	28.13 (16.35-43.94)	-	-	-	-	-
Peru	-	-	-	-	-	1	60/1124	5.34%	5.30 (5.30-5.30)	5.34 (4.17-6.82)
Sao Tome and Principe	1	311/1950	15.95%	16.00 (5.80-20.40)	13.69 (8.07-22.28)					
Saudi Arabia	-	-	-	-	-	1	14/455	3.08%	3.08 (3.08-3.08)	3.08 (1.83-5.13)
Solomon Islands	1	161/335	48.06%	37.00 (21.00-81.00)	42.19 (28.29-57.44)	-	-	-	-	-
South Korea	2	56/817	6.85%	13.55 (4.90-22.20)	10.43 (2.24-37.16)	11	4612/22331	20.65%	40.00 (0.00-100.00)	40.37 (35.81-45.11)
Sri Lanka						6	304/1284	23.68%	38.97 (5.70-90.62)	33.56 (13.63-61.79)
Taiwan	1	23/517	4.45%	2.50 (0.00-12.00)	4.09 (1.71-9.43)	4	732/3970	18.44%	31.37 (11.38-85.00)	36.55 (9.78-75.37)
Thailand	11	1995/28065	7.11%	7.30 (0.00-69.00)	7.97 (6.10-10.35)	18	1225/9849	12.44%	15.00 (0.49-45.77)	11.50 (7.51-17.22)
Vanuatu	1	13/72	18.06%	18.00 (18.00-18.00)	18.06 (10.78-28.66)	-	-	-	-	-
Vietnam	3	261/8518	3.06%	1.10 (0.00-3.50)	1.26 (0.29-5.27)	6	898/4606	19.50%	11.60 (2.90-40.90)	13.31 (4.99-30.97)

*Notes:* In this table, 'n' represents the number of positive cases, and 'N' represents the total number of participants included in each study.

Pooled seroprevalence results were generated by random-effect meta-analysis methods and were weighted by numbers of participants.

a Healthy populations included those at high risk of scrub typhus, e.g. agricultural workers, farmer, soldiers.

b Febrile patients included any patients with a fever including acute undifferentiated fever, pyrexia of unknown origin or acute encephalitis syndrome.

Among healthy people, Solomon Islands had the highest pooled seroprevalence of 42.19% (95%CI: 28.29-58.44%). Papua New Guinea, Laos, and Japan also reported high pooled seroprevalences of 28.13%, 20.28%, and 22.43% respectively. Mainland China had pooled seroprevalence of 8.30% in 25,626 people. Sao Tome and Principe and Djibouti had pooled seroprevalences of 13.69% and 6.12% respectively, while in Chile it was 0.45% and in U.S. Military Personnel in Honduras 5.60% (Table 1).

In febrile patients, India contributed the most studies (N = 103) with pooled seroprevalence of 20.98% (95%CI: 18.08-24.20%). South Korea had pooled seroprevalence among febrile patients of 40.37% (95%CI: 35.81-45.11%) from nine studies, and Japan 45.32% in three study. In Africa, two studies in Kenya had pooled seroprevalence of 4.96%. In Peru, Iraq, and Saudi Arabia pooled seroprevalences were 5.34%, 15.38%, and 3.08% respectively. The age and gender specific results can be found in appendix (Figure S3).

Among diagnostic methods employed across the seroprevalence studies, the enzyme-linked immunosorbent assay (ELISA) emerged as the predominant method, accounting for 35% of all seroprevalence articles (Figure S4). The indirect immunofluorescence assay (IFA) test was also prominently featured, being referenced in 28% of the papers. Other techniques, including the indirect immunoperoxidase (IIP) assay, and hemagglutination tests, were cited less frequently. It is noteworthy that 17.62% of studies utilized a combination of diagnostic methods to ascertain positivity. IFA was the most used method in studies conducted in mainland China, found in 50.85%, while ELISA was cited in 15.25%. In India, ELISA was the primary method, mentioned in 70.37% of studies and IFA in only 1.23%. For studies in Thailand, ELISA was used in 25.64% and IFA in 23.08% of studies.

### Incidence

Currently, only five countries/regions—Japan, mainland China, South Korea, Taiwan, and Thailand—have designated scrub typhus as a notifiable disease in their national surveillance systems. Bhutan had included it in 2010 but removed it in 2016.

**Figure 2:**
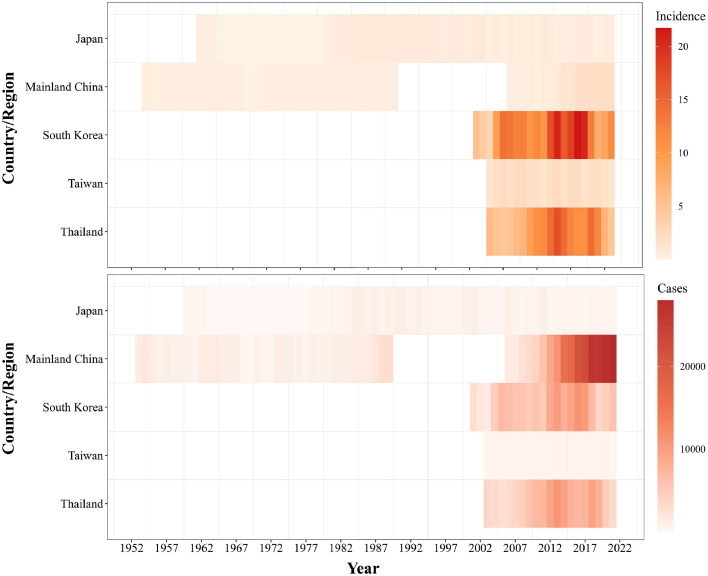
Reported annual scrub typhus incidence rate and case number in five countries/regions from 1952 to 2022. The top heatmap displays incidence rates per 100,000 population and the bottom heatmap shows total case numbers, with color intensities corresponding to the magnitude.

The incidence data from these five countries/regions suggest unique epidemiological patterns for each region (Figure 2). Mainland China reported the highest annual number of cases with 26 675 in 2018. South Korea (21.68 per 100 000 in 2016) and Thailand (17.09 per 100 000 in 2013) had the highest incidence rates. Japan's data from 1960-2022 began with 64 cases in 1960, peaking at 957 in 1984 and thereafter, annual incidence stayed relatively low. Mainland China showed significant fluctuations from 1952 to 1989, with a marked increase in case counts by the mid-1950s, variable trends in the subsequent decades, and a sharp rise post-2006. By 2019, mainland China reported 26 369 cases. For the period 2003 to 2021, Taiwan's annual case numbers remained relatively stable, ranging from 286 to 541 cases. South Korea's reported cases surged from 2637 in 2001 to 11,102 by 2016. Thailand saw a rise in the 2010s, peaking in 2013, followed by a decline. The full list of included publications is provided in appendix (Table S6).

### Case reports/series

India had the most published case reports at 102 with 241 cases and an additional 18 case series detailing 99 cases. Following India, Nepal published five case reports and three case series, with five and 27 cases, respectively. Australia, Bhutan, and Sri Lanka also reported multiple case reports/series. Countries or regions with established surveillance systems, where scrub typhus is prominently recognized, were excluded from this analysis to focus on areas where the disease's reporting may be emerging or sporadically highlighted. A location map of all case reports and case series is in Figure S2 the list of included publications is in appendix (Tables S7, S8).

### Mortality

Based on the data collected from the 129 studies which reported mortality, distinct patterns were evident across different healthcare and geographic settings (Table 2). In the context of hospital inpatient settings from 98 studies, out of 15,442 cases, 928 resulted in death, with a median (range) mortality of 5.00% (0-56.00%). Among inpatients, the case fatality rate was the highest in India, with a rate of 8.05% (0-33.33%), based on data from 64 studies encompassing 9049 cases. Broadening the scope to include all patients for which admission status was not reported, there were 221 deaths among 3325 cases, with a median (range) reported mortality rate of 6.70% (0-33.33%). This category combines outpatients, who might have had milder symptoms and who were tested or treated, with inpatients whose status was not explicitly detailed in some studies. India still had the highest combined mortality rate of 6.67% (0-32.76%), synthesized from 19 studies comprising a total of 2557 cases. Among outpatients there were 14 deaths from 382 cases, along with a reported median mortality rate of 2.17% ranging from 0 to 22.22%. These numbers emanate from community surveys and encapsulate those who might not have been under formal medical care. Within this group, the data from mainland China reveals a mortality rate of 3.92% across 204 cases based on five studies. Temporal changes and full list of included publications is in appendix (Figure S5, Table S9).

**Table 2:** Reported scrub typhus mortality across countries/regions.

Country/Region	Hospital inpatient mortality^a^				Admission not specified mortality				Outpatient mortality			
	No. studies	Death/Case (ratio)	Death/ Case ratio	Median (range)	No. studies	Death/Case	Death/ Case ratio	Median (range)	No. studies	Death/Case	Death/ Case ratio	Median (range)
**Total**	**98**	**928/15442**	**6.01%**	**5.00% (0.00%-56.00%)**	**24**	**221/3325**	**6.65%**	**6.70% (0.00%-33.33%)**	**9**	**14/382**	**3.66%**	**2.17% (0.00%-22.22%)**
Australia	-	-	-	-	-	-	-	-	1	2/118	1.69%	2.00% (2.00%-2.00%)
Bhutan	1	2/460	0.43%	0.43% (0.43%-0.43%)	-	-	-	-	1	2/9	22.22%	22.22% (22.22%-22.22%)
Cambodia	1	1/95	1.05%	1.05% (1.05%-1.05%)	-	-	-	-	-	-	-	-
India	64	729/9049	8.06%	8.05% (0.00%-33.33%)	19	162/2557	6.34%	6.67% (0.00%-32.76%)	1	2/38	5.26%	5.30% (5.30%-5.30%)
Indonesia	1	0/10	0.00%	0.00% (0.00%-0.00%)	-	-	-	-	-	-	-	-
Laos	1	1/63	1.59%	1.50% (1.50%-1.50%)	-	-	-	-	-	-	-	-
Mainland China	13	74/2583	2.86%	2.45% (1.00%-13.00%)	-	-	-		5	8/204	3.92%	2.17% (0.00%-17.65%)
Malaysia	1	1/315	0.32%	0.32% (0.32%-0.32%)	-	-		-	-	–	-	-
Nepal	4	59/741	7.96%	11.95% (1.79%-56.00%)	-	-	-	-	-	-	-	-
Solomon Islands	1	0/9	0.00%	0.00% (0.00%-0.00%)	-	-	-	-	-	-	-	-
South Korea	3	29/877	3.31%	6.10% (0.78%-10.00%)	-	-	-	–	-	-	-	-
Taiwan	1	1/39	2.56%	3.00% (3.00%-3.00%)	1	1/15	6.67%	6.67% (6.67%-6.67%)	-	-	-	-
Thailand	4	22/610	3.61%	3.22% (1.52%-6.20%)	3	12/190	6.32%	6.70% (0.00%-16.67%)	-	-	-	-
Vietnam	3	9/591	1.52%	1.20% (0.40%-4.85%)	-	-	-	-	-	-	-	-

a *Note:* Some studies only reported on admitted patients with severe conditions, while most reported on all admitted patients

Only two studies were found in which the DALYs for scrub typhus were assessed, both of which focused on Shandong Province in mainland China. Between 2006 and 2012, DALYs in Shandong peaked at 117.85, while Laiwu city recorded a maximum of 13 DALYs and a DALY rate of 1.06 per 100,000 in 2012, which was one-third of the 3.32 DALYs rate for dengue in China in the same year [[17], [18], [19]]. Further economic studies from mainland China highlighted the disease's financial toll. Hospitalization costs per person reached a maximum of $1 911.9 in Kunming, with an outbreak in Jinjiang costing an average of $1 088.5 per person [20,21]. This underscores the significant economic and health implications of scrub typhus.

### Risk factors

Thirty-seven included studies assessed risk factors for scrub typhus, comprising case-control studies (N = 28) and cross-sectional studies (N = 9). The list of included publications is in appendix (Table S10). From those studies, 12,125 scrub typhus cases were identified across 9 countries, with mainland China and Taiwan presenting the highest numbers. Twenty-one exposures were found to be significantly associated with scrub typhus odds/risk in two or more studies and were assessed in our meta-analysis. Predominant risk factors included agricultural work, specific vegetation exposure, other outdoor activities, risky personal health habits, and exposure to rodents, livestock, or poultry (Table 3). Notably, agricultural work and specific vegetation were reported in 19 and 18 studies as significant risk factors with pooled ORs of 2.8 (2.1-3.6) and 2.2 (1.4-3.4), respectively. Wearing of protective clothing outdoors was the most significant protective factor with a pooled OR of 0.6 (0.4-0.7). The forest plots for those top ten exposures can be found in Figure S6.

**Table 3:** Exposures associated with scrub typhus in case-control or cross-sectional studies.

Exposure	No. studies	Pooled odds ratio (95%CI)	p value	I^2^, %	Q
Agricultural or field work	19	2.8 (2.1- 3.6)	<0.001	77%	0
Specific vegetation^a^	18	2.2 (1.4-3.4)	0.001	92%	0
Other outdoor activities	8	2.4 (1.8-3.2)	<0.001	62%	0
Risky personal health habits^b^	7	2.2 (1.7-2.8)	<0.001	0%	0.7
Rodents	7	2.8 (1.6-4.8)	<0.001	83%	0
Poultry or livestock	6	1.6 (0.8-3.1)	0.151	88%	0
Age	5	1.6 (1-2.6)	0.054	98%	0
Pets	4	3.6 (2.3-5.5)	<0.001	0%	0.5
Protective clothing or equipment while outdoors	4	0.6 (0.4-0.7)	<0.001	21%	0.4
Female	4	1.5 (1.2-2)	0.002	67%	0
Living at the edge of village	3	1.3 (0.6-3.1)	0.513	86%	0
Awareness	3	0.6 (0.1-3.6)	0.549	91%	0
Low income	3	2.3 (1-5.3)	0.045	90%	0
Travel history	3	2.2 (0.3-14.2)	0.425	86%	0
Humid living conditions	2	4.6 (2.3-9.4)	<0.001	0%	0.6
Wash and change clothes after work	2	0.3 (0.2-0.5)	<0.001	0%	0.5
Close to fields	2	1.7 (1.2-2.3)	0.004	0%	0.7
Bite from unknown insect	2	2.8 (1.1-6.7)	0.025	75%	0
Living in cottage or bungalow	2	5.5 (2.5-12.2)	<0.001	0%	0.7
Water body	2	3.2 (1.6-6.4)	0.001	0%	0.8
Living in rural area	2	1.3 (1.1-1.6)	0.01	0%	1

a Specific vegetation included weeds, grass, bushes, wood piles, firewood, straws, lawn, and shrubs.

b Risky personal health habits included squatting to defecate or urinate, not wearing a shirt at home-open defecation, not changing undergarments or clothes, dry clothes in the grass and sitting/laying directly on household floor.

## Discussion

Our review provides the first summary estimates of the seroprevalence, incidence, and mortality rates for scrub typhus, both globally and regionally, together with a comprehensive review of individual behavioral risk factors. This comprehensive review and analysis make a significant contribution to the existing body of knowledge on scrub typhus.

Compared with previous systematic reviews [11,12], our study included more studies and estimated reported seroprevalence by random-effects meta-analysis, considering variability of the data sets. Worldwide, we found that 10.09% (95%CI: 8.85-11.50%) of individuals in healthy populations and 23.11% (95%CI: 20.80-25.61%) of febrile patients were seropositive for scrub typhus. We noted high seroprevalence observed among healthy populations in countries like Papua New Guinea (27.86%) and Japan (22.43%). But two studies conducted in Japan with 507 military personnel in combat training fields [22] and 257 agricultural workers [23] may raise the estimated number as those populations are at high risk of acquiring scrub typhus. India's largest number of studies painted a complicated picture, with seroprevalence rates ranging from 0.00% to a staggering 82.60%. Such variations likely arose from different levels of exposure and diagnostic procedures. The higher seroprevalence rates among fever patients in South Korea and Japan, 40.00% and 61.84% respectively, indicated the high burden in Asia. Iraq had a seroprevalence rate of 15.38%, hinting at emerging risks outside the traditional tsutsugamushi triangle.

However, all the above seroprevalence results are greatly affected by the diagnostic methods and positivity definitions used in the included articles. Scrub typhus lacks clinically distinctive clinical features, and the results of laboratory tests play a decisive factor in case determination. The IFA, despite being an imperfect gold standard [24], was commonly used, particularly in mainland China in our review. However, due to IFA's cost, and requirement for more sophisticated facilities and training, the ELISA has become a preferred method due to its convenience, lower cost and good performance, being the most common in serological studies and particularly dominant in Indian research [25]. Some older, but still commonly used, diagnostic methods such as the Weil-Felix OX-K test lack specificity and sensitivity [24], making the results of some articles unreliable. Complicating the situation further are differences in wide range of cutoff optical density values in commonly used ELISAs [26], the inconsistent use of antigens and the variability of diagnostic cutoff antibody titers, which are often locally validated and vary greatly between studies [27]. Such heterogeneity in diagnostic methods and cutoff points contribute greatly to the uncertainty and variability of results between studies and highlights the need for in-depth research and standardization of diagnostic approaches. Additionally, because the antibody responses to the bacterial antigens in patients are generally short-lived [28], the seroprevalence result potentially underestimated the number of people who have been previously infected.

The median mortality rates were 5.00% in hospital inpatients, 6.70% for patients for which admission status was not specified, and 2.17% of outpatients. These results reflect the situation in the context of the availability of appropriate antibiotic treatment and modern medical care, albeit in largely low-resource settings. That it is not that different from the previously reported untreated median mortality rate of 6% is worrying, and may reflect lack of timely diagnosis, lack of awareness of the disease, and consequent lack of timely and appropriate antibiotic administration [10]. The variability in reported mortality across countries is reflective of the different level of access to, and quality of, healthcare systems. Countries like India, with conspicuously elevated mortality, emphasize the pressing need for bolstered healthcare infrastructure that facilitates early diagnosis and prompt and appropriate treatment. Our detailed categorization, delineating inpatients, combined patient groups, and community populations, provides a granular insight into the disease's mortality spectrum. In the broader context of global health, our findings substantiate the postulation that countries endowed with established and easily accessible health systems invariably fare better in curtailing mortality.

We synthesized and analyzed reported incidence over 70 years (1952-2022) from five countries or regions. Data from mainland China, with the highest numbers of reported cases in 2018 and 2019, demonstrates the immense burden of scrub typhus within its borders. However, focusing solely on raw case numbers may be misleading. When scaled by population size, South Korea and Thailand had the highest incidence rates. The surge in South Korea's cases over a 15-year span could be caused by increased transmission or enhanced diagnostic efforts or both and improved levels of awareness of the disease. Thailand's trajectory – a rise followed by a decline – might indicate the success of interventions post-peak or changing environmental or host-vector interactions. Incidence of scrub typhus could also vary depending on the strength of the surveillance system in the country (passive or active, mandatory case reporting or not) and on the case definition used, whether it is based on isolation of bacteria, detection by PCR or immunological testing combined with clinical features. These diverse patterns highlight the heterogeneous nature of scrub typhus transmission and manifestations across different Asian countries and emphasize the need for sustained ongoing high-quality surveillance and establishing detection systems in other high-risk areas.

The main exposures contributing to increased scrub typhus risks were agricultural or field work, exposure to specific vegetation, other outdoor activities, and risky personal health habits. This underscores the pivotal role of environmental exposure in the disease epidemiology. Agricultural or field work emerged as a pronounced risk, aligning with prevalent occupations in the regions observed [29,30]. Although various vegetation types were reported as associated with the disease, the differences in study designs across the included studies prevented a direct comparison of the strength of association for each type of vegetation. Concurrently, protective measures like the use of appropriate clothing outdoors were shown to be mitigating factors, signifying a potential avenue for preventive strategies [[31], [32], [33]]. The meta-analysis highlighted other significant risk factors like the presence of rodents, pets, humid living conditions, and residing in specific types of housing such as cottages or bungalows [34].

Our review addresses key gaps in the understanding of scrub typhus. We provide, for the first time, a global quantification of its estimated seroprevalence and highlight evolving patterns by analyzing its incidence over a long-time span. The study sheds light on mortality, both globally and regionally, while underlining the roles of individual behaviors increasing risk of disease acquisition. We encourage further studies to validate and potentially standardize diagnostic tools and criteria, and to improve understanding of vector and transmission dynamics. Advancing research in these domains through international collaboration will endow policymakers with robust and precise data, thereby refining health interventions for affected communities.

However, this study has multiple limitations. We were unable to include many relevant conference abstracts in this review and meta-analysis as insufficient detail was provided in the abstracts. We were also unable to include some studies published in languages other than English, Chinese, Korean or Japanese, possibly introducing bias. Additionally, we excluded studies that were ambiguous in determining the status of the tested population or those that did not describe the location of infection, though these provided valuable epidemiological insights for certain regions. Furthermore, due to the diverse nature of the included studies, we did not exclude papers with low quality, as we aimed to preserve all pertinent data on this neglected disease without losing valuable insights. Moreover, there was high heterogeneity across most of our results, which arise from inevitable differences in study design, population characteristics, and diagnostic methods.

## Declaration of competing interest

The authors declare that they have no known competing financial interests or personal relationships that could have appeared to influence the work reported in this paper.

## Appendix Supplementary materials

Image, application 1
