# Supplementary material for: Global and regional seroprevalence, incidence, mortality of, and risk factors for scrub typhus: A systematic review and meta-analysis
Source: Int J Infect Dis. 2024 Sep;146:None. doi: 10.1016/j.ijid.2024.107151 (PMC11310856; doi:10.1016/j.ijid.2024.107151)
Supplement: Supplementary file 1 [file mmc1.docx]

**Supplementary appendix**

**Global and regional seroprevalence, incidence, mortality of, and risk factors for scrub typhus: a systematic review and meta-analysis**

Qian Wang ^1,2,*^, Tian Ma ^3,4^, Fangyu Ding ^3,4^, Ahyoung Lim ^5^, Saho Takaya ^5^, Kartika Saraswati ^1,6,7^, Benn Sartorius ^2,8,9^, Nicholas P.J. Day ^1,2^, Richard J Maude ^1,2,10^

^1^ Mahidol Oxford Tropical Medicine Research Unit, Faculty of Tropical Medicine, Mahidol University, Bangkok, Thailand.

^2^ Centre for Tropical Medicine and Global Health, Nuffield Department of Medicine, University of Oxford, Oxford, UK.

^3^ Institute of Geographic Sciences and Natural Resources Research, Chinese Academy of Sciences, Beijing, China.

^4^ College of Resources and Environment, University of Chinese Academy of Sciences, Beijing, China.

^5^ London School of Hygiene & Tropical Medicine, London, UK.

^6^ Saw Swee Hock School of Public Health, National University of Singapore, Singapore, Singapore.

^7^ Oxford University Clinical Research Unit Indonesia, Faculty of Medicine, Universitas Indonesia, Jakarta, Indonesia.

^8^ Centre for Clinical Research (UQCCR), Faculty of Medicine, University of Queensland, Brisbane, Australia.

^9^ Department of Health Metric Sciences, School of Medicine, University of Washington, Seattle, USA.

^10^ The Open University, Milton Keynes, UK

* Corresponding author

**Contents**

[Appendix method 3](#_Toc167381491)

[Data extraction 3](#_Toc167381492)

[Data analysis 3](#_Toc167381493)

[Geocoding 4](#_Toc167381494)

[Appendix Table S1: Full search strategy for each database. 5](#_Toc167381495)

[Appendix Table S2: Search databases and results. 7](#_Toc167381496)

[Appendix Table S3: PRISMA Checklist. 8](#_Toc167381497)

[Appendix Figure S1: Publication years of included articles. 11](#_Toc167381498)

[Appendix Figure S2: Location of included articles reported prevalence and case report and case series. 12](#_Toc167381499)

[Appendix Figure S3: Reported age and sex-specific seroprevalence by included study.. 12](#_Toc167381500)

[Appendix Table S4: Gender-Specific Prevalence among healthy populations and febrile patients in countries/regions. 14](#_Toc167381501)

[Appendix Figure S4. Diagnostic test method used in seroprevalence studies by country/region. 15](#_Toc167381502)

[Appendix Figure S5: Temporal changes in mortality. 16](#_Toc167381503)

[Appendix Figure S6: Forest plot for reported exposures associated with scrub typhus in case-control or cross-sectional studies. 16](#_Toc167381504)

[Appendix Table S5: Characteristics of included seroprevalence studies, in order of country/region and published year (n=315). 19](#_Toc167381505)

[Appendix Table S6: Characteristics of included incidence studies, in order of country/region and published year (n=150). 44](#_Toc167381506)

[Appendix Table S7: Case reports and case series of scrub typhus since 2000. 58](#_Toc167381507)

[Appendix Table S8: Characteristics of included case reports and case series, in order of country/region and published year (n=149). 59](#_Toc167381508)

[Appendix Table S9: Characteristics of included mortality studies, in order of country/region and published year (n=129). 72](#_Toc167381509)

[Appendix Table S10: Characteristics of included risk factor studies, in order of country/region and published year (n=37). 82](#_Toc167381510)

[Appendix Table S11: Quality assessment scale for rating the risk of bias. 85](#_Toc167381511)

[Appendix Table S12: Quality scores for assessing the risk of bias in the included articles. 86](#_Toc167381512)

[Reference 111](#_Toc167381513)

# Appendix method

## Data extraction

The extracted data included: article and research information (authors, publication year, publication language, country of first author affiliation, geographical region of the study, study design, investigation date); characteristics of the study participants (sample size, age, gender, symptoms, diagnostic method and definition of disease); epidemiological outcomes (prevalence/incidence/number of cases/deaths due to scrub typhus, predicted/potential burden caused, location conducted, specific period of outcome measure); risk factors outcomes (definition, source, spatial and temporal resolution, association/relationship, statistical indicators and results).

## Data analysis

A synthesis of the global prevalence of scrub typhus was performed. The variability in seroprevalence data was significant, stemming from heterogenous population demographics and diagnostic methods used. To address the variations and ensure clarity in our findings, we categorized all seroprevalence studies into two groups: those focused on healthy populations and those focused on febrile patients. By consolidating data from these studies, we compiled comprehensive statistics on the total numbers of participants tested, the numbers of positive cases, and the resultant seroprevalences and summarized the reported seroprevalence values, capturing both their median and range. Multivariable logistic regressions were performed based on the numbers of participants and the corresponding numbers of cases in the included articles using random-effects meta-analysis models. The seroprevalences with respect to age and gender were analyzed using results from a subset of studies that provided a breakdown by specific demographic characteristics. Given the variations in diagnostic criteria for scrub typhus across different countries and regions, we also presented the frequency of different testing methodologies and how these diagnostic methods were distributed across different countries, offering a nuanced understanding of regional diagnostic preferences and practices.

Similarly for the incidence data, the spatial scope and temporal span represented in the final incidence data from the studies diverged considerably due to the varied research questions and methodologies used. This was despite most incidence data being derived from numbers reported by national disease surveillance systems. Subsequently, we made concerted efforts to source the most detailed location data and extensive temporal data available directly from disease surveillance systems in the five countries or regions where scrub typhus is classified as a notifiable disease. We combined this information with population data from the UN and World Bank to derive incidence rates. By aggregating these two types of datasets, a more comprehensive and nuanced analysis of national scrub typhus incidence was possible.

To offer a comprehensive understanding of scrub typhus distribution since the turn of the century, we curated case reports and case series articles from the year 2000 onwards. Recognizing the potential variability in disease manifestations, treatment modalities, and healthcare infrastructure across regions, we stratified our analysis by country. For each country, we enumerated both the number of articles published and the cumulative reported case count, thereby providing a juxtaposed view of research interest against the actual disease burden.

We stratified mortality studies based on three patient cohorts: inpatients (i.e. those which were admitted to hospital), admission not specified (i.e. the study did not state whether patients were admitted to hospital) and outpatients (i.e. those which did not attend or were not admitted to hospital). For each of these cohorts, we collated the number of patients, documented fatalities, and subsequently derived the mortality ratio. We synthesized reported mortality rates, calculating the median and range. Narrative synthesis was used to summarize studies providing DALYs, YLDs and economic analysis with the results presented descriptively.

We conducted a meta-analysis on the risk factors related to scrub typhus, which were explored in individual-based and population-based observational studies. The pooled odds ratios (OR) and 95% confidence intervals for exposures that ranked in the top-ten in all the individual-based observational studies were calculated based on the number of participants and the corresponding number of cases in the included articles using random-effects models. Heterogeneity of case-control and cross-sectional studies were assessed using the Cochran Q-statistic and I^2^ statistic.

## Geocoding

To acquire the most precise geographical data on the location of cases and fatalities, we scrutinized the full text of the included records. The location information within these articles was typically provided in two forms: either as specific health facilities such as hospitals, clinics, or laboratories where the studies were conducted, or as administrative units like counties or cities where incidence, seroprevalence or cases were reported.

In the case of the former, we extracted the locations of the facilities. When facility details were not provided, we relied on the affiliation of the author who provided the data to determine the location. When no author contribution was specified, we resorted to the address of the corresponding author. In instances where multiple site names were provided, we documented all of them. If it was merely mentioned that the study took place at various locations without naming each one, we recorded the encompassing administrative level. In cases labeled as 'imported,' we documented the potential original location. All study locations were subjected to geocoding for the purpose of location tracking and mapping. When exact coordinates were provided, we utilized them directly. In all other cases, the Google Maps API was employed to obtain exact coordinates, using the 'ggmap' package in R software (version 4.0) to facilitate the process. Instances of geocoding failure led us to seek site information online using various resources, and any spelling variations of place names were corrected based on similarities, followed by manual geocoding using Google Maps. As for the latter scenario where location information was given as administrative units, we noted down any available combination of address, city, state, province, and country.

All the extracted results were aggregated with the reported case and death data from several countries’ disease surveillance systems to obtain a final comprehensive dataset. The Database of Global Administrative Areas (GADM version 4.1) was used to match and map data to geographic areas, and the spatial and temporal distribution of scrub typhus occurrence was generated in ArcGIS version 10.8. Geocoded results were used to produce global maps of reported seroprevalence and case reports/series.

# Appendix Table S1: Full search strategy for each database.

| **Database** | **Search string** |
| --- | --- |
| Ovid Medline | Medline (Ovid MEDLINE® Epub Ahead of Print, In-Process & Other Non-Indexed Citations, Ovid MEDLINE® Daily and Ovid MEDLINE®) 1946 to present  1 scrub typhus/  2 Orientia tsutsugamushi/  3 ("scrub typhus" or orientia or tsutsugamushi or "O. chiloensis" or "O. chuto" or "mite typhus" or "mite fever" or "Japanese river fever" or "Nippon river fever" or "Kedani fever" or "shichito fever" or "Akamushi disease" or "tropical typhus" or "Rickettsia tsutsugamushi" or "mite borne rickettsia" or "mite borne rickettsiosis" or "chigger borne rickettsia" or "chigger borne rickettsiosis" or "chigger borne typhus" or "Rickettsia orientalis" or "akamushi disease" or "XK typhus").ti,ab. 2858  4 1 or 2 or 3  5 epidemiology/  6 exp prevalence/  7 incidence/  8 risk factor/  9 (epidemiolog* or burden or distribution or prevalence or inciden* or frequen* or rate or occurren* or risk or case*).ti,ab.  10 Case Reports/  11 5 or 6 or 7 or 8 or 9 or 10  12 4 and 11 |
| Ovid Embase | Embase 1974 to present  1 scrub typhus/  2 Orientia tsutsugamushi/  3 ("scrub typhus" or orientia or tsutsugamushi or "O. chiloensis" or "O. chuto" or "mite typhus" or "mite fever" or "Japanese river fever" or "Nippon river fever" or "Kedani fever" or "shichito fever" or "Akamushi disease" or "tropical typhus" or "Rickettsia tsutsugamushi" or "mite borne rickettsia" or "mite borne rickettsiosis" or "chigger borne rickettsia" or "chigger borne rickettsiosis" or "chigger borne typhus" or "Rickettsia orientalis" or "akamushi disease" or "XK typhus").ti,ab. 3028  4 1 or 2 or 3  5 epidemiology/  6 exp prevalence/  7 incidence/  8 risk factor/  9 (epidemiolog* or burden or distribution or prevalence or inciden* or frequen* or rate or occurren* or risk or case*).ti,ab.  10 case report/  11 5 or 6 or 7 or 8 or 9 or 10  12 4 and 11 |
| PubMed | ("Scrub Typhus"[Mesh] OR scrub typhus[Title/Abstract] OR Orientia[Title/Abstract] OR Tsutsugamushi[Title/Abstract] OR O. chiloensis[Title/Abstract] OR O. chuto[Title/Abstract] OR mite typhus[Title/Abstract] OR mite fever[Title/Abstract] OR Japanese river fever[Title/Abstract] OR Nippon river fever[Title/Abstract] OR Kedani fever[Title/Abstract] OR shichito fever[Title/Abstract] OR Akamushi disease[Title/Abstract] OR tropical typhus[Title/Abstract] OR Rickettsia tsutsugamushi[Title/Abstract] OR mite borne rickettsia[Title/Abstract] OR mite borne rickettsiosis[Title/Abstract] OR chigger borne rickettsia[Title/Abstract] OR chigger borne rickettsiosis[Title/Abstract] OR chigger borne typhus[Title/Abstract] OR Rickettsia orientalis[Title/Abstract] OR akamushi disease[Title/Abstract] OR XK typhus[Title/Abstract]) AND ((epidemiolog* [Title/Abstract] OR burden[Title/Abstract] OR distribution[Title/Abstract] OR prevalence[Title/Abstract] OR inciden*[Title/Abstract] OR frequen*[Title/Abstract] OR rate[Title/Abstract] OR occurren*[Title/Abstract] OR risk[Title/Abstract] OR "Case Reports" [Publication Type] OR case*[Title/Abstract])) |
| Web of Science | (AB=((scrub typhus) OR Orientia OR Tsutsugamushi OR (O. chiloensis) OR (O. chuto) OR (mite typhus) OR (mite fever) OR (Japanese river fever) OR (Nippon river fever) OR (Kedani fever) OR (shichito fever) OR (Akamushi disease) OR (tropical typhus) OR (Rickettsia tsutsugamushi) OR (mite borne rickettsia) OR (mite borne rickettsiosis) OR (chigger borne rickettsia) OR (chigger borne rickettsiosis) OR (chigger borne typhus) OR (Rickettsia orientalis) OR (akamushi disease) OR (XK typhus))) AND (AB=((epidemiolog*) OR burden OR distribution OR prevalence OR (inciden*) OR (frequen*) OR rate OR (occurren*) OR risk OR (case*))) |
| Scopus | ( TITLE-ABS-KEY ( "scrub typhus" OR orientia OR tsutsugamushi OR "O. chiloensis" OR "O. chuto" OR "mite typhus" OR "mite fever" OR "Japanese river fever" OR "Nippon river fever" OR "Kedani fever" OR "shichito fever" OR "Akamushi disease" OR "tropical typhus" OR "Rickettsia tsutsugamushi" OR "mite borne rickettsia" OR "mite borne rickettsiosis" OR "chigger borne rickettsia" OR "chigger borne rickettsiosis" OR "chigger borne typhus" OR "Rickettsia orientalis" OR "akamushi disease" OR "XK typhus" ) AND TITLE-ABS-KEY ( epidemiolog* OR burden OR distribution OR prevalence OR inciden* OR frequen* OR rate OR occurren* OR risk OR case*) ) |
| CNKI | 摘要: (恙虫病 + 丛林斑疹伤寒) * (分布 + 时空 + 流行 + 发生 + 负担 + 风险 + 病例) |
| Google scholar | ("scrub typhus"\|"orientia Tsutsugamushi")(epidemiology\|burden\|distribution\|prevalence\|incidence\|frequency\|rate\|occurrence\|risk\| case) |
| WHO Global Index Medicus | (tw:("scrub typhus" OR orientia OR tsutsugamushi OR "O. chiloensis" OR "O. chuto" OR "mite typhus" OR "mite fever" OR "Japanese river fever" OR "Nippon river fever" OR "Kedani fever" OR "shichito fever" OR "Akamushi disease" OR "tropical typhus" OR "Rickettsia tsutsugamushi" OR "mite borne rickettsia" OR "mite borne rickettsiosis" OR "chigger borne rickettsia" OR "chigger borne rickettsiosis" OR "chigger borne typhus" OR "Rickettsia orientalis" OR "akamushi disease" OR "XK typhus")) AND (tw:(epidemiolog* OR burden OR distribution OR prevalence OR inciden* OR frequen* OR rate OR occurren* OR risk OR case*)) |
| *MedNar* | *(“scrub typhus” OR Orientia OR Tsutsugamushi OR “O. chiloensis” OR “O. chuto” OR “mite typhus” OR “mite fever” OR “Japanese river fever” OR “Nippon river fever” OR “Kedani fever” OR “shichito fever” OR “Akamushi disease” OR “tropical typhus” OR “Rickettsia tsutsugamushi” OR “mite borne rickettsia” OR “mite borne rickettsiosis” OR “chigger borne rickettsia” OR “chigger borne rickettsiosis” OR “chigger borne typhus” OR “Rickettsia orientalis” OR “akamushi disease” OR “XK typhus”) AND (“epidemiolog*” OR burden OR distribution OR prevalence OR “inciden*” OR “frequen*” OR rate OR “occurren*” OR risk OR “case*”)* |
| *ProQuest Dissertations and Theses Global* | *("scrub typhus" OR orientia OR tsutsugamushi OR "O. chiloensis" OR "O. chuto" OR "mite typhus" OR "mite fever" OR "Japanese river fever" OR "Nippon river fever" OR "Kedani fever" OR "shichito fever" OR "Akamushi disease" OR "tropical typhus" OR "Rickettsia tsutsugamushi" OR "mite borne rickettsia" OR "mite borne rickettsiosis" OR "chigger borne rickettsia" OR "chigger borne rickettsiosis" OR "chigger borne typhus" OR "Rickettsia orientalis" OR "akamushi disease" OR "XK typhus") And (epidemiolog* OR burden OR distribution OR prevalence OR inciden* OR frequen* OR rate OR occurren* OR risk OR case*)* |
| *Preprints in Europe PMC* | *(ABSTRACT: (“scrub typhus” OR Orientia OR Tsutsugamushi OR “O. chiloensis” OR “O. chuto” OR “mite typhus” OR “mite fever” OR “Japanese river fever” OR “Nippon river fever” OR “Kedani fever” OR “shichito fever” OR “Akamushi disease” OR “tropical typhus” OR “Rickettsia tsutsugamushi” OR “mite borne rickettsia” OR “mite borne rickettsiosis” OR “chigger borne rickettsia” OR “chigger borne rickettsiosis” OR “chigger borne typhus” OR “Rickettsia orientalis” OR “akamushi disease” OR “XK typhus”)) AND (ABSTRACT: (“epidemiolog*” OR burden OR distribution OR prevalence OR “inciden*” OR “frequen*” OR rate OR “occurren*” OR risk OR case*))* |
| *ClinicalTrials.gov* | *Scrub typhus* |
| *ProMED* | *Scrub typhus* |

# Appendix Table S2: Search databases and results.

| **Database** | **Result number** |
| --- | --- |
| Ovid Medline | 1646 |
| Ovid Embase | 2292 |
| PubMed | 1842 |
| Web of Science | 1240 |
| Scopus | 2328 |
| CNKI | 1292 |
| Google scholar | The first 200 of 17100 |
| WHO Global Index Medicus | 475 |
| MedNar | 279 |
| ProQuest Dissertations and Theses Global | 690 |
| Preprints in Europe PMC | 681 |
| ClinicalTrials.gov | 11 |
| ProMED | 296 |
| **Total** | **13272** |

# Appendix Table S3: PRISMA Checklist.

| **Section and Topic** | **Item #** | **Checklist item** | **Location where item is reported** |
| --- | --- | --- | --- |
| **TITLE** | | |  |
| Title | 1 | Identify the report as a systematic review. | Page 1 |
| **ABSTRACT** | | |  |
| Abstract | 2 | See the PRISMA 2020 for Abstracts checklist. | Page 1 |
| **INTRODUCTION** | | |  |
| Rationale | 3 | Describe the rationale for the review in the context of existing knowledge. | Page 2 |
| Objectives | 4 | Provide an explicit statement of the objective(s) or question(s) the review addresses. | Page 2 |
| **METHODS** | | |  |
| Eligibility criteria | 5 | Specify the inclusion and exclusion criteria for the review and how studies were grouped for the syntheses. | Page 4 |
| Information sources | 6 | Specify all databases, registers, websites, organisations, reference lists and other sources searched or consulted to identify studies. Specify the date when each source was last searched or consulted. | Page 4 |
| Search strategy | 7 | Present the full search strategies for all databases, registers and websites, including any filters and limits used. | Page 4 |
| Selection process | 8 | Specify the methods used to decide whether a study met the inclusion criteria of the review, including how many reviewers screened each record and each report retrieved, whether they worked independently, and if applicable, details of automation tools used in the process. | Page 4 |
| Data collection process | 9 | Specify the methods used to collect data from reports, including how many reviewers collected data from each report, whether they worked independently, any processes for obtaining or confirming data from study investigators, and if applicable, details of automation tools used in the process. | Page 4 |
| Data items | 10a | List and define all outcomes for which data were sought. Specify whether all results that were compatible with each outcome domain in each study were sought (e.g., for all measures, time points, analyses), and if not, the methods used to decide which results to collect. | Page 4 |
|  | 10b | List and define all other variables for which data were sought (e.g., participant and intervention characteristics, funding sources). Describe any assumptions made about any missing or unclear information. | Page 4 |
| Study risk of bias assessment | 11 | Specify the methods used to assess risk of bias in the included studies, including details of the tool(s) used, how many reviewers assessed each study and whether they worked independently, and if applicable, details of automation tools used in the process. | NA |
| Effect measures | 12 | Specify for each outcome the effect measure(s) (e.g., risk ratio, mean difference) used in the synthesis or presentation of results. |  |
| Synthesis methods | 13a | Describe the processes used to decide which studies were eligible for each synthesis (e.g., tabulating the study intervention characteristics and comparing against the planned groups for each synthesis (item #5)). | Page 5 |
|  | 13b | Describe any methods required to prepare the data for presentation or synthesis, such as handling of missing summary statistics, or data conversions. | Page 5 |
|  | 13c | Describe any methods used to tabulate or visually display results of individual studies and syntheses. | Page 5 |
|  | 13d | Describe any methods used to synthesize results and provide a rationale for the choice(s). If meta-analysis was performed, describe the model(s), method(s) to identify the presence and extent of statistical heterogeneity, and software package(s) used. | Page 5 |
|  | 13e | Describe any methods used to explore possible causes of heterogeneity among study results (e.g., subgroup analysis, meta-regression). | Page 5 |
|  | 13f | Describe any sensitivity analyses conducted to assess robustness of the synthesized results. | Page 5 |
| Reporting bias assessment | 14 | Describe any methods used to assess risk of bias due to missing results in a synthesis (arising from reporting biases). | SI Table S11 |
| Certainty assessment | 15 | Describe any methods used to assess certainty (or confidence) in the body of evidence for an outcome. | Page 5 |
| **RESULTS** | | |  |
| Study selection | 16a | Describe the results of the search and selection process, from the number of records identified in the search to the number of studies included in the review, ideally using a flow diagram. | Page 6 |
|  | 16b | Cite studies that might appear to meet the inclusion criteria, but which were excluded, and explain why they were excluded. | NA |
| Study characteristics | 17 | Cite each included study and present its characteristics. | appendix |
| Risk of bias in studies | 18 | Present assessments of risk of bias for each included study. | SI Table S12 |
| Results of individual studies | 19 | For all outcomes, present, for each study: (a) summary statistics for each group (where appropriate) and (b) an effect estimates and its precision (e.g. confidence/credible interval), ideally using structured tables or plots. | Page 7-17 |
| Results of syntheses | 20a | For each synthesis, briefly summarise the characteristics and risk of bias among contributing studies. | NA |
|  | 20b | Present results of all statistical syntheses conducted. If meta-analysis was done, present for each the summary estimate and its precision (e.g., confidence/credible interval) and measures of statistical heterogeneity. If comparing groups, describe the direction of the effect. | Page 7-17 |
|  | 20c | Present results of all investigations of possible causes of heterogeneity among study results. | NA |
|  | 20d | Present results of all sensitivity analyses conducted to assess the robustness of the synthesized results. | NA |
| Reporting biases | 21 | Present assessments of risk of bias due to missing results (arising from reporting biases) for each synthesis assessed. | Page 18-19 |
| Certainty of evidence | 22 | Present assessments of certainty (or confidence) in the body of evidence for each outcome assessed. | NA |
| **DISCUSSION** | | |  |
| Discussion | 23a | Provide a general interpretation of the results in the context of other evidence. | Page 17-20 |
|  | 23b | Discuss any limitations of the evidence included in the review. | Page 17-20 |
|  | 23c | Discuss any limitations of the review processes used. | Page 17-20 |
|  | 23d | Discuss implications of the results for practice, policy, and future research. | Page 17-20 |
| **OTHER INFORMATION** | | |  |
| Registration and protocol | 24a | Provide registration information for the review, including register name and registration number, or state that the review was not registered. | Page 3 |
|  | 24b | Indicate where the review protocol can be accessed, or state that a protocol was not prepared. | Page 3 |
|  | 24c | Describe and explain any amendments to information provided at registration or in the protocol. | Page 3 |
| Support | 25 | Describe sources of financial or non-financial support for the review, and the role of the funders or sponsors in the review. | Page 1&20 |
| Competing interests | 26 | Declare any competing interests of review authors. | Page 20 |
| Availability of data, code and other materials | 27 | Report which of the following are publicly available and where they can be found: template data collection forms; data extracted from included studies; data used for all analyses; analytic code; any other materials used in the review. | NA |

# Appendix Figure S1: Publication years of included articles.


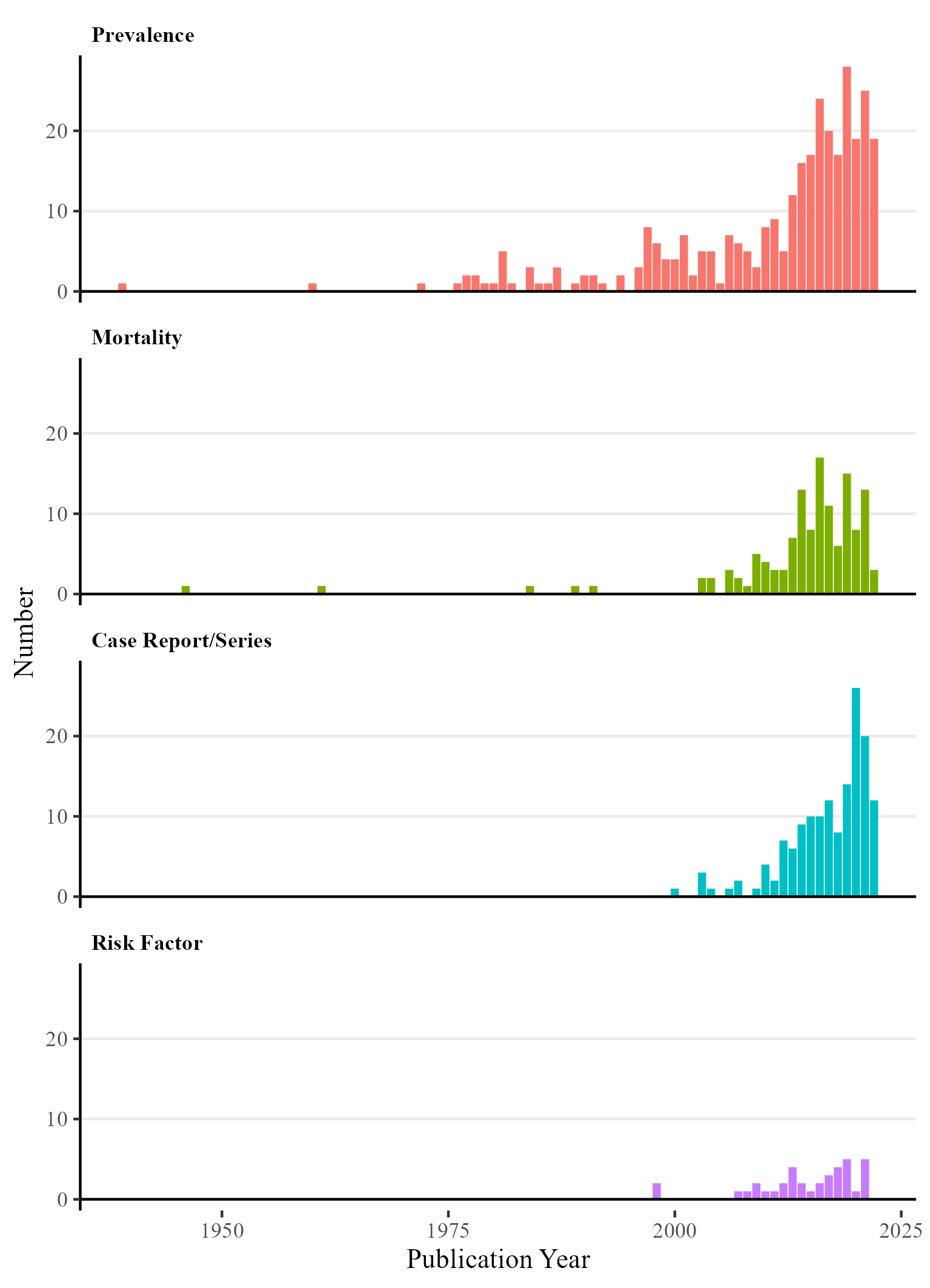


Figure S1 illustrates the number of included published studies on scrub typhus, categorized by research focus and displayed across a timeline from 1950 to the present. The first section, labeled 'Prevalence,' shows a histogram with the number of studies per year, represented by red bars, indicating an increase in publications over time, particularly in recent years. The 'Mortality' section, indicated by green bars, reveals a less consistent but present number of studies published across the decades. For 'Case Report/Series,' shown with cyan bars, there is a clear upward trend in the number of reports, especially in the most recent years, reflecting a growing interest or emergence of cases. 'Risk Factor' research, denoted by purple bars, displays a modest but increasing number of studies. Each section’s histogram bars correspond to the number of studies published in a given year, providing a visual representation of the research activity over time in each category related to scrub typhus.

# Appendix Figure S2: Location of included articles reported prevalence and case report and case series.


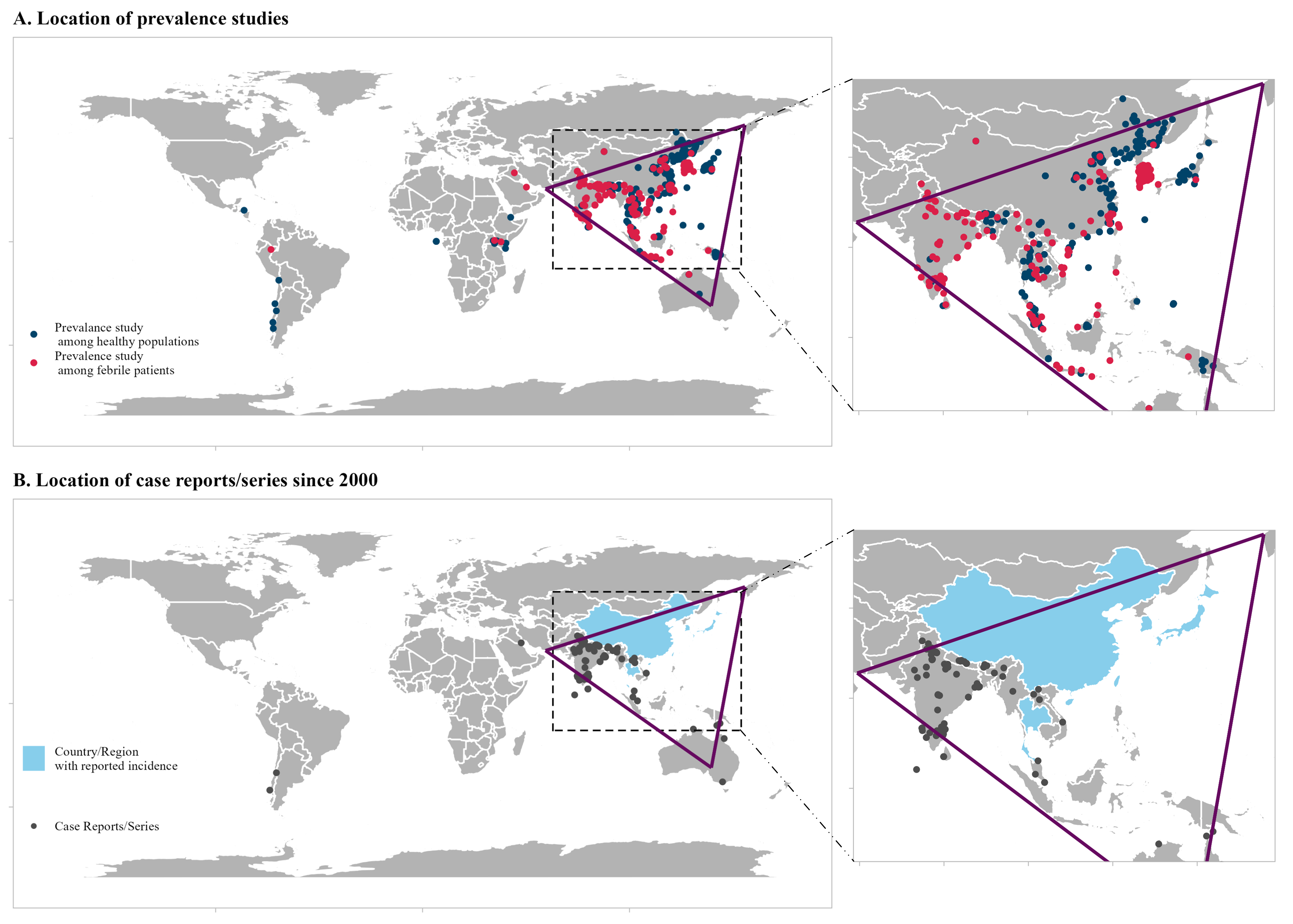


Figure S2 presents a global overview of the research on scrub typhus and the traditional tsutsugamushi triangle. Panel A illustrates the geographic distribution of prevalence studies conducted around the world, distinguished by two categories: studies among healthy populations and those among febrile patients, depicted with different colored dots. A focused inset highlights a region with a high concentration of studies. Panel B shows the locations of case reports and series related to scrub typhus since the year 2000. It includes a color-coded representation of countries or regions with reported incidence, along with the specific sites of case reports or series, represented by dots. An inset provides a detailed view of an area with notable research activity in this field.

# Appendix Figure S3: Reported age and sex-specific seroprevalence by included study. A. Gender specific seroprevalence among healthy populations; B. Gender specific seroprevalence among febrile patients; C. Age specific seroprevalence among healthy populations with age group midpoints used for bubble placement; D. Age specific seroprevalence among febrile patients with age group midpoints used for bubble placement.


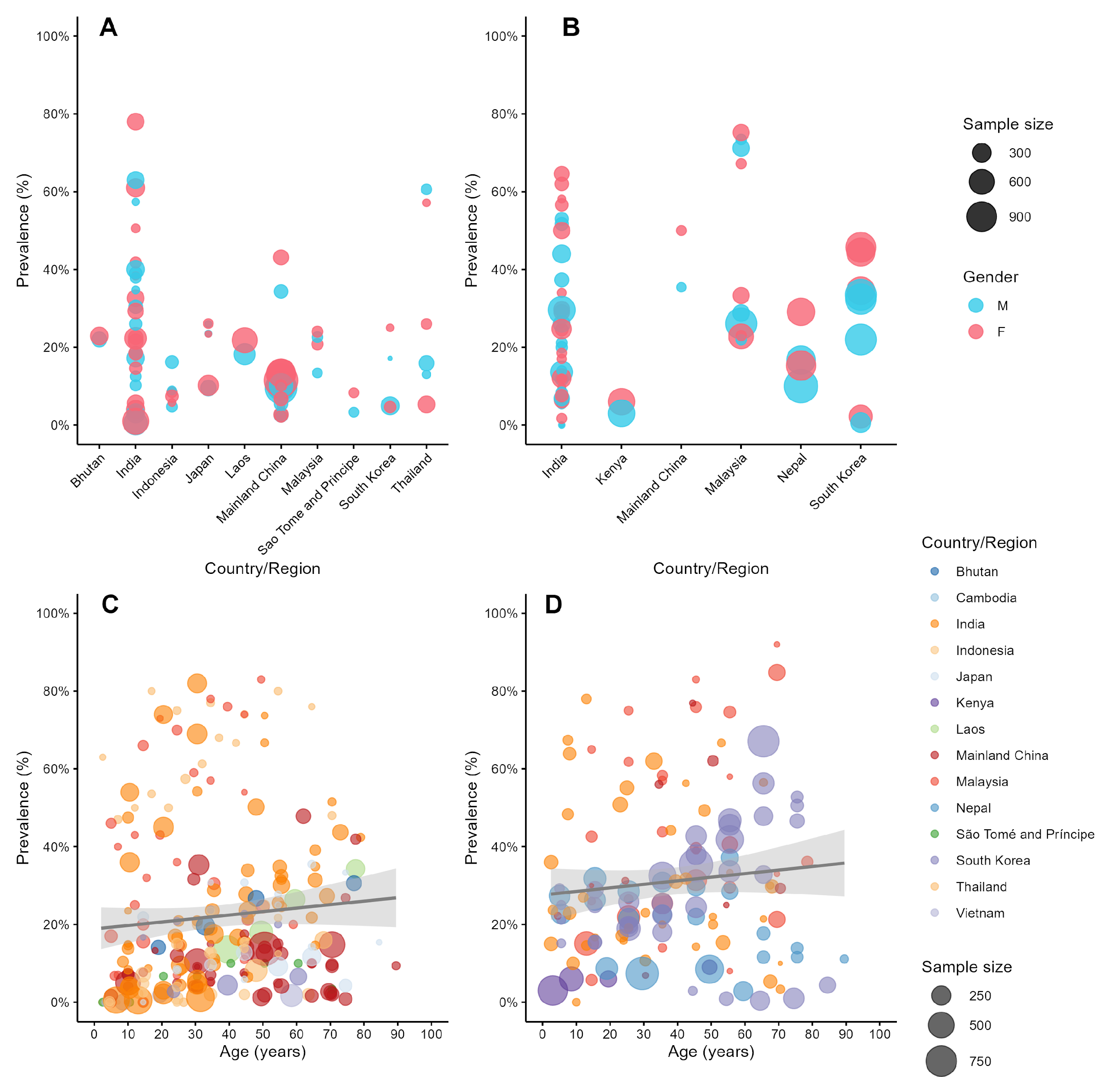


In a subset of 33 seroprevalence studies, which reported gender or age specific seroprevalence in 10 countries, the seroprevalence among healthy females was 17·04%, with a reported range of 0·98% to 78·00%, which was higher than in healthy males at 14·55% (range 0·49% to 63·00%) (Figure S3A, p<0.001). Detailed information is provided in the appendix (Table S4). The same trend was found in India, mainland China, and South Korea. However, Thailand stood as a notable exception where the seroprevalence among healthy males was higher (p<0.001) than that of healthy females (26·42%, range 12·40% to 60·58% vs 14·43%, range 5·30% to 57·14%). Among febrile populations, females generally exhibited higher seroprevalence (13·40%, range 0·98% to 78·00%)) than males (12·50% range 0·49% to 63·00%), p<0.001, Figure S3B). These significant gender disparities were also evident in India, Kenya, Nepal, and South Korea. For instance, in South Korea, out of 2 990 females, 1 055 tested positive (35·28%), whereas out of 3 301 males, 866 tested positive (26·23%, p<0.001).

Thirty-seven studies reported age-specific seroprevalence among healthy populations in 11 countries and another 21 studies provided age-specific seroprevalence among febrile patients in six countries (Figure S3). Generally, younger age groups had lower seroprevalence and as age increased, there were noticeable upward trends in seroprevalence both among healthy populations and febrile patients. This age-associated rise in seroprevalence was particularly pronounced in India and Thailand. India exhibited a continuous increase in seroprevalence with age in healthy populations, with a notable rise around the 60 years. Thailand's trend of seroprevalence among healthy people revealed a rise among the middle-aged, peaking around 40 years, and then slightly decreasing. In febrile patients, India and South Korea showed a steady increase in seroprevalence with age. In mainland China, among both healthy populations and febrile patients, there were significant peaks around 60 years and 50 years, respectively, followed by a decline and then another rise in the older age groups.

# Appendix Table S4: Gender-Specific Prevalence among healthy populations and febrile patients in countries/regions.

| **Country/region** | **Gender** | | **No.article** | **n/N** | **n/N (%)** | **Mean (%)** | **Median% (range)** | **Z** | **P value** |
| --- | --- | --- | --- | --- | --- | --- | --- | --- | --- |
| Gender specific prevalence among healthy populations | | | | | | | | | |
| Worldwide  all | F | | 33 | 3481/20433 | 17.04 | 19.95 | 13.40 (0.98~78.00) | 6.517 | 0.000* |
|  | M | | 34 | 2419/16630 | 14.55 | 18.21 | 12.50 (0.49~63.00) |  |  |
| Bhutan | F | | 1 | 119/519 | 22.93 | 22.90 | 22.90 (22.90~22.90) | 0.310 | 0.757 |
|  | M | | 1 | 76/345 | 22.03 | 22.00 | 22.00 (22.00~22.00) |  |  |
| India | F | | 8 | 1548/6430 | 24.07 | 29.44 | 22.54 (0.98~78.00) | 4.337 | 0.000* |
|  | M | | 8 | 1033/5001 | 20.66 | 26.23 | 26.00 (0.49~63.00) |  |  |
| Indonesia | F | | 3 | 31/416 | 7.45 | 7.20 | 7.40 (5.80~8.40) | -1.882 | 0.06 |
|  | M | | 3 | 52/466 | 11.16 | 9.93 | 8.90 (4.70~16.20) |  |  |
| Japan | F | | 2 | 114/881 | 12.94 | 19.90 | 23.40 (10.20~26.10) | 0.136 | 0.892 |
|  | M | | 2 | 61/481 | 12.68 | 19.67 | 23.50 (9.50~26.00) |  |  |
| Laos | F | | 1 | 251/1204 | 20.85 | 21.80 | 21.80 (21.80~21.80) | 1.613 | 0.107 |
|  | M | | 1 | 143/798 | 17.92 | 18.20 | 18.20 (18.20~18.20) |  |  |
| Mainland China | F | | 10 | 1215/9632 | 12.61 | 11.34 | 9.90 (2.19~43.10) | 4.941 | 0.000* |
|  | M | | 10 | 820/8017 | 10.23 | 9.88 | 9.92 (2.36~34.34) |  |  |
| Malaysia | F | | 2 | 72/322 | 22.36 | 22.40 | 22.40 (20.80~24.00) | 1.110 | 0.267 |
|  | M | | 2 | 48/258 | 18.60 | 18.00 | 18.00 (13.40~22.60) |  |  |
| Sao Tome and Principe | F | | 1 | 10/120 | 8.33 | 8.30 | 8.30 (8.30~8.30) | 1.652 | 0.098 |
|  | M | | 1 | 4/120 | 3.33 | 3.30 | 3.30 (3.30~3.30) |  |  |
| South Korea | F | | 2 | 24/237 | 10.13 | 14.81 | 14.81 (4.62~25.00) | 2.259 | 0.024* |
|  | M | | 2 | 33/580 | 5.69 | 11.04 | 11.04 (4.95~17.14) |  |  |
| Thailand | F | | 3 | 97/672 | 14.43 | 29.48 | 26.00 (5.30~57.14) | -5.256 | 0.000* |
|  | M | | 4 | 149/564 | 26.42 | 26.74 | 15.90 (12.40~60.58) |  |  |
| Gender specific prevalence among febrile patients | | | | | | | | | |
| Worldwide | | F | 24 | 2591/8892 | 29.14 | 29.68 | 24.60 (1.69~75.20) | 9.346 | 0.000* |
|  |  | M | 24 | 2434/10477 | 23.23 | 23.61 | 21.30 (0.00~73.40) |  |  |
| India | | F | 14 | 693/2388 | 29.02 | 26.84 | 21.51 (1.69~64.60) | 2.768 | 0.006* |
|  |  | M | 14 | 721/2817 | 25.59 | 20.31 | 13.80 (0.00~53.00) |  |  |
| Kenya | | F | 1 | 43/694 | 6.20 | 6.00 | 6.00 (6.00~6.00) | 2.460 | 0.014* |
|  |  | M | 1 | 24/707 | 3.39 | 3.00 | 3.00 (3.00~3.00) |  |  |
| Mainland China | | F | 2 | 28/56 | 50.00 | 36.82 | 36.82 (23.64~50.00) | 1.496 | 0.135 |
|  |  | M | 2 | 17/48 | 35.42 | 28.45 | 28.45 (21.48~35.42) |  |  |
| Malaysia | | F | 2 | 417/1129 | 36.94 | 44.30 | 33.30 (22.80~75.20) | 1.222 | 0.222 |
|  |  | M | 2 | 550/1587 | 34.66 | 44.28 | 28.80 (21.90~73.40) |  |  |
| Nepal | | F | 2 | 355/1635 | 21.71 | 22.20 | 22.20 (15.30~29.10) | 7.260 | 0.000* |
|  |  | M | 2 | 256/2017 | 12.69 | 13.35 | 13.35 (10.00~16.70) |  |  |
| South Korea | | F | 3 | 1055/2990 | 35.28 | 31.72 | 39.50 (2.20~45.70) | 7.783 | 0.000* |
|  |  | M | 3 | 866/3301 | 26.23 | 22.11 | 27.16 (0.60~33.50) |  |  |

This comprehensive table presents a detailed breakdown of the gender-specific prevalence of certain health conditions across various countries and regions worldwide. It includes data for both healthy populations and febrile patients. The table is organized by country or region and further segmented by gender, providing statistics such as article count, the number of cases (N) over the population (N), percentage prevalence, mean, median range, Z-statistic, and P-value. Key insights can be drawn from the varying prevalence rates between males and females in different countries, highlighting the importance of considering gender in health studies and policy making. The data showcases significant differences in health conditions between genders and across geographical locations, offering valuable information for researchers, public health officials, and policymakers.

# Appendix Figure S4. Diagnostic test method used in seroprevalence studies by country/region.


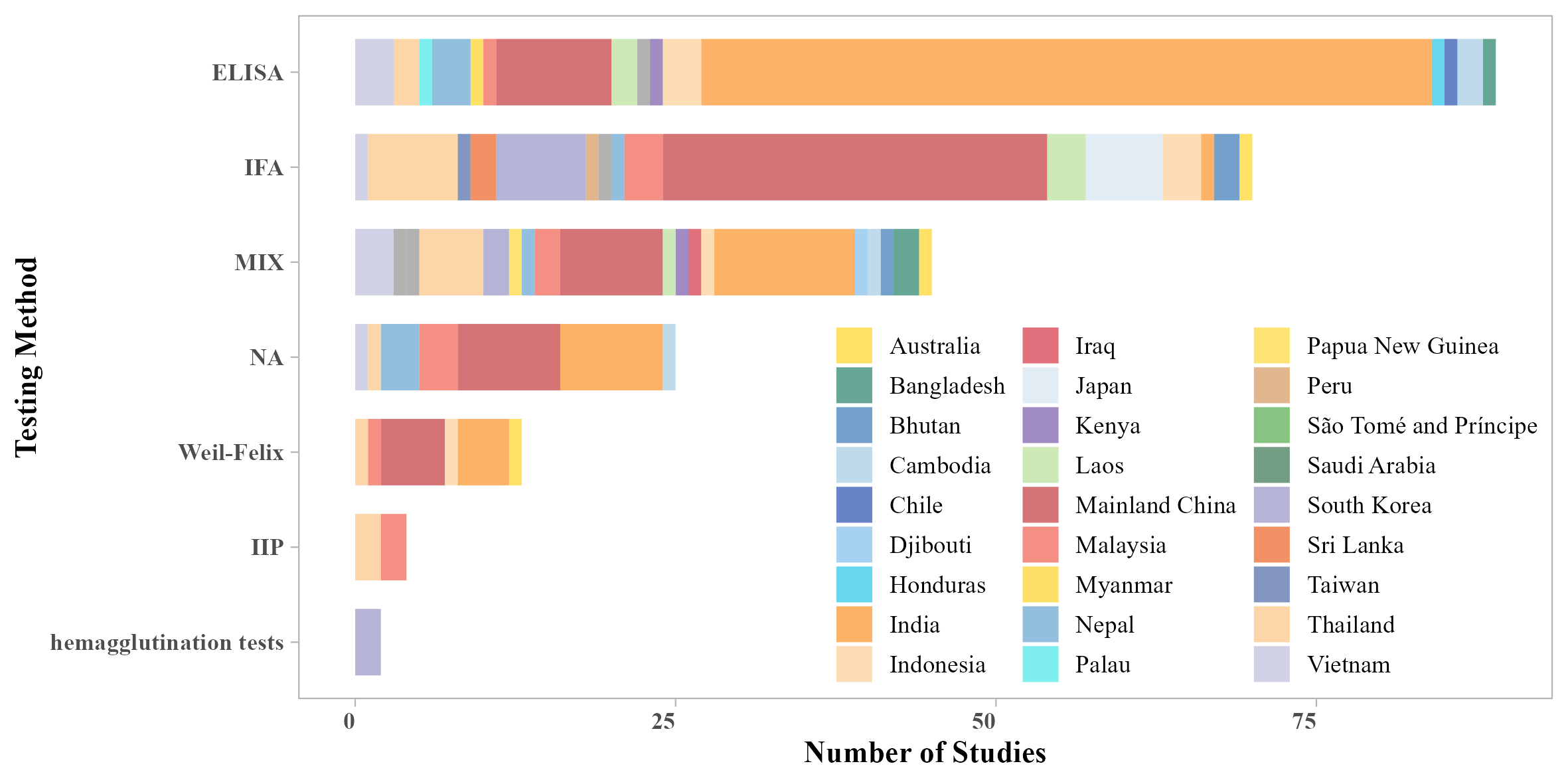


# Appendix Figure S5: Temporal changes in mortality.


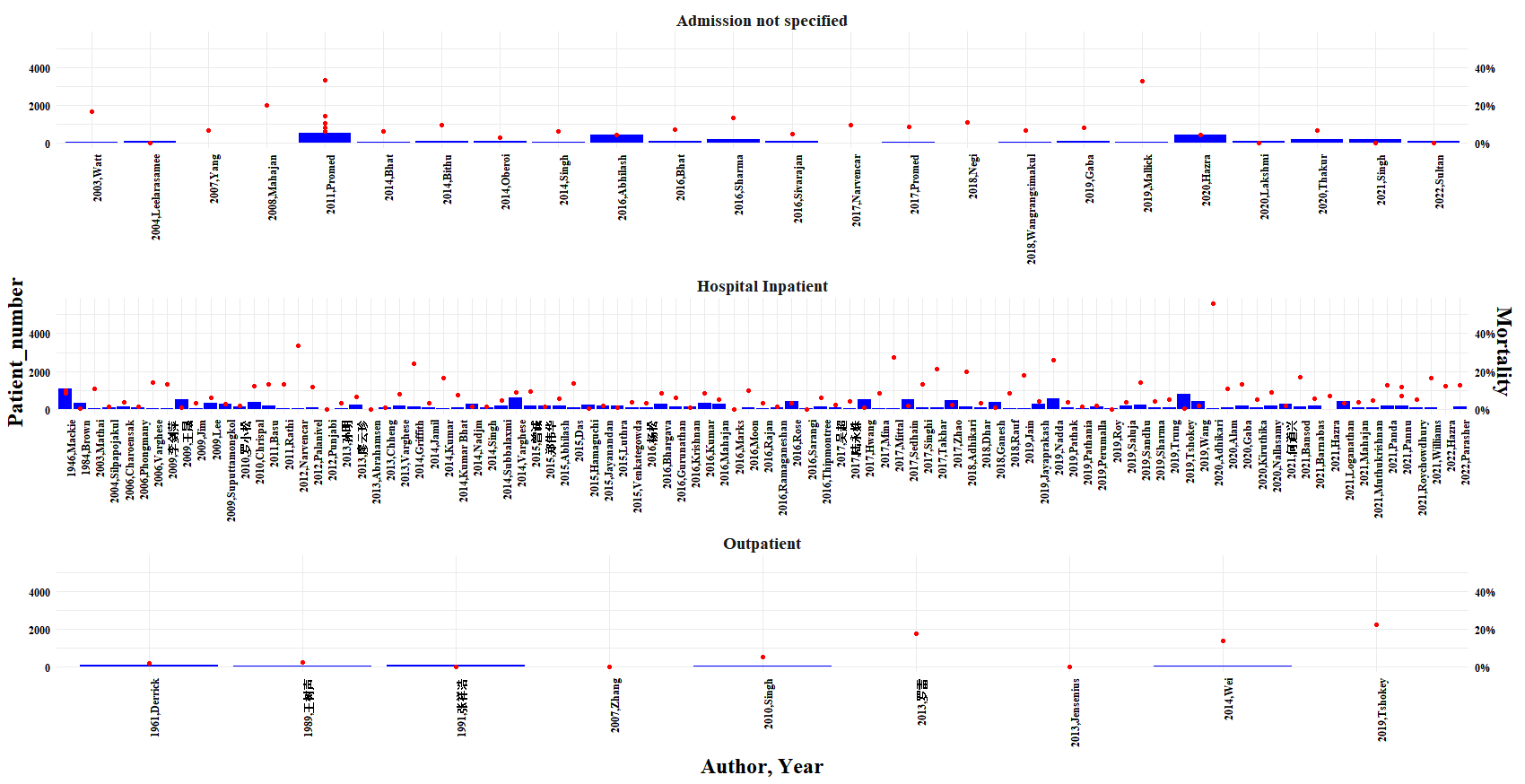


# Appendix Figure S6: Forest plot for reported exposures associated with scrub typhus in case-control or cross-sectional studies.


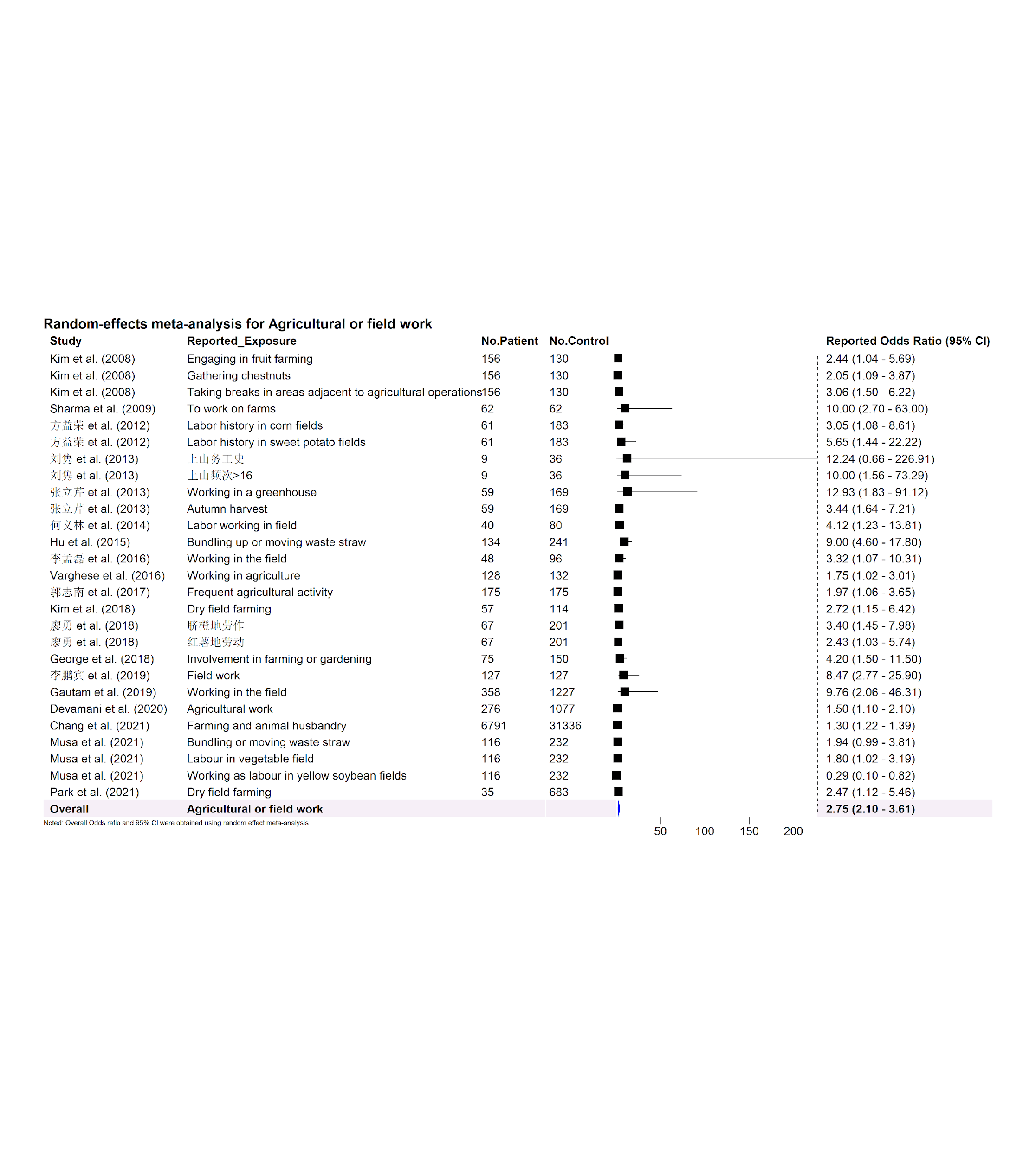




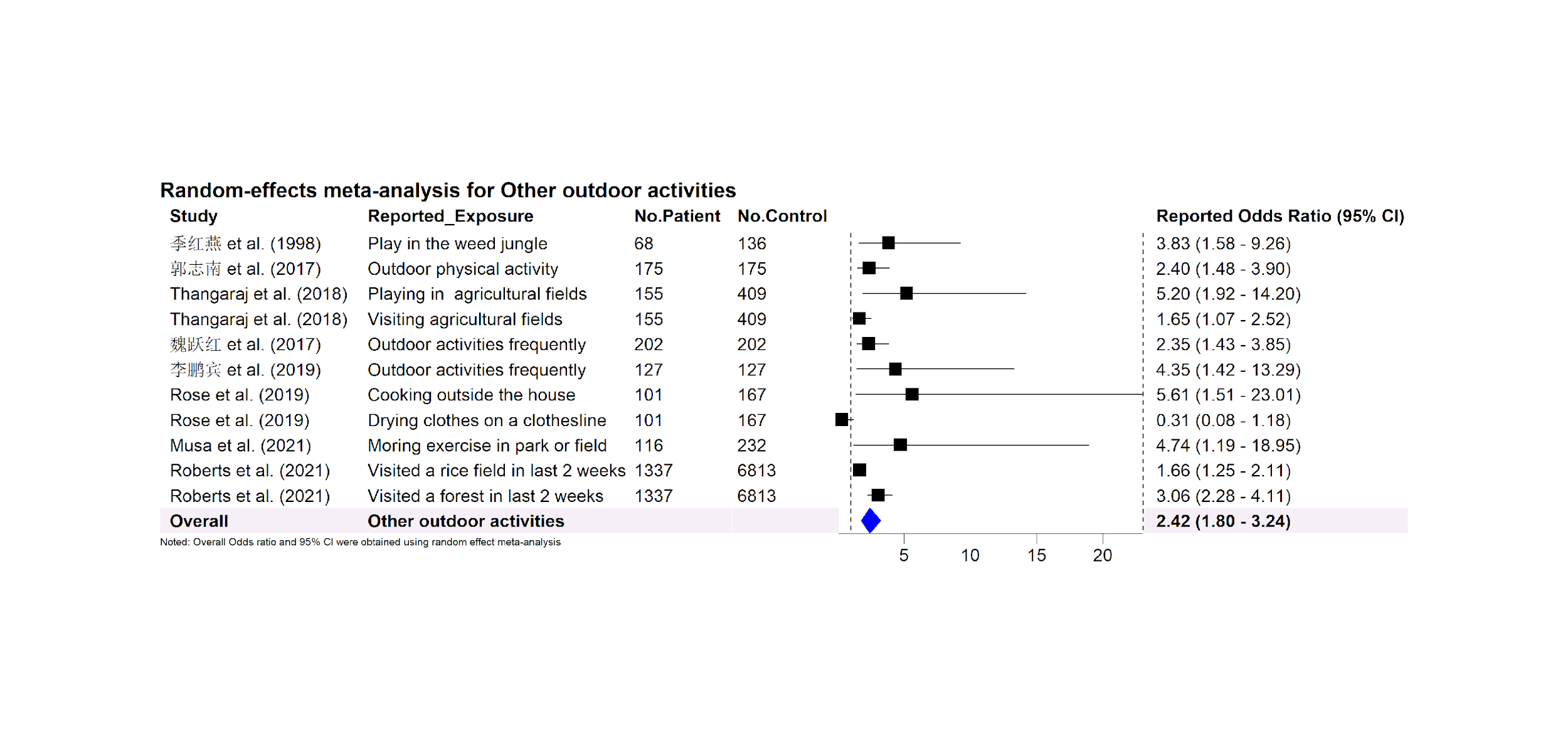


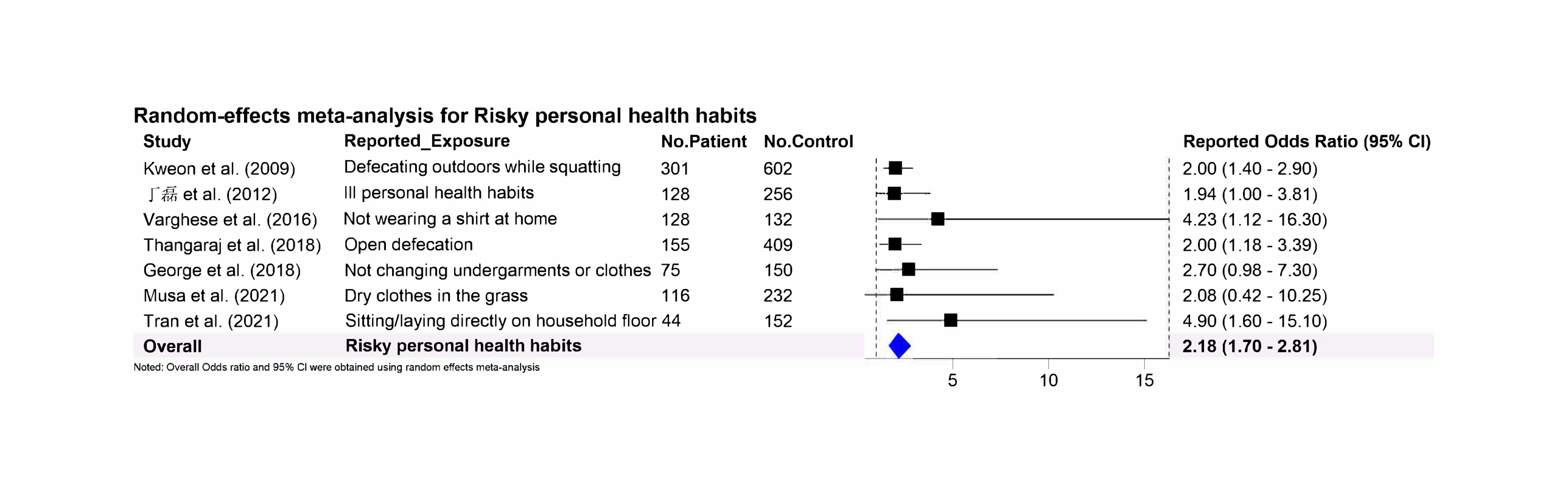


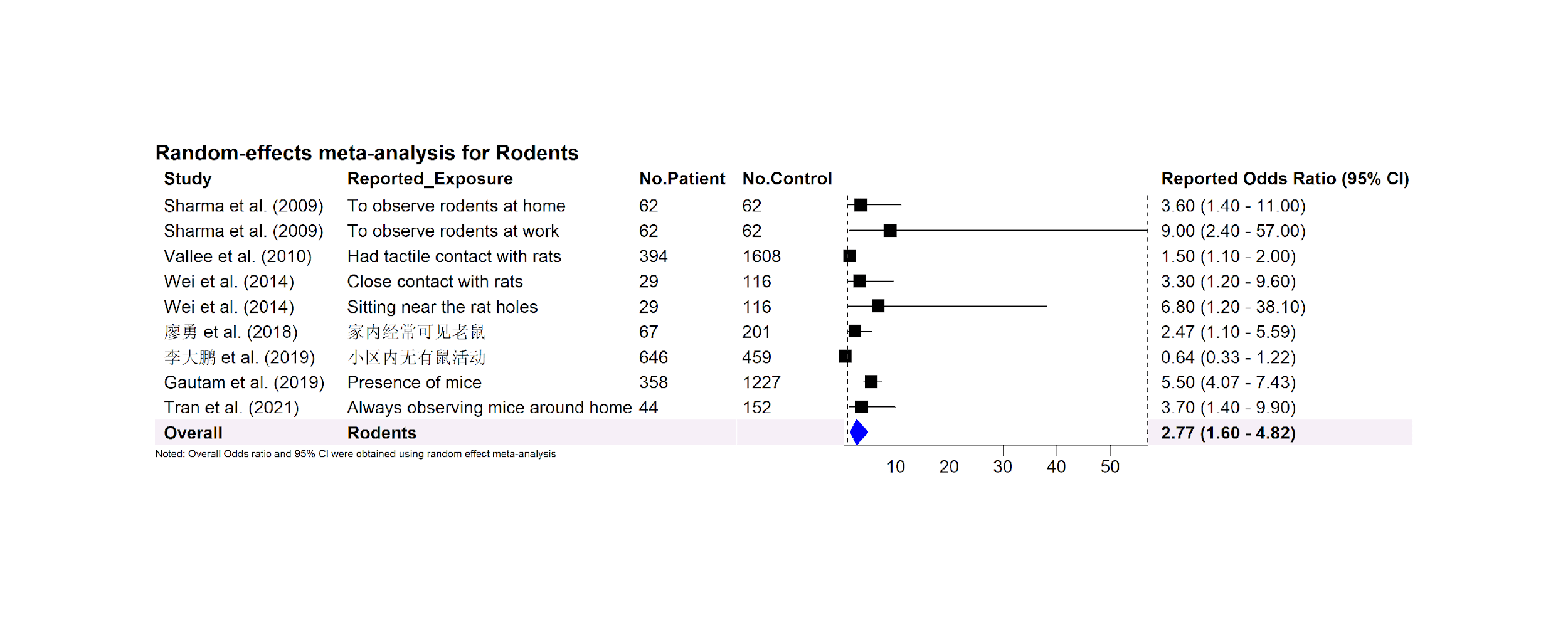


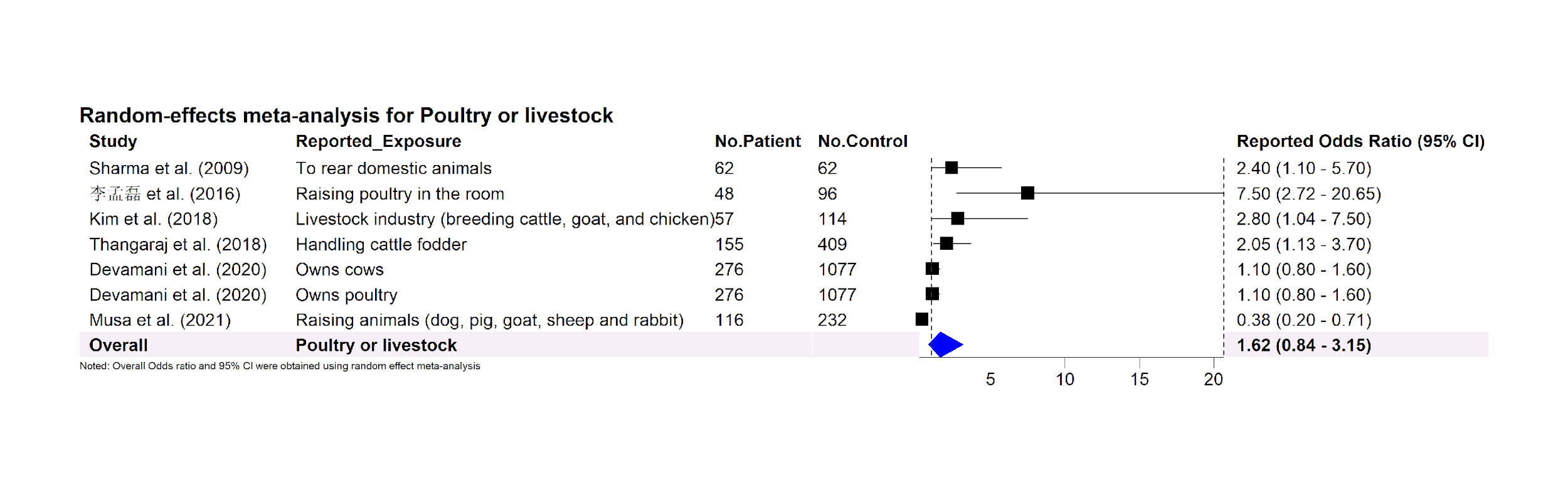

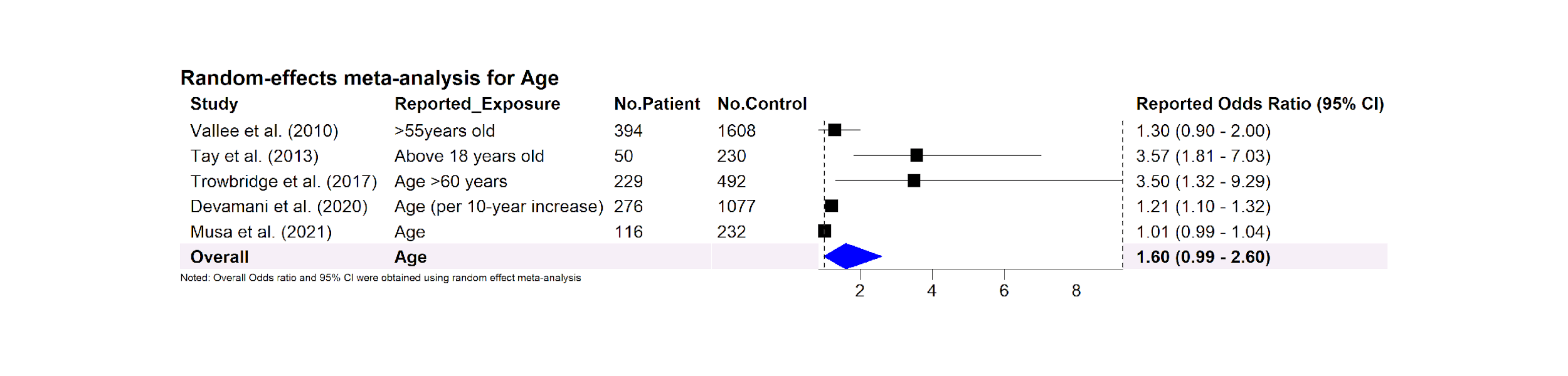


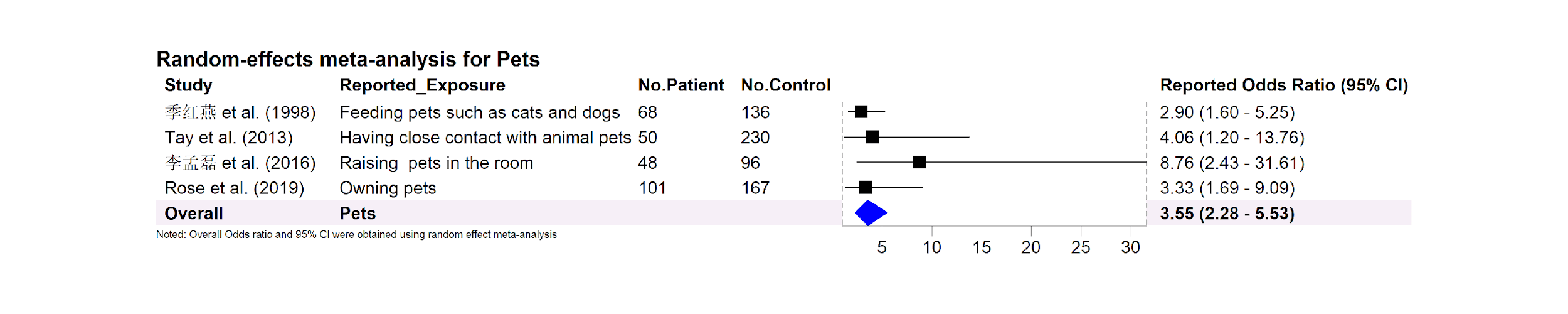

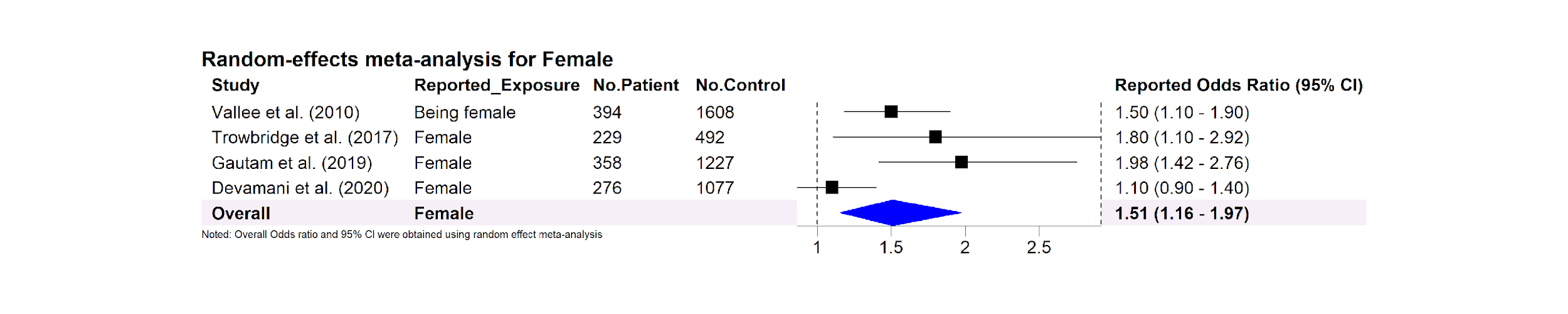


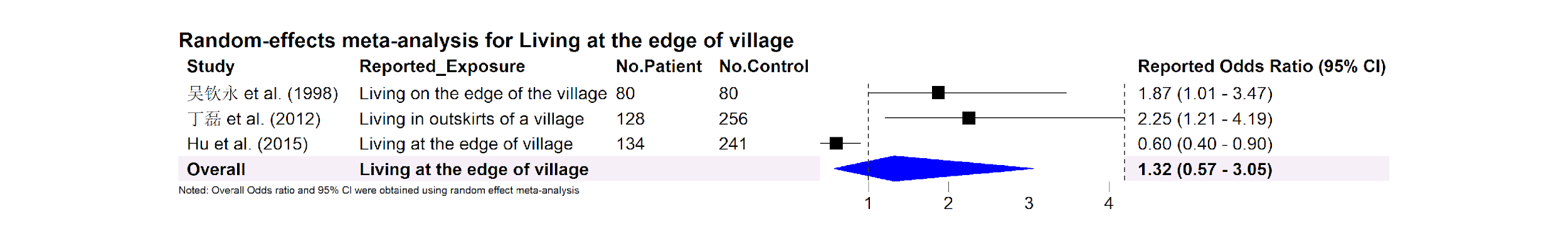


Appendix Figure S6 presents a forest plot summarizing the reported exposures associated with scrub typhus as identified in included studies. This visual representation systematically displays the effect sizes, such as odds ratios or risk ratios, along with their corresponding 95 confidence intervals for each identified exposure. The plot facilitates comparison across studies, illustrating the strength and direction of the association between various exposures and the risk of developing scrub typhus.

# Appendix Table S5: Characteristics of included seroprevalence studies, in order of country/region and published year (n=315).

| **ID** | **Author** | **Year of publication** | **Title** | **Language** | **Publication country** | **Study conducted country** | **Start-time of investigation** | **End-time of investigation** |
| --- | --- | --- | --- | --- | --- | --- | --- | --- |
| 1 | Graves, S. et.al.^1^ | 1999 | Rickettsia serosurvey in Kimberley, Western Australia | English | Australia | Australia | 1996 | 1996 |
| 2 | Harris PNA, et al. | 2016 | An seasonal of scrub typhus in military personnel despite protocols for antibiotic prophylaxis: doxycycline resistance excluded by a quantitative PCR-based susceptibility assay | English | Australia | Australia | 2011 | 2011 |
| 3 | Mathews, Karen O. et.al.^2^ | 2021 | Serological Evidence of Exposure to Spotted Fever Group and Typhus Group Rickettsiae in Australian Wildlife Rehabilitators | English | Australia | Australia | 2018 | 2018 |
| 4 | Arkell, P. et al. | 2022 | Integrated serological surveillance of acute febrile illness in the context of a lymphatic filariasis survey in Timor-Leste: a pilot study using dried blood spots. | English | Australia | Australia | 2018 | 2019 |
| 5 | Maude RR, et al. | 2016 | A prospective study of the importance of enteric fever as a cause of non-malarial febrile illness in patients admitted to Chittagong Medical College Hospital, Bangladesh | English | Bangladesh | Bangladesh | 2012 | 2012 |
| 6 | Faruque, Labib Imran. et.al.^3^ | 2017 | Prevalence and clinical presentation of Rickettsia, Coxiella, Leptospira, Bartonella and chikungunya virus infections among hospital-based febrile patients from December 2008 to November 2009 in Bangladesh | English | Bangladesh | Bangladesh | 2008.12 | 2009.11 |
| 7 | Kingston, Hugh W. et.al.^4^ | 2018 | Rickettsial Illnesses as Important Causes of Febrile Illness in Chittagong, Bangladesh | English | Australia | Bangladesh | 2014 | 2015 |
| 8 | Al Amin MM, et al. | 2019 | Molecular characterization of Orientia tsutsugamushi causing scrub typhus among febrile patients in north-central Bangladesh | English | Bangladesh | Bangladesh | 2018 | 2018 |
| 9 | Tshokey, Tshokey. et.al.^5^ | 2017 | Seroprevalence of rickettsial infections and Q fever in Bhutan | English | Australia | Bhutan | 2015 | 2015 |
| 10 | Tshokey, Tshokey. et.al.^6^ | 2018 | Rickettsial Infections and Q Fever Amongst Febrile Patients in Bhutan | English | Australia | Bhutan | 2014 | 2015 |
| 11 | Dorji, Kezang | 2019 | Clinical and Epidemiological Patterns of Scrub Typhus, an Emerging Disease in Bhutan | English | New Zealand | Bhutan | 2015.1 | 2015.12 |
| 12 | Tshokey, T. et.al.^7^ | 2019 | The epidemiology of rickettsial diseases, scrub typhus and q fever in bhutan: A first report | English | Bhutan | Bhutan | 2014 | 2014 |
| 13 | Chheng, Kheng. et.al.^8^ | 2013 | A prospective study of the causes of febrile illness requiring hospitalization in children in Cambodia | English | Cambodia | Cambodia | 2009.10.12 | 2010.10.12 |
| 14 | Mueller, Tara C. et.al.^9^ | 2014 | Acute undifferentiated febrile illness in rural Cambodia: a 3-year prospective observational study | English | Germany | Cambodia | 2008.01 | 2010.12 |
| 15 | Horwood PF, et al. | 2017 | Aetiology of acute meningoencephalitis in Cambodian children, 2010-2013. | English | Cambodia | Cambodia | 2010 | 2013 |
| 16 | Weitzel, Thomas. et.al. ^10^ | 2020 | Human seroepidemiology of Rickettsia and Orientia species in Chile - A cross-sectional study in five regions | English | Chile | Chile | 2010 | 2016 |
| 17 | Horton, K. C. et.al. ^11^ | 2016 | Evidence of rickettsia and orientia infections among abattoir workers in Djibouti | English | Egypt | Djibouti | 2010 | 2011 |
| 18 | Chao, Chien-Chung.et.al.^12^ | 2021 | Leptospirosis and Rickettsial Diseases Sero-Conversion Surveillance Among U.S. Military Personnel in Honduras | English | US | Honduras | 2000 | 2016 |
| 19 | Mathai, E. et al. | 2001 | Serological evidence for the continued presence of human rickettsioses in southern India | English | India | India |  |  |
| 20 | Isaac, Rita.et.al.^13^ | 2004 | Scrub typhus: prevalence and diagnostic issues in rural Southern India | English | India | India | 2002 | 2003 |
| 21 | Varghese, G. M. et.al. ^14^ | 2006 | Scrub typhus among hospitalised patients with febrile illness in South India: magnitude and clinical predictors | English | India | India | 2002 | 2003 |
| 22 | Kamarasu K, et al. | 2007 | Serological evidence for wide distribution of spotted fevers & typhus fever in Tamil Nadu. | English | India | India | 2004 | 2005 |
| 23 | Chrispal, Anugrah.et.al.^15^ | 2010 | Scrub typhus: an unrecognized threat in South India - clinical profile and predictors of mortality | English | India | India | 2007 | 2008 |
| 24 | Prabhakaran, A. et.al | 2010 | Serological study of rickettsial diseases in human and rodent population in Chittoor dist. (A.P.) | English | India | India | 2008.01 | 2008.02 |
| 25 | Basu, Gopal. et.al. ^16^ | 2011 | Acute kidney injury in tropical acute febrile illness in a tertiary care centre--RIFLE criteria validation | English | India | India | 2007.01 | 2008.01 |
| 26 | Rathi, Narendra B. et.al.^17^ | 2011 | Rickettsial diseases in central India: proposed clinical scoring system for early detection of spotted fever | English | India | India | 2009 | 2009 |
| 27 | Mittal, Veena.et.al.^18^ | 2012 | Serological evidence of rickettsial infections in Delhi | English | India | India | 1999 | 2009 |
| 28 | Narvencar, Kedareshwar P. S. et.al.^19^ | 2012 | Scrub typhus in patients reporting with acute febrile illness at a tertiary health care institution in Goa | English | India | India | 2009 | 2010 |
| 29 | Abrahamsen, Siri Kratter.et.al.^20^ | 2013 | Fever in the tropics: aetiology and case-fatality - a prospective observational study in a tertiary care hospital in South India | English | Norway | India | 2007.07.02 | 2007.08.02 |
| 30 | S Gurung, et.al. | 2013 | Outbreak of scrub typhus in the North East Himalayan region-Sikkim: an emerging threat | English | India | India | 2011 | 2011 |
| 31 | Bithu, R. et.al.^21^ | 2014 | Possibility of scrub typhus in fever of unknown origin (FUO) cases: an experience from Rajasthan | English | India | India | 2012 | 2012 |
| 32 | Griffith, Mathew. et.al.^22^ | 2014 | Profile of organ dysfunction and predictors of mortality in severe scrub typhus infection requiring intensive care admission | English | India | India | 2008 | 2010 |
| 33 | Kumar, Vive, et.al. | 2014 | Scrub typhus is an under-recognized cause of acute febrile illness with acute kidney injury in India | English | India | India | 2011 | 2012 |
| 34 | Oberoi, Aroma.et.al.^23^ | 2014 | Scrub typhus-an emerging entity: a study from a tertiary care hospital in North India | English | India | India | 2011 | 2012 |
| 35 | Singh, Ragini.et.al.^24^ | 2014 | A Study of Etiological Pattern in an Epidemic of Acute Febrile Illness during Monsoon in a Tertiary Health Care Institute of Uttarakhand, India | English | Inda | India | 2013.07 | 2013.11 |
| 36 | Usha, K. et.al.^25^ | 2014 | Seroprevalence of scrub typhus among febrile patients: A preliminary study | English | India | India | 2011 | 2012 |
| 37 | Sankhyan N, et.al. | 2014 | Clinical profile of scrub typhus in children and its association with hemophagocytic lymphohistiocytosis | English | India | India | 2011 | 2011 |
| 38 | Sinha P, et.al. | 2014 | Recent outbreak of scrub typhus in North Western part of India | English | India | India | 2012 | 2012 |
| 39 | Das, Dipmala. et.al. ^26^ | 2015 | Common Infectious Etiologies of Acute Febrile Illness in a Remote Geographical Location: Could Scrub Typhus be the Most Common Cause? | English | USA | India | 2013.05 | 2013.10 |
| 40 | Koraluru, Munegowda, et.al. | 2015 | Diagnostic validation of selected serological tests for detecting scrub typhus | English | India | India | 2012 | 2013 |
| 41 | Rashmi, K. S. et.al. ^27^ | 2015 | Rickettsial Diseases: A Study Evidenced by Weil-Felix Test in a Tertiary Care Hospital | English | India | India | 2014 | 2015 |
| 42 | Roopa, K. S. et.al. ^28^ | 2015 | Serodiagnosis of Scrub Typhus at a Tertiary Care Hospital from Southern India | English | India | India | 2012 | 2015 |
| 43 | Sengupta, M. et.al.^29^ | 2015 | Scrub typhus seroprevalence in healthy Indian population | English | India | India | 2012 | 2012 |
| 44 | Stephen, Selvaraj. et.al. ^30^ | 2015 | Outbreak of scrub typhus in Puducherry & Tamil Nadu during cooler months | English | India | India | 2012 | 2013 |
| 45 | Nawab T, et.al. | 2015 | A clinical study of rickettsial disease and its manifestations. | English | India | India | 2013 | 2014 |
| 46 | Usha K, et.al. | 2015 | Molecular detection of scrub typhus in Tirupati, Andhra pradesh, India. | English | India | India | 2013 | 2014 |
| 47 | Abhilash, Kundavaram Paul Prabhakar. et.al.^31^ | 2016 | Acute Undifferentiated Febrile Illness in Patients Presenting to a Tertiary Care Hospital in South India: Clinical Spectrum and Outcome | English | India | India | 2012.10 | 2013.09 |
| 48 | Sivarajan, Sunuraj, et.al | 2016 | Clinical and paraclinical profile, and predictors of outcome in 90 cases of scrub typhus, Meghalaya, India | English | India | India | 2011.09 | 2012.08 |
| 49 | Thomas, Rwituja, et.al. | 2016 | Five-year analysis of rickettsial fevers in children in South India: Clinical manifestations and complications | English | India | India | 2008.01 | 2012.12 |
| 50 | Usha, K.,et.al. | 2016 | Molecular characterization of Orientia tsutsugamushi serotypes causing scrub typhus outbreak in southern region of Andhra Pradesh, India | English | India | India | 2011 | 2013 |
| 51 | Bhargava, Anurag. et.al. ^32^ | 2016 | Scrub typhus in Uttarakhand & adjoining Uttar Pradesh: Seasonality, clinical presentations & predictors of mortality | English | India | India | 2012 | 2013 |
| 52 | Farhana, Anjum. et.al. ^33^ | 2016 | Serological Evidence of Scrub Typhus among Cases of PUO in the Kashmir Valley- A Hospital Based Study | English | India | India | 2015 | 2015 |
| 53 | Jakharia, Aniruddha. et.al. ^34^ | 2016 | Seroprevalence of Scrub Typhus Infection in Arunachal Pradesh, India | English | India | India | 2009 | 2013 |
| 54 | Kalal, B. S. et.al.^35^ | 2016 | Scrub typhus and spotted fever among hospitalised children in South India: Clinical profile and serological epidemiology | English | India | India | 2010 | 2012 |
| 55 | Khan, S. A. et.al. ^36^ | 2016 | Scrub typhus in hilly regions of north east India | English | India | India | 2013 | 2014 |
| 56 | Rajan, S. J. et.al. ^37^ | 2016 | Scrub typhus in pregnancy: Maternal and fetal outcomes | English | India | India | 2010 | 2012 |
| 57 | Sarangi, R. et.al. ^38^ | 2016 | Clinical profile of scrub typhus in children treated in a tertiary care hospital in eastern India | English | India | India | 2015 | 2015 |
| 58 | Ahmad S, et al. | 2016 | A comparative hospital-based observational study of mono- and co-infections of malaria, dengue virus and scrub typhus causing acute undifferentiated fever. | English | India | India | 2012 | 2013 |
| 59 | Khan SA, et.al. | 2016 | Seroepidemiology of rickettsial infections in Northeast India | English | India | India | 2013 | 2015 |
| 60 | Arun Babu, Thirunavukkarasu, et.al. ^39^ | 2017 | Characteristics of Pediatric Scrub Typhus Eschar in South Indian Children | English | India | India | 2013 | 2015 |
| 61 | Manjunath, V. G. et.al. ^40^ | 2017 | Clinical features, laboratory findings and complications of scrub typhus in South Indian children | English | India | India | 2011.12 | 2012.11 |
| 62 | Mina, S. S., et.al. ^41^ | 2017 | Emerging Infections in Children in North India: Scrub Typhus | English | India | India | nan | nan |
| 63 | Mittal, Mahima. et.al. ^42^ | 2017 | Scrub Typhus as a Cause of Acute Encephalitis Syndrome, Gorakhpur, Uttar Pradesh, India | English | India | India | 2016 | 2016 |
| 64 | Morch, Kristine. et.al. ^43^ | 2017 | Acute undifferentiated fever in India: a multicentre study of aetiology and diagnostic accuracy | English | Norway | India | 2011.04 | 2012.11 |
| 65 | Narvencar, Kedareshwar. et.al. ^44^ | 2017 | Rickettsial Infections in Goa-Not Just Scrub Typhus! | English | India | India | nan | nan |
| 66 | Shelke, Yogendra Pandurang.et.al.^45^ | 2017 | Spectrum of infections in acute febrile illness in central India | English | India | India | 2015 | 2016 |
| 67 | Singhi, Sunit. et.al. ^46^ | 2017 | Tropical Fevers in Indian Intensive Care Units: A Prospective Multicenter Study | English | India | India | 2013 | 2014 |
| 68 | Trowbridge, Paul. et.al. ^47^ | 2017 | Prevalence and risk factors for scrub typhus in South India | English | USA | India | nan | nan |
| 69 | Vivian Thangaraj, et.al. ^48^ | 2017 | Scrub Typhus as an Etiology of Acute Febrile Illness in Gorakhpur, Uttar Pradesh, India, 2016 | English | India | India | 2016 | 2016 |
| 70 | Jain P, et al. | 2017 | Aetiology of acute encephalitis syndrome in Uttar Pradesh, India from 2014 to 2016. | English | India | India | 2014 | 2016 |
| 71 | Srinivasan S, Menon T. | 2017 | Molecular detection of Orientia tsutsugamushi from suspected scrub typhus cases | English | India | India |  |  |
| 72 | Arvind, N. et.al. ^49^ | 2018 | Clinical and microbiological profile of patients with acute febrile illness attending a tertiary care hospital in South India | English | India | India | 2016.01 | 2016.12 |
| 73 | Giri, Prabahs Prasun, et.al.^50^ | 2018 | Scrub Typhus - A Major Cause of Pediatric Intensive Care Admission and Multiple Organ Dysfunction Syndrome: A Single-Center Experience from India | English | India | India | 2012 | 2015 |
| 74 | Jacob, S. M. et.al. ^51^ | 2018 | Seroprevalence and clinical manifestations of scrub typhus infection in Chennai city: A cross-sectional study | English | India | India | 2015 | 2016 |
| 75 | Jain, Parul. et.al. ^52^ | 2018 | Emergence of Orientia tsutsugamushi as an important cause of Acute Encephalitis Syndrome in India | English | India | India | 2015.06 | 2016.09 |
| 76 | Raina, Sujeet. et.al. ^53^ | 2018 | Coinfections as an aetiology of acute undifferentiated febrile illness among adult patients in the sub-Himalayan region of north India | English | India | India | 2016.09 | 2016.10 |
| 77 | Rauf, Abdul. et.al.^54^ | 2018 | Non-Respiratory and Non-Diarrheal Causes of Acute Febrile Illnesses in Children Requiring Hospitalization in a Tertiary Care Hospital in North India: A Prospective Study | English | India | India | 2014 | 2014 |
| 78 | Rizvi, Meher. et.al. ^55^ | 2018 | Prevalence of scrub typhus in pyrexia of unknown origin and assessment of interleukin-8, tumor necrosis factor-alpha, and interferon-gamma levels in scrub typhus-positive patients | English | India | India | 2014 | 2014 |
| 79 | Koralur, Munegowda, et.al. | 2018 | Scrub typhus diagnosis on acute specimens using serological and molecular assays - a 3-year prospective study | English | India | India | 2012 | 2015 |
| 80 | Stephen, Selvaraj. et.al. ^56^ | 2018 | Serological evidence of spotted fever group rickettsiosis in and around Puducherry, south India-A three years study | English | India | India | 2012 | 2015 |
| 81 | Mittal M, et.al. | 2018 | Acute Encephalitis Syndrome in Gorakhpur, Uttar Pradesh, 2016: Clinical and Laboratory Findings. | English | India | India | 2016 | 2016 |
| 82 | Behera, Bijayini.et.al. ^57^ | 2019 | Clinico-epidemiological analysis of scrub typhus in hospitalised patients presenting with acute undifferentiated febrile illness: A hospital-based study from Eastern India | English | India | India | 2017.01 | 2017.12 |
| 83 | Boda, Subbarayudu. and Goutham, V.V.N. ^58^ | 2019 | Clinical Spectrum of Acute Undifferentiated Fever - An Experience from a Tertiary Care Centre | English | India | India | 2018.06 | 2019.06 |
| 84 | Devamani, Carol S. et.al. ^59^ | 2019 | Hospitalisations and outpatient visits for undifferentiated fever attributable to scrub typhus in rural South India: Retrospective cohort and nested case-control study | English | India | India | 2018 | 2018 |
| 85 | Jain, Deepak. et.al. ^60^ | 2019 | Scrub typhus infection, not a benign disease: an experience from a tertiary care center in Northern India | English | India | India | 2017 | 2017 |
| 86 | Jha, Ruchi and Anil Kumar Jaiswal ^61^ | 2019 | Clinico-laboratory profile of central nervous system infection by scrub typhus at a tertiary care hospital | English | India | India | 2016 | 2018 |
| 87 | Mallick, Sanjay Kumar. et.al.^62^ | 2019 | Scrub typhus: a hospital-based study in the northern districts of West Bengal, India | English | India | India | 2016 | 2018 |
| 88 | Patil, R. and Kulkarni, S.^63^ | 2019 | Evaluation of Acute Febrile Illness in Patients Presenting to a Tertiary Care Hospital | English | India | India | 2018.08 | 2019.02 |
| 89 | Perumalla, Susmitha Karunasree, et.al. ^64^ | 2019 | Eschar and IgM ELISA in the diagnosis of scrub typhus | English | India | India | 2015 | 2017 |
| 90 | Rao, Pavitra N. et.al. ^65^ | 2019 | Dengue, chikungunya, and scrub typhus are important etiologies of non-malarial febrile illness in Rourkela, Odisha, India | English | USA | India | 2016.01 | 2017.01 |
| 91 | Shankar, V., et.al. ^66^ | 2019 | Scrub Typhus in a Tertiary Care Hospital in Visakhapatnam | English | India | India | 2017 | 2018 |
| 92 | Bal M, et al. | 2019 | Profile of Pediatric Scrub Typhus in Odisha, India. | English | India | India | 2017 | 2017 |
| 93 | Basu S, et al. | 2019 | Clinical profile and therapeutic response of scrub typhus in children: A recent trend from Eastern India | English | India | India | 2017 | 2017 |
| 94 | Alam, Areesha. et.al. ^67^ | 2020 | Prediction Rule for Scrub Typhus Meningoencephalitis in Children: Emerging Disease in North India | English | India | India | 2016 | 2018 |
| 95 | Devamani, Carol S. et.al. ^68^ | 2020 | Risk Factors for Scrub Typhus, Murine Typhus, and Spotted Fever Seropositivity in Urban Areas, Rural Plains, and Peri-Forest Hill Villages in South India: A Cross-Sectional Study | English | India | India | nan | nan |
| 96 | Hazra, D. et.al. ^69^ | 2020 | Scrub typhus: Clinical presentation and severity | English | India | India | 2012 | 2013 |
| 97 | Kamble, Suchit. et.al. ^70^ | 2020 | Seroprevalence & seroincidence of Orientia tsutsugamushi infection in Gorakhpur, Uttar Pradesh, India: A community-based serosurvey during lean (April-May) & epidemic (October-November) periods for acute encephalitis syndrome | English | India | India | 2016 | 2016 |
| 98 | Lakshmi, Ram Mohan Mylavarapu Venkata Naga, et.al. ^71^ | 2020 | Prevalence of scrub typhus in a tertiary care centre in Telangana, south India | English | India | India | 2018 | 2018 |
| 99 | Vikram, Kumar. et.al. ^72^ | 2020 | Scrub typhus and leptospirosis in rural and urban settings of central India: a preliminary evaluation | English | India | India | 2018 | 2018 |
| 100 | Thakur, C. K. et.al. | 2020 | Scrub typhus in patients with acute febrile illness: a 5-year study from India | English | India | India | 2013 | 2018 |
| 101 | Yaqoob, S. et.al. ^73^ | 2020 | Scrub typhus: A Neglected tropical Disease and A Potential Threat in North India | English | India | India | 2017 | 2017 |
| 102 | Tiwari, S.Nanda, M. | 2020 | Seroprevalence of scrub typhus among Pyrexia of Unknown Origin patients: A study from tertiary care hospital in Eastern Odisha, India | English | India | India | 2019 | 2019 |
| 103 | Anitharaj V, | 2020 | Scrub typhus in Puducherry, India: Application of nested PCR targeting three different genes - 56 kDa, 47 kDa and groEL of Orientia tsutsugamushi and comparison with ST IgM ELISA | English | India | India | 2012 | 2017 |
| 104 | Saravanan N, et al. | 2020 | Detection of scrub typhus by real-time polymerase chain reaction and immunoglobulin M ELISA among patients with acute febrile illness | English | India | India | 2017 | 2018 |
| 105 | Anupriya, A., et.al. ^74^ | 2021 | Scrub typhus infection among puo cases in the pediatric population in a tertiary care hospital | English | India | India | 2019 | 2020 |
| 106 | Bal, Madhusmita, et.al. ^75^ | 2021 | Scrub typhus associated acute kidney injury: An emerging health problem in Odisha, India | English | India | India | 2018 | 2019 |
| 107 | Behera, Sthita Pragnya, et.al. ^76^ | 2021 | Molecular Detection and Genetic Characterization of Orientia tsutsugamushi from Hospitalized Acute Encephalitis Syndrome Cases During Two Consecutive Outbreaks in Eastern Uttar Pradesh, India | English | India | India | 2016 | 2017 |
| 108 | Chaudhari SP, et al. | 2021 | Journey towards national institute of one health in India | English | India | India | 2015 | 2019 |
| 109 | Kavirayani, V. et.al. ^77^ | 2021 | Clinical profile and role of serology in pediatric acute febrile illness: Experience from a tertiary care hospital in South India | English | India | India | 2018 | 2018 |
| 110 | Khan, Siraj A. et.al. ^78^ | 2021 | Seroprevalence of Rickettsial Infections in Northeast India: A Population-Based Cross-Sectional Survey | English | India | India | 2017 | 2018 |
| 111 | Mahajan, Vidushi, et.al. ^79^ | 2021 | Spectrum of Multiorgan Dysfunction in Scrub Typhus Infection | English | India | India | 2013 | 2017 |
| 112 | Matlani, M. et.al. ^80^ | 2021 | Etiological trends and epidemiological profile of tropical fever in children presenting with acute undifferentiated fever at a tertiary care centre in north India | English | India | India | 2019.06 | 2019.12 |
| 113 | Mansoor, Tabeen, et.al. | 2021 | Rickettsial Infections among the Undifferentiated Febrile Patients Attending a Tertiary Care Teaching Hospital of Northern India: A Longitudinal Study | English | India | India | 2016 | 2017 |
| 114 | Mittal, Vineeta S, et.al. ^81^ | 2021 | Scrub typhus: An under-reported and emerging threat - hospital based study from central and eastern Uttar Pradesh, India | English | India | India | 2018 | 2019 |
| 115 | Panda, A., et.al. ^82^ | 2021 | Clinico-epidemiological and outcome of scrub typhus in paediatric patients: An observational study from Odisha, India | English | India | India | 2017 | 2019 |
| 116 | Paulraj, Philip Samuel, et.al. ^83^ | 2021 | First seroprevalence report of scrub typhus from the tribal belts of the Nilgiris district, Tamil Nadu, India | English | India | India | 2014.10 | 2016.03 |
| 117 | Singh, Sweta, et.al. ^84^ | 2021 | Seroprevalence trends of Scrub typhus among the febrile patients of Northern India: A prospective cross-sectional study | English | India | India | 2017 | 2020 |
| 118 | Somasunder, V. M. et.al. ^85^ | 2021 | Serological detection and epidemiological factors associated with scrub typhus among undifferentiated febrile illness patients in a tertiary care hospital | English | India | India | 2019 | 2020 |
| 119 | Raychaudhuri D, et al. | 2021 | COVID-19 and Co-infection in children: The Indian perspectives | English | India | India | 2020 | 2020 |
| 120 | Husain, Uneza, et.al. ^86^ | 2022 | Scrub Typhus and its co-infection with Leptospirosis at a tertiary care hospital in Uttar Pradesh | English | India | India | 2019 | 2019 |
| 121 | Sultan, A., et.al. ^87^ | 2022 | Scrub Typhus: An Emerging Etiology among Undiagnosed Febrile Cases in Western Part of Uttar Pradesh, India | English | India | India | 2017 | 2020 |
| 122 | Tandale BV, | 2022 | Infectious causes of acute encephalitis syndrome hospitalizations in Central India, 2018–20. | English | India | India | 2018 | 2020 |
| 123 | Mondal, T., | 2022 | A study of scrub typhus in a medical college hospital in West Bengal, India. | English | India | India | 2019 | 2020 |
| 124 | Devamani, C. S., | 2022 | The incidence of Orientia tsutsugamushi infection in rural South India. | English | India | India | 2018 | 2019 |
| 125 | Narang, R. et al. | 2022 | Scrub typhus in urban areas of Wardha district in central India. | English | India | India | 2015 | 2016 |
| 126 | Bhatia, M. et al. | 2022 | Serological evidence of human leptospirosis in patients with acute undifferentiated febrile illness from Uttarakhand, India: A pilot study. | English | India | India | 2017 | 2017 |
| 127 | Nanaware, N. et al. | 2022 | Genotypic characterization of Orientia tsutsugamushi isolated from acute encephalitis syndrome and acute febrile illness cases in the Gorakhpur area, Uttar Pradesh, India. | English | India | India | 2018 | 2018 |
| 128 | D'Cruz, S., | 2022 | Geography and prevalence of rickettsial infections in Northern Tamil Nadu, India: a cross-sectional study. | English | India | India | 2017 | 2020 |
| 129 | Baidya, A., | 2022 | Prevalence, clinico-laboratory features, and the functional outcome of children with scrub typhus meningoencephalitis—a cohort study. | English | India | India | 2019 | 2021 |
| 130 | Sharma, S.,. | 2022 | Seasonal, Regional and Demographic Trends in Patients with Acute Undifferentiated Fever in Northern India. | English | India | India | 2019 | 2019 |
| 131 | Bhowmick, I. P. et al. | 2022 | Diagnosis of Indigenous Non-Malarial Vector-Borne Infections from Malaria Negative Samples from Community and Rural Hospital Surveillance in Dhalai District, Tripura, North-East India. | English | India | India | 2019 | 2020 |
| 132 | Wolff JW, De Graaf W^88^ | 1939 | Two New Cases of Mite Fever in Java | Dutch | Indonesia | Indonesia | 1947 | 1948 |
| 133 | Dennis DT, et.al. ^89^ | 1981 | A survey of scrub and murine typhus in the Ancol section of Jakarta, Indonesia. Southeast Asian | English | Indonesia | Indonesia | 1977 | 1977 |
| 134 | Gandahusada S, et al.^90^ | 1981 | Infectious disease risks to transmigrant communities in Indonesia: A survey in Lampung province, Sumatra. | English | Indonesia | Indonesia | 1975 | 1975 |
| 135 | Hadi TR, et.al. ^91^ | 1984 | Ekologi penyakit scrub typhus di Unit Desa Transmigrasi Mulyorejo, Way Abung III, Lampung Utara. | Indonesian | Indonesia | Indonesia | 1982 | 1982 |
| 136 | Corwin, A. L. et.al. ^92^ | 1997 | Short report: surveillance of rickettsial infections in Indonesian military personnel during peace keeping operations in Cambodia | English | Indonesia | Indonesia | 1992 | 1993 |
| 137 | Richards, A. L. et.al. ^93^ | 1997 | Seroepidemiologic evidence for murine and scrub typhus in Malang, Indonesia | English | Indonesia | Indonesia | 1994 | 1994 |
| 138 | Richards, Allen L. et.al. ^94^ | 2003 | Serologic evidence of infection with ehrlichiae and spotted fever group rickettsiae among residents of Gag Island, Indonesia | English | Indonesia | Indonesia | nan | nan |
| 139 | Punjabi, Narain H. et.al. ^95^ | 2012 | Etiology of acute, non-malaria, febrile illnesses in Jayapura, northeastern Papua, Indonesia | English | Indonesia | Indonesia | 1997.11 | 2000.02 |
| 140 | Lokida, Dewi, et.al. ^96^ | 2020 | Underdiagnoses of Rickettsia in patients hospitalized with acute fever in Indonesia: observational study results | English | Indonesia | Indonesia | 2013 | 2016 |
| 141 | Hamzah, I. H. et.al. ^97^ | 2020 | Comparative study between serological tests and PCR for diagnosis of Rickettsial diseases in patients with fever of unknown origin | English | Iraq | Iraq | nan | nan |
| 142 | Olson, J. G. et.al. ^98^ | 1979 | Seroepidemiological evidence of infectious diseases in United States marine corps personnel, Okinawa, Japan, 1975-1976 | English | USA | Japan | 1975 | 1976 |
| 143 | Miyairi, T. et.al. ^99^ | 1980 | [Shichito fever in Izu Shichito Islands. 4. Incidence of anti-R. tsutsugamushi antibody among inhabitant's sera (author's transl)] | Japanese | Japan | Japan | 1978 | 1978 |
| 144 | Takada, N. et.al. ^100^ | 1984 | [Epidemiology of tsutsugamushi disease in Hokuriku district, Japan. 1. Prevalence of antibody to Rickettsia tsutsugamushi] | Japanese | Japan | Japan | 1983 | 1983 |
| 145 | Kasuya, S. et.al. ^101^ | 1985 | [Studies on tsutsugamushi disease in Gifu Prefecture. I. Isolation of Rickettsia tsutsugamushi from wild rodents at a new area and epidemiological studies on patients and latent patients in the prefecture] | Japanese | Japan | Japan | 1982 | 1983 |
| 146 | Ishikura, M. et.al. ^102^ | 1987 | Epidemiological studies on the background of the endemic occurrence of tsutsugamushi disease in Toyama Prefecture. II. Anti-Rickettsia tsutsugamushi antibody-positive rate in inhabitants of endemic and nonendemic areas | English | Japan | Japan | 1978.12 | 1980.12 |
| 147 | Sando, Eiichiro, et.al. ^103^ | 2018 | Distinguishing Japanese Spotted Fever and Scrub Typhus, Central Japan, 2004- 2015 | English | Japan | Japan | 2011 | 2015 |
| 148 | Thiga, Jacqueline W. et.al. ^104^ | 2015 | High seroprevalence of antibodies against spotted fever and scrub typhus bacteria in patients with febrile Illness, Kenya | English | Kenya | Kenya | 2009 | 2013 |
| 149 | Maina, A. N. et.al. ^105^ | 2016 | Q fever, scrub typhus, and rickettsial diseases in children, Kenya, 2011-2012 | English | USA | Kenya | 2011 | 2012 |
| 150 | Phongmany, Simaly. et.al. ^106^ | 2006 | Rickettsial infections and fever, Vientiane, Laos | English | Laos | Laos | 2001 | 2003 |
| 151 | Vallee, Julie, et.al. ^107^ | 2010 | Contrasting spatial distribution and risk factors for past infection with scrub typhus and murine typhus in Vientiane City, Lao PDR | English | France | Laos | 2006.02 | 2006.03 |
| 152 | Syhavong B, et al. | 2010 | The infective causes of hepatitis and jaundice amongst hospitalised patients in Vientiane, Laos. | English | Laos | Laos | 2001 | 2004 |
| 153 | Mayxay, Mayfong, et.al. ^108^ | 2013 | Causes of non-malarial fever in Laos: a prospective study | English | Laos | Laos | 2008.05.02/2008.09.05 | 2010.12.28/2010.12.26 |
| 154 | Dittrich, Sabine, et.al. ^109^ | 2015 | Orientia, rickettsia, and leptospira pathogens as causes of CNS infections in Laos: a prospective study | English | Laos | Laos | 2003.01.10 | 2011.11.25 |
| 155 | Mayxay M, et al. | 2015 | Causes of fever in rural southern Laos | English | Laos | Laos | 2003 | 2004 |
| 156 | Phetsouvanh R, et al. | 2015 | The diversity and geographical structure of orientia tsutsugamushi strains from scrub typhus patients in Laos | English | Laos | Laos | 2008 | 2012 |
| 157 | Mayxay M, et al. | 2015 | Causes of fever in rural southern Laos | English | Laos | Laos | 2003 | 2004 |
| 158 | Chansamouth, Vilada, et.al. ^110^ | 2016 | The Aetiologies and Impact of Fever in Pregnant Inpatients in Vientiane, Laos | English | Laos | Laos | 2006 | 2010 |
| 159 | Roberts, Tamalee, et.al. | 2021 | A spatio-temporal analysis of scrub typhus and murine typhus in Laos; implications from changing landscapes and climate | English | Lao | Laos | 2003.5 | 2017.1 |
| 160 | 高韵苕, et.al.^111^ | 1960 | 福建××岛恙虫病流行病学调查 | Chinese | Mainland China | Mainland China | 1953 | 1955 |
| 161 | 刘国栋, et.al.^112^ | 1987 | 吉林省珲春地区恙虫病及其它立克次体病调查 | Chinese | Mainland China | Mainland China | 1986 | 1986 |
| 162 | 耿际泉,et.al.^113^ | 1987 | 云南东南部立克次体病血清学调查与病原体分离 | Chinese | Mainland China | Mainland China | nan | nan |
| 163 | 张鸿武 ^114^ | 1991 | 恙虫病患者和流行区健康人的外斐氏反应结果分析 | Chinese | Mainland China | Mainland China | 1989 | 1990 |
| 164 | 刘国栋, et,al.^115^ | 1992 | 獐子岛部分健康人群恙虫病自然感染状况调查 | Chinese | Mainland China | Mainland China | 1989.08 | 1989.08 |
| 165 | Lu, Z. et.al. ^116^ | 1994 | A new natural focus of scrub typhus found in Hunchun | Chinese | Mainland China | Mainland China | 1992.05 | 1992.06 |
| 166 | 王均利, et.al. ^117^ | 1996 | 蒙阴县恙虫病调查报告 | Chinese | Mainland China | Mainland China | 1986 | 1989 |
| 167 | 胡玲美, et.al. ^118^ | 1996 | 东北部分地区恙虫病血清学调查 | Chinese | Mainland China | Mainland China | 1991 | 1994 |
| 168 | Yang, Z. Q., et.al. ^119^ | 1997 | [Studies on clinical epidemiology of Tsutsugamushi disease of the autumn-winter type in the easten suburbs of Jinan] | Chinese | Mainland China | Mainland China | 1988 | 1990 |
| 169 | 王如德, 怀燕 | 1997 | 平邑县恙虫病暴发流行病学调查报告 | Chinese | China | Mainland China | 1996 | 1997 |
| 170 | 周剑平, et.al.^120^ | 1997 | 莆田市98例恙虫病流行病学调查报告 | Chinese | Mainland China | Mainland China | nan | nan |
| 171 | 郑乡占, et.al. ^121^ | 1997 | 山西南部恙虫病流行病学调查 | Chinese | Mainland China | Mainland China | 1994 | 1995 |
| 172 | 黄昭穗, et.al. ^122^ | 1998 | 南日岛健康人群恙虫病血清学调查 | Chinese | Mainland China | Mainland China | 1997 | 1997 |
| 173 | 鲁志新, et.al. ^123^ | 1999 | 东北三省部分地区人血清恙虫病立克次体抗体调查 | Chinese | Mainland China | Mainland China | 1997 | nan |
| 174 | 刘运喜, et.al. ^124^ | 1999 | 山东费县秋冬型恙虫病疫源地调查研究 | Chinese | Mainland China | Mainland China | 1995.05 | 1997.04 |
| 175 | Wang, S.et.al. ^125^ | 2000 | [The recognition of the epidemic area of tsutsugamushi disease on Nan Peng Lie Island in China and the strategy of prevention] | Chinese | Mainland China | Mainland China | 1998.05 | 1999.04 |
| 176 | 陈香蕊, et.al. ^126^ | 2000 | 山西省恙虫病调查与病原学研究 | Chinese | Mainland China | Mainland China | 1995 | 1995 |
| 177 | 唐天开, et.al. ^127^ | 2001 | 南海某珊瑚岛恙虫病流行病学调查 | Chinese | Mainland China | Mainland China | nan | nan |
| 178 | 袁庆虹,et.al. ^128^ | 2001 | 云南大理地区发热病人恙虫病抗体调查 | Chinese | Mainland China | Mainland China | 1998 | 1999 |
| 179 | 陈素良, et.al. ^129^ | 2001 | 河北某村恙虫病发病特点的流行病学研究 | Chinese | Mainland China | Mainland China | 1997 | 1997 |
| 180 | 陈素良, et.al. ^130^ | 2001 | 河北省恙虫病的血清流行病学研究 | Chinese | Mainland China | Mainland China | nan | nan |
| 181 | 林碧瑚, et.al. ^131^ | 2003 | 海南岛立克次体血清学和病原学调查报告 | Chinese | Mainland China | Mainland China | 1993 | 1993 |
| 182 | 袁高林,et.al. ^132^ | 2003 | 宁德市恙虫病地理流行病学调查 | Chinese | Mainland China | Mainland China | 1981 | 1981 |
| 183 | 操敏, et.al. ^133^ | 2004 | 福建省平潭岛春季恙虫病流行病学调查研究 | Chinese | Mainland China | Mainland China | 2002 | 2002 |
| 184 | 吴志伟, et.al. ^134^ | 2005 | 河南省立克次体流行病学调查 | Chinese | Mainland China | Mainland China | nan | nan |
| 185 | Hengbin, Guo, et.al. ^135^ | 2006 | The foci of scrub typhus and strategies of prevention in the Spring in Pingtan Island, Fujian Province | English | Mainland China | Mainland China | nan | nan |
| 186 | 周建荣, et.al. ^136^ | 2007 | 106例感染性疾病致不明原因长期发热病因分析 | Chinese | Mainland China | Mainland China | 2001.01 | 2006.04 |
| 187 | 尹骏 and展海燕^137^ | 2007 | 靖江市人群恙虫病感染情况调查 | Chinese | Mainland China | Mainland China | 2004 | 2004 |
| 188 | 张倩, et.al. ^138^ | 2007 | 内蒙古、新疆地区恙虫病流行病学调查研究 | Chinese | Mainland China | Mainland China | 2005 | 2006 |
| 189 | 吴中发, et.al. ^139^ | 2008 | 江西赣州市恙虫病的调查研究 | Chinese | Mainland China | Mainland China | 1998 | 2007 |
| 190 | 林光宇, et.al. ^140^ | 2008 | 福建省北部林区人群人粒细胞无形体血清流行病学检测 | Chinese | Mainland China | Mainland China | 2007 | 2007 |
| 191 | 王珊珊, et.al. ^141^ | 2008 | 广东省硇州岛恙虫病疫源地的证实 | Chinese | Mainland China | Mainland China | 2003 | 2003 |
| 192 | Liu YX, et al. | 2009 | Clinical characteristics of the autumn-winter type scrub typhus cases in south of Shandong province, northern China | English | China | Mainland China | 1995 | 2006 |
| 193 | Chai, Cheng-liang, et.al. ^142^ | 2010 | [Sero-epidemiologic investigation on tick-borne diseases of humans and domestic animals in Zhejiang province] | Chinese | Mainland China | Mainland China | 2009.03 | 2009.05 |
| 194 | 刘红, et.al. ^143^ | 2010 | 安徽省不同地区人群和家畜恙虫病血清流行病学调查 | Chinese | Mainland China | Mainland China | 2009 | 2009 |
| 195 | 薛家芹, et.al. ^144^ | 2010 | 环泰山区域恙虫病东方体感染状况调查及基因序列分析 | Chinese | Mainland China | Mainland China | 2002 | 2007 |
| 196 | 何似, 谢忠杭, 陈阳,陈亮,邓艳琴,王灵岚, | 2011 | 福建省2006-2009年恙虫病流行特征分析 | Chinese | Mainland China | Mainland China | 2006 | 2009 |
| 197 | 张颖, et.al. ^145^ | 2011 | 天津市农牧地区2007—2009年莫氏立克次体、横赛巴尔通体、恙虫病东方体血清流行病学调查 | Chinese | Mainland China | Mainland China | 2007 | 2009 |
| 198 | 苏静静 and 杨占清^146^ | 2011 | 济南战区（山东）部分驻地秋冬型恙虫病流行病学特征与预防研究 | Chinese | Mainland China | Mainland China | nan | nan |
| 199 | 姜仁杰, et.al. ^147^ | 2011 | 盐城市2006-2010年恙虫病流行病学研究 | Chinese | Mainland China | Mainland China | 2006 | 2010 |
| 200 | 谭兆营, et.al. ^148^ | 2012 | 江苏省几种主要类型人畜立克次体抗体阳性检出情况的现况调查 | Chinese | Mainland China | Mainland China | nan | nan |
| 201 | Liu YX, et al. | 2013 | Consistency of the Key Genotypes of Orientia tsutsugamushi in Scrub Typhus Patients, Rodents, and Chiggers from a New Endemic Focus of Northern China. | English | Mainland China | Mainland China | 1995 | 2006 |
| 202 | 常利涛, et.al. ^149^ | 2013 | 云南省人和家畜立克次体病血清流行病学调查(英文) | English | Mainland China | Mainland China | 2009 | 2009 |
| 203 | 张立芹, et.al. ^150^ | 2013 | 北京市平谷区常住人口恙虫病感染状况调查 | Chinese | Mainland China | Mainland China | 2012 | 2012 |
| 204 | 田丽丽, et.al. ^151^ | 2013 | 北京市恙虫病病例流行病学特征及实际感染病例数估计 | Chinese | Mainland China | Mainland China | 2008 | 2012 |
| 205 | 邱尔臣, et.al. ^152^ | 2013 | 新疆部分地区人群恙虫病血清流行病学调查 | Chinese | Mainland China | Mainland China | nan | nan |
| 206 | 李静, et.al.^153^ | 2014 | 云南省某大学新生中莱姆病和恙虫病的血清流行病学调查 | Chinese | Mainland China | Mainland China | 2008 | 2008 |
| 207 | 申安然, et.al.^154^ | 2014 | 陇川县2009-2013年恙虫病流行现状分析 | Chinese | Mainland China | Mainland China | 2009 | 2013 |
| 208 | De, Wu, et.al. | 2015 | Scrub typhus, a disease with increasing threat in Guangdong, China | English | China | Mainland China | 2012 | 2014 |
| 209 | 谭文文, et.al.^155^ | 2015 | 宜兴市一般人群立克次体病血清流行病学调查 | Chinese | Mainland China | Mainland China | 2009.10 | 2009.10 |
| 210 | 韩腾伟, et.al.^156^ | 2015 | 福建省2010—2013年恙虫病流行特征分析 | Chinese | Mainland China | Mainland China | 2010 | 2013 |
| 211 | 吕燕宁, et.al.^157^ | 2016 | 北京市远郊区恙虫病血清流行病学调查 | Chinese | Mainland China | Mainland China | 2012 | 2013 |
| 212 | 张曙光, et.al.^158^ | 2016 | 北京市延庆区恙虫病人群感染状况与危险因素研究 | Chinese | Mainland China | Mainland China | 2011 | 2011 |
| 213 | 陈永亮, et.al.^159^ | 2016 | 北京市北部山区恙虫病血清流行病学调查分析 | Chinese | Mainland China | Mainland China | 2011 | 2011 |
| 214 | 展海燕, et.al.^160^ | 2017 | 江苏省靖江市2011-2015年恙虫病流行特征及影响因素 | Chinese | Mainland China | Mainland China | 2011 | 2015 |
| 215 | 张欢, et.al.^161^ | 2018 | 广州市2015年流行性出血热抗体阴性样本病原谱分析 | Chinese | Mainland China | Mainland China | 2015 | 2015 |
| 216 | 张立芹, et.al.^162^ | 2018 | 北京市平谷区农村社区医院恙虫病筛查结果分析 | Chinese | Mainland China | Mainland China | 2016 | 2016 |
| 217 | 李飒, et.al.^163^ | 2019 | 北京市昌平区恙虫病血清流行病学调查及危险因素分析 | Chinese | Mainland China | Mainland China | 2017 | 2017 |
| 218 | Gu, X. L. et.al.^164^ | 2021 | Misdiagnosis of scrub typhus as hemorrhagic fever with renal syndrome and potential coinfection of both diseases in patients in shandong province, china, 2013-2014 | English | Mainland China | Mainland China | 2013 | 2014 |
| 219 | Rainey, J. J. et al. | 2022 | Etiology of acute febrile illnesses in Southern China: Findings from a two-year sentinel surveillance project, 2017–2019. | English | China | Mainland China | 2017 | 2019 |
| 220 | Cadigan, F. C., et.al.^165^ | 1972 | The effect of habitat on the prevalence of human scrub typhus in Malaysia | English | Malaysia | Malaysia | nan | nan |
| 221 | Brown, G. W. et.al.^166^ | 1976 | Scrub typhus: a common cause of illness in indigenous populations | English | Malaysia | Malaysia | 1975 | 1975 |
| 222 | Brown, G. W. et.al.^167^ | 1978 | Serological evidence for a high incidence of transmission of Rickettsia tsutsugamushi in two Orang Asli settlements in Peninsular Malaysia | English | Malaysia | Malaysia | 1975 | 1975 |
| 223 | Brown GW, et.al. | 1978 | Single dose doxycycline therapy for scrub typhus | English | Malaysia | Malaysia | 1976 | 1977 |
| 224 | Shirai A, et.al. | 1981 | Rickettsia tsutsugamushi antibody in mother/cord pairs of sera. | English | Malaysia | Malaysia | 1980 | 1980 |
| 225 | Brown, G. W. et.al.^168^ | 1984 | Febrile illness in Malaysia--an analysis of 1,629 hospitalized patients | English | Malaysia | Malaysia | 1975 | 1979 |
| 226 | Taylor, A. et.al.^169^ | 1986 | An analysis of febrile illnesses among members of the Malaysian Police Field Force | English | Malaysia | Malaysia | 1983.03 | 1984.02 |
| 227 | Tee, T. S. et.al.^170^ | 1999 | Seroepidemiologic survey of Orientia tsutsugamushi, Rickettsia typhi, and TT118 spotted fever group rickettsiae in rubber estate workers in Malaysia | English | Malaysia | Malaysia | 1996 | 1997 |
| 228 | Sagin, D. et.al.^171^ | 2000 | Rickettsial infection in five remote Orang Ulu villages in upper Rejang River, Sarawak, Malaysia | English | Malaysia | Malaysia | nan | nan |
| 229 | Tay, S. T. et.al.^172^ | 2000 | Antibodies to Orientia tsutsugamushi, Rickettsia typhi and spotted fever group rickettsiae among febrile patients in rural areas of Malaysia | English | Malaysia | Malaysia | 1995 | 1997 |
| 230 | Tay, S. T. et.al.^173^ | 2003 | Antibody prevalence of Orientia tsutsugamushi, Rickettsia typhi and TT118 spotted fever group rickettsiae among Malaysian blood donors and febrile patients in the urban areas | English | Malaysia | Malaysia | 1998 | 1999 |
| 231 | Tay, Sun Tee et.al.^174^ | 2013 | Antibody prevalence and factors associated with exposure to Orientia tsutsugamushi in different aboriginal subgroups in West Malaysia | English | Malaysia | Malaysia | 2007.11 | 2010.08 |
| 232 | Grigg, Matthew J., et.al. ^175^ | 2020 | Rickettsioses as Major Etiologies of Unrecognized Acute Febrile Illness, Sabah, East Malaysia | English | Malaysia | Malaysia | 2013 | 2015 |
| 233 | Elders, Philip N. D. et.al. ^176^ | 2021 | Serological evidence indicates widespread distribution of rickettsioses in Myanmar | English | Myanmar | Myanmar | 2019 | 2019 |
| 234 | Brown GW, et.al. | 1981 | Antibodies to typhus in Eastern Nepal. | English | Nepal | Nepal | 1978 | 1979 |
| 235 | Murdoch, David R. et.al. ^177^ | 2004 | The etiology of febrile illness in adults presenting to Patan hospital in Kathmandu, Nepal | English | New Zealand | Nepal | 2001 | 2001 |
| 236 | Blacksell, Stuart D. et.al. ^178^ | 2007 | Serological and blood culture investigations of Nepalese fever patients | English | Thailand | Nepal | nan | nan |
| 237 | Upadhyaya, B. et.al. | 2016 | Scrub Typhus: An Emerging Neglected Tropical Disease in Nepal | English | Nepal | Nepal | 2015 | 2015 |
| 238 | Sedhain, A. and Bhattarai, G. R.^179^ | 2017 | Renal Manifestation in Scrub Typhus during a Major Outbreak in Central Nepal | English | Nepal | Nepal | 2016 | 2016 |
| 239 | Pathak, Santosh, et.al. | 2019 | Clinical profile, complications and outcome of scrub typhus in children: A hospital based observational study in central Nepal | English | Nepal | Nepal | 2016.07 | 2017.08 |
| 240 | Dhimal, Meghnath,et.al. | 2019 | Confirmation of local transmission of Orientia tsutsugamushi during scrub typhus outbreaks in Nepal | English | Nepal | Nepal | 2015 | 2017 |
| 241 | Gautam, Rajendra, et.al. ^180^ | 2019 | Epidemiology, Risk Factors and Seasonal Variation of Scrub Typhus Fever in Central Nepal | English | Nepal | Nepal | 2017.04 | 2018.03 |
| 242 | Sharma, K., et.al. ^181^ | 2019 | Prevalence and knowledge of scrub typhus in patients with acute febrile illness in teaching hospital, Chitwan, Nepal | English | Nepal | Nepal | 2018 | 2018 |
| 243 | Thapa, Sangita, et.al. ^182^ | 2020 | Burden of scrub typhus among patients with acute febrile illness attending tertiary care hospital in Chitwan, Nepal | English | Nepal | Nepal | 2016.07 | 2017.06 |
| 244 | Gautam, Rajendra, et.al. | 2020 | Diagnostic evaluation of IgM ELISA and IgM Immunofluorescence assay for the diagnosis of Acute Scrub Typhus in central Nepal | English | Nepal | Nepal | 2017 | 2018 |
| 245 | Gautam, R.et.al. | 2021 | Scrub typhus and molecular characterization of orientia tsutsugamushi from central nepal | English | Nepal | Nepal | 2018 | 2018 |
| 246 | Pokhrel, Anil, et.al. ^183^ | 2021 | Seroprevalence and Clinical Features of Scrub Typhus among Febrile Patients Attending a Referral Hospital in Kathmandu, Nepal | English | Nepal | Nepal | 2018 | 2019 |
| 247 | Madhup, S. K. et.al. | 2021 | Seroprevalence of Scrub Typhus in Patients Attending Dhulikhel Hospital, Kavre | English | Nepal | Nepal | 2019 | 2021 |
| 248 | Shrestha, S. et al. | 2022 | Clinical profile and biochemical abnormalities in Scrub Typhus: A cross-sectional study. | English | Nepal | Nepal | 2019 | 2022 |
| 249 | Demma, Linda J., et.al. ^184^ | 2006 | Scrub typhus, Republic of Palau | English | USA | Palau | 2003 | 2003 |
| 250 | Spicer, P. E., et.al. ^185^ | 2007 | Scrub typhus (Orientia tsutsugamushi), spotted fever (Rickettsia australis) and dengue fever as possible causes of mysterious deaths in the Strickland Gorge area of Southern Highlands and West Sepik Provinces of Papua New Guinea | English | Australia | Papua New Guinea | 2001 | 2001 |
| 251 | Kocher, C., et.al. ^186^ | 2017 | Serologic evidence of scrub typhus in the peruvian Amazon | English | Switzerland | Peru | 2013 | 2013 |
| 252 | Yen, Tsai-Ying, et.al. ^187^ | 2019 | Serologic Evidence for Orientia Exposure in the Democratic Republic of Sao Tome and Principe | English | Taiwan | Sao Tome and Principe | 2016 | 2016 |
| 253 | Muzaheed, et.al. ^188^ | 2020 | Hospital based serological evidence of rickettsial diseases and assessment diagnostic tests of pyrexia of unknown origin | English | Saudi Arabia | Saudi Arabia | nan | nan |
| 254 | Miles, J. A. R., et.al. | 1981 | Scrub typhus in the Eastern Solomon Islands and Northern Vanuatu (New Hebrides) | English | New Zealand | Solomon Islands and Vanuatu | 1971 | 1972 |
| 255 | Chang, W. H., et.al. ^189^ | 1989 | Seroepidemiological survey of tsutsugamushi disease in Korea, 1987 and 1988 | Korean | South Korea | South Korea | 1987 | 1988 |
| 256 | Chang, W. H., et.al. ^190^ | 1990 | Seroepidemiological survey of Tsutsugamushi disease in Korea, 1989 | Korean | South Korea | South Korea | 1989 | 1989 |
| 257 | Jin-Won, Song, et.al. ^191^ | 1998 | Seroepidemiologic Analysis of Acute Febrile Illness During 1994-1995 in Korea | Korean | South Korea | South Korea | 1994 | 1995 |
| 258 | Jin-Won, Song, et.al. ^192^ | 1998 | Seroepidemiologic Analysis of Acute Febrile Illness from Korea in 1996 | Korean | South Korea | South Korea | 1996 | 1996 |
| 259 | Min-Ja, K. I. M., et.al. ^193^ | 2001 | A Prospective Multicenter Study of the Etiological Analysis in Adults with Community-Acquired Pneumonia: Legionella, Leptospira, Hantaan virus and Orientia tsutsugamushi | Korean | South Korea | South Korea | 1997 | 2000 |
| 260 | Song, J. W., et.al. ^194^ | 2002 | Seroepidemiologic analysis of acute febrile illness in Korea during 1997-1998 | Korean | South Korea | South Korea | 1997 | 1998 |
| 261 | Jang, Won-Jong, et.al. ^195^ | 2004 | First serologic evidence of human spotted fever group rickettsiosis in Korea | English | South Korea | South Korea | 1992.12 | 1993.11 |
| 262 | Jung, Ho-Chul, et.al. ^196^ | 2015 | Etiologies of acute undifferentiated fever and clinical prediction of scrub typhus in a non-tropical endemic area | English | South Korea | South Korea | 2009 | 2013 |
| 263 | Dong-Seob, K. I. M., et.al.^197^ | 2019 | A Study on the Febrile Illness in Autumn among Farmers in Gyeongju-si: Scrub typhus, Leptospirosis, HFRS | Korean | South Korea | South Korea | 2014 | 2015 |
| 264 | Hwang JH, et.al. | 2019 | High seroprevalence of mycoplasma pneumoniae and Chlamydia pneumoniae among scrub typhus patients in South Korea. | English | South Korea | South Korea | 2016 | 2017 |
| 265 | Yen NTH, et.al. | 2019 | Severe fever with thrombocytopenia syndrome virus infection or mixed infection with scrub typhus in South Korea in 2000-2003. | English | South Korea | South Korea | 2000 | 2003 |
| 266 | Park, Ji-Hyuk, et.al. ^198^ | 2021 | Seroprevalence and Factors Associated with Scrub Typhus Infection among Forestry Workers in National Park Offices in South Korea | English | South Korea | South Korea | 2016 | 2016 |
| 267 | Um, J., et.al. ^199^ | 2021 | Seroprevalence of scrub typhus, murine typhus and spotted fever groups in North Korean refugees | English | South Korea | South Korea | 2019 | 2019 |
| 268 | Premaratna R, et.al. | 2008 | Rickettsial infections and their clinical presentations in the Western Province of Sri Lanka: a hospital-based study. | English | Sri Lanka | Sri Lanka | 2002 | 2003 |
| 269 | Liyanapathirana, Veranja, et.al. ^200^ | 2011 | Seroepidemiology of rickettsioses in Sri Lanka: a patient based study | English | Sri Lanka | Sri Lanka | 2007 | 2011 |
| 270 | Kularatne, S. A. M., et.al. ^201^ | 2013 | Rickettsioses in the central hills of Sri Lanka: serological evidence of increasing burden of spotted fever group | English | Sri Lanka | Sri Lanka | 2002 | 2007 |
| 271 | Premaratna, Ranjan, et.al. ^202^ | 2014 | Rickettsial infection among military personnel deployed in Northern Sri Lanka | English | Sri Lanka | Sri Lanka | 2008 | 2009 |
| 272 | Pradeepan JA, et.al. | 2014 | Emerging scrub typhus infection in the northern region of Sri Lanka | English | Sri Lanka | Sri Lanka | 2012 | 2013 |
| 273 | Gayani Tillekeratne L, et al. | 2020 | Previously derived host gene expression classifiers identify bacterial and viral etiologies of acute febrile respiratory illness in a south asian population. | English | USA | Sri Lanka | 2012 | 2013 |
| 274 | Bourgeois, A. L. et.al. | 1977 | Epidemiological and serological study of scrub typhus among Chinese military in the Pescadores islands of Taiwan | English | USA | Taiwan | 1975 | 1975 |
| 275 | Olson, J. G. and Bourgeois, A. L.^203^ | 1977 | Rickettsia tsutsugamushi infection and scrub typhus incidence among Chinese military personnel in the Pescadores Islands | English | Taiwan | Taiwan | 1976 | nan |
| 276 | Yang, Hui-Hua,et.al. | 2012 | New genotypes of Orientia tsutsugamushi isolated from humans in Eastern Taiwan | English | China | Taiwan | 2002 | 2008 |
| 277 | Lai CH, et al. | 2014 | Human spotted fever group rickettsioses are underappreciated in Southern Taiwan, particularly for the species closely-related to Rickettsia felis. | English | Taiwan | Taiwan | 2004 | 2009 |
| 278 | Tsai KH, et al. | 2019 | Human granulocytic anaplasmosis in Kinmen, an offshore island of Taiwan. | English | Taiwan | Taiwan | 2011 | 2012 |
| 279 | Johnson, D. E., et.al. ^204^ | 1982 | Sero-epidemiological survey of Rickettsia tsutsugamushi infection in a rural Thai village | English | Thailand | Thailand | 1976 | 1976 |
| 280 | Duffy, P. E., et.al. ^205^ | 1990 | Murine typhus identified as a major cause of febrile illness in a camp for displaced Khmers in Thailand | English | Thailand | Thailand | 1988 | 1988 |
| 281 | Silpapojakul, K., et.al. ^206^ | 1991 | Scrub and murine typhus in children with obscure fever in the tropics | English | Thailand | Thailand | 1985 | 1987 |
| 282 | Strickman, D., et.al. ^207^ | 1994 | Prevalence of antibodies to rickettsiae in the human population of suburban Bangkok | English | Thailand | Thailand | nan | nan |
| 283 | Eamsila, C., et.al.^208^ | 1996 | Antibodies to Orientia tsutsugamushi in Thai soldiers | English | Thailand | Thailand | 1989 | 1991 |
| 284 | Frances, S. P., et.al. ^209^ | 1997 | Antibodies to Orientia tsutsugamushi in soldiers in northeastern Thailand | English | Thailand | Thailand | 1991.09 | 1992.10 |
| 285 | Chanyasanha, C., et.al. ^210^ | 1998 | Seroprevalence of scrub typhus infection in patients with pyrexia at some malaria clinics in three western provinces of Thailand | English | Thailand | Thailand | 1994 | 1994 |
| 286 | Tanskul P, et al. | 1998 | A New Ecology for Scrub Typhus Associated with a Focus of Antibiotic Resistance in Rice Farmers in Thailand. | English | Thailand | Thailand | 1993 | 1995 |
| 287 | Chinprasatsak S, et al. | 2001 | Evaluation of a newly developed dipstick test for the rapid diagnosis of scrub typhus in febrile patients. | English | Thailand | Thailand | 1995 | 1996 |
| 288 | Thap LC, et.al. | 2002 | Septic shock secondary to scrub typhus: Characteristics and complications. | English | Cambodia | Thailand | 2001 | 2002 |
| 289 | Pradutkanchana, Jintana, et.al. ^211^ | 2003 | The etiology of acute pyrexia of unknown origin in children after a flood | English | Thailand | Thailand | 2000 | 2000 |
| 290 | Leelarasamee, Amorn,et.al. | 2004 | Etiologies of acute undifferentiated febrile illness in Thailand | English | Thailand | Thailand | 1991 | 1993 |
| 291 | T. Singhsilarak, S., et al. | 2006 | Possible acute coinfections in Thai malaria patients | English | Thailand | Thailand | na | na |
| 292 | Suttinont C, et al. | 2006 | Causes of acute, undifferentiated, febrile illness in rural Thailand: Results of a prospective observational study | English | Thailand | Thailand | 2001 | 2002 |
| 293 | Suputtamongkol, Y., et.al. ^212^ | 2009 | Epidemiology and clinical aspects of rickettsioses in Thailand | English | Thailand | Thailand | 2000.10 | 2003.03 |
| 294 | Thitivichianlert, Sataporn, et.al. ^213^ | 2009 | Incidence of rickettsial infection in patients with acute fever in provincial Thai army hospitals | English | Thailand | Thailand | 2005.06 | 2006.10 |
| 295 | McGready R, et al. | 2010 | Arthropod borne disease: the leading cause of fever in pregnancy on the Thai-Burmese border | English | Thailand | Thailand | 2004 | 2006 |
| 296 | Wuttikon Rodkvamtook, et.al. | 2011 | Isolation and Characterization of Orientia tsutsugamushi from Rodents Captured following a Scrub Typhus Outbreak at a Military Training Base, Bothong District, Chonburi Province, Central Thailand | English | Thailand | Thailand | 2002 | 2002 |
| 297 | Ruang-areerate T, et.al. | 2011 | Genotype diversity and distribution of Orientia tsutsugamushi causing scrub typhus in Thailand. | English | Thailand | Thailand | 2004 | 2007 |
| 298 | W. Rodkvamtook, et al. | 2013 | Scrub typhus outbreak, northern Thailand, 2006-2007 | English | Thailand | Thailand | 2006 | 2007 |
| 299 | Thipmontree, Wilawan, et.al. ^214^ | 2014 | Human leptospirosis trends: northeast Thailand, 2001-2012 | English | Thailand | Thailand | 2001.07 | 2002.12 |
| 300 | Thipmontree, Wilawan, et.al. ^215^ | 2016 | Scrub Typhus in Northeastern Thailand: Eschar Distribution, Abnormal Electrocardiographic Findings, and Predictors of Fatal Outcome | English | Thailand | Thailand | 2011 | 2012 |
| 301 | Bhengsri S, et al. | 2016 | Sennetsu neorickettsiosis, spotted fever group, and typhus group rickettsioses in three provinces in Thailand. | English | Thailand | Thailand | 2002 | 2005 |
| 302 | Hinjoy, S., et.al. ^216^ | 2017 | Zoonotic and vector borne agents causing disease in adult patients hospitalized due to fever of unknown origin in Thailand | English | Thailand | Thailand | 2013 | 2015 |
| 303 | Rodkvamtook, W., et.al. ^217^ | 2018 | Scrub typhus outbreak in Chonburi Province, Central Thailand, 2013 | English | Thailand | Thailand | 2013 | 2013 |
| 304 | Wangrangsimakul, Tri, et.al. ^218^ | 2018 | Causes of acute undifferentiated fever and the utility of biomarkers in Chiangrai, northern Thailand | English | Thailand | Thailand | 2006.08 | 2008.10 |
| 305 | Chaisiri, Kittipong, et.al. ^219^ | 2022 | Risk factors analysis for neglected human rickettsioses in rural communities in Nan province, Thailand: A community-based observational study along a landscape gradient | English | Thailand | Thailand | 2018 | 2018 |
| 306 | Gonwong, Siriphan, et.al.^220^ | 2022 | Nationwide Seroprevalence of Scrub Typhus, Typhus, and Spotted Fever in Young Thai Men | English | Thailand | Thailand | 2007 | 2012 |
| 307 |  | 1998 | Promed Post - ProMED-mail-1998-07-21 | English |  | Vietnam | nan | nan |
| 308 | Nadjm, Behzad, et.al. ^221^ | 2014 | Scrub typhus in the northern provinces of Vietnam: an observational study of admissions to a national referral hospital | English | Vietnam | Vietnam | 2001 | 2003 |
| 309 | Hamaguchi, Sugihiro, et.al.^222^ | 2015 | Clinical and Epidemiological Characteristics of Scrub Typhus and Murine Typhus among Hospitalized Patients with Acute Undifferentiated Fever in Northern Vietnam | English | Japan | Vietnam | 2001.03 | 2003.02 |
| 310 | Nguyen, Hang L. K., et.al.^223^ | 2017 | The genotypes of Orientia tsutsugamushi, identified in scrub typhus patients in northern Vietnam | English | Vietnam | Vietnam | 2015 | 2016 |
| 311 | Trung, Nguyen Vu, et.al. ^224^ | 2017 | Seroprevalence of Scrub Typhus, Typhus, and Spotted Fever Among Rural and Urban Populations of Northern Vietnam | English | Vietnam | Vietnam | 2011 | 2012 |
| 312 | Katoh, Shungo, et.al. ^225^ | 2019 | Challenges in diagnosing scrub typhus among hospitalized patients with undifferentiated fever at a national tertiary hospital in northern Vietnam | English | Japan | Vietnam | 2012.06 | 2013.05 |
| 313 | Le-Viet, Nhiem, et.al. ^226^ | 2019 | Prospective case-control analysis of the aetiologies of acute undifferentiated fever in Vietnam | English | Vietnam | Vietnam | 2016 | 2016 |
| 314 | Trung, Nguyen et.al. | 2019 | Clinical Manifestations and Molecular Diagnosis of Scrub Typhus and Murine Typhus, Vietnam, 2015-2017 | English | Vietnam | Vietnam | 2015.03 | 2017.03 |
| 315 | Trung, N. V. et al. | 2022 | Systematic surveillance of rickettsial diseases in 27 hospitals from 26 provinces throughout Vietnam. | English | Vietnam | Vietnam | 2018 | 2019 |

*Note: nan=not available.*

# Appendix Table S6: Characteristics of included incidence studies, in order of country/region and published year (n=150).

| **ID** | **Author** | **Year of publication** | **Title** | **Language** | **Publication country** | **Study conducted country** | **Start-time of investigation** | **End-time of investigation** |
| --- | --- | --- | --- | --- | --- | --- | --- | --- |
| 1 | Dorji, KezangPhuentshok, YoentenZangpo, TandinDorjee, SitharDorjee, ChenchoJolly, PeterMorris, RogerMarquetoux, NellyMcKenzie, Joanna | 2019 | Clinical and Epidemiological Patterns of Scrub Typhus, an Emerging Disease in Bhutan | English | New Zealand | Bhutan | 2015.1 | 2015.12 |
| 2 | Devamani, Carol S.Prakash, John A. J.Alexander, NealSuzuki, MotoiSchmidt, Wolf-Peter | 2019 | Hospitalisations and outpatient visits for undifferentiated fever attributable to scrub typhus in rural South India: Retrospective cohort and nested case-control study | English | India | India | 2018 | 2018 |
| 3 | Hashimoto, ShujiKawado, MiyukiMurakami, YoshitakaIzumida, MichikoOhta, AkikoTada, YukiShigematsu, MikaYasui, YoshinoriTaniguchi, KiyosuNagai, Masaki | 2007 | Epidemics of vector-borne diseases observed in infectious disease surveillance in Japan, 2000-2005 | English | Japan | Japan | 2000 | 2005 |
| 4 | Kinoshita, HitomiArima, YuzoShigematsu, MikaSunagawa, TomimasaSaijo, MasayukiOishi, KazunoriAndo, Shuji | 2021 | Descriptive epidemiology of rickettsial infections in Japan: Scrub typhus and Japanese spotted fever, 2007-2016 | English | Japan | Japan | 2007 | 2016 |
| 5 | Roberts, TamaleeParker, Daniel M.Bulterys, Philip L.Rattanavong, SayaphetElliott, IvoPhommasone, KoukeoMayxay, MayfongChansamouth, ViladaRobinson, Matthew T.Blacksell, Stuart D.Newton, Paul N. | 2021 | A spatio-temporal analysis of scrub typhus and murine typhus in Laos; implications from changing landscapes and climate | English | Laos | Laos | 2003.5 | 2017.1 |
| 6 | Corwin, A.Soderquist, R.Suwanabun, N.Sattabongkot, J.Martin, L.Kelly, D.Beecham, J. | 1999 | Scrub typhus and military operations in Indochina | English | Indonesia | Laos, Vietnam, Cambodia | 1996 | 1998 |
| 7 | 高韵苕卢玉韵吴光华王福彭梁荫荪赵予秀何尤薛敏荣 | 1960 | 福建××岛恙虫病流行病学调查 | Chinese | Mainland China | Mainland China | 1953 | 1955 |
| 8 | 何子圭 | 1981 | 红河州恙虫病流行病学分析(260例恙虫病例分析) | Chinese | Mainland China | Mainland China | 1952 | 1977 |
| 9 | 苏德茂, 姜仁杰, 王遵道,王伯勋,吉根娣,杨连柏,徐毛华,刘玉,郭桓彬,陈香芯,胥照平,石建时,吴杨生, | 1989 | 东台市恙虫病爆发流行的调查报告 | Chinese | Mainland China | Mainland China | 1987 | 1987 |
| 10 | 张祥浩, 李顺波, 张呈芳, | 1991 | 莒县恙虫病流行病学调查报告 | Chinese | Mainland China | Mainland China | 1989 | 1989 |
| 11 | 雷亚民, 冯锡光, 陈渊民,鲁锦荣, | 1991 | 永德县恙虫病流行因素调查 | Chinese | Mainland China | Mainland China | 1978 | 1987 |
| 12 | 王玉珍 | 1992 | 临沂地区1986～1989年恙虫病流行情况 | Chinese | Mainland China | Mainland China | 1986 | 1989 |
| 13 | 王均利, 杨玉富, 姚允超, | 1993 | 山东一村庄连续5年恙虫病流行 | Chinese | Mainland China | Mainland China | 1986 | 1990 |
| 14 | 戴东方，阎文明，梁福田，武祥善 | 1995 | 费县1988～1992年恙虫病疫情监测 | Chinese | Mainland China | Mainland China | 1988 | 1992 |
| 15 | 王均利，李萍，段爱香，许运珍，姚允超，孟凡彬，刘玉华，王玉琴 | 1996 | 蒙阴县恙虫病调查报告 | Chinese | Mainland China | Mainland China | 1986 | 1989 |
| 16 | 郑乡占, 霍勤, 江志礼,覃顺寿,段林枝,李清, | 1997 | 山西南部恙虫病流行病学调查 | Chinese | Mainland China | Mainland China | 1994 | 1995 |
| 17 | 刘运喜, 吴钦永, 杨占清,彭佐林,苗仲水,秦德太,于仁芝,王刚,王增君,苏明,朱洪霞,郭士成, | 1999 | 费县秋冬型恙虫病流行病学及影响因素调查研究 | Chinese | Mainland China | Mainland China | 1994 | 1997 |
| 18 | 刘运喜, 吴钦永, 杨占清,彭佐林,苗仲水,孟祥瑞,丛乐滋,苏明, | 1999 | 山东费县秋冬型恙虫病疫源地调查研究 | Chinese | Mainland China | Mainland China | 1995.5 | 1997.4 |
| 19 | 袁高林, 陈文锦, 李翔英,李方平, | 2003 | 宁德市恙虫病地理流行病学调查 | Chinese | Mainland China | Mainland China | nan | nan |
| 20 | 王李河 | 2004 | 增城市2001～2003年恙虫病流行情况分析 | Chinese | Mainland China | Mainland China | 2001 | 2003 |
| 21 | 吴爱芬, 方永恩, 许跃文, | 2006 | 霞浦县1957~2005年恙虫病流行病学及临床分析 | Chinese | Mainland China | Mainland China | 1957 | 2005 |
| 22 | Zhang, Li-juanLi, Xue-meiZhang, De-rongZhang, Jing-shanDi, YingLuan, Ming-chunFu, Xiu-ping | 2007 | Molecular epidemic survey on co-prevalence of scrub typhus and marine typhus in Yuxi city, Yunnan province of China | English | Mainland China | Mainland China | 2002 | 2004 |
| 23 | 虞华珍, 蔡衍珊, 曾明深,陈志强,叶双岚,李小向, | 2007 | 一起恙虫病暴发的调查分析 | Chinese | Mainland China | Mainland China | 2006.06 | 2006.07 |
| 24 | 吴中发, 邓海智, 朱丰秀,温江鸿,刘素华, | 2008 | 江西赣州市恙虫病的调查研究 | Chinese | Mainland China | Mainland China | 1998 | 2007 |
| 25 | 杨丽萍, 赵仲堂 | 2008 | 山东地区秋冬型恙虫病流行病学研究 | Chinese | Mainland China | Mainland China | 2006.1.1 | 2006.12.31 |
| 26 | 方益荣, 吴家兵 | 2010 | 2008年安徽省阜阳市恙虫病疫情分析 | Chinese | Mainland China | Mainland China | 2008 | 2008 |
| 27 | 何似, 谢忠杭, 陈阳,陈亮,邓艳琴,王灵岚, | 2011 | 福建省2006-2009年恙虫病流行特征分析 | Chinese | Mainland China | Mainland China | 2006 | 2009 |
| 28 | 姜仁杰, 沈进进, 张盐姝,张红军,郭恒彬,张守印,谈忠鸣, | 2011 | 盐城市2006-2010年恙虫病流行病学研究 | Chinese | Mainland China | Mainland China | 2006 | 2010 |
| 29 | 孙良, 宋秀萍, 万俊峰,朱理业,刘奇泉,杜杰,孙业桓, | 2012 | 安徽省阜阳市2008-2010年恙虫病疫情流行病学分析 | Chinese | Mainland China | Mainland China | 2008 | 2010 |
| 30 | 方益荣, 朱保平, 叶冬青, | 2012 | 安徽省阜阳市恙虫病疫源地调查研究 | Chinese | Mainland China | Mainland China | 2006 | 2010 |
| 31 | 胡文穗, 李美霞, 梁会营,王大虎,邝志光, | 2012 | 广州地区2006-2010年恙虫病流行病学特征分析 | Chinese | Mainland China | Mainland China | 2006 | 2010 |
| 32 | 钱卫娟, 何义林, 尹骏,张翔,戴文军,徐贞,朱赛进, | 2013 | 2011-2012年靖江市恙虫病流行特征分析 | Chinese | Mainland China | Mainland China | 2011 | 2012 |
| 33 | Zhang, W. Y.Wang, L. Y.Ding, F.Hu, W. B.Soares Magalhaes, R. J.Sun, H. L.Liu, Y. X.Liu, Q. Y.Huang, L. Y.Clements, A. C. A.Li, S. L.Li, C. Y. | 2013 | Scrub Typhus in Mainland China, 2006-2012: The Need for Targeted Public Health Interventions | English | Mainland China | Mainland China | 2006 | 2012 |
| 34 | 杨伟, 顾冬梅, 秦成奎, | 2013 | 大丰市2006—2012年恙虫病流行特征分析 | Chinese | Mainland China | Mainland China | 2006 | 2012 |
| 35 | 彭志强, 钟豪杰, 梁文佳, | 2013 | 广东省2006—2011年恙虫病流行病学分析 | Chinese | Mainland China | Mainland China | 2006 | 2011 |
| 36 | 刘隽, 陈邦华, 吴德,刘文华,姚丽君,毛晓庭,肖亮恒,钟豪杰,彭志强, | 2013 | 广东省一起恙虫病暴发疫情流行病学调查 | Chinese | Mainland China | Mainland China | 2011 | 2011 |
| 37 | 李加全, 李杨雪 | 2014 | 2006-2012年保山市恙虫病流行病学分析与防制对策 | Chinese | Mainland China | Mainland China | 2006 | 2012 |
| 38 | 吴玮, 王大虎, 魏跃红, | 2014 | 2012年广州市恙虫病疫情分析 | Chinese | Mainland China | Mainland China | 2011 | 2012 |
| 39 | Wang, T.Yao, Y.Huang, X.Peng, Z. | 2014 | Characteristics of Scrub Typhus epidemic in Guangdong Province from 2006 to 2012 | Chinese | Mainland China | Mainland China | 2006 | 2012 |
| 40 | Yang, L. P.Liu, J.Wang, X. J.Ma, W.Jia, C. X.Jiang, B. F. | 2014 | Effects of meteorological factors on scrub typhus in a temperate region of China | English | Mainland China | Mainland China | 2006.01 | 2012.12 |
| 41 | Li, TiegangYang, ZhicongDong, ZhiqiangWang, Ming | 2014 | Meteorological factors and risk of scrub typhus in Guangzhou, southern China, 2006-2012 | English | Mainland China | Mainland China | 2006 | 2012 |
| 42 | Wei, YuehongHuang, YongLuo, LeiXiao, XincaiLiu, LanYang, Zhicong | 2014 | Rapid increase of scrub typhus: an epidemiology and spatial-temporal cluster analysis in Guangzhou City, Southern China, 2006-2012 | English | Mainland China | Mainland China | 2006 | 2012 |
| 43 | 孙良, 万俊峰, 田亚珍,梁长流,丁振涛,龚磊, | 2014 | 安徽省阜阳市2011～2013年恙虫病流行特征分析 | Chinese | Mainland China | Mainland China | 2011 | 2013 |
| 44 | 李旋, 严国进, 杨昌林,刘东林, | 2014 | 东台市2011-2013年恙虫病流行病学调查 | Chinese | Mainland China | Mainland China | 2011 | 2013 |
| 45 | 廖勇, 李如, 杨健平,黄仁发, | 2014 | 赣州市2008-2012年恙虫病流行病学分析 | Chinese | Mainland China | Mainland China | 2008 | 2012 |
| 46 | 罗雷, 李晓宁, 景钦隆,肖新才,李意兰,曹庆,魏跃红,杨智聪, | 2014 | 广州市2006-2012年重点媒介传染病流行特征分析 | Chinese | Mainland China | Mainland China | 2006 | 2012 |
| 47 | 余平, 程慧健, 魏雄杰, | 2014 | 江西省2006-2012年恙虫病流行特征分析 | Chinese | Mainland China | Mainland China | 2006 | 2012 |
| 48 | 申安然, 罗启松, 姚留昌,师敏,田宗茂, | 2014 | 陇川县2009-2013年恙虫病流行现状分析 | Chinese | Mainland China | Mainland China | 2009 | 2013 |
| 49 | 张萌, 赵仲堂 | 2014 | 秋冬型恙虫病疫区扩散的流行病学特征与机制研究 | Chinese | Mainland China | Mainland China | 2006 | 2013 |
| 50 | 陈华芳, 陈国伟, 柯雪梅,郭志南,林敏珍, | 2014 | 厦门市2009-2012年恙虫病流行情况分析 | Chinese | Mainland China | Mainland China | 2009 | 2012 |
| 51 | 刘传鸽, 冯梓来, 雷洁贞,崔楚平, | 2014 | 云浮市2006-2012年恙虫病流行病学分析 | Chinese | Mainland China | Mainland China | 2006 | 2012 |
| 52 | 赵安云 | 2014 | 云南省屏边县恙虫病流行特征分析 | Chinese | Mainland China | Mainland China | 2008 | 2012 |
| 53 | 方益荣, 张丽杰, 李群, | 2014 | 中国2009年恙虫病疫情分析 | Chinese | Mainland China | Mainland China | 2009 | 2009 |
| 54 | Peng, Pei-YingXu, LeiWang, Gu-XianHe, Wen-YuanYan, Ting-LiangGuo, Xian-Guo | 2015 | Epidemiological characteristics and spatiotemporal patterns of scrub typhus in Yunnan Province from 2006 to 2017 | Chinese | Mainland China | Mainland China | 2013 | 2013 |
| 55 | 任达飞, 胡建利, 谈忠鸣,张翔,何义林,尹俊,徐铮,鲍昌俊,移钱华,钱卫娟,王慎骄,吴斌,杨海玉,汤奋扬, | 2015 | 1起恙虫病暴发疫情的流行病学调查及病例经济负担分析 | English | Mainland China | Mainland China | 2006 | 2012 |
| 56 | Yang, Li-PingLiang, Si-YuanWang, Xian-JunLi, Xiu-JunWu, Yan-LingMa, Wei | 2015 | Burden of disease measured by disability-adjusted life years and a disease forecasting time series model of scrub typhus in Laiwu, China | English | Mainland China | Mainland China | 2006 | 2013 |
| 57 | Zheng, L.Yang, H. L.Bi, Z. W.Kou, Z. Q.Zhang, L. Y.Zhang, A. H.Yang, L.Zhao, Z. T. | 2015 | Epidemic characteristics and spatio-temporal patterns of scrub typhus during 2006-2013 in Tai'an, Northern China | English | Mainland China | Mainland China | 2006 | 2013 |
| 58 | De, WuJing, KouHuan, ZhangQiong, Zhou HuiMonagin, CorinaMin, Zhong JianPing, HuangWen, Ke ChangYan, Lin Jin | 2015 | Scrub typhus, a disease with increasing threat in Guangdong, China | Chinese | Mainland China | Mainland China | 2010 | 2013 |
| 59 | 韩腾伟, 刘菁, 洪荣涛,何似,陈亮,陈阳,邓艳琴, | 2015 | 福建省2010—2013年恙虫病流行特征分析 | Chinese | Mainland China | Mainland China | 2005 | 2013 |
| 60 | 张育富, 周明浩, 刘大鹏,杨维芳,刘慧,陈红娜,吴治明,褚宏亮, | 2015 | 江苏沿海地区2005—2013年虫媒传染病流行特征 | Chinese | Mainland China | Mainland China | 2006 | 2012 |
| 61 | 梁思园, 杨丽萍, 王显军,丁国永, | 2015 | 山东省2006—2012年恙虫病疾病负担分析 | Chinese | Mainland China | Mainland China | 2006 | 2013 |
| 62 | 王敬忠, 李怀昕, 谢旭,梅树江,马汉武, | 2015 | 深圳市2006～2013年恙虫病流行特征分析 | Chinese | Mainland China | Mainland China | 2008 | 2014 |
| 63 | 陈辉, 张丽杰, 唐雪峰,刘学成,曹一欧,周丽君,周兴余,袁伟,蒋德勇, | 2015 | 四川省2008—2014年恙虫病发病流行病学特征分析 | Chinese | Mainland China | Mainland China | 2006 | 2014 |
| 64 | 曹婧, 亚红祥, 袁庆虹,张云智, | 2016 | 云南省2006-2014年恙虫病发病率的聚类分析（英文） | Chinese | Mainland China | Mainland China | 2006 | 2014 |
| 65 | 李晓宁, 王大虎, 黄勇,马钰,罗雷,肖新才,杨智聪,魏跃红, | 2016 | 2006-2014年广州市儿童恙虫病流行特征分析 | Chinese | Mainland China | Mainland China | 2006 | 2014 |
| 66 | 高丽芬, 胡海梅, 胡秋凌,吴学林,罗琼梅,钱发宝, | 2016 | 2006-2014年云南省楚雄州恙虫病流行特征分析 | Chinese | Mainland China | Mainland China | 2011 | 2014 |
| 67 | 王祥旭, 宋传锋, 王安庆,张伟,张频, | 2016 | 2011-2014年日照市恙虫病流行病学分析 | English | Mainland China | Mainland China | 2006 | 2014 |
| 68 | Wu, Yi-ChengQian, QuanMagalhaes, Ricardo J. SoaresHan, Zhi-HaiHaque, UbydulWeppelmann, Thomas A.Hu, Wen-BiaoLiu, Yun-XiSun, Yan-SongZhang, Wen-YiLi, Shen-Long | 2016 | Rapid Increase in Scrub Typhus Incidence in Mainland China, 2006-2014 | Chinese | Mainland China | Mainland China | 2010 | 2014 |
| 69 | 李晓宁, 黄勇, 马钰,罗雷,肖新才,杨智聪,魏跃红, | 2016 | 广州市2010-2014年恙虫病流行特征及时空聚集性分析 | Chinese | Mainland China | Mainland China | 2005 | 2014 |
| 70 | 陈胤忠, 李峰, 徐慧,黄连成,顾振国,孙中友,严国进,朱叶江,汤池, | 2016 | 江苏省盐城市沿海滩涂2005-2014年恙虫病时空分布特征及影响因素分析 | Chinese | Mainland China | Mainland China | 2010 | 2013 |
| 71 | 颜玉炳, 郭志南, 陈小平, | 2016 | 厦门市2010—2013年恙虫病流行特征及时空分布分析 | Chinese | Mainland China | Mainland China | 2008 | 2014 |
| 72 | 颜玉炳, 郭志南, 陈小平, | 2016 | 厦门市恙虫病流行特征及发病趋势预测效果研究 | Chinese | Mainland China | Mainland China | 2006 | 2013 |
| 73 | 孙烨, 方立群, 曹务春, | 2016 | 山东、安徽、江苏省2006-2013年秋冬型恙虫病流行特征及影响因素研究 | Chinese | Mainland China | Mainland China | 2006 | 2014 |
| 74 | 杨慧, 毕振旺, 寇增强,郑丽,赵仲堂, | 2016 | 山东省2006-2014年秋冬型恙虫病空间流行病学分析 | Chinese | Mainland China | Mainland China | 2006 | 2014 |
| 75 | 金加洪, 曹恒志 | 2016 | 射阳县2006-2014年恙虫病流行趋势分析 | Chinese | Mainland China | Mainland China | 2006 | 2013 |
| 76 | 王敬忠, 陈凤媚, 李怀昕, | 2016 | 深圳市2006-2013年恙虫病时空分布特征 | Chinese | Mainland China | Mainland China | 2006 | 2014 |
| 77 | 吴义城, 张文义, 李申龙, | 2016 | 我国大陆地区恙虫病时空特征分析及风险预测研究 | Chinese | Mainland China | Mainland China | 1950 | 2015 |
| 78 | 孙烨, 方立群, 曹务春, | 2016 | 我国恙虫病地方性流行南北异质性比较研究 | Chinese | Mainland China | Mainland China | 2015 | 2015 |
| 79 | 谢冰艳, 张嘉陵, 孙传武,刁曼,刘小勇, | 2017 | 徐州市睢宁县恙虫病病例回顾性调查 | Chinese | Mainland China | Mainland China | 2006 | 2015 |
| 80 | 辛化雷, 李晓静, 徐士林,张永杰,祖荣强,裴迎新,孙健平, | 2017 | 2006-2015年江苏省盐城市恙虫病流行病学特征分析 | Chinese | Mainland China | Mainland China | 2006 | 2015 |
| 81 | 辛化雷, 张永杰, 胡建利,孙军玲,裴迎新,祖荣强, | 2017 | 2006-2015年江苏省恙虫病流行病学特征分析 | Chinese | Mainland China | Mainland China | 2008 | 2015 |
| 82 | 谢月梅, 周世卿, 程国婷, | 2017 | 2008-2015年广宁县恙虫病流行特征 | English | Mainland China | Mainland China | 2006 | 2014 |
| 83 | Wei, YuehongHuang, YongLi, XiaoningMa, YuTao, XiaWu, XinweiYang, Zhicong | 2017 | Climate variability, animal reservoir and transmission of scrub typhus in Southern China | Chinese | Mainland China | Mainland China | 2004 | 2015 |
| 84 | Wang, L.Wang, P.Wang, T.Zhang, L.Yang, S.Cui, F.Wang, Z.Zhai, W.Wang, X.Ding, S. | 2017 | Epidemiological characteristics of natural focus and vector borne infectious diseases in Zibo City Shandong Province between 2004 and 2015 | Chinese | Mainland China | Mainland China | 2006 | 2015 |
| 85 | 张萌, 杨新金, 张克春,叶新,谢润生,彭志强, | 2017 | 广东省2006―2015年恙虫病时空聚集性分析 | Chinese | Mainland China | Mainland China | 2006 | 2015 |
| 86 | 刘艳慧, 李乔玄, 景钦隆,李铁钢,任红艳, | 2017 | 广州市2006-2015年恙虫病疫情时空分布特征 | Chinese | Mainland China | Mainland China | 2011 | 2015 |
| 87 | 魏跃红, 王大虎, 鲁影,李晓宁,吴新伟,陶霞,陈守义,杨智聪, | 2017 | 广州市2011-2015年常见鼠传疾病流行病学分析 | Chinese | Mainland China | Mainland China | 2011 | 2015 |
| 88 | 展海燕, 徐贞, 何义林, | 2017 | 江苏省靖江市2011-2015年恙虫病流行特征及影响因素 | Chinese | Mainland China | Mainland China | 2013 | 2015 |
| 89 | 郭志南, 林勇, 陈国伟, | 2017 | 厦门市2013-2015年恙虫病流行特征和感染危险因素分析 | Chinese | Mainland China | Mainland China | 2006 | 2016 |
| 90 | 杨慧, 赵仲堂 | 2017 | 山东地区恙虫病流行特征及ELISA临床诊断价值研究 | Chinese | Mainland China | Mainland China | 2007 | 2016 |
| 91 | 王芳琼, 郭雄 | 2018 | 云南省楚雄市2007-2016年恙虫病流行特征分析 | Chinese | Mainland China | Mainland China | 2006 | 2013 |
| 92 | Sun, Y.Shi, C.Li, X. L.Fang, L. Q.Cao, W. C. | 2018 | [Epidemiology of scrub typhus and influencing factors in Yunnan province, 2006-2013] | Chinese | Mainland China | Mainland China | 2007 | 2016 |
| 93 | 凌远理, 陈晓燕 | 2018 | 2007-2016年广州市海珠区恙虫病流行特征分析 | Chinese | Mainland China | Mainland China | 2011 | 2015 |
| 94 | 上官致洋, 吴斐琳, 严秀英,章志红,郑月娥,朱小康, | 2018 | 2011-2015年抚州市恙虫病流行特征分析 | Chinese | Mainland China | Mainland China | 2013 | 2016 |
| 95 | 赖长剑, 王若琳, 张楠楠,李孟磊, | 2018 | 2013-2016年汝南县恙虫病流行病学特征分析 | Chinese | Mainland China | Mainland China | 2010 | 2017 |
| 96 | 李鹏宾, 邱宗耀, 柯志攀,曾鸿, | 2018 | 广东省佛山市南海区2010-2017年恙虫病流行特征分析 | Chinese | Mainland China | Mainland China | 2008 | 2017 |
| 97 | 林建民, 段成明, 俞发辉, | 2018 | 浦城县2008—2017年恙虫病流行特征 | Chinese | Mainland China | Mainland China | 1954 | 2016 |
| 98 | 郭志南, 陈敏, 陈华芳,陈国伟, | 2018 | 厦门市恙虫病流行病学特征与防控策略 | Chinese | Mainland China | Mainland China | 2010 | 2016 |
| 99 | 陈海龙, 吴瑞, 李江博,薛泽润,王丽荣,马超锋,李恒新, | 2018 | 西安市2010年至2016年恙虫病流行特征分析 | Chinese | Mainland China | Mainland China | 2006 | 2017 |
| 100 | 袁庆虹, 陈敏, 杨向东, | 2019 | 云南省2006-2017年恙虫病流行病学分析 | Chinese | Mainland China | Mainland China | 2006 | 2017 |
| 101 | 高丽芬, 吴学林, 胡海梅,钱发宝,罗琼梅,袁嘉忆,阎勇, | 2019 | 2006-2017年楚雄州3种鼠传疾病流行分析 | Chinese | Mainland China | Mainland China | 2006 | 2017 |
| 102 | 廖勇, 黄仁发, 胡晓军,郭俊,黄鸿山,李建华,刘晓青,徐建民, | 2019 | 2006-2017年江西省恙虫病流行病学分析 | English | Mainland China | Mainland China | 2009 | 2017 |
| 103 | Chen, RuiKou, ZengqiangXu, LiuchenCao, JieLiu, ZiweiWen, XiaojingWang, ZhiyuWen, Hongling | 2019 | Analysis of epidemiological characteristics of four natural-focal diseases in Shandong Province, China in 2009-2017: A descriptive analysis | English | Mainland China | Mainland China | 1957/2006 | 1989/2017 |
| 104 | Ren, JiangpingSun, JiminWang, ZhengtingLing, FengShi, XuguangZhang, RongLiu, YingChen, ZhipingChen, Enfu | 2019 | Re-emergence of scrub typhus in Zhejiang Province, southern China: A 45-year population-based surveillance study | English | Mainland China | Mainland China | 2006 | 2017 |
| 105 | Yue, YujuanRen, DongshengLiu, XiaoboWang, YujiaoLiu, QiyongLi, Guichang | 2019 | Spatio-temporal patterns of scrub typhus in mainland China, 2006-2017 | English | Mainland China | Mainland China | 1980/2006 | 1989/2013 |
| 106 | Yao, HongwuWang, YixingMi, XianmiaoSun, YeLiu, KunLi, XinlouRen, XiangGeng, MengjieYang, YangWang, LipingLiu, WeiFang, Liqun | 2019 | The scrub typhus in mainland China: spatiotemporal expansion and risk prediction underpinned by complex factors | Chinese | Mainland China | Mainland China | 2008 | 2017 |
| 107 | 朱梦, 姚慧媛, 宋丹丹,马婉婉,吴家兵,龚磊, | 2019 | 安徽省2008-2017年恙虫病流行特征与重复感染分析 | Chinese | Mainland China | Mainland China | 2008 | 2018 |
| 108 | 王玉姣, 闫冬明, 李贵昌,许磊,岳玉娟,王君,鲁亮,孙琬琬,周若冰,母群征,刘起勇,李兴洲, | 2019 | 北京市平谷区2008-2018年恙虫病流行特征分析 | Chinese | Mainland China | Mainland China | 2015 | 2016 |
| 109 | 李大鹏, 魏跃红, 杨智聪, | 2019 | 广州市恙虫病危险因素与Ot-Sta56基因分型研究 | Chinese | Mainland China | Mainland China | 2008 | 2017 |
| 110 | 张育富, 刘大鹏, 褚宏亮, | 2019 | 江苏省2008-2017年恙虫病流行特征和时空聚集性分析 | Chinese | Mainland China | Mainland China | 2006 | 2016 |
| 111 | 余向华, 倪朝荣, 潘琼娇, | 2019 | 温州市2006—2016年恙虫病流行特征分析 | Chinese | Mainland China | Mainland China | 2010 | 2016 |
| 112 | 何恺, 黄东升, 安丽,张腾,赵丽娟,李庆芳, | 2019 | 云南省保山市2006-2016年恙虫病流行特征分析 | Chinese | Mainland China | Mainland China | 2008 | 2015 |
| 113 | 刘晓宁, 柳燕 | 2020 | 安徽省阜阳市秋冬型恙虫病病原体基因型及流行危险因素研究 | Chinese | Mainland China | Mainland China | 2009 | 2018 |
| 114 | 李健艺 | 2020 | 2009-2018年肇庆市恙虫病流行特征分析 | Chinese | Mainland China | Mainland China | 2010 | 2018 |
| 115 | 夏仪, 张爱玲, 张建花,蔡恩茂,陈立英, | 2020 | 2010-2018年云南省红河县恙虫病流行特征分析 | English | Mainland China | Mainland China | 1952 | 2016 |
| 116 | Li, ZhongjieXin, HualeiSun, JunlingLai, ShengjieZeng, LingjiaZheng, CanjunRay, Sarah E.Weaver, Nicole DavisWang, LipingYu, JianxingFeng, ZijianHay, Simon I.Gao, George F. | 2020 | Epidemiologic Changes of Scrub Typhus in China, 1952-2016 | English | Mainland China | Mainland China | 2006 | 2018 |
| 117 | Luo, LiGuo, ZhinanLei, ZhaoHu, QingqingChen, MinChen, FanghuaZhao, ZeyuRui, JiaLiu, XingchunZhu, YuanzhaoWang, YaoYang, MengChen, Tianmu | 2020 | Epidemiology of tsutsugamushi disease and its relationship with meteorological factors in Xiamen city, China | Chinese | Mainland China | Mainland China | 2009 | 2018 |
| 118 | 汪君君姚永帅马涛徐庆许阳婷郑颖孙红敏丰罗菊吴小清张敏 | 2020 | 2009—2018年南京市主要虫媒传染病流行病学特征分析 | English | Mainland China | Mainland China | 2006 | 2018 |
| 119 | Xin, HualeiFu, PengSun, JunlingLai, ShengjieHu, WenbiaoClements, Archie C. A.Sun, JianpingCui, JingHay, Simon I.Li, XiaojingLi, Zhongjie | 2020 | Risk mapping of scrub typhus infections in Qingdao city, China | English | Mainland China | Mainland China | 2006 | 2017 |
| 120 | Xin, HualeiSun, JunlingYu, JianxingHuang, JileiChen, QiulanWang, LipingLai, ShengjieClements, Archie C. A.Hu, WenbiaoLi, Zhongjie | 2020 | Spatiotemporal and demographic characteristics of scrub typhus in Southwest China, 2006-2017: An analysis of population-based surveillance data | Chinese | Mainland China | Mainland China | 2008 | 2018 |
| 121 | 王玉姣, 李贵昌, 刘起勇,李兴洲, | 2020 | 北京市平谷区恙虫病自然疫源地研究 | Chinese | Mainland China | Mainland China | 2006 | 2017 |
| 122 | 彭佳, 廖羽, 张萌,邓爱萍,张应涛,彭志强, | 2020 | 广东省2006—2017年恙虫病流行特征分析 | Chinese | Mainland China | Mainland China | 2009 | 2018 |
| 123 | 余兰芳, 李素芳, 唐荣德,黄德仁,梁锦添,李春明,吴永灵, | 2020 | 江门市新会区崖门镇近11年恙虫病流行趋势与特征 | Chinese | Mainland China | Mainland China | 2006 | 2018 |
| 124 | 胡挺松, 李应, 胡秋凌,吴学林,高丽芬,李国伟,王志勇,胡海梅,王意银,张海林,张富强, | 2021 | 云南省楚雄州恙虫病流行特征及其病原体东方体基因分型研究 | Chinese | Mainland China | Mainland China | 2006 | 2020 |
| 125 | 陈祖华, 李琴, 胥磊,闫殊利,白永华,刘艺,张耀, | 2021 | 2006—2020年攀枝花市恙虫病流行特征分析 | Chinese | Mainland China | Mainland China | 2009 | 2019 |
| 126 | 吕燕宁, 别楚皞, 窦相峰,陈丽娟,李夫,孙玉兰,李仁清,王全意, | 2021 | 2009-2019年北京市恙虫病流行特征分析 | Chinese | Mainland China | Mainland China | 2011 | 2018 |
| 127 | 闫佳, 黄琳, 潘婷婷,刘建, | 2021 | 2011-2018年驻马店市恙虫病流行特征分析 | Chinese | Mainland China | Mainland China | 2015 | 2019 |
| 128 | 马蒙蒙, 王大虎, 鲁影,景钦隆, | 2021 | 广州市2015—2019年恙虫病流行特征分析 | Chinese | Mainland China | Mainland China | 2013 | 2019 |
| 129 | 李孟磊, 王文华, 李懿,尤爱国,陈伟, | 2021 | 河南省2013—2019年恙虫病流行病学特征分析 | Chinese | Mainland China | Mainland China | 2007 | 2020 |
| 130 | 王加志, 刘安恒, 杨洁,杨开芬, | 2022 | 腾冲市2007—2020年恙虫病流行特征 | Chinese | Mainland China | Mainland China | 2015 | 2018 |
| 131 | 莫嘉延, 欧阳茜, 黄庆超,黄城, | 2014 | 2015-2018年佛山市高明区恙虫病发病特点分析 | English | Mainland China | Mainland China | 2006 | 2017 |
| 132 | Bang, Hyeong-AeLee, Myeong-JinLee, Won-Chang | 2008 | Comparative research on epidemiological aspects of tsutsugamushi disease (scrub typhus) between Korea and Japan | English | South Korea | South Korea | 2001 | 2005 |
| 133 | Kim, Si Heon,Jang, Jae Yeon | 2010 | [Correlations between climate change-related infectious diseases and meteorological factors in Korea] | Korean | South Korea | South Korea | 2001 | 2008 |
| 134 | Noh, MaengseokLee, YoungjoChu, ChaeshinGwack, JinYoun, Seung-KiHuh, Sun | 2013 | Are there spatial and temporal correlations in the incidence distribution of scrub typhus in Korea? | English | South Korea | South Korea | 2001 | 2011 |
| 135 | Park, Ji-HyukKim, Sun-JaYoun, Seung-KiPark, KisooGwack, Jin | 2014 | Epidemiology of scrub typhus and the eschars patterns in South Korea from 2008 to 2012 | English | South Korea | South Korea | 2008 | 2012 |
| 136 | Lee, Hyeong-WooCho, Pyo YunMoon, Sung-UngNa, Byoung-KukKang, Yoon-JoongSohn, YoungjooYoun, Seung-KiHong, YeongseonKim, Tong-Soo | 2015 | Current situation of scrub typhus in South Korea from 2001-2013 | English | South Korea | South Korea | 2001 | 2013 |
| 137 | Lee, Sung Uk | 2017 | Epidemiologic characteristics of scrub typhus on Jeju Island | English | South Korea | South Korea | 2011.01 | 2016.12 |
| 138 | Olson, J. G.Bourgeois, A. L. | 1977 | Rickettsia tsutsugamushi infection and scrub typhus incidence among Chinese military personnel in the Pescadores Islands | English | Taiwan | Taiwan | 1976 | 1976 |
| 139 | Lee, Yeong-ShengWang, Pei-HuaTseng, Shu-JenKo, Ching-FenTeng, Hwa-Jen | 2006 | Epidemiology of scrub typhus in eastern Taiwan, 2000-2004 | English | Mainland China | Taiwan | 2000 | 2004 |
| 140 | Kuo, Chi-ChienHuang, Jing-LunKo, Chia-YingLee, Pei-FenWang, Hsi-Chieh | 2011 | Spatial analysis of scrub typhus infection and its association with environmental and socioeconomic factors in Taiwan | English | Taiwan | Taiwan | 2003 | 2008 |
| 141 | Wardrop, Nicola A.Kuo, Chi-ChienWang, Hsi-ChiehClements, Archie C. A.Lee, Pei-FenAtkinson, Peter M. | 2013 | Bayesian spatial modelling and the significance of agricultural land use to scrub typhus infection in Taiwan | English | UK | Taiwan | 2003 | 2011 |
| 142 | Tsai, Pui-Jen | 2013 | Scrub typhus and comparisons of four main ethnic communities in taiwan in 2004 versus 2008 using geographically weighted regression | English | Taiwan | Taiwan | 2000 | 2010 |
| 143 | Lin, F. H.Chou, Y. C.Chien, W. C.Chung, C. H.Hsieh, C. J.Yu, C. P. | 2021 | Epidemiology and risk factors for notifiable scrub typhus in taiwan during the period 2010–2019 | English | Taiwan | Taiwan | 1996 | 2014 |
| 144 | Chang, Yi-ChinSun, WuLin, Jiun-NongChen, Yen-HsuLai, Chung-HsuLee, Chen-Hsiang | 2021 | Epidemiology and risk factors of scrub typhus in Taiwan: A nationwide database study from 1996 to 2014 | English | Taiwan | Taiwan | 2016 | 2020 |
| 145 | Lin, En-ChengTu, Hung-PinHong, Chien-Hui | 2022 | Halved Incidence of Scrub Typhus after Travel Restrictions to Confine a Surge of COVID-19 in Taiwan | English | Taiwan | Taiwan | 2006 | 2016 |
| 146 | Chen, Hsiang-FeiPeng, Shih-HuanTsai, Kun-HsienYang, Cheng-FenChang, Mei-ChunHsueh, Yeou-LinSu, Chien-LingWang, Ruo-YuShu, Pei-YunYang, Su-Lin | 2019 | Molecular epidemiology of scrub typhus in Taiwan during 2006-2016 | English | Taiwan | Taiwan | 2010 | 2019 |
| 147 | Wangrangsimakul, TriElliott, IvoNedsuwan, SupalertKumlert, RawadeeHinjoy, SoawapakChaisiri, KittipongDay, Nicholas P. J.Morand, Serge | 2020 | The estimated burden of scrub typhus in Thailand from national surveillance data (2003-2018) | English | Thailand | Thailand | 2003 | 2018 |
| 148 | Elliott, I | 2018 | The Clinical Epidemiology of Scrub Typhus in Humans, Chiggers and Rodents | English | UK | Thailand | 2003 | 2017 |
| 149 | Watthanaworawit, Wanitda | 2021 | Evaluations of Diagnostic Tests for Undifferentiated Febrile Illness on the Thailand-Myanmar (Burma) Border | English | UK | Thailand | 2003 | 2014 |
| 150 | Mungmungpuntipantip, RujittikaWiwanitkit, Viroj | 2003 | Correlation between rainfall and the prevalence of scrub typhus: an observation from a tropical endemic country | English | Tailand | Thailand | 2019 | 2019 |

# Appendix Table S7: Case reports and case series of scrub typhus since 2000.

| **Country/region** | **Case reports** | | **Case series** | |
| --- | --- | --- | --- | --- |
|  | **No. paper** | **No. cases** | **No. paper** | **No. cases** |
| **Total** | **120** | **280** | **29** | **160** |
| Australia | 3 | 4 | 2 | 21 |
| Bhutan | 3 | 16 | **-** | **-** |
| Chile | 1 | 1 | 1 | 3 |
| India | 102 | 241 | 18 | 99 |
| Kuwait | 1 | 1 | **-** | **-** |
| Laos | **-** | **--** | 2 | 4 |
| Malaysia | 1 | 1 | 1 | 3 |
| Maldives | 1 | 1 | - | - |
| Myanmar | **-** | **-** | 1 | 1 |
| Nepal | 5 | 5 | 3 | 27 |
| Sri Lanka | 3 | 10 | **-** | **-** |
| Vietnam | **-** | **-** | 1 | 2 |

# Appendix Table S8: Characteristics of included case reports and case series, in order of country/region and published year (n=149).

| **ID** | **Author** | **Year of publication** | **Title** | **Language** | **Study_type** | **Publication country** | **Study conducted country** |
| --- | --- | --- | --- | --- | --- | --- | --- |
| 1 | Faa, Antony G., William J. H. McBride, Gaynor Garstone, Robert E. Thompson and Peter Holt | 2003 | Scrub typhus in the Torres Strait islands of north Queensland, Australia | English | case series | Australia | Australia |
| 2 | Ralph, Anna, Mark Raines, Peter Whelan and Bart J. Currie | 2004 | Scrub typhus in the Northern Territory: exceeding the boundaries of Litchfield National Park | English | case report | Australia | Australia |
| 3 | Unsworth, Nathan B., John Stenos, Antony G. Faa and Stephen R. Graves | 2007 | Three rickettsioses, Darnley Island, Australia | English | case report | Australia | Australia |
| 4 | Izzard, Leonard, Andrew Fuller, Stuart D. Blacksell, Daniel H. Paris, Allen L. Richards, Nuntipa Aukkanit, Chelsea Nguyen, Ju Jiang, Stan Fenwick, Nicholas P. J. Day, Stephen Graves and John Stenos | 2010 | Isolation of a novel Orientia species (O. chuto sp. nov.) from a patient infected in Dubai | English | case report | Australia | Australia |
| 5 | Stewart, Alexandra G. A., Simon Smith, Enzo Binotto and Josh Hanson | 2020 | Clinical Features of Rickettsial Infection in Children in Tropical Australia-A Report of 15 Cases | English | case series | Australia | Australia |
| 6 | Tshokey, T., S. Graves, D. Tshering, K. Phuntsho, K. Tshering and J. Stenos | 2017 | Scrub typhus outbreak in a remote primary school, Bhutan, 2014 | English | case report | Bhutan | Bhutan |
| 7 | Tshering, Sangay, Namkha Dorji, Dago Dem and Tandin Om | 2021 | Scrub typhus in pregnancy presenting with permanent hearing loss: A case report | English | case report | Bhutan | Bhutan |
| 8 | Jatsho, Jimba | 2022 | An unusual presentation of scrub typhus in a child: a case report | English | case report | Bhutan | Bhutan |
| 9 | Balcells, M. Elvira, Ricardo Rabagliati, Patricia Garcia, Helena Poggi, David Oddo, Marcela Concha, Katia Abarca, Ju Jiang, Daryl J. Kelly, Allen L. Richards and Paul A. Fuerst | 2011 | Endemic scrub typhus-like illness, Chile | English | case report | USA | Chile |
| 10 | Weitzel, Thomas, Sabine Dittrich, Javier Lopez, Weerawat Phuklia, Constanza Martinez-Valdebenito, Katia Velasquez, Stuart D. Blacksell, Daniel H. Paris and Katia Abarca | 2016 | Endemic Scrub Typhus in South America | English | case series | Chile | Chile |
| 11 | Cracco, C., C. Delafosse, L. Baril, Y. Lefort, C. Morelot, J. P. Derenne, F. Bricaire and T. Similowski | 2000 | Multiple organ failure complicating probable scrub typhus | English | case report | France | Imported from South East Asia |
| 12 | Vliegenthart-Jongbloed, Klaske, Mariana de Mendonca Melo, Lennert Slobbe, Matthias F. C. Beersma and Perry J. J. van Genderen | 2013 | Imported scrub typhus in The Netherlands | English | case report | Netherlands | Imported from South East Asia |
| 13 | Rajagopal, R., C. Khati, V. Vasdev and A. Trehan | 2003 | Scrub typhus: a case report | English | case report | India | India |
| 14 | Mathai, E., J. M. Rolain, L. Verghese, M. Mathai, P. Jasper, G. Verghese and D. Raoult | 2003 | Case reports: scrub typhus during pregnancy in India | English | case series | India | India |
| 15 | Mahajan, S. K. and D. Bakshi | 2007 | Acute reversible hearing loss in scrub typhus | English | case report | India | India |
| 16 | Mahajan, A., D. S. Jasrotia, R. S. Charak, T. Kumar, P. L. Bhagat, N. Sharma and B. B. Gupta | 2010 | Scrub typhus: Jammu outbreak-2009 | English | case report | India | India |
| 17 | Ahmad, Sohaib, Saurabh Srivastava, Sanjiv Kumar Verma, Prashant Puri and Nadia Shirazi | 2010 | Scrub typhus in Uttarakhand, India: a common rickettsial disease in an uncommon geographical region | English | case report | India | India |
| 18 | Mahajan, Sanjay K., Sharath N. M. Babu, Dhruv Sharma, Digvijay Singh, Anil Kanga and Satinder S. Kaushal | 2011 | Scrub typhus presenting as acute abdomen | English | case report | India | India |
| 19 | Mahajan, Sanjay K., Sharath Babu, Digvijay Singh, Anil Kanga and Satinder S. Kaushal | 2012 | Scrub typhus and leptospirosis coScrub typhus and leptospirosis co-infection in Himalayan region-infection in Himalayan region | English | case report | India | India |
| 20 | Sharma, A., R. Raina, P. Dhiman, Adarsh, I. Madhabhavi and P. Panda | 2012 | Rare coinfection of scrub typhus and malaria in immunocompetent person | English | case report | India | India |
| 21 | Gupta, Naveen, Veena Mittal, B. Gurung and U. Sherpa | 2012 | Pediatric Scrub typhus in South Sikkim | English | case report | India | India |
| 22 | Iqbal, Nayyar, Stalin Viswanathan, Bhavith Remalayam, Vivekanandan Muthu and Tarun George | 2012 | Pancreatitis and MODS Due to Scrub Typhus and Dengue Co-Infection | English | case report | India | India |
| 23 | Subbanna, P. K. and S. Suri | 2012 | Multi-organ dysfunction in scrub typhus | English | case report | India | India |
| 24 | Saifudheen, K., K. G. Sajeeth Kumar, James Jose, V. Veena and V. Abdul Gafoor | 2012 | First case of scrub typhus with meningoencephalitis from Kerala: An emerging infectious threat | English | case report | India | India |
| 25 | Devarajan, T. V., Suma M. Victor, Shabeena Khan and V. Shrilekha | 2012 | A case of scrub typhus with haematuria | English | case report | India | India |
| 26 | Yadav, D., A. Chopra, A. K. Dutta, S. Kumar and V. Kumar | 2013 | Scrub typhus: An uncommon cause of pyrexia without focus | English | case report | India | India |
| 27 | Goswami, Dibyajyoti, Arlangki Hing, Ananta Das and Monaliza Lyngdoh | 2013 | Scrub typhus complicated by acute respiratory distress syndrome and acute liver failure: a case report from Northeast India | English | case report | India | India |
| 28 | Kumar, Bhatrahalli Ashok Praveen, Arinagnalli Subbanna Praveen Kumar and E. Sharvanan | 2013 | Rhabdomyolysis in scrub typhus: an unusual presentation | English | case report | India | India |
| 29 | Karanth, Suman S., Anurag Gupta and Mukhyaprana Prabhu | 2013 | Pure cerebellitis due to scrub typhus: a unique case report | English | case report | India | India |
| 30 | Mookkappan, Sudhagar, Aneesh Basheer, Saranya Chidambaram, Nagarajan Natarajan and Bhairappa Shrimanth | 2014 | Transient adrenal insufficiency and post-treatment bradycardia in scrub typhus - a case report | English | case report | India | India |
| 31 | Raj, Selvin Sundar, Adhiti Krishnamoorthy, Manjeera Jagannati and Kundavaram Pp Abhilash | 2014 | Splenic infarct due to scrub typhus | English | case report | India | India |
| 32 | Prakash, A., A. S. Acharya, N. Jain, D. Bhattacharya and M. Chhabra | 2014 | Scrub typhus-an emerging public health problem in Delhi! | English | case report | India | India |
| 33 | Saxena, Amrish, Benjamine Khiangte and Iadarilang Tiewsoh | 2014 | Scrub typhus complicated by acute respiratory distress syndrome and multiorgan failure; an unrecognized alarming entity in central India: a report of two cases | English | case report | India | India |
| 34 | Bhatt, Alok, Aravind A. Menon, Rama Bhat and Siddalingana Gouda Thaplar Gurusiddana | 2014 | Pancreatitis in scrub typhus | English | case report | India | India |
| 35 | Sethi, Sunil, Amber Prasad, Manisha Biswal, Vinay Kumar Hallur, Abhishek Mewara, Navneet Gupta, Shipra Galhotra, Gagandeep Singh and Kusum Sharma | 2014 | Outbreak of scrub typhus in North India: a re-emerging epidemic | English | case report | India | India |
| 36 | Manickam, Kumar, Satheeshkumar Sunderkumar, Sridharan Chinnaraj and Shobhana Sivathanu | 2014 | Massive consolidation: a rare manifestation of paediatric Scrub typhus | English | case report | India | India |
| 37 | G K, Poomalar and Rekha R | 2014 | A case series of scrub typhus in obstetrics | English | case series | India | India |
| 38 | Ahmed, Atif Shaikh Iqbal, Abhilash Paul Prabhakar Kundavaram, Sowmya Sathyendra and Ooriapadickal C. Abraham | 2014 | Acute pancreatitis due to scrub typhus | English | case report | India | India |
| 39 | Subroto, C., S. Chakravarthy, A. C. Pravin, B. T. Jyoti, C. Banerjee and P. Bera | 2015 | Typhus Fever--A Diagnostic Dilemma | English | case report | India | India |
| 40 | Sankuratri, Srinivas, Pavani Kalagara, Kartika Balaji Samala, Prabhakar Krishna Veledandi and Srinadh Babu Atiketi | 2015 | Scrub Typhus with Acute Respiratory Distress Syndrome (ARDS) and its Management in Intensive Care Unit: A Case Report | English | case report | India | India |
| 41 | Devarajan, V., P. S. Nambi, R. Gopalakrishnan and A. Murali | 2015 | Scrub typhus in hospitalized children: A missed entity? | English | case report | India | India |
| 42 | Chandramohan, Anandhi, Soma Venkatesh, Gunasekaran Dhandapany and Selvaraj Stephen | 2015 | Scrub Typhus Co-infection in an Adolescent Girl with Varicella | English | case report | India | India |
| 43 | Sood, Shikha, Sanjeev Sharma and Shweta Khanna | 2015 | Role of advanced MRI brain sequences in diagnosing neurological complications of scrub typhus | English | case report | India | India |
| 44 | Basheer, Aneesh, Somanath Padhi, Vinoth Boopathy, Saumyaranjan Mallick, Shashikala Nair, Renu G'Boy Varghese and Reba Kanungo | 2015 | Hemophagocytic Lymphohistiocytosis: an Unusual Complication of Orientia tsutsugamushi Disease (Scrub Typhus) | English | case report | Italy | India |
| 45 | Koti, Neeraja, Aswani S Mareddy, Shivashankara K Nagri and Chandrashekar U Kudru | 2015 | Dancing eyes and dancing feet in scrub typhus | English | case report | India | India |
| 46 | Eswaradass, P. V. and C. Eswaradass | 2015 | Clinical, laboratory findings and complications of scrub typhus-meningoencephalitis: Case series | English | case report | India | India |
| 47 | Mittal, M., G. Mittal, G. Goel and Y. Singh | 2015 | Bilateral symmetrical brachial plexopathy in association with scrub typhus: A rare presentation | English | case report | India | India |
| 48 | Banda, Giridhar Reddy, Srikrishna Raghavendra Boddu, Priyanka Ballal and Jayaprakash Belle | 2016 | An Unusual Presentation of Scrub Typhus | English | case report | India | India |
| 49 | Pai, R., R. Chaudhry, N. Gupta, P. B. Sryma, A. Biswas and A. B. Dey | 2016 | Tricky typhus ticks two: A report of two sisters from North India presenting with acute respiratory distress syndrome due to scrub typhus | English | case report | India | India |
| 50 | Ete, Tony, Jaya Mishra, Bhupen Barman, Sumantro Mondal and Rondeep Kumar Nath Sivam | 2016 | Scrub Typhus Presenting with Bilateral Lateral Rectus Palsy in A Female | English | case report | India | India |
| 51 | Mahajan, Sanjay K., Sudhir Sharma, Madan Kaushik, Rajiv Raina, Preyander Thakur, Gagan Preet Taneja and Jai Bharat Sharma | 2016 | Scrub Typhus Presenting as Acute Cerebellitis | English | case report | India | India |
| 52 | Chaturvedi, Amit, Monica Gupta, Shweta Bhardwaj and Dipti Handa | 2016 | Scrub typhus masquerading as acute pancreatitis | English | case report | India | India |
| 53 | Ray, Animesh, Vivek Nangia, R. S. Chatterji and Navin Dalal | 2016 | Scrub typhus infection presenting as acute heart failure: A case report and systematic review of literature of cardiopulmonary involvement in scrub typhus infection | English | case report | India | India |
| 54 | Meena, Monika, Minakshi Rohilla, Vanita Jain, Jaswinder Kalra and Grv Prasad | 2016 | Scrub typhus in pregnancy: a case series | English | case series | India | India |
| 55 | A, Jeyasuriya, Badrinath Ak and Raghunathan R | 2016 | A case of multiorgan dysfunction due to scrub typhus infection | English | case report | India | India |
| 56 | Biswal, M., A. Jafra, N. Sahni, M. Sharma, P. Sikka and V. Suri | 2017 | Unusual presentation of scrub typhus in a post-partum patient: A diagnostic dilemma | English | case report | India | India |
| 57 | Seow, Christopher Wen-Xing, Veena Logarajah and Natalie Woon Hui Tan | 2017 | Typhoid and Scrub Typhus Coinfection in a Returned Traveler | English | case report | Singapore | India |
| 58 | Rohini, K., J. H. Reddy, L. Reddenna, H. Reddy and P. Venkatesh | 2017 | Scrub typhus: A case report | English | case report | India | India |
| 59 | Dhanapriya, J., T. Dineshkumar, R. Sakthirajan, S. Murugan, V. Jayaprakash, T. Balasubramaniyan and N. Gopalakrishnan | 2017 | Scrub typhus meningitis in a renal transplant recipient | English | case report | India | India |
| 60 | Sarma, Nilendu and Sayantani Chakraborty | 2017 | Scrub Typhus in Southern Districts of West Bengal | English | case series | India | India |
| 61 | Jajoo, Mamta, Dipti Kumar and Sameeksha Manchanda | 2017 | Scrub Typhus in a New Born | English | case report | India | India |
| 62 | Kumar, P., R. Charaniya, A. Ghosh, R. Sahoo, S. Murthy and S. Mittal | 2017 | Scrub typhus during dengue outbreak in Delhi: A case series and review of literature | English | case series | India | India |
| 63 | Vajpayee, Shailja, R. K. Gupta and M. L. Gupta | 2017 | Scrub typhus causing neonatal hepatitis with acute liver failure-A case series | English | case series | India | India |
| 64 | Koraluru, Munegowda, Manideep Nandigam, Indira Bairy, Sudha Vidyasagar and Muralidhar Varma | 2017 | Multiple eschars in scrub typhus: a case report | English | case report | India | India |
| 65 | Sv, Padmavathi Devi, Aruna M, Anil Cv Kumar, Hari Krishna Reddy, Sangeetha Bl and V. Siva Kumar | 2017 | Acute pancreatitis associated with scrub typhus | English | case report | India | India |
| 66 | Garg, A., A. Jain and R. Kashyap | 2018 | Travel-acquired scrub typhus infection masked by dengue fever in a patient from nonendemic area | English | case report | India | India |
| 67 | Saha, Bibhuti, Soumyadip Chatterji, Kaushik Mitra, Shibendu Ghosh, Arindam Naskar, Manab Kumar Ghosh, Suprio Parui, Ayusmati Thakur, Boudhayan Bhattacharya, Debajyoti Majumdar and Sayan Chakraborty | 2018 | Socio-demographic and Clinico-Epidemiological Study of Scrub Typhus in Two Tertiary Care Hospitals of Kolkata | English | case series | India | India |
| 68 | Sarkar, M., S. Banerjee and S. Roychowdhuri | 2018 | Scrub Typhus with Varied Clinical Presentations | English | case series | India | India |
| 69 | Zainab, M., A. K. Gupta and S. Guha | 2018 | Scrub typhus in children | English | case series | India | India |
| 70 | Shveta, K. Agarwal, B. Rishi and Khushboo | 2018 | Scrub typhus complicated by acute respiratory distress syndrome and haemophagocytic lymphohistiocytosis in a young patient | English | case report | India | India |
| 71 | Agrawal, A., S. Gupta, B. Aggarwal and S. Rana | 2018 | Scrub typhus coinfection with acute hepatitis a in a child with acute liver failure | English | case report | India | India |
| 72 | Roy, S., S. Chakrabartty, S. D. Majumdar, S. Chakravarti and S. Barman | 2018 | Multiple Eschars on Lip in Scrub Typhus | English | case report | India | India |
| 73 | Avasthi, G. L., P. Patel, R. Kuka and R. Mahajan | 2018 | Acute fulminant myocarditis as a rare manifestation in complicated scrub typhus: A case report | English | case report | India | India |
| 74 | Sethuraman, Vinoth Kumar and Kavitha Balasubramanian | 2019 | An Unusual Clinical Presentation of Scrub Typhus | English | case report | India | India |
| 75 | Singh, K. R., C. G. Singh, S. Khumukcham, N. D. Marak, P. Kumar, H. Parasmani and G. A. Kharshiing | 2019 | Scrub typhus: An unusual cause of acute abdomen | English | case series | India | India |
| 76 | Mohanty, Aroop, Ankita Kabi, Pratima Gupta, Mithilesh Kumar Jha, U. Sasi Rekha and Anusha Krishna Raj | 2019 | Scrub typhus - A case series from the state of Sikkim, India | English | case series | India | India |
| 77 | Chaudhry, Rama, Chandan Kumar Thakur, Nitin Gupta, Tanu Sagar, Tej Bahadur, Naveet Wig, Rita Sood and Mahesh Chandra Misra | 2019 | Mortality due to scrub typhus - report of five cases | English | case series | India | India |
| 78 | Bhargava, Anurag, Sathiq Ali, Rohit Rajeevan and Raghavendra Udyavara | 2019 | Marked elevation of serum lipase in scrub typhus without pancreatitis: a report of three cases | English | case report | India | India |
| 79 | Tandon, Ruchika, Amit Kumar and Ajay Kumar | 2019 | Long-Segment Myelitis, Meningoencephalitis, and Axonal Polyneuropathy in a Case of Scrub Typhus | English | case report | India | India |
| 80 | Mehta, Sudhir, Nidhi Sharma, Laxmi Kant Goyal, Sandhya Gulati, Tara Chand, Om Prakash Nag and Sahdev Patel | 2019 | Hemophagocytic Lymphohistiocytosis in a Patient of Scrub Typhus | English | case report | India | India |
| 81 | Gomber, Apoorva, Vijay Kumar, Shashikant Singh, Manjari Kishore and Sadhna Marwah | 2019 | Hemophagocytic Lymphohistiocytosis (HLH) Associated With Scrub Typhus: Report Of 2 Cases | English | case report | India | India |
| 82 | Dev, Nishant, Rahul Kumar and Dilip Kumar | 2019 | Guillain-Barre syndrome: a rare complication of leptospirosis and scrub typhus co-infection | English | case report | India | India |
| 83 | Guha, S. and G. Guha | 2019 | Four cases of scrub typhus probably complicated by Kawasaki Disease | English | case series | India | India |
| 84 | Mehta, Vibha, Ankith Bhasi, Prasan Kumar Panda and Pratima Gupta | 2019 | A coinfection of severe leptospirosis and scrub typhus in Indian Himalayas | English | case report | India | India |
| 85 | Venketesan, Sivaranjini, Dheeraj Jain, Stalin Viswanathan and Murugesan Sivagurunathan Gayathri | 2019 | Case Report: Acute pyelonephritis and hearing loss in scrub typhus | English | case report | India | India |
| 86 | Roy, S. and S. Chakrabartty | 2019 | Acute Disseminated Encephalomyelitis as a Complication of Scrub Typhus in Children | English | case report | India | India |
| 87 | Tayade, A., S. Acharya, N. Balankhe, A. Ghule and S. Lahole | 2020 | Splenic infarction complicating scrub typhus | English | case report | India | India |
| 88 | Goyal, Manjeet K., Yogesh C. Porwal, Arun Gogna and Sameer Gulati | 2020 | Splenic infarct with scrub typhus: a rare presentation | English | case report | India | India |
| 89 | Annadatha, Akhilesh, Sourya Acharya, Amol Andhale and Sree Karthik Pratapa | 2020 | Splenic Abscess Complicating Scrub Typhus Induced Splenic Infarct - A Rare Case Report | English | case report | India | India |
| 90 | Maitra, S., U. K. Chakravarty and K. Ray | 2020 | Scrub typhus meningismus: A diagnostic dilemma | English | case report | India | India |
| 91 | Kotwal, Mudit, Esha Vaish, K. K. Gupta and Ahmad Ozair | 2020 | Scrub typhus manifesting with intracerebral hemorrhage: Case report and review of literature | English | case report | India | India |
| 92 | Pannu, Ashok Kumar, S. Smitha, Nithiyanandan Ravi, Yogesh Chander, Manisha Biswal, Vikas Suri and Savita Kumari | 2020 | Scrub typhus cholecystitis | English | case report | India | India |
| 93 | Sukumar, C. A., A. R. Poduval, N. Bhat and S. Vidyasagar | 2020 | Scrub and Spleen: Scrub Typhus with a Splenic Infarct | English | case report | India | India |
| 94 | Ghosh, Tarapada, Saba Annigeri, Arindam Ghosh, Koushik Mondal and Shiladitya Misra | 2020 | Pulmonary thromboembolism - A rare complication in a scrub typhus infection | English | case report | India | India |
| 95 | Thoyyib, Muhammed, Shamsudeen Moideen and N. A. Uvais | 2020 | Pulmonary parenchymal involvement: A rare manifestation of adult scrub typhus | English | case report | India | India |
| 96 | Hulkoti, V., A. Dubey, S. Kumar and S. Pratapa | 2020 | Psychotic scrub typhus: A Case Report | English | case report | India | India |
| 97 | Lamech, T. M., M. Chellaraj, R. Penchalaiah and D. Dhanasekaran | 2020 | Pseudothrombocytopenia in Patients with Scrub Typhus Infection | English | case report | India | India |
| 98 | Minhas, Ashish, Nishchint Sharma, Shalini Sharma and Versha Verma | 2020 | Post-CPR scrub typhus patient with ARDS: A case report | English | case report | India | India |
| 99 | Sardana, Vijay and Prashant Shringi | 2020 | Neurological Manifestations of Scrub Typhus: A Case Series from Tertiary Care Hospital in Southern East Rajasthan | English | case series | India | India |
| 100 | Atam, Virendra, Avirup Majumdar, D. Himanshu, Vivek Kumar and Isha Atam | 2020 | Massive splenomegaly in scrub typhus: A rare presentation | English | case report | India | India |
| 101 | Gaba, Saurabh, Swati Garg, Monica Gupta and Rekha Gupta | 2020 | Haemorrhagic encephalitis in the garb of scrub typhus | English | case report | India | India |
| 102 | Dave, M., S. Jain, H. Nath and Nagraj | 2020 | Digital gangrene: A rare complication in scrub typhus | English | case report | India | India |
| 103 | Singh, Vishal, Satish Chandra Mishra, Nikesh A. Agarwal, Binod B. Raut and Pulkit Singh | 2020 | Dengue with Scrub Typhus Coinfection in Northern India | English | case report | India | India |
| 104 | Kaur, Parminder, Reena Jain, Pankaj Kumar, Shivani Randev and Vishal Guglani | 2020 | Clinical Spectrum and Outcome of Acute Encephalitis Syndrome in Children with Scrub Typhus: A Series of Eight Cases from India | English | case report | India | India |
| 105 | Sharma, Sanjiv, Samhita Panda, Sarbesh Tiwari, Apoorv Patel and Vidhi Jain | 2020 | Chronic encephalopathy and locked-in state due to scrub typhus related CNS vasculitis | English | case report | India | India |
| 106 | Gupta, Samiksha, Sahil Grover, Monica Gupta and Daljinderjit Kaur | 2020 | Cerebellitis as a rare manifestation of scrub typhus fever | English | case report | India | India |
| 107 | Saxena, P., D. Chadha and R. Goyal | 2020 | A case series on neurological insights of scrub typhus | English | case series | India | India |
| 108 | Soundararajan, Sunitha, Stalin Viswanathan, Dheeraj Jain, Vijayalatchumy Krishnamurthy and Murugesan S. Gayathri | 2020 | Acute Parkinsonism and Cerebral Salt-wasting-related Hyponatremia in Scrub Typhus | English | case report | India | India |
| 109 | Mandal, T., U. Chakraborty and J. Pal | 2021 | Unusual neurological manifestations of scrub typhus | English | case report | India | India |
| 110 | Chandra, Atanu, Aritra Kumar Ray, Arkapravo Hati and Uddalak Chakraborty | 2021 | Tropical pyomyositis in scrub typhus: a rare association | English | case report | India | India |
| 111 | Singh, Ankur, Akansha Anjali, Rajniti Prasad, Pradyot Prakash and Om Prakash Mishra | 2021 | Scrub typhus: A rare cause of secondary nephrotic syndrome | English | case report | India | India |
| 112 | Agarwal, M., Aishwarya, M. Bairwa and Ravikant | 2021 | Scrub typhus with Guillain-Barre syndrome: An atypical sequela due to delayed diagnosis | English | case report | India | India |
| 113 | Das, Sayonee, Sidhartha Chattopadhyay, Kausik Munsi and Sagar Basu | 2021 | Scrub typhus with cerebral venous sinus thrombosis: a rare presentation | English | case report | India | India |
| 114 | Abbas, Syed F., Ahmad Ozair, Vivek Kumar and D. Himanshu | 2021 | Scrub Typhus Presenting with Hemiparesis: Case Report of a Rare Manifestation | English | case report | India | India |
| 115 | Srinithi, R., R. Ramya, A. P. Krithika and S. Sundari | 2021 | Scrub Typhus Presenting as Lobar Pneumonia: A Case Report on Uncommon Presentation of Scrub Typhus | English | case report | India | India |
| 116 | Kumar, A. P. G., S. S. Kanna and D. Babu | 2021 | Scrub Typhus Presenting as Acute Pancreatitis: A Rare Case | English | case report | India | India |
| 117 | Chauhan, Ajay, Aditya Jandial, Kundan Mishra and Rajeev Sandal | 2021 | Scrub typhus and lateral rectus palsy: an uncommon presentation of a common illness | English | case report | India | India |
| 118 | Pal Grewal, Tejinder and Mohan Kumar H | 2021 | Scrub eschar | English | case report | India | India |
| 119 | Philip, A. J., S. Jagdish, S. Nair and N. E. George | 2021 | A Rare Case of Scrub Typhus with Eschar and Abscess Formation Over Previous Surgical Scar Site-Scar Site Scrub Typhus | English | case report | India | India |
| 120 | Arasu, Meenupriya, Nagalakshmi Swaminathan, Anusha Cherian and Magesh Parthiban | 2021 | Prone ventilation in a pregnant patient with scrub typhus-induced acute respiratory distress syndrome | English | case report | India | India |
| 121 | Mohanty, C. R., S. Bellapukonda and Z. Shaikh | 2021 | Presence of bilateral vocal cord palsy with stridor: A case study on an unusual complication of scrub typhus | English | case report | India | India |
| 122 | Kumar, H. C. K., K. J. Kumar, V. G. Manjunath and S. Balaji | 2021 | Hemophagocytic lymphohistiocytosis associated with coinfection of scrub typhus and Dengue Fever in a child: A case report | English | case series | India | India |
| 123 | Marwah, A. and P. M. Marwah | 2021 | Atypical and severe manifestations of scrub typhus in children: Hiding without the rash | English | case series | India | India |
| 124 | Naik, Shailendra S., Mayurnath R. Bedadala, Manik Sharma and Himanshu Sethi | 2022 | Unusual Magnetic Resonance Imaging Features of Scrub Typhus Encephalitis | English | case report | India | India |
| 125 | Ghosh, Ritwik, Subhrajyoti Biswas, Arnab Mandal, Kaustav De, Srijit Bandyopadhyay, Sona Singh Sardar, Arpan Mandal and Julian Benito-Leon | 2022 | Scrub Typhus Presenting as Unilateral Abducens Nerve Palsy | English | case report | India | India |
| 126 | Gupta, Samiksha, Gautam Jesrani, Saurabh Gaba and Monica Gupta | 2022 | Scrub typhus manifesting as electrocardiographic disturbance: A case report and review of literature | English | case report | India | India |
| 127 | Thakur, Chandan Kumar, Priyam Batra, E. V. Vinayaraj, K. Sreenath, Nisha Rathor, Urvashi B. Singh, Ridhima Bhatia, Ajisha Aravindan, Naveet Wig, Randeep Guleria and Rama Chaudhry | 2022 | Scrub typhus in two COVID-19 patients: a diagnostic dilemma | English | case series | India | India |
| 128 | Singh, A. K., R. Sharma and G. Varadaraj | 2022 | Every lid swelling is not stye: A rare presentation of scrub typhus eschar | English | case report | India | India |
| 129 | Sen, Saswati, Bhagabat Nayak and Sucheta Parija | 2022 | Bilateral optic disc edema with subconjunctival hemorrhage: Attributed to scrub typhus? | English | case report | India | India |
| 130 | Dalal, Preeti and Neeraj Singla | 2022 | An Atypical Case of Complete Heart Block in Scrub Typhus | English | case report | India | India |
| 131 | Kumar, A., S. Tanwar, S. Gupta and R. Chetiwal | 2022 | Acute respiratory distress syndrome complicating scrub typhus in pregnancy | English | case report | India | India |
| 132 | Majumder, Srinanda, Moumita Samanta and Tapan Kumar SinhaMahapatra | 2022 | Acute demyelination of the medulla oblongata owing to scrub typhus in a 7-year-old boy: case report | English | case report | India | India |
| 133 | Nasr, M., M. M. A. Mostafa and S. K. Surrun | 2009 | Scrub typhus associated with systemic lupus erythematosus: A case reportted with systemic lupus erythematosus | English | case report | Kuwait | Kuwait |
| 134 | Eisermann, Philip, Jessica Rauch, Stefan Reuter, Lukas Eberwein, Ute Mehlhoop, Petra Allartz, Birgit Muntau and Dennis Tappe | 2020 | Complex Cytokine Responses in Imported Scrub Typhus Cases, Germany, 2010-2018 | English | case series | Germany | Laos |
| 135 | Costa, Cecilia, Anna Ferrari, Raffaella Binazzi, Anna Beltrame, Danilo Tacconi, Lucia Moro, Sophie Edouard, Philippe Parola, Dora Buonfrate and Federico Gobbi | 2021 | Imported scrub typhus in Europe: Report of three cases and a literature review | English | case series | Italy | Laos |
| 136 | Mohamed Zan, H. A., Ponnampalavanar Sasheela, S. F. S. Omar, Puthucheary D. Savithiri, Yvonne Al Lim and T. T. Sun | 2016 | Genetic variants of Orientia tsutsugamushi identified from scrub typhus cases in Malaysia | English | case series | Malaysia | Malaysia |
| 137 | Hafizuddin, A. and M. A. Md Hanif | 2020 | A paralyzing bite: An unorthodox case of scrub typhus in a non-aboriginal malaysian patient | English | case report | Malaysia | Malaysia |
| 138 | Imad, Hisham Ahmed, Aishath Azna Ali, Mariyam Nahuza, Rajan Gurung, Abdulla Ubaid, Aishath Maeesha, Sariu Ali Didi, Rajib Kumar Dey, Abdullah Isneen Hilmy, Aishath Hareera, Ibrahim Afzal, Wasin Matsee, Wang Nguitragool, Emi E. Nakayama and Tatsuo Shioda | 2021 | Acalculous Cholecystitis in a Young Adult with Scrub Typhus: A Case Report and Epidemiology of Scrub Typhus in the Maldives | English | case report | Thailand | Maldives |
| 139 | Sapkota, Surendra, Sudeep Bhandari, Subash Sapkota and Rabin Hamal | 2017 | Dengue and Scrub Typhus Coinfection in a Patient Presenting with Febrile Illness | English | case report | Nepal | Nepal |
| 140 | Acharya, Suman, Jayant Kumar Yadav, Nischal Khanal, Raju Bhandari and Bikal Ghimire | 2019 | Acute Severe Calculous Cholecystitis with Multiorgan Failure Complicated by Scrub Typhus | English | case report | Nepal | Nepal |
| 141 | Bhattarai, Tulsi, Sujan Chandra Poudel, Nishma Pokharel and Suraj Bhattarai | 2020 | Scrub typhus as a rare cause of acute pyelonephritis: case report | English | case report | Nepal | Nepal |
| 142 | Gautam, Jeevan, Randhir S. Yadav, Shumneva Shrestha, Bishnu Mohan Singh and Renusha Maharjan | 2021 | Scrub Typhus: Report of Three Cases From Rural Nepal and a Brief Literature Review | English | case series | USA | Nepal |
| 143 | Subedi, Prakriti, Manoj Ghimire, Karun Shrestha, Kalpana Ghimire, Sudeep Adhikari and Bishal Tiwari | 2021 | Dengue and scrub typhus co-infection causing septic shock | English | case report | Nepal | Nepal |
| 144 | Basukala, Sunil, Shriya Sharma, Ayush Tamang and Ujwal Bhusal | 2022 | A suspected surgical case of acute abdomen: A case report of scrub typhus | English | case report | Nepal | Nepal |
| 145 | Gurung, Shekhar, Saurab Karki, Subashchandra Pokharel and Kishor Bhatta | 2022 | Scrub typhus in a primary health care center of Nepal: A case series | English | case series | Nepal | Nepal |
| 146 | Kurup, Asok, Aneesh Issac, Jin Phang Loh, Too Bou Lee, Robert Chua, Pradeep Bist, Chien-Chung Chao, Michael Lewis, Duane J. Gubler, Wei Mei Ching, Eng Eong Ooi and Bindu Sukumaran | 2013 | Scrub typhus with sepsis and acute respiratory distress syndrome | English | case report | Singapore | Singapore |
| 147 | Premaratna, R., T. G. A. N. Chandrasena, A. S. Dassayake, A. D. Loftis, G. A. Dasch and H. J. de Silva | 2006 | Acute hearing loss due to scrub typhus: a forgotten complication of a reemerging disease | English | case report | USA | Sri Lanka |
| 148 | Premaratna, R., B. M. T. P. Nawasiwatte, M. A. Niriella, T. G. A. N. Chandrasena, N. K. B. K. R. G. W. Bandara, R. P. V. J. Rajapakse and H. J. de Silva | 2010 | Scrub typhus Scrub typhus mimicking enteric fever; a report of three patientsmimicking enteric fever; a report of three patients | English | case report | Sri Lanka | Sri Lanka |
| 149 | Premaratna, Ranjan, S. H. Nuwan Chamara Wijayalath, J. K. N. Dhanushka Miththinda, N. K. B. K. R. G. Wijesinghe Bandara and H. Janaka de Silva | 2015 | Scrub typhus mimicking Parkinson's disease | English | case report | Sri Lanka | Sri Lanka |

# Appendix Table S9: Characteristics of included mortality studies, in order of country/region and published year (n=129).

| **ID** | **Author** | **Year of publication** | **Title** | **Language** | **Publication country** | **Study conducted country** | **Start-time of investigation** | **End-time of investigation** |
| --- | --- | --- | --- | --- | --- | --- | --- | --- |
| 1 | Derrick, E. H., et.al.^227^ | 1961 | The incidence and distribution of scrub typhus in North Queensland | English | Australia | Australia | 1951 | 1955 |
| 2 | Tshokey, T., et.al.^7^ | 2019 | The epidemiology of rickettsial diseases, scrub typhus and q fever in Bhutan: A first report | English | Bhutan | Bhutan | 2014 | 2014 |
| 3 | Chheng, Kheng, et.al.^8^ | 2013 | A prospective study of the causes of febrile illness requiring hospitalization in children in Cambodia | English | Cambodia | Cambodia | 2009.10.12 | 2010.10.12 |
| 4 | Mackie, T. T., et.al.^228^ | 1946 | Observations on tsutsugamushi disease (scrub typhus) in Assam and Burma | English | India | India | 1944 | 1945 |
| 5 | Mathai, E., et.al.^229^ | 2003 | Outbreak of scrub typhus in southern India during the cooler months | English | India | India | 2001 | 2002 |
| 6 | Varghese, G. M., et.al.^14^ | 2006 | Scrub typhus among hospitalised patients with febrile illness in South India: magnitude and clinical predictors | English | India | India | 2002 | 2003 |
| 7 | Mahajan, Sanjay K., et.al.^230^ | 2008 | Pediatric scrub typhus in Indian Himalayas | English | India | India | nan | nan |
| 8 | Chrispal, Anugrah, et.al.^231^ | 2010 | Acute undifferentiated febrile illness in adult hospitalized patients: the disease spectrum and diagnostic predictors - an experience from a tertiary care hospital in South India | English | India | India | 2007.01 | 2008.01 |
| 9 | Singh, S. Ibungochouba, et.al.^232^ | 2010 | An outbreak of scrub typhus in Bishnupur district of Manipur, India, 2007 | English | India | India | 2007.05 | 2007 |
| 10 | Chrispal, Anugrah, et.al.^15^ | 2010 | Scrub typhus: an unrecognized threat in South India - clinical profile and predictors of mortality | English | India | India | 2007 | 2008 |
| 11 | Basu, Gopal, et.al.^16^ | 2011 | Acute kidney injury in tropical acute febrile illness in a tertiary care centre--RIFLE criteria validation | English | India | India | 2007.01 | 2008.01 |
| 12 | Rathi, Narendra B., et.al.^17^ | 2011 | Rickettsial diseases in central India: proposed clinical scoring system for early detection of spotted fever | English | India | India | 2009 | 2009 |
| 13 | NA | 2011 | Promed Post - ProMED-mail-2011-12-08 | English | India | India | nan | nan |
| 14 | Palanivel, Sengottaiya, et.al.^233^ | 2012 | Clinical profile of scrub typhus in children | English | India | India | 2010.10 | 2011.03 |
| 15 | Narvencar, Kedareshwar P. S., et.al.^19^ | 2012 | Scrub typhus in patients reporting with acute febrile illness at a tertiary health care institution in Goa | English | India | India | 2009 | 2010 |
| 16 | Abrahamsen, Siri Kratter, et.al.^20^ | 2013 | Fever in the tropics: aetiology and case-fatality - a prospective observational study in a tertiary care hospital in South India | English | Norway | India | 2007.07.02 | 2007.08.02 |
| 17 | Varghese, George M., et.al.^234^ | 2013 | Scrub typhus in South India: clinical and laboratory manifestations, genetic variability, and outcome | English | India | India | 2009 | 2010 |
| 18 | Singh, S. P., et.al.^235^ | 2014 | A study of complications of scrub typhus in a tertiary health care institute of Uttarakhand, India | English | India | India | 2012.12 | 2013.11 |
| 19 | Singh, Ragini, et.al.^24^ | 2014 | A Study of Etiological Pattern in an Epidemic of Acute Febrile Illness during Monsoon in a Tertiary Health Care Institute of Uttarakhand, India | English | Inda | India | 2013.07 | 2013.11 |
| 20 | Jamil, Md, et.al.^236^ | 2014 | Clinical Manifestations and Complications of Scrub Typhus: A Hospital Based Study from North Eastern India | English | India | India | 2014.11 | 2015.04 |
| 21 | Varghese, George M., et.al.^237^ | 2014 | Clinical profile and improving mortality trend of scrub typhus in South India | English | India | India | 2005 | 2010 |
| 22 | Subbalaxmi, M. V. S., et.al.^238^ | 2014 | Outbreak of scrub typhus in Andhra Pradesh--experience at a tertiary care hospital | English | India | India | 2011 | 2012 |
| 23 | Bithu, R., et.al.^21^ | 2014 | Possibility of scrub typhus in fever of unknown origin (FUO) cases: an experience from Rajasthan | English | India | India | 2012 | 2012 |
| 24 | Griffith, Mathew, et.al.^22^ | 2014 | Profile of organ dysfunction and predictors of mortality in severe scrub typhus infection requiring intensive care admission | English | India | India | 2008 | 2010 |
| 25 | Kumar Bhat, Nowneet, et.al.^239^ | 2014 | Scrub typhus in children at a tertiary hospital in north India: clinical profile and complications | English | India | India | 2011 | 2012 |
| 26 | Kumar, Vivek, et.al.^240^ | 2014 | Scrub typhus is an under-recognized cause of acute febrile illness with acute kidney injury in India | English | India | India | 2011 | 2012 |
| 27 | Bhat, N. K., et.al.^241^ | 2014 | Scrub typhus: A common rickettsial disease emerging in a new geographical region of north India | English | India | India | 2013 | 2013 |
| 28 | Oberoi, Aroma, et.al.^23^ | 2014 | Scrub typhus-an emerging entity: a study from a tertiary care hospital in North India | English | India | India | 2011 | 2012 |
| 29 | Luthra, M., et.al.^242^ | 2015 | A study of scrub typhus cases from a tertiary care hospital in Dehradun | English | Inda | India | 2014.08 | 2014.12 |
| 30 | Das, Dipmala, et.al.^26^ | 2015 | Common Infectious Etiologies of Acute Febrile Illness in a Remote Geographical Location: Could Scrub Typhus be the Most Common Cause? | English | USA | India | 2013.05 | 2013.10 |
| 31 | Jayanandan, Rajesh, et.al.^243^ | 2015 | Health care seeking behaviour and expenditure pattern among Scrub Typhus patients attending a tertiary care hospital in Mysore city | English | India | India | 2013.01 | 2013.12 |
| 32 | Abhilash, Kundavaram Paul Prabhakar et.al.^244^ | 2015 | Scrub typhus meningitis: An under-recognized cause of aseptic meningitis in India | English | India | India | 2005 | 2011 |
| 33 | Venkategowda, Pradeep M., et.al.^245^ | 2015 | Scrub typhus: Clinical spectrum and outcome | English | India | India | 2012 | 2013 |
| 34 | Abhilash, Kundavaram Paul Prabhakar, et.al.^31^ | 2016 | Acute Undifferentiated Febrile Illness in Patients Presenting to a Tertiary Care Hospital in South India: Clinical Spectrum and Outcome | English | India | India | 2012.10 | 2013.09 |
| 35 | Sivarajan, Sunuraj, et.al.^246^ | 2016 | Clinical and paraclinical profile, and predictors of outcome in 90 cases of scrub typhus, Meghalaya, India | English | India | India | 2011.09 | 2012.08 |
| 36 | Gurunathan, P. S., et.al.^247^ | 2016 | Clinical Profile, Morbidity Pattern and Outcome of Children with Scrub Typhus | English | India | India | 2012.03 | 2014.08 |
| 37 | Ramaganeshan, D., et.al.^248^ | 2016 | Investigatory Profile and Treatment Outcome of Paediatric Scrub Typhus Patients: A Tertiary Care Hospital Experience in Southern India | English | India | India | 2013.07 | 2015.12 |
| 38 | Mahajan, Sanjay K., et.al.^249^ | 2016 | Pattern of Clinical Presentation, Laboratory Findings and Mortality Risk Among Patients of Scrub Typhus in Western Himalayas | English | India | India | 2010 | 2011 |
| 39 | Krishnan, R., et.al.^250^ | 2016 | Pediatric scrub typhus in Southern Kerala: An emerging public health problem | English | India | India | 2011 | 2015 |
| 40 | Sharma, Navneet, et.al.^251^ | 2016 | Scrub Typhus in a Tertiary Care Hospital in North India | English | India | India | 2013 | 2014 |
| 41 | Rajan, S. J., et.al.^37^ | 2016 | Scrub typhus in pregnancy: Maternal and fetal outcomes | English | India | India | 2010 | 2012 |
| 42 | Bhargava, Anurag, et.al.^32^ | 2016 | Scrub typhus in Uttarakhand & adjoining Uttar Pradesh: Seasonality, clinical presentations & predictors of mortality | English | India | India | 2012 | 2013 |
| 43 | Bhat, Nowneet Kumar, et.al.^252^ | 2016 | Scrub Typhus: A Clinico-Laboratory Differentiation of Children with and without Meningitis | English | India | India | nan | nan |
| 44 | Rose, Winsley, et.al.^253^ | 2016 | Distribution of Eschar in Pediatric Scrub Typhus | English | India | India | 2010 | 2014 |
| 45 | Sarangi, R., et.al.^38^ | 2016 | Clinical profile of scrub typhus in children treated in a tertiary care hospital in eastern India | English | India | India | 2015 | 2015 |
| 46 | Kumar, Ritesh, et.al.^254^ | 2016 | Clinical Profile and Complications of Scrub Typhus: Hospital-Based Study in Sub-Himalayan Region | English | India | India | 2012 | 2013 |
| 47 | nan | 2017 | Promed Post - ProMED-mail-2017-12-31 | English | nan | India | 2017 | 2017 |
| 48 | Narvencar, Kedareshwar, et.al.^44^ | 2017 | Rickettsial Infections in Goa-Not Just Scrub Typhus! | English | India | India | nan | nan |
| 49 | Mittal, Mahima, et.al.^42^ | 2017 | Scrub Typhus as a Cause of Acute Encephalitis Syndrome, Gorakhpur, Uttar Pradesh, India | English | India | India | 2016 | 2016 |
| 50 | Takhar, Rajendra Prasad, et.al.^255^ | 2017 | Scrub typhus: A prospective, observational study during an outbreak in Rajasthan, India | English | India | India | 2014 | 2014 |
| 51 | Singhi, Sunit, et.al.^46^ | 2017 | Tropical Fevers in Indian Intensive Care Units: A Prospective Multicenter Study | English | India | India | 2013 | 2014 |
| 52 | Mina, S. S., et.al.^41^ | 2017 | Emerging Infections in Children in North India: Scrub Typhus | English | India | India | nan | nan |
| 53 | Negi, Ankita, et.al.^256^ | 2018 | A Study of Acute Febrile Illness with Thrombocytopenia from Tertiary Care Centre of Uttarakhand | English | India | India | 2015.09 | 2017.04 |
| 54 | Ganesh, Ramaswamy, et.al.^257^ | 2018 | Clinical profile and outcome of children with scrub typhus from Chennai, South India | English | India | India | 2012.01 | 2015.12 |
| 55 | Dhar, S. K., et.al.^258^ | 2018 | Clinical spectrum of scrub typhus in a tertiary care hospital at eastern India | English | India | India | 2015.04 | 2017.10 |
| 56 | Rauf, Abdul, et.al.^54^ | 2018 | Non-Respiratory and Non-Diarrheal Causes of Acute Febrile Illnesses in Children Requiring Hospitalization in a Tertiary Care Hospital in North India: A Prospective Study | English | India | India | 2014 | 2014 |
| 57 | Nadda, Nishant, et.al.^259^ | 2019 | A descriptive study to quantify severity in patients with severe scrub typhus in hilly city of Northern India | English | India | India | 2016.06 | 2017.05 |
| 58 | Roy, S., et.al.^260^ | 2019 | A study on scrub typhus in children: Experience in a tertiary care hospital | English | India | India | 2015.05 | 2016.04 |
| 59 | Sandhu, A., et.al.^261^ | 2019 | Assessment of Predictors for Severity in Scrub Typhus-A Case-Control Study | English | India | India | 2012.01 | 2015.12 |
| 60 | Gaba, S., et.al.^262^ | 2019 | Clinical outcome and predictors of severity in scrub typhus patients at a tertiary care hospital in Chandigarh, India | English | India | India | nan | nan |
| 61 | Sharma, Ritin, et.al.^263^ | 2019 | Predictors of Severity in Scrub Typhus | English | India | India | 2015 | 2016 |
| 62 | Jain, Deepak, et.al.^60^ | 2019 | Scrub typhus infection, not a benign disease: an experience from a tertiary care center in Northern India | English | India | India | 2017 | 2017 |
| 63 | Mallick, Sanjay Kumar, et.al.^62^ | 2019 | Scrub typhus: a hospital-based study in the northern districts of West Bengal, India | English | India | India | 2016 | 2018 |
| 64 | Saluja, M., et.al.^264^ | 2019 | Scrub typhus: Epidemiology, clinical presentation, diagnostic approach, and outcomes | English | India | India | 2017 | 2018 |
| 65 | Pathania, Monika, et.al.^265^ | 2019 | Scrub typhus: Overview of demographic variables, clinical profile, and diagnostic issues in the sub-Himalayan region of India and its comparison to other Indian and Asian studies | English | India | India | 2014 | 2014 |
| 66 | Jayaprakash, V., et.al.^266^ | 2019 | Scrub typhus-associated acute kidney injury: A study from a South Indian Tertiary Care Hospital | English | India | India | 2015 | 2017 |
| 67 | Perumalla, Susmitha Karunasree, et.al.^64^ | 2019 | Eschar and IgM ELISA in the diagnosis of scrub typhus | English | India | India | 2015 | 2017 |
| 68 | Nallasamy, Karthi, et.al.^267^ | 2020 | Clinical Profile and Predictors of Intensive Care Unit Admission in Pediatric Scrub Typhus: A Retrospective Observational Study from North India | English | India | India | 2013.01 | 2015.12 |
| 69 | Kiruthika, V. M., et.al.^268^ | 2020 | Clinical Profile and Predictors of Outcome for Paediatric Scrub Typhus at a Tertiary Care Hospital in South India | English | India | India | nan | nan |
| 70 | Alam, Areesha, et.al.^67^ | 2020 | Prediction Rule for Scrub Typhus Meningoencephalitis in Children: Emerging Disease in North India | English | India | India | 2016 | 2018 |
| 71 | Lakshmi, Ram Mohan Mylavarapu Venkata Naga, et.al.^71^ | 2020 | Prevalence of scrub typhus in a tertiary care centre in Telangana, south India | English | India | India | 2018 | 2018 |
| 72 | Thakur, C. K., et.al.^269^ | 2020 | Scrub typhus in patients with acute febrile illness: a 5-year study from India | English | India | India | 2013 | 2018 |
| 73 | Hazra, D., et.al.^69^ | 2020 | Scrub typhus: Clinical presentation and severity | English | India | India | 2012 | 2013 |
| 74 | Gaba, Saurabh, et.al.^270^ | 2020 | Hepatic and Renal Profile of Scrub Typhus Patients at a Tertiary Care Center in India | English | India | India | 2016 | 2018 |
| 75 | Pannu, A., et.al.^271^ | 2021 | Circulatory and hepatic failure at admission predicts mortality of severe scrub typhus patients: A prospective cohort study | English | India | India | 2017.07 | 2020.10 |
| 76 | Bansod, Yogendra V., et.al.^272^ | 2021 | Clinical Profile and Treatment Outcome in Scrub Typhus Patients in Central India | English | India | India | 2018 | 2018 |
| 77 | Singh, Sweta, et.al.^84^ | 2021 | Seroprevalence trends of Scrub typhus among the febrile patients of Northern India: A prospective cross-sectional study | English | India | India | 2017 | 2020 |
| 78 | Mahajan, Vidushi, et.al.^79^ | 2021 | Spectrum of Multiorgan Dysfunction in Scrub Typhus Infection | English | India | India | 2013 | 2017 |
| 79 | Barnabas, Rohit, et.al.^273^ | 2021 | Prospective study to assess the treatment modalities and fever defervescence in patients with scrub typhus from a tertiary care centre in South India | English | India | India | 2013 | 2015 |
| 80 | Roychowdhury, S., et.al.^274^ | 2021 | A Menace without Specific Feature - Scrub Typhus a Reemerging Disease | English | India | India | 2018 | 2020 |
| 81 | Williams, Vijai, et.al.^275^ | 2021 | Hyperferritinemia in children hospitalized with scrub typhus | English | India | India | 2019 | 2019 |
| 82 | Panda, A., et.al.^82^ | 2021 | Clinico-epidemiological and outcome of scrub typhus in paediatric patients: An observational study from Odisha, India | English | India | India | 2017 | 2019 |
| 83 | Muthukrishnan, K., et.al.^276^ | 2021 | Clinical profile and predictors of outcome for pediatric scrub typhus at a tertiary care hospital | English | India | India | 2012 | 2019 |
| 84 | Pannu, Ashok Kumar, et.al.^277^ | 2021 | Circulating cardiac biomarkers and echocardiographic abnormalities in patients with scrub typhus: A prospective cohort study from a tertiary care center in North India | English | India | India | 2017 | 2018 |
| 85 | Loganathan, S. K., et.al.^278^ | 2021 | Acute respiratory distress syndrome in paediatric scrub typhus | English | India | India | 2010 | 2015 |
| 86 | Parasher, V., et.al.^279^ | 2022 | Pattern of admission and clinical outcome of scrub typhus patients admitted in PICU in Southern Rajasthan | English | India | India | 2016 | 2020 |
| 87 | Sultan, A., et.al.^87^ | 2022 | Scrub Typhus: An Emerging Etiology among Undiagnosed Febrile Cases in Western Part of Uttar Pradesh, India | English | India | India | 2017 | 2020 |
| 88 | Punjabi, Narain H., et.al.^95^ | 2012 | Etiology of acute, non-malaria, febrile illnesses in Jayapura, northeastern Papua, Indonesia | English | Indonesia | Indonesia | 1997.11 | 2000.02 |
| 89 | Phongmany, Simaly, et.al.^106^ | 2006 | Rickettsial infections and fever, Vientiane, Laos | English | Laos | Laos | 2001 | 2003 |
| 90 | 王树声, et.al.^280^ | 1989 | 广西桂林市郊恙虫病暴发流行的流行病学、病原学和血清学研究 | Chinese | Mainland China | Mainland China | 1986 | 1986 |
| 91 | 张祥浩, et.al.^281^ | 1991 | 莒县恙虫病流行病学调查报告 | Chinese | Mainland China | Mainland China | 1989 | 1989 |
| 92 | Zhang, Li-juan, et.al.^282^ | 2007 | Molecular epidemic survey on co-prevalence of scrub typhus and marine typhus in Yuxi city, Yunnan province of China | English | Mainland China | Mainland China | 2002 | 2004 |
| 93 | 李剑萍, et.al.^283^ | 2009 | 30例恙虫病临床特征分析 | Chinese | Mainland China | Mainland China | 2003.06 | 2008.11 |
| 94 | 王晟^284^ | 2009 | 500例恙虫病并发症临床分析 | Chinese | Mainland China | Mainland China | 1993 | 2007 |
| 95 | 罗小松 ^285^ | 2010 | 小儿恙虫病150例临床分析 | Chinese | Mainland China | Mainland China | 2000.06 | 2009.10 |
| 96 | 廖云珍 and 叶晓光 ^286^ | 2013 | 广州地区恙虫病流行病学和临床特征分析 | Chinese | Mainland China | Mainland China | 1985 | 2012 |
| 97 | 罗雷, et.al.^287^ | 2013 | 广州一起公园恙虫病聚集性事件的现场调查与应急处置 | Chinese | Mainland China | Mainland China | 2012 | 2012 |
| 98 | 孙明, et.al.^288^ | 2013 | 宿迁地区32例恙虫病临床特征分析 | Chinese | Mainland China | Mainland China | 2010 | 2012 |
| 99 | Wei, Yuehong, et.al.^289^ | 2014 | A city park as a potential epidemic site of scrub typhus: a case-control study of an outbreak in Guangzhou, China | English | Mainland China | Mainland China | 2012.05 | 2012.06 |
| 100 | 郑伟华, et.al.^290^ | 2015 | 广东省中山地区恙虫病临床与流行病学特征及防治 | Chinese | Mainland China | Mainland China | 2009 | 2013 |
| 101 | 曾诚, et.al.^291^ | 2015 | 广西恙虫病并多器官损害168例临床特征分析 | Chinese | Mainland China | Mainland China | 2009 | 2014 |
| 102 | 杨松, et.al.^292^ | 2016 | 58例恙虫病并发肺部感染的流行病学及临床分析 | Chinese | Mainland China | Mainland China | 2013 | 2015 |
| 103 | 陆永姝, et.al.^293^ | 2017 | 24例重症恙虫病患者的临床观察与护理 | Chinese | Mainland China | Mainland China | 2014.01 | 2015.12 |
| 104 | Zhao, Dongying, et.al.^294^ | 2017 | Clinical Predictors of Multiple Organ Dysfunction Syndromes in Pediatric patients with Scrub Typhus | English | Mainland China | Mainland China | 2010.01 | 2015.01 |
| 105 | 吴超, et.al.^295^ | 2017 | 恙虫病误诊病例临床诊治剖析 | Chinese | Mainland China | Mainland China | nan | nan |
| 106 | Wang, Ying, et.al.^296^ | 2019 | [Analysis of the clinical characteristics of severe tsutsugamushi disease in Yunnan Province from 2017 to 2018] | Chinese | Mainland China | Mainland China | 2017 | 2018 |
| 107 | 何道兴.^297^ | 2021 | 南平地区260例恙虫病患者的临床分析及流行病特征 | Chinese | Mainland China | Mainland China | 2015 | 2020 |
| 108 | Brown, G. W., et.al.^168^ | 1984 | Febrile illness in Malaysia--an analysis of 1,629 hospitalized patients | English | Malaysia | Malaysia | 1975 | 1979 |
| 109 | Jensenius, Mogens, et.al.^298^ | 2013 | Acute and potentially life-threatening tropical diseases in western travelers--a GeoSentinel multicenter study, 1996-2011 | English | Norway | nan | 1996.06 | 2011.08 |
| 110 | Sedhain, A. and Bhattarai, G.B.^299^ | 2017 | Renal Manifestation in Scrub Typhus during a Major Outbreak in Central Nepal | English | Nepal | Nepal | 2016 | 2016 |
| 111 | Adhikari, Shital, et.al.^300^ | 2018 | Predictors of Mortality in Scrub Typhus Infection Requiring Intensive Care Admission in Tertiary Healthcare Centre of Nepal | English | Nepal | Nepal | 2016 | 2017 |
| 112 | Pathak, Santosh, et.al.^301^ | 2019 | Clinical profile, complications and outcome of scrub typhus in children: A hospital based observational study in central Nepal | English | Nepal | Nepal | 2016.07 | 2017.08 |
| 113 | Adhikari, Shital, et.al.^302^ | 2020 | Outcome Predictors in Scrub Typhus Requiring Ventilator and Vasopressor Support | English | Nepal | Nepal | 2016 | 2017 |
| 114 | Marks, Michael, et.al.^303^ | 2016 | An outbreak investigation of scrub typhus in Western Province, Solomon Islands, 2014 | English | United Kingdom | Solomon Islands | 2014.05.05 | 2014.05.11 |
| 115 | Lee, Chang-Seop, et.al.^304^ | 2009 | Risk factors leading to fatal outcome in scrub typhus patients | English | South Korea | South Korea | 2000 | 2006 |
| 116 | Moon, Kyoung Min, et.al.^305^ | 2016 | Risk Factors for Mechanical Ventilation in Patients with Scrub Typhus Admitted to Intensive Care Unit at a University Hospital | English | South Korea | South Korea | 2004 | 2014 |
| 117 | Hwang, Kyung, et.al.^306^ | 2017 | Incidence, risk factors and clinical outcomes of acute kidney injury associated with scrub typhus: a retrospective study of 510 consecutive patients in South Korea (2001-2013) | English | South Korea | South Korea | 2001 | 2013 |
| 118 | Yang, Shang-Hsien, et.al.^307^ | 2007 | Childhood scrub typhus in eastern Taiwan: ten-year experience from a medical center | English | Taiwan | Taiwan | 1997.01 | 2006.12 |
| 119 | Jim, Wai-Tim, et.al.^308^ | 2009 | Clinical manifestations, laboratory findings and complications of pediatric scrub typhus in eastern Taiwan | English | Taiwan | Taiwan | 1992.01.01 | 2002.12.31 |
| 120 | Watt, G., et.al.^309^ | 2003 | Differentiating dengue virus infection from scrub typhus in Thai adults with fever | English | Thailand | Thailand | 1991 | 1991 |
| 121 | Leelarasamee, Amorn, et.al.^310^ | 2004 | Etiologies of acute undifferentiated febrile illness in Thailand | English | Thailand | Thailand | 1991 | 1993 |
| 122 | Silpapojakul, Kamkarn, et.al.^311^ | 2004 | Paediatric scrub typhus in Thailand: a study of 73 confirmed cases | English | Thailand | Thailand | 1985 | 2002 |
| 123 | Charoensak, A., et.al.^312^ | 2006 | Scrub typhus: chest radiographic and clinical findings in 130 Thai patients | English | Thailand | Thailand | 2001 | 2002 |
| 124 | Suputtamongkol, Y., et.al.^212^ | 2009 | Epidemiology and clinical aspects of rickettsioses in Thailand | English | Thailand | Thailand | 2000.1 | 2003.03 |
| 125 | Thipmontree, Wilawan, et.al.^215^ | 2016 | Scrub Typhus in Northeastern Thailand: Eschar Distribution, Abnormal Electrocardiographic Findings, and Predictors of Fatal Outcome | English | Thailand | Thailand | 2011 | 2012 |
| 126 | Wangrangsimakul, Tri, et.al.^218^ | 2018 | Causes of acute undifferentiated fever and the utility of biomarkers in Chiangrai, northern Thailand | English | Thailand | Thailand | 2006.08 | 2008.10 |
| 127 | Nadjm, Behzad, et.al.^221^ | 2014 | Scrub typhus in the northern provinces of Vietnam: an observational study of admissions to a national referral hospital | English | Vietnam | Vietnam | 2001 | 2003 |
| 128 | Hamaguchi, Sugihiro, et.al.^222^ | 2015 | Clinical and Epidemiological Characteristics of Scrub Typhus and Murine Typhus among Hospitalized Patients with Acute Undifferentiated Fever in Northern Vietnam | English | Japan | Vietnam | 2001.03 | 2003.02 |
| 129 | Trung, Nguyen Vu, et.al.^313^ | 2019 | Clinical Manifestations and Molecular Diagnosis of Scrub Typhus and Murine Typhus, Vietnam, 2015-2017 | English | Vietnam | Vietnam | 2015.03 | 2017.03 |

*Note: nan=not available.*

# Appendix Table S10: Characteristics of included risk factor studies, in order of country/region and published year (n=37).

| **ID** | **Author** | **Year of publication** | **Title** | **Language** | **Publication country** | **Study conducted country** | **Start-time of investigation** | **End-time of investigation** |
| --- | --- | --- | --- | --- | --- | --- | --- | --- |
| 1 | Sharma, Puran K., et.al.^314^ | 2009 | Scrub typhus in Darjeeling, India: opportunities for simple, practical prevention measures | English | India | India | 2005 | 2005 |
| 2 | Varghese, George M., et.al.^315^ | 2016 | Epidemiology & risk factors of scrub typhus in south India | English | India | India | 2006 | 2011 |
| 3 | Trowbridge Paul et.al.^47^ | 2017 | Prevalence and risk factors for scrub typhus in South India | English | India | India | nan | nan |
| 4 | Thangaraj, Jeromie Wesley Vivian, et.al.^316^ | 2018 | Risk Factors for Acquiring Scrub Typhus among Children in Deoria and Gorakhpur Districts, Uttar Pradesh, India, 2017 | English | India | India | 2017.10.03 | 2017.11.11 |
| 5 | George, Tina, Rajan, et.al.^317^ | 2018 | Risk Factors for Acquiring Scrub Typhus among the Adults | English | India | India | 2013.10 | 2013.10 |
| 6 | Rose, Winsley, et.al.^318^ | 2019 | Risk factors for acquisition of scrub typhus in children admitted to a tertiary centre and its surrounding districts in South India: a case control study | English | India | India | 2015.10 | 2016.12 |
| 7 | Devamani, Carol S., et.al.^68^ | 2020 | Risk Factors for Scrub Typhus, Murine Typhus, and Spotted Fever Seropositivity in Urban Areas, Rural Plains, and Peri-Forest Hill Villages in South India: A Cross-Sectional Study | English | India | India | nan | nan |
| 8 | Vallee, Julie, et.al.^107^ | 2010 | Contrasting spatial distribution and risk factors for past infection with scrub typhus and murine typhus in Vientiane City, Lao PDR | English | France | Laos | 2006 | 2006 |
| 9 | Roberts, Tamalee, et.al.^319^ | 2021 | A spatio-temporal analysis of scrub typhus and murine typhus in Laos; implications from changing landscapes and climate | English | Laos | Laos | 2003.05 | 2017.10 |
| 10 | 季红燕, et.al.^320^ | 1998 | 68例少儿恙虫病1∶2匹配病例对照研究 | Chinese | Mainland China | Mainland China | 1992 | 1997 |
| 11 | 吴钦永, et.al.^321^ | 1998 | 山东省恙虫病危险因素病例的对照研究 | Chinese | Mainland China | Mainland China | 1995.09 | 1995.12 |
| 12 | 张倩, et.al.^138^ | 2007 | 内蒙古、新疆地区恙虫病流行病学调查研究 | Chinese | Mainland China | Mainland China | 2005 | 2006 |
| 13 | 查震球, et.al.^322^ | 2011 | 安徽省阜阳市恙虫病疫源地调查研究 | Chinese | Mainland China | Mainland China | 2009 | 2009 |
| 14 | 丁磊, et.al.^323^ | 2012 | 秋冬型恙虫病流行特征及影响因素研究 | Chinese | Mainland China | Mainland China | 2011.01 | 2011.03 |
| 15 | 方益荣, et.al.^324^ | 2012 | 安徽省阜阳市恙虫病疫源地调查研究 | Chinese | Mainland China | Mainland China | 2009 | 2009 |
| 16 | 刘隽, et.al.^325^ | 2013 | 广东省一起恙虫病暴发疫情流行病学调查 | Chinese | Mainland China | Mainland China | 2011.06 | 2011.07 |
| 17 | 邱尔臣,et.al.^152^ | 2013 | 新疆部分地区人群恙虫病血清流行病学调查 | Chinese | Mainland China | Mainland China | nan | nan |
| 18 | 张立芹, et.al.^326^ | 2013 | 北京市平谷区恙虫病危险因素的病例对照研究 | Chinese | Mainland China | Mainland China | 2011 | 2011 |
| 19 | 何义林, et.al.^327^ | 2014 | 2012年靖江市恙虫病危险因素的病例对照研究 | Chinese | Mainland China | Mainland China | 2012 | 2012 |
| 20 | Wei, Yuehong, et.al.^289^ | 2014 | A city park as a potential epidemic site of scrub typhus: a case-control study of an outbreak in Guangzhou, China | English | Mainland China | Mainland China | 2012.05 | 2012.06 |
| 21 | Hu, Jianli, et.al.^328^ | 2015 | Clinical characteristics and risk factors of an outbreak with scrub typhus in previously unrecognized areas, Jiangsu province, China 2013 | English | Mainland China | Mainland China | 2013.10 | 2013.11 |
| 22 | 李孟磊, et.al.^329^ | 2016 | 河南省一起恙虫病暴发疫情调查分析 | Chinese | Mainland China | Mainland China | 2015.10 | 2015.11 |
| 23 | 郭志南, et.al.^330^ | 2017 | 厦门市2013-2015年恙虫病流行特征和感染危险因素分析 | Chinese | Mainland China | Mainland China | 2013 | 2015 |
| 24 | 魏跃红, et.al.^331^ | 2017 | 广州市恙虫病危险因素的病例对照研究 | Chinese | Mainland China | Mainland China | 2015.05 | 2015.11 |
| 25 | 廖勇, et.al.^332^ | 2018 | 江西省赣州市恙虫病危险因素调查 | Chinese | Mainland China | Mainland China | 2015 | 2016 |
| 26 | 李大鹏, et.al.^333^ | 2019 | 广州市恙虫病危险因素与Ot-Sta56基因分型研究 | Chinese | Mainland China | Mainland China | 2015.01.01 | 2016.12.31 |
| 27 | 李鹏宾, et.al.^334^ | 2019 | 佛山市南海区恙虫病危险因素病例对照研究 | Chinese | Mainland China | Mainland China | 2018 | 2018 |
| 28 | 李飒,et.al.^163^ | 2019 | 北京市昌平区恙虫病血清流行病学调查及危险因素分析 | Chinese | Mainland China | Mainland China | 2017 | 2017 |
| 29 | Musa, T. H., et.al.^335^ | 2021 | Factors associated with Scrub Typhus infection: A case-control study from Luhe, China | English | Mainland China | Mainland China | 2015.10 | 2017.12 |
| 30 | Tay, Sun Tee, et.al.^174^ | 2013 | Antibody prevalence and factors associated with exposure to Orientia tsutsugamushi in different aboriginal subgroups in West Malaysia | English | Malaysia | Malaysia | 2007 | 2010 |
| 31 | Gautam, Rajendra, et.al.^180^ | 2019 | Epidemiology, Risk Factors and Seasonal Variation of Scrub Typhus Fever in Central Nepal | English | Nepal | Nepal | 2017 | 2018 |
| 32 | Kim, D. M., et.al.^336^ | 2008 | Risk-factors for human infection with Orientia tsutsugamushi: a case-control study in Korea | English | South Korea | South Korea | 2005 | 2005 |
| 33 | Kweon, Sun-Seog, et.al.^337^ | 2009 | A community-based case-control study of behavioral factors associated with scrub typhus during the autumn epidemic season in South Korea | English | South Korea | South Korea | 2007.10 | 2007.12 |
| 34 | Kim, Dong-Seob, et.al.^338^ | 2018 | Awareness and Work-Related Factors Associated with Scrub Typhus: A Case-Control Study from South Korea | English | South Korea | South Korea | 2015.01 | 2015.12 |
| 35 | Park, Ji-Hyuk, et.al.^198^ | 2021 | Seroprevalence and Factors Associated with Scrub Typhus Infection among Forestry Workers in National Park Offices in South Korea | English | South Korea | South Korea | 2016 | 2016 |
| 36 | Chang, Yi-Chin, et.al.^339^ | 2021 | Epidemiology and risk factors of scrub typhus in Taiwan: A nationwide database study from 1996 to 2014 | English | Taiwan | Taiwan | 1996 | 2014 |
| 37 | Tran, Hanh Thi Duc, et.al.^340^ | 2021 | Ecological and behavioural risk factors of scrub typhus in central Vietnam: a case-control study | English | Switzerland | Vietnam | 2018.08 | 2020.03 |

*Note: nan=not available.*

# Appendix Table S11: Quality assessment scale for rating the risk of bias.

| **Bias type** | **Score=2** | **Score=1** | **Score=0** |
| --- | --- | --- | --- |
| Selection 1 (sample population) | 1) Sample from the general population, not a select group.  2) Consecutive unselected population.  3) Rationale for case and control selection explained.  4) Inclusion and exclusion criteria clearly stated.  5) Patient’s demographic characteristics clearly described. | 1) Sample selected from large population, but selection criteria not defined.  2) Sample selection ambiguous but may be representative.  3) Rationale for cases and controls not explained.  4) Eligibility criteria not explained.  5) Analysis to adjust for sampling strategy bias. | 1) Highly select population making it difficult to generalise finding.  2) Sample selection ambiguous and sample unlikely to be representative. |
| Selection 2 (sample size) | 1) Sample size calculation performed and adequate.  2) The patient’s history clearly described and presented as a timeline for case report/series. | 1) Sample size calculation performed and reasons for not meeting sample size given.  2) Sample size calculation not performed but all eligible persons.  3) The patient’s history clearly described but no timeline described for case report/series. | 1) Not justified.  2) Sample size estimation unclear or only sub-sample studied.  3) No history or timeline described for case report/series. |
| Selection 3 (information definition) | 1) The case very clearly defined.  2) Measures of exposure/risk clearly defined, valid, reliable, and implemented consistently (including the same period) across all study participants. | 1) The case defined but lacks full clarity.  2) Measures of exposure/risk clear, valid, and reliable, but there may be minor inconsistencies in implementation. | 1) Case definitions are unclear or vague.  2) Measures of exposure/risk are unclear, invalid, unreliable, or inconsistently implemented. |
| Performance | 1) Assessment by the data from diagnosis using consistent criteria and direct examination.  2) The intervention(s) or treatment procedure(s) clearly described. | 1) Assessment from administrative database or register.  2) Assessment from hospital record or interviewer. | 1) Assessment from non-validated data or generic estimate from the overall population. |
| Outcome | 1) The statistical analysis is clearly described and appropriate.  2) The outcomes measured in a valid and reliable way.  3) Analysis accounts for common adjustments and includes control variables.  4) The case report/series provide takeaway lessons. | 1) Analysis does not account for common adjustment. | 1) The statistical analysis or the way to measure the outcome not appropriate, not described, or incomplete.  2) The outcomes are measured in an invalid or unreliable way.  3) Analysis does not account for common adjustments and lacks control variables. |

*Note: This table is adapted from the study "Global and regional prevalence, burden, and risk factors for carotid atherosclerosis: a systematic review, meta-analysis, and modelling study."^341^*

# Appendix Table S12: Quality scores for assessing the risk of bias in the included articles.

| **Study ID** | **Authors** | **Publication year** | **Selection 1** | **Selection 2** | **Selection 3** | **Performance** | **Outcome** | **Total** |
| --- | --- | --- | --- | --- | --- | --- | --- | --- |
| **Seroprevalence (N=315), mean score=7.83** | | | | | | | | |
| 1 | Graves, S. et.al.^1^ | 1999 | 1 | 2 | 2 | 2 | 1 | 8 |
| 2 | Harris PNA, et al. | 2016 | 1 | 2 | 2 | 2 | 1 | 8 |
| 3 | Mathews, Karen O. et.al.^2^ | 2021 | 1 | 2 | 2 | 2 | 1 | 8 |
| 4 | Arkell, P. et al. | 2022 | 2 | 2 | 2 | 2 | 2 | 10 |
| 5 | Maude RR, et al. | 2016 | 1 | 2 | 2 | 2 | 1 | 8 |
| 6 | Faruque, Labib Imran. et.al.^3^ | 2017 | 2 | 2 | 2 | 2 | 1 | 9 |
| 7 | Kingston, Hugh W. et.al.^4^ | 2018 | 2 | 2 | 2 | 2 | 1 | 9 |
| 8 | Al Amin MM, et al. | 2019 | 2 | 2 | 2 | 2 | 1 | 9 |
| 9 | Tshokey, Tshokey. et.al.^5^ | 2017 | 2 | 2 | 2 | 2 | 1 | 9 |
| 10 | Tshokey, Tshokey. et.al.^6^ | 2018 | 2 | 2 | 2 | 2 | 1 | 9 |
| 11 | Dorji, Kezang | 2019 | 2 | 2 | 2 | 2 | 1 | 9 |
| 12 | Tshokey, T. et.al.^7^ | 2019 | 2 | 1 | 2 | 2 | 0 | 7 |
| 13 | Chheng, Kheng. et.al.^8^ | 2013 | 2 | 2 | 2 | 2 | 1 | 9 |
| 14 | Mueller, Tara C. et.al.^9^ | 2014 | 2 | 2 | 2 | 2 | 1 | 9 |
| 15 | Horwood PF, et al. | 2017 | 1 | 2 | 2 | 2 | 1 | 8 |
| 16 | Weitzel, Thomas. et.al. ^10^ | 2020 | 2 | 2 | 2 | 2 | 2 | 10 |
| 17 | Horton, K. C. et.al. ^11^ | 2016 | 1 | 1 | 2 | 2 | 2 | 8 |
| 18 | Chao, Chien-Chung.et.al.^12^ | 2021 | 1 | 2 | 2 | 2 | 1 | 8 |
| 19 | Mathai, E. et al. | 2001 | 2 | 2 | 2 | 2 | 1 | 9 |
| 20 | Isaac, Rita.et.al.^13^ | 2004 | 2 | 1 | 1 | 2 | 0 | 6 |
| 21 | Varghese, G. M. et.al. ^14^ | 2006 | 2 | 2 | 1 | 2 | 1 | 8 |
| 22 | Kamarasu K, et al. | 2007 | 2 | 2 | 2 | 2 | 0 | 8 |
| 23 | Chrispal, Anugrah.et.al.^15^ | 2010 | 2 | 2 | 2 | 2 | 1 | 9 |
| 24 | Prabhakaran, A. et.al | 2010 | 2 | 2 | 2 | 2 | 1 | 9 |
| 25 | Basu, Gopal. et.al. ^16^ | 2011 | 2 | 2 | 1 | 2 | 1 | 8 |
| 26 | Rathi, Narendra B. et.al.^17^ | 2011 | 0 | 2 | 2 | 2 | 1 | 7 |
| 27 | Mittal, Veena.et.al.^18^ | 2012 | 2 | 1 | 1 | 2 | 1 | 7 |
| 28 | Narvencar, Kedareshwar P. S. et.al.^19^ | 2012 | 2 | 2 | 2 | 2 | 1 | 9 |
| 29 | Abrahamsen, Siri Kratter.et.al.^20^ | 2013 | 2 | 1 | 1 | 2 | 1 | 7 |
| 30 | S Gurung, et.al. | 2013 | 1 | 2 | 1 | 2 | 0 | 6 |
| 31 | Bithu, R. et.al.^21^ | 2014 | 2 | 2 | 1 | 2 | 1 | 8 |
| 32 | Griffith, Mathew. et.al.^22^ | 2014 | 1 | 2 | 2 | 2 | 1 | 8 |
| 33 | Kumar, Vive, et.al. | 2014 | 2 | 2 | 2 | 2 | 0 | 8 |
| 34 | Oberoi, Aroma.et.al.^23^ | 2014 | 2 | 1 | 1 | 2 | 1 | 7 |
| 35 | Singh, Ragini.et.al.^24^ | 2014 | 2 | 2 | 2 | 2 | 1 | 9 |
| 36 | Usha, K. et.al.^25^ | 2014 | 1 | 2 | 2 | 2 | 1 | 8 |
| 37 | Sankhyan N, et.al. | 2014 | 1 | 1 | 1 | 2 | 1 | 6 |
| 38 | Sinha P, et.al. | 2014 | 0 | 2 | 1 | 2 | 1 | 6 |
| 39 | Das, Dipmala. et.al. ^26^ | 2015 | 2 | 2 | 2 | 2 | 1 | 9 |
| 40 | Koraluru, Munegowda, et.al. | 2015 | 0 | 2 | 1 | 2 | 1 | 6 |
| 41 | Rashmi, K. S. et.al. ^27^ | 2015 | 0 | 2 | 2 | 2 | 1 | 7 |
| 42 | Roopa, K. S. et.al. ^28^ | 2015 | 1 | 2 | 1 | 2 | 1 | 7 |
| 43 | Sengupta, M. et.al.^29^ | 2015 | 2 | 1 | 2 | 2 | 1 | 8 |
| 44 | Stephen, Selvaraj. et.al. ^30^ | 2015 | 0 | 1 | 2 | 2 | 1 | 6 |
| 45 | Nawab T, et.al. | 2015 | 2 | 1 | 1 | 2 | 0 | 6 |
| 46 | Usha K, et.al. | 2015 | 0 | 2 | 2 | 2 | 0 | 6 |
| 47 | Abhilash, Kundavaram Paul Prabhakar. et.al.^31^ | 2016 | 2 | 2 | 2 | 2 | 1 | 9 |
| 48 | Sivarajan, Sunuraj, et.al | 2016 | 0 | 2 | 2 | 2 | 1 | 7 |
| 49 | Thomas, Rwituja, et.al. | 2016 | 0 | 2 | 2 | 2 | 1 | 7 |
| 50 | Usha, K.,et.al. | 2016 | 2 | 2 | 1 | 2 | 1 | 8 |
| 51 | Bhargava, Anurag. et.al. ^32^ | 2016 | 2 | 2 | 2 | 2 | 1 | 9 |
| 52 | Farhana, Anjum. et.al. ^33^ | 2016 | 2 | 2 | 2 | 2 | 1 | 9 |
| 53 | Jakharia, Aniruddha. et.al. ^34^ | 2016 | 2 | 1 | 1 | 2 | 2 | 8 |
| 54 | Kalal, B. S. et.al.^35^ | 2016 | 1 | 2 | 2 | 2 | 1 | 8 |
| 55 | Khan, S. A. et.al. ^36^ | 2016 | 2 | 2 | 2 | 2 | 1 | 9 |
| 56 | Rajan, S. J. et.al. ^37^ | 2016 | 1 | 2 | 1 | 2 | 1 | 7 |
| 57 | Sarangi, R. et.al. ^38^ | 2016 | 2 | 1 | 2 | 2 | 1 | 8 |
| 58 | Ahmad S, et al. | 2016 | 2 | 2 | 1 | 2 | 1 | 8 |
| 59 | Khan SA, et.al. | 2016 | 2 | 2 | 2 | 2 | 1 | 9 |
| 60 | Arun Babu, Thirunavukkarasu, et.al. ^39^ | 2017 | 2 | 2 | 2 | 2 | 1 | 9 |
| 61 | Manjunath, V. G. et.al. ^40^ | 2017 | 2 | 2 | 2 | 2 | 1 | 9 |
| 62 | Mina, S. S., et.al. ^41^ | 2017 | 2 | 2 | 2 | 2 | 1 | 9 |
| 62 | Mittal, Mahima. et.al. ^42^ | 2017 | 2 | 1 | 1 | 2 | 1 | 7 |
| 63 | Morch, Kristine. et.al. ^43^ | 2017 | 1 | 1 | 2 | 2 | 1 | 7 |
| 64 | Narvencar, Kedareshwar. et.al. ^44^ | 2017 | 2 | 2 | 2 | 2 | 1 | 9 |
| 65 | Shelke, Yogendra Pandurang.et.al.^45^ | 2017 | 2 | 1 | 2 | 2 | 1 | 8 |
| 66 | Singhi, Sunit. et.al. ^46^ | 2017 | 2 | 2 | 1 | 2 | 1 | 8 |
| 67 | Trowbridge, Paul. et.al. ^47^ | 2017 | 2 | 2 | 2 | 2 | 1 | 9 |
| 69 | Vivian Thangaraj, et.al. ^48^ | 2017 | 2 | 2 | 2 | 2 | 1 | 9 |
| 70 | Jain P, et al. | 2017 | 1 | 2 | 2 | 2 | 1 | 8 |
| 71 | Srinivasan S, Menon T. | 2017 | 0 | 1 | 2 | 2 | 1 | 6 |
| 72 | Arvind, N. et.al. ^49^ | 2018 | 2 | 2 | 2 | 2 | 1 | 9 |
| 73 | Giri, Prabahs Prasun, et.al.^50^ | 2018 | 1 | 2 | 1 | 2 | 1 | 7 |
| 74 | Jacob, S. M. et.al. ^51^ | 2018 | 2 | 1 | 1 | 2 | 1 | 7 |
| 75 | Jain, Parul. et.al. ^52^ | 2018 | 1 | 2 | 2 | 2 | 1 | 8 |
| 76 | Raina, Sujeet. et.al. ^53^ | 2018 | 2 | 2 | 2 | 2 | 1 | 9 |
| 77 | Rauf, Abdul. et.al.^54^ | 2018 | 1 | 2 | 1 | 2 | 1 | 7 |
| 78 | Rizvi, Meher. et.al. ^55^ | 2018 | 2 | 2 | 2 | 2 | 1 | 9 |
| 79 | Koralur, Munegowda, et.al. | 2018 | 0 | 2 | 2 | 2 | 1 | 7 |
| 80 | Stephen, Selvaraj. et.al. ^56^ | 2018 | 1 | 2 | 1 | 2 | 1 | 7 |
| 81 | Mittal M, et.al. | 2018 | 1 | 2 | 2 | 2 | 1 | 8 |
| 82 | Behera, Bijayini.et.al. ^57^ | 2019 | 2 | 2 | 2 | 2 | 1 | 9 |
| 83 | Boda, Subbarayudu. and Goutham, V.V.N. ^58^ | 2019 | 2 | 2 | 1 | 2 | 1 | 8 |
| 84 | Devamani, Carol S. et.al. ^59^ | 2019 | 2 | 1 | 2 | 2 | 1 | 8 |
| 85 | Jain, Deepak. et.al. ^60^ | 2019 | 2 | 2 | 2 | 2 | 1 | 9 |
| 86 | Jha, Ruchi and Anil Kumar Jaiswal ^61^ | 2019 | 1 | 2 | 0 | 2 | 1 | 6 |
| 87 | Mallick, Sanjay Kumar. et.al.^62^ | 2019 | 2 | 2 | 1 | 2 | 1 | 8 |
| 88 | Patil, R. and Kulkarni, S.^63^ | 2019 | 2 | 2 | 2 | 2 | 1 | 9 |
| 89 | Perumalla, Susmitha Karunasree, et.al. ^64^ | 2019 | 2 | 2 | 2 | 2 | 0 | 8 |
| 90 | Rao, Pavitra N. et.al. ^65^ | 2019 | 2 | 2 | 2 | 2 | 1 | 9 |
| 91 | Shankar, V., et.al. ^66^ | 2019 | 0 | 1 | 1 | 2 | 1 | 5 |
| 92 | Bal M, et al. | 2019 | 2 | 2 | 1 | 2 | 1 | 8 |
| 93 | Basu S, et al. | 2019 | 0 | 1 | 2 | 2 | 0 | 5 |
| 94 | Alam, Areesha. et.al. ^67^ | 2020 | 1 | 2 | 2 | 2 | 1 | 8 |
| 95 | Devamani, Carol S. et.al. ^68^ | 2020 | 2 | 2 | 2 | 2 | 1 | 9 |
| 96 | Hazra, D. et.al. ^69^ | 2020 | 2 | 2 | 1 | 2 | 1 | 8 |
| 97 | Kamble, Suchit. et.al. ^70^ | 2020 | 1 | 2 | 2 | 2 | 1 | 8 |
| 98 | Lakshmi, Ram Mohan Mylavarapu Venkata Naga, et.al. ^71^ | 2020 | 1 | 2 | 2 | 2 | 1 | 8 |
| 99 | Vikram, Kumar. et.al. ^72^ | 2020 | 2 | 2 | 2 | 2 | 1 | 9 |
| 100 | Thakur, C. K. et.al. | 2020 | 2 | 2 | 2 | 2 | 1 | 9 |
| 101 | Yaqoob, S. et.al. ^73^ | 2020 | 2 | 2 | 2 | 2 | 1 | 9 |
| 102 | Tiwari, S.Nanda, M. | 2020 | 0 | 2 | 1 | 2 | 1 | 6 |
| 103 | Anitharaj V, | 2020 | 0 | 2 | 2 | 2 | 0 | 6 |
| 104 | Saravanan N, et al. | 2020 | 2 | 2 | 1 | 2 | 1 | 8 |
| 106 | Anupriya, A., et.al. ^74^ | 2021 | 2 | 2 | 2 | 2 | 1 | 9 |
| 107 | Bal, Madhusmita, et.al. ^75^ | 2021 | 1 | 2 | 2 | 2 | 1 | 8 |
| 108 | Behera, Sthita Pragnya, et.al. ^76^ | 2021 | 2 | 2 | 1 | 2 | 1 | 8 |
| 109 | Chaudhari SP, et al. | 2021 | 2 | 2 | 1 | 2 | 1 | 8 |
| 110 | Kavirayani, V. et.al. ^77^ | 2021 | 2 | 2 | 2 | 2 | 1 | 9 |
| 111 | Khan, Siraj A. et.al. ^78^ | 2021 | 2 | 2 | 1 | 2 | 1 | 8 |
| 112 | Mahajan, Vidushi, et.al. ^79^ | 2021 | 2 | 2 | 1 | 2 | 1 | 8 |
| 113 | Matlani, M. et.al. ^80^ | 2021 | 2 | 2 | 1 | 2 | 1 | 8 |
| 114 | Mansoor, Tabeen, et.al. | 2021 | 2 | 2 | 1 | 2 | 1 | 8 |
| 115 | Mittal, Vineeta S, et.al. ^81^ | 2021 | 2 | 2 | 1 | 2 | 1 | 8 |
| 115 | Panda, A., et.al. ^82^ | 2021 | 1 | 2 | 1 | 2 | 1 | 7 |
| 116 | Paulraj, Philip Samuel, et.al. ^83^ |  | 2 | 1 | 2 | 2 | 1 | 8 |
| 117 | Singh, Sweta, et.al. ^84^ | 2021 | 2 | 2 | 2 | 2 | 1 | 9 |
| 118 | Somasunder, V. M. et.al. ^85^ | 2021 | 2 | 2 | 2 | 2 | 1 | 9 |
| 119 | Raychaudhuri D, et al. | 2021 | 1 | 2 | 1 | 2 | 0 | 6 |
| 120 | Husain, Uneza, et.al. ^86^ | 2022 | 2 | 2 | 2 | 2 | 1 | 9 |
| 121 | Sultan, A., et.al. ^87^ | 2022 | 2 | 2 | 2 | 2 | 1 | 9 |
| 122 | Tandale BV, | 2022 | 1 | 2 | 1 | 2 | 1 | 7 |
| 123 | Mondal, T., | 2022 | 2 | 2 | 1 | 2 | 1 | 8 |
| 124 | Devamani, C. S., | 2022 | 2 | 2 | 2 | 2 | 0 | 8 |
| 125 | Narang, R. et al. | 2022 | 2 | 2 | 2 | 2 | 2 | 10 |
| 126 | Bhatia, M. et al. | 2022 | 2 | 1 | 1 | 2 | 1 | 7 |
| 127 | Nanaware, N. et al. | 2022 | 1 | 1 | 2 | 2 | 1 | 7 |
| 128 | D'Cruz, S., | 2022 | 2 | 2 | 2 | 2 | 1 | 9 |
| 129 | Baidya, A., | 2022 | 1 | 2 | 1 | 2 | 2 | 8 |
| 130 | Sharma, S.,. | 2022 | 2 | 2 | 1 | 2 | 1 | 8 |
| 131 | Bhowmick, I. P. et al. | 2022 | 1 | 2 | 1 | 2 | 1 | 7 |
| 132 | Wolff JW, De Graaf W^88^ | 1939 | 1 | 2 | 2 | 2 | 1 | 8 |
| 133 | Dennis DT, et.al. ^89^ | 1981 | 2 | 2 | 2 | 2 | 1 | 9 |
| 134 | Gandahusada S, et al.^90^ | 1981 | 2 | 1 | 2 | 2 | 1 | 8 |
| 135 | Hadi TR, et.al. ^91^ | 1984 | 2 | 2 | 2 | 2 | 1 | 9 |
| 136 | Corwin, A. L. et.al. ^92^ | 1997 | 2 | 2 | 2 | 2 | 2 | 10 |
| 137 | Richards, A. L. et.al. ^93^ | 1997 | 1 | 2 | 2 | 2 | 1 | 8 |
| 138 | Richards, Allen L. et.al. ^94^ | 2003 | 2 | 1 | 1 | 2 | 1 | 7 |
| 139 | Punjabi, Narain H. et.al. ^95^ | 2012 | 1 | 2 | 2 | 2 | 1 | 8 |
| 140 | Lokida, Dewi, et.al. ^96^ | 2020 | 2 | 1 | 2 | 2 | 1 | 8 |
| 141 | Hamzah, I. H. et.al. ^97^ | 2020 | 1 | 1 | 2 | 2 | 1 | 7 |
| 142 | Olson, J. G. et.al. ^98^ | 1979 | 1 | 2 | 2 | 2 | 1 | 8 |
| 143 | Miyairi, T. et.al. ^99^ | 1980 | 1 | 2 | 1 | 2 | 1 | 7 |
| 144 | Takada, N. et.al. ^100^ | 1984 | 2 | 1 | 2 | 2 | 1 | 8 |
| 145 | Kasuya, S. et.al. ^101^ | 1985 | 2 | 0 | 1 | 2 | 1 | 6 |
| 146 | Ishikura, M. et.al. ^102^ | 1987 | 1 | 1 | 2 | 2 | 1 | 7 |
| 147 | Sando, Eiichiro, et.al. ^103^ | 2006 | 0 | 1 | 1 | 2 | 1 | 5 |
| 147 | Thiga, Jacqueline W. et.al. ^104^ | 2018 | 0 | 2 | 2 | 2 | 1 | 7 |
| 148 | Maina, A. N. et.al. ^105^ | 2008 | 1 | 0 | 2 | 2 | 1 | 6 |
| 148 | Phongmany, Simaly. et.al. ^106^ | 2015 | 2 | 2 | 2 | 2 | 1 | 9 |
| 149 | Vallee, Julie, et.al. ^107^ | 2016 | 2 | 2 | 2 | 2 | 1 | 9 |
| 150 | Syhavong B, et al. | 2006 | 2 | 2 | 2 | 2 | 1 | 9 |
| 151 | Mayxay, Mayfong, et.al. ^108^ | 2010 | 2 | 2 | 1 | 2 | 2 | 9 |
| 152 | Dittrich, Sabine, et.al. ^109^ | 2010 | 2 | 2 | 2 | 2 | 1 | 9 |
| 153 | Mayxay M, et al. | 2013 | 2 | 2 | 2 | 2 | 1 | 9 |
| 154 | Phetsouvanh R, et al. | 2015 | 1 | 2 | 2 | 2 | 1 | 8 |
| 156 | Mayxay M, et al. | 2015 | 0 | 2 | 1 | 2 | 1 | 6 |
| 157 | Chansamouth, Vilada, et.al. ^110^ | 2015 | 1 | 2 | 2 | 2 | 1 | 8 |
| 158 | Roberts, Tamalee, et.al. | 2016 | 1 | 2 | 2 | 2 | 1 | 8 |
| 159 | 高韵苕, et.al.^111^ | 2021 | 0 | 2 | 1 | 2 | 1 | 6 |
| 160 | 刘国栋, et.al.^112^ | 1960 | 2 | 2 | 2 | 2 | 1 | 9 |
| 161 | 耿际泉,et.al.^113^ | 1987 | 2 | 1 | 2 | 2 | 1 | 8 |
| 162 | 张鸿武 ^114^ | 1987 | 1 | 2 | 2 | 2 | 1 | 8 |
| 163 | 刘国栋, et,al.^115^ | 1991 | 2 | 2 | 2 | 2 | 1 | 9 |
| 164 | Lu, Z. et.al. ^116^ | 1992 | 2 | 2 | 2 | 2 | 1 | 9 |
| 165 | 王均利, et.al. ^117^ | 1994 | 1 | 1 | 2 | 2 | 1 | 7 |
| 166 | 胡玲美, et.al. ^118^ | 1996 | 2 | 1 | 2 | 2 | 1 | 8 |
| 167 | Yang, Z. Q., et.al. ^119^ | 1996 | 2 | 1 | 2 | 2 | 1 | 8 |
| 168 | 王如德, 怀燕 | 1997 | 0 | 2 | 2 | 2 | 1 | 7 |
| 169 | 周剑平, et.al.^120^ | 1997 | 0 | 2 | 2 | 2 | 1 | 7 |
| 170 | 郑乡占, et.al. ^121^ | 1997 | 2 | 2 | 2 | 2 | 1 | 9 |
| 171 | 黄昭穗, et.al. ^122^ | 1997 | 2 | 1 | 2 | 2 | 1 | 8 |
| 172 | 鲁志新, et.al. ^123^ | 1998 | 1 | 1 | 2 | 2 | 1 | 7 |
| 173 | 刘运喜, et.al. ^124^ | 1999 | 2 | 2 | 2 | 2 | 1 | 9 |
| 174 | Wang, S.et.al. ^125^ | 1999 | 2 | 1 | 2 | 2 | 1 | 8 |
| 175 | 陈香蕊, et.al. ^126^ | 2000 | 1 | 0 | 2 | 2 | 1 | 6 |
| 176 | 唐天开, et.al. ^127^ | 2000 | 1 | 1 | 1 | 2 | 1 | 6 |
| 177 | 袁庆虹,et.al. ^128^ | 2001 | 2 | 2 | 2 | 2 | 1 | 9 |
| 178 | 陈素良, et.al. ^129^ | 2001 | 2 | 1 | 1 | 2 | 1 | 7 |
| 179 | 陈素良, et.al. ^130^ | 2001 | 2 | 0 | 2 | 2 | 1 | 7 |
| 180 | 林碧瑚, et.al. ^131^ | 2001 | 2 | 1 | 2 | 2 | 1 | 8 |
| 181 | 袁高林,et.al. ^132^ | 2003 | 2 | 2 | 2 | 2 | 1 | 9 |
| 182 | 操敏, et.al. ^133^ | 2003 | 1 | 0 | 1 | 2 | 1 | 5 |
| 183 | 吴志伟, et.al. ^134^ | 2004 | 1 | 2 | 2 | 2 | 1 | 8 |
| 184 | Hengbin, Guo, et.al. ^135^ | 2005 | 2 | 2 | 1 | 2 | 1 | 8 |
| 185 | 周建荣, et.al. ^136^ | 2006 | 1 | 2 | 2 | 2 | 1 | 8 |
| 186 | 尹骏 and展海燕^137^ | 2007 | 2 | 2 | 1 | 2 | 0 | 7 |
| 187 | 张倩, et.al. ^138^ | 2007 | 2 | 2 | 2 | 2 | 1 | 9 |
| 188 | 吴中发, et.al. ^139^ | 2007 | 2 | 2 | 2 | 2 | 1 | 9 |
| 189 | 林光宇, et.al. ^140^ | 2008 | 1 | 2 | 1 | 2 | 1 | 7 |
| 190 | 王珊珊, et.al. ^141^ | 2008 | 1 | 2 | 2 | 2 | 1 | 8 |
| 191 | Liu YX, et al. | 2008 | 2 | 1 | 2 | 2 | 1 | 8 |
| 192 | Chai, Cheng-liang, et.al. ^142^ | 2009 | 0 | 2 | 2 | 2 | 0 | 6 |
| 193 | 刘红, et.al. ^143^ | 2010 | 2 | 2 | 2 | 2 | 1 | 9 |
| 194 | 薛家芹, et.al. ^144^ | 2010 | 2 | 2 | 2 | 2 | 1 | 9 |
| 195 | 何似, 谢忠杭, 陈阳,陈亮,邓艳琴,王灵岚, | 2010 | 2 | 1 | 1 | 2 | 1 | 7 |
| 196 | 张颖, et.al. ^145^ | 2011 | 0 | 2 | 1 | 2 | 1 | 6 |
| 197 | 苏静静 and 杨占清^146^ | 2011 | 1 | 1 | 2 | 2 | 1 | 7 |
| 198 | 姜仁杰, et.al. ^147^ | 2011 | 2 | 2 | 2 | 2 | 1 | 9 |
| 199 | 谭兆营, et.al. ^148^ | 2011 | 2 | 2 | 1 | 2 | 1 | 8 |
| 200 | Liu YX, et al. | 2012 | 2 | 2 | 2 | 2 | 1 | 9 |
| 201 | 常利涛, et.al. ^149^ | 2013 | 0 | 2 | 2 | 2 | 0 | 6 |
| 202 | 张立芹, et.al. ^150^ | 2013 | 2 | 1 | 2 | 2 | 1 | 8 |
| 203 | 田丽丽, et.al. ^151^ | 2013 | 2 | 2 | 1 | 2 | 1 | 8 |
| 204 | 邱尔臣, et.al. ^152^ | 2013 | 2 | 2 | 2 | 2 | 1 | 9 |
| 205 | 李静, et.al.^153^ | 2013 | 2 | 2 | 2 | 2 | 1 | 9 |
| 206 | 申安然, et.al.^154^ | 2014 | 2 | 1 | 1 | 2 | 1 | 7 |
| 207 | De, Wu, et.al. | 2014 | 0 | 1 | 1 | 2 | 1 | 5 |
| 208 | 谭文文, et.al.^155^ | 2015 | 1 | 1 | 2 | 2 | 1 | 7 |
| 209 | 韩腾伟, et.al.^156^ | 2015 | 2 | 2 | 2 | 2 | 1 | 9 |
| 210 | 吕燕宁, et.al.^157^ | 2015 | 0 | 2 | 1 | 2 | 1 | 6 |
| 211 | 张曙光, et.al.^158^ | 2016 | 2 | 2 | 2 | 2 | 1 | 9 |
| 212 | 陈永亮, et.al.^159^ | 2016 | 2 | 2 | 2 | 2 | 1 | 9 |
| 213 | 展海燕, et.al.^160^ | 2016 | 1 | 1 | 1 | 2 | 1 | 6 |
| 214 | 张欢, et.al.^161^ | 2017 | 2 | 2 | 1 | 2 | 1 | 8 |
| 215 | 张立芹, et.al.^162^ | 2018 | 1 | 2 | 1 | 2 | 1 | 7 |
| 216 | 李飒, et.al.^163^ | 2018 | 1 | 1 | 2 | 2 | 1 | 7 |
| 217 | Gu, X. L. et.al.^164^ | 2019 | 2 | 1 | 1 | 2 | 1 | 7 |
| 218 | Rainey, J. J. et al. | 2021 | 0 | 2 | 2 | 2 | 1 | 7 |
| 219 | Cadigan, F. C., et.al.^165^ | 2022 | 2 | 2 | 2 | 2 | 1 | 9 |
| 220 | Brown, G. W. et.al.^166^ | 1972 | 2 | 1 | 2 | 2 | 1 | 8 |
| 221 | Brown, G. W. et.al.^167^ | 1976 | 2 | 2 | 2 | 2 | 1 | 9 |
| 222 | Brown GW, et.al. | 1978 | 0 | 2 | 2 | 2 | 1 | 7 |
| 223 | Shirai A, et.al. | 1978 | 0 | 2 | 0 | 2 | 1 | 5 |
| 224 | Brown, G. W. et.al.^168^ | 1981 | 1 | 2 | 2 | 2 | 0 | 7 |
| 225 | Taylor, A. et.al.^169^ | 1984 | 2 | 2 | 2 | 2 | 1 | 9 |
| 226 | Tee, T. S. et.al.^170^ | 1986 | 1 | 2 | 1 | 2 | 0 | 6 |
| 227 | Sagin, D. et.al.^171^ | 1999 | 1 | 2 | 2 | 2 | 1 | 8 |
| 228 | Tay, S. T. et.al.^172^ | 2000 | 2 | 1 | 2 | 2 | 1 | 8 |
| 229 | Tay, S. T. et.al.^173^ | 2000 | 2 | 0 | 2 | 2 | 1 | 7 |
| 230 | Tay, Sun Tee et.al.^174^ | 2003 | 0 | 2 | 2 | 2 | 1 | 7 |
| 231 | Grigg, Matthew J., et.al. ^175^ | 2013 | 2 | 1 | 2 | 2 | 2 | 9 |
| 232 | Elders, Philip N. D. et.al. ^176^ | 2020 | 1 | 2 | 2 | 2 | 1 | 8 |
| 233 | Brown GW, et.al. | 2021 | 1 | 1 | 2 | 2 | 2 | 8 |
| 234 | Murdoch, David R. et.al. ^177^ | 1981 | 0 | 2 | 2 | 2 | 1 | 7 |
| 235 | Blacksell, Stuart D. et.al. ^178^ | 2004 | 2 | 2 | 2 | 2 | 1 | 9 |
| 236 | Upadhyaya, B. et.al. | 2007 | 2 | 2 | 2 | 2 | 1 | 9 |
| 237 | Sedhain, A. and Bhattarai, G. R.^179^ | 2016 | 1 | 2 | 2 | 2 | 1 | 8 |
| 238 | Pathak, Santosh, et.al. | 2017 | 2 | 2 | 2 | 2 | 1 | 9 |
| 239 | Dhimal, Meghnath,et.al. | 2019 | 0 | 2 | 1 | 2 | 1 | 6 |
| 240 | Gautam, Rajendra, et.al. ^180^ | 2019 | 0 | 1 | 2 | 2 | 1 | 6 |
| 241 | Sharma, K., et.al. ^181^ | 2019 | 2 | 2 | 2 | 2 | 1 | 9 |
| 242 | Thapa, Sangita, et.al. ^182^ | 2019 | 2 | 2 | 1 | 2 | 1 | 8 |
| 243 | Gautam, Rajendra, et.al. | 2020 | 2 | 2 | 2 | 2 | 1 | 9 |
| 244 | Gautam, R.et.al. | 2020 | 0 | 2 | 2 | 2 | 0 | 6 |
| 245 | Pokhrel, Anil, et.al. ^183^ | 2021 | 0 | 2 | 2 | 2 | 1 | 7 |
| 246 | Madhup, S. K. et.al. | 2021 | 2 | 2 | 1 | 2 | 1 | 8 |
| 247 | Shrestha, S. et al. | 2021 | 0 | 2 | 2 | 2 | 1 | 7 |
| 248 | Demma, Linda J., et.al. ^184^ | 2022 | 2 | 2 | 2 | 2 | 1 | 9 |
| 249 | Spicer, P. E., et.al. ^185^ | 2006 | 2 | 1 | 2 | 2 | 1 | 8 |
| 250 | Kocher, C., et.al. ^186^ | 2007 | 2 | 1 | 1 | 2 | 1 | 7 |
| 251 | Yen, Tsai-Ying, et.al. ^187^ | 2017 | 2 | 2 | 2 | 2 | 1 | 9 |
| 252 | Muzaheed, et.al. ^188^ | 2019 | 0 | 2 | 2 | 2 | 1 | 7 |
| 253 | Miles, J. A. R., et.al. | 2020 | 2 | 2 | 1 | 2 | 0 | 7 |
| 254 | Chang, W. H., et.al. ^189^ | 1981 | 2 | 1 | 2 | 2 | 1 | 8 |
| 255 | Chang, W. H., et.al. ^190^ | 1989 | 2 | 2 | 1 | 2 | 1 | 8 |
| 256 | Jin-Won, Song, et.al. ^191^ | 1990 | 2 | 2 | 1 | 2 | 1 | 8 |
| 257 | Jin-Won, Song, et.al. ^192^ | 1998 | 2 | 2 | 2 | 2 | 1 | 9 |
| 258 | Min-Ja, K. I. M., et.al. ^193^ | 1998 | 2 | 2 | 2 | 2 | 1 | 9 |
| 259 | Song, J. W., et.al. ^194^ | 2001 | 2 | 2 | 1 | 2 | 1 | 8 |
| 260 | Jang, Won-Jong, et.al. ^195^ | 2002 | 2 | 2 | 1 | 2 | 1 | 8 |
| 261 | Jung, Ho-Chul, et.al. ^196^ | 2004 | 2 | 2 | 1 | 2 | 1 | 8 |
| 262 | Dong-Seob, K. I. M., et.al.^197^ | 2015 | 2 | 2 | 2 | 2 | 1 | 9 |
| 263 | Hwang JH, et.al. | 2019 | 1 | 2 | 2 | 2 | 1 | 8 |
| 264 | Yen NTH, et.al. | 2019 | 0 | 2 | 2 | 2 | 0 | 6 |
| 265 | Park, Ji-Hyuk, et.al. ^198^ | 2019 | 1 | 1 | 2 | 2 | 0 | 6 |
| 266 | Um, J., et.al. ^199^ | 2021 | 1 | 2 | 2 | 2 | 1 | 8 |
| 267 | Premaratna R, et.al. | 2021 | 1 | 1 | 2 | 2 | 1 | 7 |
| 268 | Liyanapathirana, Veranja, et.al. ^200^ | 2008 | 0 | 1 | 2 | 2 | 0 | 5 |
| 269 | Kularatne, S. A. M., et.al. ^201^ | 2011 | 0 | 2 | 1 | 2 | 0 | 5 |
| 270 | Premaratna, Ranjan, et.al. ^202^ | 2013 | 0 | 2 | 2 | 2 | 1 | 7 |
| 271 | Pradeepan JA, et.al. | 2014 | 2 | 1 | 2 | 2 | 1 | 8 |
| 272 | Gayani Tillekeratne L, et al. | 2014 | 0 | 1 | 2 | 2 | 0 | 5 |
| 273 | Bourgeois, A. L. et.al. | 2020 | 2 | 2 | 2 | 2 | 1 | 9 |
| 274 | Olson, J. G. and Bourgeois, A. L.^203^ | 1977 | 0 | 1 | 2 | 2 | 1 | 6 |
| 275 | Yang, Hui-Hua,et.al. | 1977 | 1 | 1 | 2 | 2 | 1 | 7 |
| 276 | Lai CH, et al. | 2012 | 0 | 2 | 2 | 2 | 1 | 7 |
| 277 | Tsai KH, et al. | 2014 | 1 | 2 | 2 | 2 | 1 | 8 |
| 278 | Johnson, D. E., et.al. ^204^ | 2019 | 0 | 2 | 2 | 2 | 0 | 6 |
| 279 | Duffy, P. E., et.al. ^205^ | 1982 | 2 | 2 | 1 | 2 | 1 | 8 |
| 280 | Silpapojakul, K., et.al. ^206^ | 1990 | 2 | 1 | 2 | 2 | 0 | 7 |
| 281 | Strickman, D., et.al. ^207^ | 1991 | 2 | 2 | 2 | 2 | 1 | 9 |
| 282 | Eamsila, C., et.al.^208^ | 1994 | 2 | 2 | 2 | 2 | 1 | 9 |
| 283 | Frances, S. P., et.al. ^209^ | 1996 | 1 | 2 | 2 | 2 | 1 | 8 |
| 284 | Chanyasanha, C., et.al. ^210^ | 1997 | 1 | 2 | 2 | 2 | 1 | 8 |
| 285 | Tanskul P, et al. | 1998 | 2 | 2 | 2 | 2 | 1 | 9 |
| 286 | Chinprasatsak S, et al. | 1998 | 0 | 2 | 2 | 2 | 1 | 7 |
| 287 | Thap LC, et.al. | 2001 | 2 | 1 | 2 | 2 | 1 | 8 |
| 288 | Pradutkanchana, Jintana, et.al. ^211^ | 2002 | 1 | 1 | 2 | 2 | 1 | 7 |
| 289 | Leelarasamee, Amorn,et.al. | 2003 | 2 | 2 | 2 | 2 | 1 | 9 |
| 290 | T. Singhsilarak, S., et al. | 2004 | 2 | 2 | 2 | 2 | 1 | 9 |
| 291 | Suttinont C, et al. | 2006 | 1 | 2 | 1 | 2 | 1 | 7 |
| 292 | Suputtamongkol, Y., et.al. ^212^ | 2006 | 2 | 1 | 2 | 2 | 1 | 8 |
| 293 | Thitivichianlert, Sataporn, et.al. ^213^ | 2009 | 2 | 2 | 1 | 2 | 1 | 8 |
| 294 | McGready R, et al. | 2009 | 2 | 2 | 2 | 2 | 1 | 9 |
| 295 | Wuttikon Rodkvamtook, et.al. | 2010 | 1 | 2 | 2 | 2 | 1 | 8 |
| 296 | Ruang-areerate T, et.al. | 2011 | 1 | 2 | 1 | 2 | 1 | 7 |
| 297 | W. Rodkvamtook, et al. | 2011 | 1 | 2 | 2 | 2 | 1 | 8 |
| 298 | Thipmontree, Wilawan, et.al. ^214^ | 2013 | 2 | 2 | 1 | 2 | 0 | 7 |
| 299 | Thipmontree, Wilawan, et.al. ^215^ | 2014 | 2 | 2 | 2 | 2 | 1 | 9 |
| 300 | Bhengsri S, et al. | 2016 | 2 | 2 | 2 | 2 | 1 | 9 |
| 301 | Hinjoy, S., et.al. ^216^ | 2016 | 2 | 2 | 2 | 2 | 1 | 9 |
| 302 | Rodkvamtook, W., et.al. ^217^ | 2017 | 2 | 2 | 2 | 2 | 1 | 9 |
| 303 | Wangrangsimakul, Tri, et.al. ^218^ | 2018 | 1 | 1 | 2 | 2 | 1 | 7 |
| 304 | Chaisiri, Kittipong, et.al. ^219^ | 2018 | 2 | 2 | 2 | 2 | 1 | 9 |
| 305 | Gonwong, Siriphan, et.al.^220^ | 2022 | 2 | 2 | 2 | 2 | 2 | 10 |
| 306 | ProMed | 2022 | 2 | 0 | 2 | 2 | 2 | 8 |
| 307 | Nadjm, Behzad, et.al. ^221^ | 1998 | 1 | 0 | 0 | 0 | 1 | 2 |
| 308 | Hamaguchi, Sugihiro, et.al.^222^ | 2014 | 2 | 2 | 2 | 2 | 1 | 9 |
| 309 | Nguyen, Hang L. K., et.al.^223^ | 2015 | 2 | 2 | 2 | 2 | 1 | 9 |
| 311 | Trung, Nguyen Vu, et.al. ^224^ | 2017 | 2 | 2 | 1 | 2 | 1 | 8 |
| 312 | Katoh, Shungo, et.al. ^225^ | 2019 | 1 | 2 | 2 | 2 | 1 | 8 |
| 313 | Le-Viet, Nhiem, et.al. ^226^ | 2019 | 2 | 2 | 2 | 2 | 1 | 9 |
| 314 | Trung, Nguyen et.al. | 2019 | 0 | 2 | 2 | 2 | 1 | 7 |
| 315 | Trung, N. V. et al. | 2022 | 0 | 2 | 2 | 2 | 1 | 7 |
| **Case report/series (N=149), mean score=7.40** | | | | | | | | |
| 1 | Faa, et.al. | 2003 | 2 | 2 | 2 | 1 | 1 | 8 |
| 2 | Ralph, et.al. | 2004 | 2 | 2 | 2 | 2 | 2 | 10 |
| 3 | Unsworth, et.al. | 2007 | 2 | 2 | 2 | 1 | 0 | 7 |
| 4 | Izzard, et.al. | 2010 | 2 | 2 | 2 | 2 | 2 | 10 |
| 5 | Stewart, et.al. | 2020 | 2 | 0 | 2 | 1 | 1 | 6 |
| 6 | Tshokey, et.al. | 2017 | 2 | 2 | 1 | 2 | 2 | 9 |
| 7 | Tshering, et.al. | 2021 | 2 | 1 | 2 | 1 | 1 | 7 |
| 8 | Jatsho, et.al. | 2022 | 2 | 1 | 1 | 2 | 2 | 8 |
| 9 | Balcells, et.al. | 2011 | 2 | 2 | 2 | 0 | 1 | 7 |
| 10 | Weitzel, et.al. | 2016 | 2 | 2 | 2 | 1 | 1 | 8 |
| 11 | Cracco, et.al. | 2000 | 2 | 1 | 2 | 2 | 2 | 9 |
| 12 | Vliegenthart-Jongbloed, et.al. | 2013 | 2 | 2 | 2 | 1 | 1 | 8 |
| 13 | Rajagopal, et.al. | 2003 | 2 | 0 | 1 | 1 | 1 | 5 |
| 14 | Mathai, et.al. | 2003 | 2 | 1 | 2 | 1 | 0 | 6 |
| 15 | Mahajan, et.al. | 2007 | 2 | 1 | 2 | 1 | 1 | 7 |
| 16 | Mahajan, et.al. | 2010 | 2 | 2 | 1 | 1 | 2 | 8 |
| 17 | Ahmad, et.al. | 2010 | 2 | 1 | 2 | 1 | 1 | 7 |
| 18 | Mahajan, et.al. | 2011 | 2 | 1 | 2 | 1 | 0 | 6 |
| 19 | Mahajan, et.al. | 2012 | 2 | 1 | 1 | 2 | 1 | 7 |
| 20 | Sharma, et.al. | 2012 | 2 | 2 | 1 | 1 | 1 | 7 |
| 21 | Gupta, et.al. | 2012 | 2 | 1 | 2 | 1 | 1 | 7 |
| 22 | Iqbal, et.al. | 2012 | 2 | 1 | 2 | 1 | 1 | 7 |
| 23 | Subbanna, et.al. | 2012 | 2 | 1 | 1 | 2 | 1 | 7 |
| 24 | Saifudheen, et.al. | 2012 | 2 | 1 | 2 | 1 | 1 | 7 |
| 25 | Devarajan, et.al. | 2012 | 2 | 1 | 2 | 1 | 1 | 7 |
| 26 | Yadav, et.al. | 2013 | 2 | 1 | 2 | 1 | 1 | 7 |
| 27 | Goswami, et.al. | 2013 | 2 | 2 | 2 | 2 | 2 | 10 |
| 28 | Kumar, et.al. | 2013 | 2 | 1 | 1 | 2 | 2 | 8 |
| 29 | Karanth, et.al. | 2013 | 2 | 1 | 2 | 1 | 1 | 7 |
| 30 | Mookkappan, et.al. | 2014 | 2 | 1 | 2 | 2 | 2 | 9 |
| 31 | Raj, et.al. | 2014 | 2 | 1 | 2 | 1 | 1 | 7 |
| 32 | Prakash, et.al. | 2014 | 2 | 2 | 2 | 1 | 2 | 9 |
| 33 | Saxena, et.al. | 2014 | 2 | 1 | 2 | 2 | 2 | 9 |
| 34 | Bhatt, et.al. | 2014 | 2 | 0 | 2 | 2 | 2 | 8 |
| 35 | Sethi, et.al. | 2014 | 2 | 0 | 2 | 0 | 2 | 6 |
| 36 | Manickam, et.al. | 2014 | 2 | 1 | 2 | 2 | 2 | 9 |
| 37 | G K, et.al. | 2014 | 2 | 0 | 1 | 2 | 1 | 6 |
| 38 | Ahmed, et.al. | 2014 | 2 | 1 | 1 | 2 | 1 | 7 |
| 39 | Subroto, et.al. | 2015 | 2 | 1 | 2 | 2 | 2 | 9 |
| 40 | Sankuratri, et.al. | 2015 | 2 | 1 | 2 | 2 | 1 | 8 |
| 41 | Devarajan, et.al. | 2015 | 2 | 2 | 1 | 1 | 1 | 7 |
| 42 | Chandramohan, et.al. | 2015 | 2 | 1 | 2 | 1 | 1 | 7 |
| 43 | Sood, et.al. | 2015 | 2 | 1 | 2 | 1 | 2 | 8 |
| 44 | Basheer, et.al. | 2015 | 2 | 1 | 2 | 2 | 2 | 9 |
| 45 | Koti, et.al. | 2015 | 2 | 1 | 2 | 1 | 0 | 6 |
| 46 | Eswaradass, et.al. | 2015 | 2 | 0 | 2 | 1 | 2 | 7 |
| 47 | Mittal, et.al. | 2015 | 2 | 1 | 2 | 1 | 1 | 7 |
| 48 | Banda, et.al. | 2016 | 2 | 1 | 1 | 2 | 2 | 8 |
| 49 | Pai, et.al. | 2016 | 2 | 1 | 2 | 2 | 1 | 8 |
| 50 | Ete, et.al. | 2016 | 2 | 2 | 1 | 1 | 1 | 7 |
| 51 | Mahajan, et.al. | 2016 | 2 | 0 | 1 | 1 | 1 | 5 |
| 52 | Chaturvedi, et.al. | 2016 | 2 | 1 | 1 | 2 | 2 | 8 |
| 53 | Ray, et.al. | 2016 | 2 | 1 | 1 | 2 | 2 | 8 |
| 54 | Meena, et.al. | 2016 | 2 | 1 | 1 | 1 | 1 | 6 |
| 55 | A, et.al. | 2016 | 2 | 1 | 1 | 2 | 1 | 7 |
| 56 | Biswal, et.al. | 2017 | 2 | 1 | 2 | 2 | 2 | 9 |
| 57 | Seow, et.al. | 2017 | 2 | 1 | 2 | 2 | 2 | 9 |
| 58 | Rohini, et.al. | 2017 | 2 | 0 | 1 | 1 | 1 | 5 |
| 59 | Dhanapriya, et.al. | 2017 | 2 | 1 | 1 | 2 | 1 | 7 |
| 60 | Sarma, et.al. | 2017 | 2 | 0 | 1 | 2 | 1 | 6 |
| 61 | Jajoo, et.al. | 2017 | 2 | 2 | 1 | 2 | 2 | 9 |
| 62 | Kumar, et.al. | 2017 | 2 | 0 | 1 | 2 | 2 | 7 |
| 63 | Vajpayee, et.al. | 2017 | 2 | 0 | 1 | 2 | 2 | 7 |
| 64 | Koraluru, et.al. | 2017 | 2 | 1 | 2 | 0 | 1 | 6 |
| 65 | Sv, et.al. | 2017 | 2 | 1 | 1 | 0 | 1 | 5 |
| 66 | Garg, et.al. | 2018 | 2 | 1 | 2 | 2 | 2 | 9 |
| 67 | Saha, et.al. | 2018 | 2 | 2 | 2 | 1 | 1 | 8 |
| 68 | Sarkar, et.al. | 2018 | 2 | 1 | 2 | 1 | 1 | 7 |
| 69 | Zainab, et.al. | 2018 | 2 | 1 | 2 | 2 | 1 | 8 |
| 70 | Shveta, et.al. | 2018 | 2 | 2 | 1 | 1 | 2 | 8 |
| 71 | Agrawal, et.al. | 2018 | 2 | 1 | 1 | 1 | 1 | 6 |
| 72 | Roy, et.al. | 2018 | 2 | 1 | 2 | 1 | 1 | 7 |
| 73 | Avasthi, et.al. | 2018 | 2 | 1 | 1 | 1 | 0 | 5 |
| 74 | Sethuraman, et.al. | 2019 | 2 | 2 | 0 | 2 | 2 | 8 |
| 75 | Singh, et.al. | 2019 | 2 | 2 | 2 | 2 | 2 | 10 |
| 76 | Mohanty, et.al. | 2019 | 2 | 1 | 1 | 2 | 1 | 7 |
| 77 | Chaudhry, et.al. | 2019 | 2 | 1 | 2 | 1 | 1 | 7 |
| 78 | Bhargava, et.al. | 2019 | 2 | 0 | 2 | 0 | 0 | 4 |
| 79 | Tandon, et.al. | 2019 | 2 | 1 | 1 | 1 | 0 | 5 |
| 80 | Mehta, et.al. | 2019 | 2 | 0 | 1 | 1 | 1 | 5 |
| 81 | Gomber, et.al. | 2019 | 2 | 1 | 1 | 1 | 1 | 6 |
| 82 | Dev, et.al. | 2019 | 2 | 1 | 1 | 1 | 0 | 5 |
| 83 | Guha, et.al. | 2019 | 2 | 2 | 2 | 2 | 0 | 8 |
| 84 | Mehta, et.al. | 2019 | 2 | 2 | 1 | 2 | 2 | 9 |
| 85 | Venketesan, et.al. | 2019 | 2 | 2 | 2 | 2 | 2 | 10 |
| 86 | Roy, et.al. | 2019 | 2 | 1 | 2 | 1 | 1 | 7 |
| 87 | Tayade, et.al. | 2020 | 2 | 1 | 1 | 1 | 1 | 6 |
| 88 | Goyal, et.al. | 2020 | 2 | 1 | 2 | 1 | 1 | 7 |
| 89 | Annadatha, et.al. | 2020 | 2 | 0 | 1 | 2 | 2 | 7 |
| 90 | Maitra, et.al. | 2020 | 2 | 1 | 1 | 2 | 2 | 8 |
| 91 | Kotwal, et.al. | 2020 | 2 | 0 | 1 | 2 | 2 | 7 |
| 92 | Pannu, et.al. | 2020 | 2 | 1 | 2 | 2 | 1 | 8 |
| 93 | Sukumar, et.al. | 2020 | 2 | 1 | 1 | 1 | 1 | 6 |
| 94 | Ghosh, et.al. | 2020 | 2 | 0 | 2 | 2 | 2 | 8 |
| 95 | Thoyyib, et.al. | 2020 | 2 | 1 | 2 | 1 | 2 | 8 |
| 96 | Hulkoti, et.al. | 2020 | 2 | 2 | 1 | 2 | 2 | 9 |
| 97 | Lamech, et.al. | 2020 | 2 | 0 | 1 | 0 | 1 | 4 |
| 98 | Minhas, et.al. | 2020 | 2 | 1 | 1 | 2 | 1 | 7 |
| 99 | Sardana, et.al. | 2020 | 2 | 0 | 2 | 1 | 1 | 6 |
| 100 | Atam, et.al. | 2020 | 2 | 1 | 2 | 1 | 1 | 7 |
| 101 | Gaba, et.al. | 2020 | 2 | 2 | 1 | 2 | 2 | 9 |
| 102 | Dave, et.al. | 2020 | 2 | 1 | 1 | 2 | 2 | 8 |
| 103 | Singh, et.al. | 2020 | 2 | 1 | 2 | 2 | 2 | 9 |
| 104 | Kaur, et.al. | 2020 | 2 | 0 | 1 | 2 | 2 | 7 |
| 105 | Sharma, et.al. | 2020 | 2 | 1 | 1 | 1 | 1 | 6 |
| 106 | Gupta, et.al. | 2020 | 2 | 1 | 1 | 1 | 2 | 7 |
| 107 | Saxena, et.al. | 2020 | 2 | 0 | 1 | 1 | 1 | 5 |
| 108 | Soundararajan, et.al. | 2020 | 2 | 2 | 1 | 2 | 2 | 9 |
| 109 | Mandal, et.al. | 2021 | 2 | 1 | 1 | 2 | 0 | 6 |
| 110 | Chandra, et.al. | 2021 | 2 | 1 | 2 | 2 | 2 | 9 |
| 111 | Singh, et.al. | 2021 | 2 | 1 | 2 | 2 | 2 | 9 |
| 112 | Agarwal, et.al. | 2021 | 2 | 1 | 2 | 2 | 2 | 9 |
| 113 | Das, et.al. | 2021 | 2 | 1 | 1 | 2 | 2 | 8 |
| 114 | Abbas, et.al. | 2021 | 2 | 1 | 1 | 1 | 0 | 5 |
| 115 | Srinithi, et.al. | 2021 | 2 | 0 | 1 | 1 | 1 | 5 |
| 116 | Kumar, et.al. | 2021 | 2 | 1 | 1 | 1 | 1 | 6 |
| 117 | Chauhan, et.al. | 2021 | 2 | 0 | 1 | 1 | 2 | 6 |
| 118 | Pal Grewal, et.al. | 2021 | 2 | 1 | 1 | 1 | 1 | 6 |
| 119 | Philip, et.al. | 2021 | 2 | 1 | 1 | 2 | 1 | 7 |
| 120 | Arasu, et.al. | 2021 | 2 | 0 | 2 | 2 | 2 | 8 |
| 121 | Mohanty, et.al. | 2021 | 2 | 1 | 1 | 1 | 1 | 6 |
| 122 | Kumar, et.al. | 2021 | 2 | 1 | 2 | 1 | 1 | 7 |
| 123 | Marwah, et.al. | 2021 | 2 | 0 | 1 | 2 | 2 | 7 |
| 124 | Naik, et.al. | 2022 | 2 | 1 | 1 | 2 | 2 | 8 |
| 125 | Ghosh, et.al. | 2022 | 2 | 0 | 2 | 2 | 2 | 8 |
| 126 | Gupta, et.al. | 2022 | 2 | 1 | 1 | 2 | 2 | 8 |
| 127 | Thakur, et.al. | 2022 | 2 | 2 | 2 | 2 | 2 | 10 |
| 128 | Singh, et.al. | 2022 | 2 | 1 | 2 | 1 | 2 | 8 |
| 129 | Sen, et.al. | 2022 | 2 | 1 | 2 | 2 | 2 | 9 |
| 130 | Dalal, et.al. | 2022 | 2 | 1 | 2 | 1 | 1 | 7 |
| 131 | Kumar, et.al. | 2022 | 2 | 1 | 1 | 2 | 2 | 8 |
| 132 | Majumder, et.al. | 2022 | 2 | 1 | 2 | 2 | 1 | 8 |
| 133 | Nasr, et.al. | 2009 | 2 | 1 | 2 | 2 | 0 | 7 |
| 134 | Eisermann, et.al. | 2020 | 2 | 2 | 1 | 2 | 2 | 9 |
| 135 | Costa, et.al. | 2021 | 2 | 1 | 2 | 2 | 2 | 9 |
| 136 | Mohamed Zan, et.al. | 2016 | 2 | 1 | 2 | 1 | 2 | 8 |
| 137 | Hafizuddin, et.al. | 2020 | 2 | 1 | 2 | 1 | 2 | 8 |
| 138 | Imad, et.al. | 2021 | 2 | 2 | 1 | 2 | 0 | 7 |
| 139 | Sapkota, et.al. | 2017 | 2 | 2 | 1 | 1 | 1 | 7 |
| 140 | Acharya, et.al. | 2019 | 2 | 1 | 1 | 2 | 2 | 8 |
| 141 | Bhattarai, et.al. | 2020 | 2 | 2 | 1 | 2 | 1 | 8 |
| 142 | Gautam, et.al. | 2021 | 2 | 1 | 1 | 2 | 2 | 8 |
| 143 | Subedi, et.al. | 2021 | 2 | 2 | 1 | 2 | 2 | 9 |
| 144 | Basukala, et.al. | 2022 | 2 | 1 | 2 | 2 | 2 | 9 |
| 145 | Gurung, et.al. | 2022 | 2 | 2 | 1 | 2 | 2 | 9 |
| 146 | Kurup, et.al. | 2013 | 2 | 1 | 2 | 2 | 2 | 9 |
| 147 | Premaratna, et.al. | 2006 | 2 | 1 | 2 | 1 | 1 | 7 |
| 148 | Premaratna, et.al. | 2010 | 2 | 1 | 2 | 1 | 0 | 6 |
| 149 | Premaratna, et.al. | 2015 | 2 | 1 | 2 | 1 | 2 | 8 |
| **Mortality (N=129), mean score=6.76** | | | | | | | | |
| 1 | Derrick, E. H., et.al. | 1961 | 2 | 2 | 2 | 2 | 1 | 9 |
| 2 | Tshokey, T., et.al. | 2019 | 2 | 1 | 0 | 2 | 0 | 5 |
| 3 | Chheng, Kheng, et.al. | 2013 | 2 | 2 | 2 | 1 | 0 | 7 |
| 4 | Mackie, T. T., et.al. | 1946 | 1 | 2 | 0 | 1 | 1 | 5 |
| 5 | Mathai, E., et.al. | 2003 | 1 | 2 | 0 | 1 | 1 | 5 |
| 6 | Varghese, G. M., et.al. | 2006 | 1 | 2 | 2 | 1 | 1 | 7 |
| 7 | Mahajan, Sanjay K., et.al. | 2008 | 2 | 1 | 2 | 1 | 1 | 7 |
| 8 | Chrispal, Anugrah, et.al. | 2010 | 1 | 2 | 1 | 1 | 1 | 6 |
| 9 | Singh, S. Ibungochouba, et.al. | 2010 | 2 | 2 | 2 | 2 | 1 | 9 |
| 10 | Chrispal, Anugrah, et.al. | 2010 | 1 | 2 | 2 | 1 | 1 | 7 |
| 11 | Basu, Gopal, et.al. | 2011 | 2 | 2 | 1 | 1 | 1 | 7 |
| 12 | Rathi, Narendra B., et.al. | 2011 | 1 | 2 | 2 | 1 | 1 | 7 |
| 13 | NA | 2011 | 2 | 2 | 0 | 0 | 0 | 4 |
| 14 | Palanivel, Sengottaiya, et.al. | 2012 | 1 | 2 | 1 | 1 | 1 | 6 |
| 15 | Narvencar, Kedareshwar P. S., et.al. | 2012 | 2 | 2 | 1 | 1 | 1 | 7 |
| 16 | Abrahamsen, Siri Kratter, et.al. | 2013 | 2 | 1 | 1 | 1 | 1 | 6 |
| 17 | Varghese, George M., et.al. | 2013 | 1 | 2 | 2 | 1 | 1 | 7 |
| 18 | Singh, S. P., et.al. | 2014 | 2 | 2 | 2 | 1 | 1 | 8 |
| 19 | Singh, Ragini, et.al. | 2014 | 2 | 2 | 2 | 1 | 0 | 7 |
| 20 | Jamil, Md, et.al. | 2014 | 1 | 2 | 1 | 1 | 1 | 6 |
| 21 | Varghese, George M., et.al. | 2014 | 1 | 2 | 2 | 1 | 1 | 7 |
| 22 | Subbalaxmi, M. V. S., et.al. | 2014 | 1 | 2 | 2 | 1 | 1 | 7 |
| 23 | Bithu, R., et.al. | 2014 | 2 | 2 | 1 | 1 | 1 | 7 |
| 24 | Griffith, Mathew, et.al. | 2014 | 1 | 2 | 2 | 1 | 1 | 7 |
| 25 | Kumar Bhat, Nowneet, et.al. | 2014 | 1 | 2 | 1 | 1 | 1 | 6 |
| 26 | Kumar, Vivek, et.al. | 2014 | 1 | 2 | 2 | 1 | 1 | 7 |
| 27 | Bhat, N. K., et.al. | 2014 | 2 | 2 | 1 | 1 | 1 | 7 |
| 28 | Oberoi, Aroma, et.al. | 2014 | 2 | 2 | 2 | 1 | 1 | 8 |
| 29 | Luthra, M., et.al. | 2015 | 2 | 2 | 2 | 1 | 1 | 8 |
| 30 | Das, Dipmala, et.al. | 2015 | 1 | 2 | 2 | 1 | 1 | 7 |
| 31 | Jayanandan, Rajesh, et.al. | 2015 | 1 | 2 | 2 | 1 | 1 | 7 |
| 32 | Abhilash, Kundavaram Paul Prabhakar et.al. | 2015 | 1 | 2 | 2 | 1 | 1 | 7 |
| 33 | Venkategowda, Pradeep M., et.al. | 2015 | 1 | 2 | 2 | 1 | 1 | 7 |
| 34 | Abhilash, Kundavaram Paul Prabhakar, et.al. | 2016 | 1 | 2 | 2 | 1 | 1 | 7 |
| 35 | Sivarajan, Sunuraj, et.al. | 2016 | 1 | 2 | 1 | 1 | 1 | 6 |
| 36 | Gurunathan, P. S., et.al. | 2016 | 1 | 2 | 1 | 1 | 1 | 6 |
| 37 | Ramaganeshan, D., et.al. | 2016 | 2 | 2 | 1 | 1 | 1 | 7 |
| 38 | Mahajan, Sanjay K., et.al. | 2016 | 1 | 2 | 2 | 1 | 1 | 7 |
| 39 | Krishnan, R., et.al. | 2016 | 1 | 2 | 2 | 1 | 1 | 7 |
| 40 | Sharma, Navneet, et.al. | 2016 | 2 | 2 | 2 | 1 | 1 | 8 |
| 41 | Rajan, S. J., et.al. | 2016 | 1 | 2 | 1 | 1 | 1 | 6 |
| 42 | Bhargava, Anurag, et.al. | 2016 | 1 | 2 | 2 | 1 | 1 | 7 |
| 43 | Bhat, Nowneet Kumar, et.al. | 2016 | 2 | 2 | 1 | 1 | 1 | 7 |
| 44 | Rose, Winsley, et.al. | 2016 | 1 | 2 | 2 | 1 | 1 | 7 |
| 45 | Sarangi, R., et.al. | 2016 | 1 | 2 | 2 | 1 | 1 | 7 |
| 46 | Kumar, Ritesh, et.al. | 2016 | 1 | 2 | 1 | 1 | 1 | 6 |
| 47 | nan | 2017 | 2 | 2 | 0 | 0 | 1 | 5 |
| 48 | Narvencar, Kedareshwar, et.al. | 2017 | 2 | 2 | 2 | 1 | 1 | 8 |
| 49 | Mittal, Mahima, et.al. | 2017 | 1 | 2 | 2 | 1 | 1 | 7 |
| 50 | Takhar, Rajendra Prasad, et.al. | 2017 | 1 | 2 | 1 | 1 | 1 | 6 |
| 51 | Singhi, Sunit, et.al. | 2017 | 1 | 2 | 2 | 1 | 1 | 7 |
| 52 | Mina, S. S., et.al. | 2017 | 1 | 2 | 1 | 1 | 0 | 5 |
| 53 | Negi, Ankita, et.al. | 2018 | 1 | 2 | 0 | 1 | 1 | 5 |
| 54 | Ganesh, Ramaswamy, et.al. | 2018 | 1 | 2 | 2 | 1 | 1 | 7 |
| 55 | Dhar, S. K., et.al. | 2018 | 1 | 2 | 1 | 1 | 1 | 6 |
| 56 | Rauf, Abdul, et.al. | 2018 | 1 | 2 | 1 | 1 | 1 | 6 |
| 57 | Nadda, Nishant, et.al. | 2019 | 1 | 2 | 2 | 1 | 1 | 7 |
| 58 | Roy, S., et.al. | 2019 | 2 | 2 | 2 | 1 | 1 | 8 |
| 59 | Sandhu, A., et.al. | 2019 | 1 | 2 | 2 | 1 | 1 | 7 |
| 60 | Gaba, S., et.al. | 2019 | 2 | 2 | 2 | 1 | 1 | 8 |
| 61 | Sharma, Ritin, et.al. | 2019 | 1 | 2 | 1 | 1 | 1 | 6 |
| 62 | Jain, Deepak, et.al. | 2019 | 1 | 2 | 2 | 1 | 1 | 7 |
| 63 | Mallick, Sanjay Kumar, et.al. | 2019 | 2 | 2 | 1 | 1 | 1 | 7 |
| 64 | Saluja, M., et.al. | 2019 | 1 | 2 | 1 | 1 | 1 | 6 |
| 65 | Pathania, Monika, et.al. | 2019 | 1 | 2 | 1 | 1 | 1 | 6 |
| 66 | Jayaprakash, V., et.al. | 2019 | 1 | 2 | 1 | 1 | 1 | 6 |
| 67 | Perumalla, Susmitha Karunasree, et.al. | 2019 | 1 | 2 | 2 | 1 | 1 | 7 |
| 68 | Nallasamy, Karthi, et.al. | 2020 | 1 | 2 | 2 | 1 | 1 | 7 |
| 69 | Kiruthika, V. M., et.al. | 2020 | 1 | 2 | 1 | 1 | 1 | 6 |
| 70 | Alam, Areesha, et.al. | 2020 | 1 | 2 | 2 | 1 | 1 | 7 |
| 71 | Lakshmi, Ram Mohan Mylavarapu Venkata Naga, et.al. | 2020 | 2 | 2 | 2 | 1 | 1 | 8 |
| 72 | Thakur, C. K., et.al. | 2020 | 2 | 2 | 2 | 1 | 0 | 7 |
| 73 | Hazra, D., et.al. | 2020 | 2 | 2 | 1 | 1 | 1 | 7 |
| 74 | Gaba, Saurabh, et.al. | 2020 | 1 | 2 | 1 | 1 | 1 | 6 |
| 75 | Pannu, A., et.al. | 2021 | 1 | 2 | 2 | 1 | 1 | 7 |
| 76 | Bansod, Yogendra V., et.al. | 2021 | 1 | 2 | 2 | 1 | 1 | 7 |
| 77 | Singh, Sweta, et.al. | 2021 | 2 | 2 | 2 | 1 | 1 | 8 |
| 78 | Mahajan, Vidushi, et.al. | 2021 | 1 | 2 | 1 | 1 | 1 | 6 |
| 79 | Barnabas, Rohit, et.al. | 2021 | 1 | 2 | 2 | 1 | 1 | 7 |
| 80 | Roychowdhury, S., et.al. | 2021 | 1 | 2 | 1 | 1 | 1 | 6 |
| 81 | Williams, Vijai, et.al. | 2021 | 1 | 2 | 2 | 1 | 1 | 7 |
| 82 | Panda, A., et.al. | 2021 | 1 | 2 | 1 | 1 | 1 | 6 |
| 83 | Muthukrishnan, K., et.al. | 2021 | 1 | 2 | 1 | 1 | 1 | 6 |
| 84 | Pannu, Ashok Kumar, et.al. | 2021 | 1 | 2 | 1 | 1 | 1 | 6 |
| 85 | Loganathan, S. K., et.al. | 2021 | 1 | 2 | 2 | 1 | 1 | 7 |
| 86 | Parasher, V., et.al. | 2022 | 1 | 2 | 0 | 1 | 1 | 5 |
| 87 | Sultan, A., et.al. | 2022 | 2 | 2 | 2 | 1 | 1 | 8 |
| 88 | Punjabi, Narain H., et.al. | 2012 | 1 | 1 | 2 | 1 | 1 | 6 |
| 89 | Phongmany, Simaly, et.al. | 2006 | 1 | 2 | 2 | 1 | 1 | 7 |
| 90 | 王树声, et.al. | 1989 | 2 | 2 | 1 | 2 | 1 | 8 |
| 91 | 张祥浩, et.al. | 1991 | 2 | 2 | 0 | 2 | 1 | 7 |
| 92 | Zhang, Li-juan, et.al. | 2007 | 2 | 2 | 2 | 2 | 1 | 9 |
| 93 | 李剑萍, et.al. | 2009 | 2 | 2 | 2 | 1 | 1 | 8 |
| 94 | 王晟 | 2009 | 2 | 2 | 2 | 1 | 0 | 7 |
| 95 | 罗小松 | 2010 | 1 | 2 | 2 | 1 | 1 | 7 |
| 96 | 廖云珍 and 叶晓光 | 2013 | 1 | 2 | 1 | 1 | 1 | 6 |
| 97 | 罗雷, et.al. | 2013 | 1 | 2 | 1 | 2 | 1 | 7 |
| 98 | 孙明, et.al. | 2013 | 1 | 2 | 2 | 1 | 1 | 7 |
| 99 | Wei, Yuehong, et.al. | 2014 | 2 | 2 | 2 | 2 | 1 | 9 |
| 100 | 郑伟华, et.al. | 2015 | 1 | 2 | 2 | 1 | 1 | 7 |
| 101 | 曾诚, et.al. | 2015 | 1 | 2 | 0 | 1 | 1 | 5 |
| 102 | 杨松, et.al. | 2016 | 1 | 2 | 2 | 1 | 1 | 7 |
| 103 | 陆永姝, et.al. | 2017 | 1 | 2 | 2 | 1 | 0 | 6 |
| 104 | Zhao, Dongying, et.al. | 2017 | 1 | 2 | 2 | 1 | 1 | 7 |
| 105 | 吴超, et.al. | 2017 | 1 | 2 | 2 | 1 | 1 | 7 |
| 106 | Wang, Ying, et.al. | 2019 | 1 | 2 | 0 | 1 | 1 | 5 |
| 107 | 何道兴. | 2021 | 1 | 2 | 2 | 1 | 1 | 7 |
| 108 | Brown, G. W., et.al. | 1984 | 2 | 2 | 2 | 1 | 1 | 8 |
| 109 | Jensenius, Mogens, et.al. | 2013 | 1 | 2 | 1 | 2 | 1 | 7 |
| 110 | Sedhain, A. and Bhattarai, G.B. | 2017 | 1 | 2 | 2 | 1 | 1 | 7 |
| 111 | Adhikari, Shital, et.al. | 2018 | 1 | 2 | 2 | 1 | 1 | 7 |
| 112 | Pathak, Santosh, et.al. | 2019 | 2 | 2 | 1 | 1 | 1 | 7 |
| 113 | Adhikari, Shital, et.al. | 2020 | 1 | 2 | 2 | 1 | 1 | 7 |
| 114 | Marks, Michael, et.al. | 2016 | 1 | 1 | 2 | 1 | 1 | 6 |
| 115 | Lee, Chang-Seop, et.al. | 2009 | 1 | 2 | 2 | 1 | 1 | 7 |
| 116 | Moon, Kyoung Min, et.al. | 2016 | 1 | 2 | 2 | 1 | 1 | 7 |
| 117 | Hwang, Kyung, et.al. | 2017 | 1 | 2 | 2 | 1 | 1 | 7 |
| 118 | Yang, Shang-Hsien, et.al. | 2007 | 2 | 2 | 2 | 1 | 1 | 8 |
| 119 | Jim, Wai-Tim, et.al. | 2009 | 1 | 2 | 2 | 1 | 1 | 7 |
| 120 | Watt, G., et.al. | 2003 | 2 | 2 | 2 | 1 | 1 | 8 |
| 121 | Leelarasamee, Amorn, et.al. | 2004 | 2 | 2 | 2 | 1 | 1 | 8 |
| 122 | Silpapojakul, Kamkarn, et.al. | 2004 | 2 | 2 | 2 | 1 | 0 | 7 |
| 123 | Charoensak, A., et.al. | 2006 | 1 | 2 | 2 | 1 | 1 | 7 |
| 124 | Suputtamongkol, Y., et.al. | 2009 | 1 | 2 | 2 | 1 | 1 | 7 |
| 125 | Thipmontree, Wilawan, et.al. | 2016 | 1 | 2 | 2 | 1 | 1 | 7 |
| 126 | Wangrangsimakul, Tri, et.al. | 2018 | 2 | 2 | 2 | 1 | 1 | 8 |
| 127 | Nadjm, Behzad, et.al. | 2014 | 1 | 2 | 1 | 1 | 1 | 6 |
| 128 | Hamaguchi, Sugihiro, et.al. | 2015 | 1 | 2 | 2 | 1 | 1 | 7 |
| 129 | Trung, Nguyen Vu, et.al. | 2019 | 2 | 2 | 2 | 1 | 1 | 8 |
| **Risk factor (N=37), mean score=8.70** | | | | | | | | |
| 1 | Sharma, Puran K., et.al.^314^ | 2009 | 2 | 2 | 2 | 2 | 1 | 9 |
| 2 | Varghese, George M., et.al.^315^ | 2016 | 2 | 2 | 0 | 2 | 1 | 7 |
| 3 | Trowbridge Paul et.al.^47^ | 2017 | 2 | 2 | 2 | 2 | 1 | 9 |
| 4 | Thangaraj, Jeromie Wesley Vivian, et.al.^316^ | 2018 | 2 | 2 | 2 | 2 | 2 | 10 |
| 5 | George, Tina, Rajan, et.al.^317^ | 2018 | 2 | 2 | 2 | 2 | 1 | 9 |
| 6 | Rose, Winsley, et.al.^318^ | 2019 | 2 | 2 | 2 | 2 | 1 | 9 |
| 7 | Devamani, Carol S., et.al.^68^ | 2020 | 1 | 2 | 2 | 2 |  | 7 |
| 8 | Vallee, Julie, et.al.^107^ | 2010 | 2 | 2 | 2 | 2 | 1 | 9 |
| 9 | Roberts, Tamalee, et.al.^319^ | 2021 | 2 | 2 | 1 | 2 | 1 | 8 |
| 10 | 季红燕, et.al.^320^ | 1998 | 2 | 2 | 0 | 2 | 1 | 7 |
| 11 | 吴钦永, et.al.^321^ | 1998 | 2 | 2 | 1 | 2 | 1 | 8 |
| 12 | 张倩, et.al.^138^ | 2007 | 2 | 2 | 2 | 2 | 1 | 9 |
| 13 | 查震球, et.al.^322^ | 2011 | 2 | 2 | 2 | 2 | 1 | 9 |
| 14 | 丁磊, et.al.^323^ | 2012 | 2 | 2 | 2 | 2 | 1 | 9 |
| 15 | 方益荣, et.al.^324^ | 2012 | 2 | 2 | 2 | 2 | 1 | 9 |
| 16 | 刘隽, et.al.^325^ | 2013 | 2 | 1 | 2 | 2 | 1 | 8 |
| 17 | 邱尔臣,et.al.^152^ | 2013 | 2 | 2 | 2 | 2 | 1 | 9 |
| 18 | 张立芹, et.al.^326^ | 2013 | 2 | 2 | 2 | 2 | 1 | 9 |
| 19 | 何义林, et.al.^327^ | 2014 | 2 | 2 | 1 | 2 | 1 | 8 |
| 20 | Wei, Yuehong, et.al.^289^ | 2014 | 2 | 2 | 2 | 2 | 2 | 10 |
| 21 | Hu, Jianli, et.al.^328^ | 2015 | 2 | 2 | 2 | 2 | 1 | 9 |
| 22 | 李孟磊, et.al.^329^ | 2016 | 2 | 2 | 0 | 2 | 1 | 7 |
| 23 | 郭志南, et.al.^330^ | 2017 | 2 | 2 | 1 | 2 | 1 | 8 |
| 24 | 魏跃红, et.al.^331^ | 2017 | 2 | 2 | 1 | 2 | 1 | 8 |
| 25 | 廖勇, et.al.^332^ | 2018 | 2 | 2 | 1 | 2 | 1 | 8 |
| 26 | 李大鹏, et.al.^333^ | 2019 | 2 | 2 | 1 | 2 | 1 | 8 |
| 27 | 李鹏宾, et.al.^334^ | 2019 | 2 | 2 | 2 | 2 | 1 | 9 |
| 28 | 李飒,et.al.^163^ | 2019 | 2 | 2 | 2 | 2 | 1 | 9 |
| 29 | Musa, T. H., et.al.^335^ | 2021 | 2 | 2 | 2 | 2 | 2 | 10 |
| 30 | Tay, Sun Tee, et.al.^174^ | 2013 | 2 | 2 | 2 | 2 | 2 | 10 |
| 31 | Gautam, Rajendra, et.al.^180^ | 2019 | 2 | 2 | 2 | 2 | 2 | 10 |
| 32 | Kim, D. M., et.al.^336^ | 2008 | 2 | 2 | 0 | 2 | 1 | 7 |
| 33 | Kweon, Sun-Seog, et.al.^337^ | 2009 | 2 | 2 | 2 | 2 | 2 | 10 |
| 34 | Kim, Dong-Seob, et.al.^338^ | 2018 | 2 | 2 | 2 | 2 | 2 | 10 |
| 35 | Park, Ji-Hyuk, et.al.^198^ | 2021 | 1 | 2 | 2 | 2 | 1 | 8 |
| 36 | Chang, Yi-Chin, et.al.^339^ | 2021 | 2 | 2 | 2 | 2 | 1 | 9 |
| 37 | Tran, Hanh Thi Duc, et.al.^340^ | 2021 | 2 | 2 | 2 | 2 | 2 | 10 |

# Reference

1. Graves S, Wang L, Nack Z, Jones S. Rickettsia serosurvey in Kimberley ,Western Australia. *The American journal of tropical medicine and hygiene* 1999; **60**(5): 786-9.

2. Mathews KO, Phalen D, Norris JM, et al. Serological Evidence of Exposure to Spotted Fever Group and Typhus Group Rickettsiae in Australian Wildlife Rehabilitators. *Pathogens (Basel, Switzerland)* 2021; **10**(6).

3. Faruque LI, Zaman RU, Gurley ES, et al. Prevalence and clinical presentation of Rickettsia, Coxiella, Leptospira, Bartonella and chikungunya virus infections among hospital-based febrile patients from December 2008 to November 2009 in Bangladesh. *BMC infectious diseases* 2017; **17**(1): 141.

4. Kingston HW, Hossain M, Leopold S, et al. Rickettsial Illnesses as Important Causes of Febrile Illness in Chittagong, Bangladesh. *Emerging infectious diseases* 2018; **24**(4).

5. Tshokey T, Stenos J, Durrheim D, Eastwood K, Nguyen C, Graves S. Seroprevalence of rickettsial infections and Q fever in Bhutan. *PLoS neglected tropical diseases* 2017; **11**(11): e0006107.

6. Tshokey T, Stenos J, Durrheim DN, et al. Rickettsial Infections and Q Fever Amongst Febrile Patients in Bhutan. *Tropical medicine and infectious disease* 2018; **3**(1).

7. Tshokey T, Stenos J, Durrheim DN, et al. The epidemiology of rickettsial diseases, scrub typhus and q fever in bhutan: A first report. *American Journal of Tropical Medicine and Hygiene* 2019; **101**(5): 363-4.

8. Chheng K, Carter MJ, Emary K, et al. A prospective study of the causes of febrile illness requiring hospitalization in children in Cambodia. *PloS one* 2013; **8**(4): e60634.

9. Mueller TC, Siv S, Khim N, et al. Acute undifferentiated febrile illness in rural Cambodia: a 3-year prospective observational study. *PloS one* 2014; **9**(4): e95868.

10. Weitzel T, Acosta-Jamett G, Jiang J, et al. Human seroepidemiology of Rickettsia and Orientia species in Chile - A cross-sectional study in five regions. *Ticks and tick-borne diseases* 2020; **11**(6): 101503.

11. Horton KC, Jiang J, Maina A, et al. Evidence of rickettsia and orientia infections among abattoir workers in Djibouti. *American Journal of Tropical Medicine and Hygiene* 2016; **95**(2): 462-5.

12. Chao C-C, Zhang Z, Belinskaya T, Chen H-W, Ching W-M. Leptospirosis and Rickettsial Diseases Sero-Conversion Surveillance Among U.S. Military Personnel in Honduras. *Military medicine* 2021.

13. Isaac R, Varghese G, Mathai E, J M, Joseph I. Scrub typhus: prevalence and diagnostic issues in rural Southern India. *Clinical in fectious diseases: an official publication of the Infectious Diseases Society of America* 2004; **39**(9): 1395-6.

14. Varghese GM, Abraham OC, Mathai D, et al. Scrub typhus among hospitalised patients with febrile illness in South India: magnitude and clinical predictors. *The Journal of infection* 2006; **52**(1): 56-60.

15. Chrispal A, Boorugu H, Gopinath KG, et al. Scrub typhus: an unrecognized threat in South India - clinical profile and predictors of mortality. *Tropical doctor* 2010; **40**(3): 129-33.

16. Basu G, Chrispal A, Boorugu H, et al. Acute kidney injury in tropical acute febrile illness in a tertiary care centre--RIFLE criteria validation. *Nephrology, dialysis, transplantation : official publication of the European Dialysis and Transplant Association - European Renal Association* 2011; **26**(2): 524-31.

17. Rathi NB, Rathi AN, Goodman MH, Aghai ZH. Rickettsial diseases in central India: proposed clinical scoring system for early detection of spotted fever. *Indian pediatrics* 2011; **48**(11): 867-72.

18. Mittal V, Gupta N, Bhattacharya D, et al. Serological evidence of rickettsial infections in Delhi. *The Indian journal of medical research* 2012; **135**(4): 538-41.

19. Narvencar KPS, Rodrigues S, Nevrekar RP, et al. Scrub typhus in patients reporting with acute febrile illness at a tertiary health care institution in Goa. *The Indian journal of medical research* 2012; **136**(6): 1020-4.

20. Abrahamsen SK, Haugen CN, Rupali P, et al. Fever in the tropics: aetiology and case-fatality - a prospective observational study in a tertiary care hospital in South India. *BMC infectious diseases* 2013; **13**: 355.

21. Bithu R, Kanodia V, Maheshwari RK. Possibility of scrub typhus in fever of unknown origin (FUO) cases: an experience from Rajasthan. *Indian journal of medical microbiology* 2014; **32**(4): 387-90.

22. Griffith M, Peter JV, Karthik G, et al. Profile of organ dysfunction and predictors of mortality in severe scrub typhus infection requiring intensive care admission. *Indian journal of critical care medicine : peer-reviewed, official publication of Indian Society of Critical Care Medicine* 2014; **18**(8): 497-502.

23. Oberoi A, Varghese SR. Scrub typhus-an emerging entity: a study from a tertiary care hospital in North India. *Indian journal of public health* 2014; **58**(4): 281-3.

24. Singh R, Singh SP, Ahmad N. A Study of Etiological Pattern in an Epidemic of Acute Febrile Illness during Monsoon in a Tertiary Health Care Institute of Uttarakhand, India. *Journal of clinical and diagnostic research : JCDR* 2014; **8**(6): MC01-3.

25. Usha K, Kumar E, Kalawat U, Siddhartha Kumar B, Chaudhury A, Sai Gopal DVR. Seroprevalence of scrub typhus among febrile patients: A preliminary study. *Asian Journal of Pharmaceutical and Clinical Research* 2014; **7**: 19-21.

26. Das D, Das B, Roy AD, Singh TSK. Common Infectious Etiologies of Acute Febrile Illness in a Remote Geographical Location: Could Scrub Typhus be the Most Common Cause? *Br J Med Med Res* 2015.

27. Rashmi KS, Murthy NS, Ravikumar KL. Rickettsial Diseases: A Study Evidenced by Weil-Felix Test in a Tertiary Care Hospital. *International Journal of Scientific Study* 2015; **3**(8): 128-31.

28. Roopa KS, Karthika K, Sugumar M, Bammigatti C, Shamanna SB, Harish BN. Serodiagnosis of Scrub Typhus at a Tertiary Care Hospital from Southern India. *Journal of clinical and diagnostic research : JCDR* 2015; **9**(11): DC05-7.

29. Sengupta M, Anandan S, Daniel D, JudePrakash JAJ. Scrub typhus seroprevalence in healthy Indian population. *Journal of Clinical and Diagnostic Research* 2015; **9**(10): DM01-DM2.

30. Stephen S, Sangeetha B, Ambroise S, et al. Outbreak of scrub typhus in Puducherry & Tamil Nadu during cooler months. *The Indian journal of medical research* 2015; **142**(5): 591-7.

31. Abhilash KPP, Jeevan JA, Mitra S, et al. Acute Undifferentiated Febrile Illness in Patients Presenting to a Tertiary Care Hospital in South India: Clinical Spectrum and Outcome. *Journal of global infectious diseases* 2016; **8**(4): 147-54.

32. Bhargava A, Kaushik R, Kaushik RM, et al. Scrub typhus in Uttarakhand & adjoining Uttar Pradesh: Seasonality, clinical presentations & predictors of mortality. *The Indian journal of medical research* 2016; **144**(6): 901-9.

33. Farhana A, Bali N, Kanth F, Farooq R, Haq IU, Shah P. Serological Evidence of Scrub Typhus among Cases of PUO in the Kashmir Valley- A Hospital Based Study. *Journal of clinical and diagnostic research : JCDR* 2016; **10**(5): DC24-6.

34. Jakharia A, Borkakoty B, Biswas D, Yadav K, Mahanta J. Seroprevalence of Scrub Typhus Infection in Arunachal Pradesh, India. *Vector borne and zoonotic diseases (Larchmont,NY)* 2016; **16**(10): 659-63.

35. Kalal BS, Puranik P, Nagaraj S, Rego S, Shet A. Scrub typhus and spotted fever among hospitalised children in South India: Clinical profile and serological epidemiology. *Indian journal of medical microbiology* 2016; **34**(3): 293-8.

36. Khan SA, Khamo V, Uriah HJ, Bora T, Dutta P. Scrub typhus in hilly regions of northeast India. *Indian Journal of Medical Research* 2016; **144**: 138-40.

37. Rajan SJ, Sathyendra S, Mathuram AJ. Scrub typhus in pregnancy: Maternal and fetal outcomes. *Obstetric Medicine* 2016; **9**(4): 164-6.

38. Sarangi R, Pradhan S, Debata NC, Mahapatra S. Clinical profile of scrub typhus in children treated in a tertiary care hospital in eastern India. *Pediatria Polska* 2016; **91**(4): 308-11.

39. Arun Babu T, Vijayadevagaran V, Ananthakrishnan S. Characteristics of Pediatric Scrub Typhus Eschar in South Indian Children. *Pediatric dermatology* 2017; **34**(2): 124-7.

40. Manjunath VG, Hedda S, Vijay Kumar GS, Kumar JK, Murthy DS. Clinical features, laboratory findings and complications of scrub typhus in South Indian children. *Journal of Nepal Paediatric Society* 2017; **37**(1): 21-4.

41. Mina SS, Kumar V, Chhapola V. Emerging Infections in Children in North India: Scrub Typhus. *Journal of Pediatric Infectious Diseases* 2017; **12**(2): 114-8.

42. Mittal M, Thangaraj JWV, Rose W, et al. Scrub Typhus as a Cause of Acute Encephalitis Syndrome, Gorakhpur, Uttar Pradesh, India. *Emerging infectious diseases* 2017; **23**(8): 1414-6.

43. Morch K, Manoharan A, Chandy S, et al. Acute undifferentiated fever in India: a multicentre study of aetiology and diagnostic accuracy. *BMC infectious diseases* 2017; **17**(1): 665.

44. Narvencar K, Kaur G, Rodrigues S. Rickettsial Infections in Goa-Not Just Scrub Typhus! *The Journal of the Association of Physicians of India* 2017; **65**(8): 24-7.

45. Shelke YP, Deotale VS, Maraskolhe DL. Spectrum of infections in acute febrile illness in central India. *Indian journal of medical microbiology* 2017; **35**(4): 480-4.

46. Singhi S, Rungta N, Nallasamy K, et al. Tropical Fevers in Indian Intensive Care Units: A Prospective Multicenter Study. *Indian journal of critical care medicine : peer-reviewed, official publication of Indian Society of Critical Care Medicine* 2017; **21**(12): 811-8.

47. Trowbridge Paul PD, Premkumar, Prasanna S., Varghese George M. Prevalence and risk factors for scrub typhus in South India. *Tropical medicine & international health: TM&IH* 2017; **22**(5): 576-82.

48. Vivian Thangaraj JW, Mittal M, Verghese VP, et al. Scrub Typhus as an Etiology of Acute Febrile Illness in Gorakhpur, Uttar Pradesh, India, 2016. *The American journal of tropical medicine and hygiene* 2017; **97**(5): 1313-5.

49. Arvind N, Prabhakar K, Savitha N, Mahendra M. Clinical and microbiological profile of patients with acute febrile illness attending a tertiary care hospital in South India. *Journal of Pure and Applied Microbiology* 2018; **12**(2): 757-63.

50. Giri PP, Roy J, Saha A. Scrub Typhus - A Major Cause of Pediatric Intensive Care Admission and Multiple Organ Dysfunction Syndrome: A Single-Center Experience from India. *Indian journal of critical care medicine : peer-reviewed, official publication of Indian Society of Critical Care Medicine* 2018; **22**(2): 107-10.

51. Jacob SM, Sekkizhar G, Kanagasabai S, Gopal P, Gopal T, Elumalai S. Seroprevalence and clinical manifestations of scrub typhus infection in Chennai city: A cross-sectional study. *International Journal of Health and Allied Sciences* 2018; **7**(3): 201-3.

52. Jain P, Prakash S, Tripathi PK, et al. Emergence of Orientia tsutsugamushi as an important cause of Acute Encephalitis Syndrome in India. *PLoS neglected tropical diseases* 2018; **12**(3): e0006346.

53. Raina S, Raina RK, Agarwala N, Raina SK, Sharma R. Coinfections as an aetiology of acute undifferentiated febrile illness among adult patients in the sub-Himalayan region of north India. *Journal of vector borne diseases* 2018; **55**(2): 130-6.

54. Rauf A, Singhi S, Nallasamy K, Walia M, Ray P. Non-Respiratory and Non-Diarrheal Causes of Acute Febrile Illnesses in Children Requiring Hospitalization in a Tertiary Care Hospital in North India: A Prospective Study. *The American journal of tropical medicine and hygiene* 2018; **99**(3): 783-8.

55. Rizvi M, Sultan A, Chowdhry M, et al. Prevalence of scrub typhus in pyrexia of unknown origin and assessment of interleukin-8, tumor necrosis factor-alpha, and interferon-gamma levels in scrub typhus-positive patients. *Indian journal of pathology & microbiology* 2018; **61**(1): 76-80.

56. Stephen S, Ambroise S, Gunasekaran D, et al. Serological evidence of spotted fever group rickettsiosis in and around Puducherry, south India-A three years study. *Journal of vector borne diseases* 2018; **55**(2): 144-50.

57. Behera B, Biswal M, Das RR, et al. Clinico-epidemiological analysis of scrub typhus in hospitalised patients presenting with acute undifferentiated febrile illness: A hospital-based study from Eastern India. *Indian journal of medical microbiology* 2019; **37**(2): 278-80.

58. Boda S, Goutham VVN. Clinical Spectrum of Acute Undifferentiated Fever - An Experience from a Tertiary Care Centre. 2019; **6**.

59. Devamani CS, Prakash JAJ, Alexander N, Suzuki M, Schmidt W-P. Hospitalisations and outpatient visits for undifferentiated fever attributable to scrub typhus in rural South India: Retrospective cohort and nested case-control study. *PLoS neglected tropical diseases* 2019; **13**(2): e0007160.

60. Jain D, Nand N, Giri K, Bhutani J. Scrub typhus infection, not a benign disease: an experience from a tertiary care center in Northern India. *Medicine and pharmacy reports* 2019; **92**(1): 36-42.

61. Jha R, Jaiswal AK. Clinico-laboratory profile of central nervous system infection by scrub typhus at a tertiary care hospital. 2019; **6**.

62. Mallick SK, Hazra S, Nandi T, Sarkar A. Scrub typhus: a hospital-based study in the northern districts of West Bengal, India. 2019; **7**.

63. Patil R, Kulkarni S. Evaluation of Acute Febrile Illness in Patients Presenting to a Tertiary Care Hospital. *Journal of Evolution of Medical and Dental Sciences-Jemds* 2019; **8**(50): 3801-4.

64. Perumalla SK, Paul S, Abhilash KPP, et al. Eschar and IgM ELISA in the diagnosis of scrub typhus. *Indian journal of medical microbiology* 2019; **37**(1): 113-5.

65. Rao PN, van Eijk AM, Choubey S, et al. Dengue, chikungunya, and scrub typhus are important etiologies of non-malarial febrile illness in Rourkela, Odisha, India. *BMC infectious diseases* 2019; **19**(1): 572.

66. Shankar V, Roopalatha M, Lakshmi S. Scrub Typhus in a Tertiary Care Hospital in Visakhapatnam. *Journal of Evolution of Medical and Dental Sciences-Jemds* 2019; **8**(40): 3028-31.

67. Alam A, Agarwal P, Prabha J, et al. Prediction Rule for Scrub Typhus Meningoencephalitis in Children: Emerging Disease in North India. *Journal of child neurology* 2020; **35**(12): 820-7.

68. Devamani CS, Schmidt W-P, Ariyoshi K, Anitha A, Kalaimani S, Prakash JAJ. Risk Factors for Scrub Typhus, Murine Typhus, and Spotted Fever Seropositivity in Urban Areas, Rural Plains, and Peri-Forest Hill Villages in South India: A Cross-Sectional Study. *The American journal of tropical medicine and hygiene* 2020; **103**(1): 238-48.

69. Hazra D, Fernandes J, Nekkanti A, Abhilash K. Scrub typhus: Clinical presentation and severity. *Current Medical Issues* 2020; **18**(2): 111-4.

70. Kamble S, Mane A, Sane S, et al. Seroprevalence & seroincidence of Orientia tsutsugamushi infection in Gorakhpur, Uttar Pradesh, India: A community-based serosurvey during lean (April-May) & epidemic (October-November) periods for acute encephalitis syndrome. *The Indian journal of medical research* 2020; **151**(4): 350-60.

71. Lakshmi RMMVN, Dharma TV, Sudhaharan S, et al. Prevalence of scrub typhus in a tertiary care centre in Telangana, south India. *Iranian journal of microbiology* 2020; **12**(3): 204-8.

72. Vikram K, Agarwala P, Bhargava A, Jain Y, Jagzape T, Wasnik P. Scrub typhus and leptospirosis in rural and urban settings of central India: a preliminary evaluation. *Tropical doctor* 2020; **50**(2): 111-5.

73. Yaqoob S, Siddiqui AH, Shukla P. scrub typhus: A Neglected tropical Disease and A Potential Threat in North India. *Journal of Pure and Applied Microbiology* 2020; **14**(2): 1589-93.

74. Anupriya A, Rajkumar B, Prabhusaran N, Banthavi PS. Scrub typhus infection among puo cases in the pediatric population in a tertiary care hospital. *Asian Journal of Pharmaceutical and Clinical Research* 2021; **14**(5): 137-9.

75. Bal M, Kar CR, Behera HK, et al. Scrub typhus associated acute kidney injury: An emerging health problem in Odisha, India. *Journal of vector borne diseases* 2021; **58**(4): 359-67.

76. Behera SP, Kumar N, Singh R, et al. Molecular Detection and Genetic Characterization of Orientia tsutsugamushi from Hospitalized Acute Encephalitis Syndrome Cases During Two Consecutive Outbreaks in Eastern Uttar Pradesh, India. *Vector borne and zoonotic diseases (Larchmont, NY)* 2021; **21**(10): 747-52.

77. Kavirayani V, Madiyal M, Aroor S, Chhabra S. Clinical profile and role of serology in pediatric acute febrile illness: Experience from a tertiary care hospital in South India. *Clinical Epidemiology and Global Health* 2021; **12**: 100898.

78. Khan SA, Murhekar MV, Bora T, et al. Seroprevalence of Rickettsial Infections in Northeast India: A Population-Based Cross-Sectional Survey. *Asia-Pacific journal of public health* 2021; **33**(5): 516-22.

79. Mahajan V, Guglani V, Singla N, Chander J. Spectrum of Multiorgan Dysfunction in Scrub Typhus Infection. *Journal of tropical pediatrics* 2021; **67**(4).

80. Matlani M, Kumar P, Meena SS, Vashistha M, Dogra V. Etiological trends and epidemiological profile of tropical fever in children presenting with acute undifferentiated fever at a tertiary care centre in north india. *Journal of Nepal Paediatric Society* 2021; **41**(1): 54-60.

81. Mittal V, Singh P, Shukla S, Karoli R. Scrub typhus: An under-reported and emerging threat - hospital based study from central and eastern Uttar Pradesh, India. *Journal of vector borne diseases* 2021; **58**(4): 323-8.

82. Panda A, Kishore SV, Pradhan M, Champatiray J. Clinico-epidemiological and outcome of scrub typhus in paediatric patients: An observational study from Odisha, India. *Journal of Clinical and Diagnostic Research* 2021; **15**(7): SC1-SC4.

83. Paulraj PS, Renu G, Ranganathan K, Leo VJ, Veeramanoharan R. First seroprevalence report of scrub typhus from the tribal belts of the Nilgiris district, Tamil Nadu, India. *The Indian journal of medical research* 2021; **153**(4): 503-7.

84. Singh S, Patel SS, Sahu C, Ghoshal U. Seroprevalence trends of Scrub typhus among the febrile patients of Northern India: A prospective cross-sectional study. *Journal of family medicine and primary care* 2021; **10**(7): 2552-7.

85. Somasunder VM, Akila K, Sijimol S, et al. Serological detection and epidemiological factors associated with scrub typhus among undifferentiated febrile illness patients in a tertiary care hospital. *Journal of Pure and Applied Microbiology* 2021; **15**(3): 1442-8.

86. Husain U, Kalyan RK, Jahan A, Gupta KK, Verma SK. Scrub Typhus and its co-infection with Leptospirosis at a tertiary care hospital in Uttar Pradesh. *Tropical doctor* 2022: 494755221077744.

87. Sultan A, Shuaib A, Rizvi M, Khan F, Chaudhry R, Thakur CK. Scrub Typhus: An Emerging Etiology among Undiagnosed Febrile Cases in Western Part of Uttar Pradesh, India. *Journal of Pure and Applied Microbiology* 2022; **16**(1): 606-12.

88. Wolff J, De Graaf W. Two New Cases oí Mite Fever in Java. *Geneeskundig Tijdschrift voor Nederlandsche-Indie* 1939; **79**(39).

89. Dennis D, Hadi T, Brown R, Sukaeri S, Leksana B, Cholid R. A survey of scrub and murine typhus in the Ancol section of Jakarta, Indonesia. *The Southeast Asian Journal of Tropical Medicine and Public Health* 1981; **12**(4): 574-80.

90. Gandahusada S, Dennis D, Stafford E, et al. Infectious disease risks to transmigrant communities in Indonesia: a survey in Lampung province, Sumatra. *Indonesian Bulletin of Health Research* 1981; **9**(1): 20381.

91. Hadi TR, Supalin S, Nalim S, Chang A. Ekologi Penyakit Scrub Typhus di Unit Desa Transmigrasi Mulyorejo, Way Abung Ih, Lampung Utara: suatu Penelitian Pendahuluan. *Indonesian Bulletin of Health Research* 1984; **12**(2): 63374.

92. Corwin AL, Soeprapto W, Widodo PS, et al. Short report: surveillance of rickettsial infections in Indonesian military personnel during peace keeping operations in Cambodia. *The American journal of tropical medicine and hygiene* 1997; **57**(5): 569-70.

93. Richards AL, Soeatmadji DW, Widodo MA, et al. Seroepidemiologic evidence for murine and scrub typhus in Malang, Indonesia. *The American journal of tropical medicine and hygiene* 1997; **57**(1): 91-5.

94. Richards AL, Ratiwayanto S, Rahardjo E, et al. Serologic evidence of infection with ehrlichiae and spotted fever group rickettsiae among residents of Gag Island, Indonesia. *The American journal of tropical medicine and hygiene* 2003; **68**(4): 480-4.

95. Punjabi NH, Taylor WRJ, Murphy GS, et al. Etiology of acute, non-malaria, febrile illnesses in Jayapura, northeastern Papua, Indonesia. *The American journal of tropical medicine and hygiene* 2012; **86**(1): 46-51.

96. Lokida D, Hadi U, Lau C-Y, et al. Underdiagnoses of Rickettsia in patients hospitalized with acute fever in Indonesia: observational study results. *BMC infectious diseases* 2020; **20**(1): 364.

97. Hamzah IH, Saeed NAAAH, Al-Gharrawi SAR. Comparative study between serological tests and PCR for diagnosis of Rickettsial diseases in patients with fever of unknown origin. *Meta Gene* 2020; **25**: 100762.

98. Olson JG, Bourgeois AL, Irving GS, Hodge FA, Van Peenen PF. Seroepidemiological evidence of infectious diseases in United States marine corps personnel, Okinawa, Japan, 1975-1976. *Military Medicine* 1979; **144**(3): 175-6.

99. Miyairi T, Kijima H, Murata M, et al. [Shichito fever in Izu Shichito Islands. 4. Incidence of anti-R. tsutsugamushi antibody among inhabitant's sera (author's transl)]. *Kansenshogaku zasshi The Journal of the Japanese Association for Infectious Diseases* 1980; **54**(6): 284-90.

100. Takada N, Tatefuji N, Hoshino T, Ogata A, Fujiki N. [Epidemiology of tsutsugamushi disease in Hokuriku district, Japan. 1. Prevalence of antibody to Rickettsia tsutsugamushi]. *Kansenshogaku zasshiThe Journal of the Japanese Association for Infectious Diseases* 1984; **58**(12): 1279-84.

101. Kasuya S, Iwasa M, Hioki A, et al. [Studies on tsutsugamushi disease in Gifu Prefecture. I. Isolation of Rickettsia tsutsugamushi from wild rodents at a new area and epidemiological studies on patients and latent patients in the prefecture]. *Kansenshogaku zasshi The Journal of the Japanese Association for Infectious Diseases* 1985; **59**(5): 471-7.

102. Ishikura M, Watanabe M, Matsuura K, et al. Epidemiological studies on the background of the endemic occurrence of tsutsugamushi disease in Toyama Prefecture. II. Anti-Rickettsia tsutsugamushi antibody-positive rate in inhabitants of endemic and nonendemic areas. *Microbiol Immunol* 1987; **31**(10): 985-99.

103. Sando E, Suzuki M, Katoh S, et al. Distinguishing Japanese Spotted Fever and Scrub Typhus, Central Japan, 2004- 2015. *Emerging infectious diseases* 2018; **24**(9): 1633-41.

104. Thiga JW, Mutai BK, Eyako WK, et al. High seroprevalence of antibodies against spotted fever and scrub typhus bacteria in patients with febrile Illness, Kenya. *Emerging infectious diseases* 2015; **21**(4): 688-91.

105. Maina AN, Farris CM, Odhiambo A, et al. Q fever, scrub typhus, and rickettsial diseases in children, Kenya, 2011-2012. *Emerging Infectious Diseases* 2016; **22**(5): 883-6.

106. Phongmany S, Rolain J-M, Phetsouvanh R, et al. Rickettsial infections and fever, Vientiane, Laos. *Emerging infectious diseases* 2006; **12**(2): 256-62.

107. Vallee J, Thaojaikong T, Moore CE, et al. Contrasting spatial distribution and risk factors for past infection with scrub typhus and murine typhus in Vientiane City, Lao PDR. *PLoS neglected tropical diseases* 2010; **4**(12): e909.

108. Mayxay M, Castonguay-Vanier J, Chansamouth V, et al. Causes of non-malarial fever in Laos: a prospective study. *The Lancet Global health* 2013; **1**(1): e46-54.

109. Dittrich S, Rattanavong S, Lee SJ, et al. Orientia, rickettsia, and leptospira pathogens as causes of CNS infections in Laos: a prospective study. *The Lancet Global health* 2015; **3**(2): e104-12.

110. Chansamouth V, Thammasack S, Phetsouvanh R, et al. The Aetiologies and Impact of Fever in Pregnant Inpatients in Vientiane, Laos. *PLoS neglected tropical diseases* 2016; **10**(4): e0004577.

111. 高韵苕, 卢玉韵, 吴光华, et al. 福建××岛恙虫病流行病学调查. *人民军医* 1960: 51-8.

112. 刘国栋, 刘国平, 金理华, 娄丹, 张兴旺. 吉林省珲春地区恙虫病及其它立克次体病调查. *中国人兽共患病杂志* 1987; (5): 41-2.

113. 耿际泉, 周朝益, 麦家和, 张殿兰, 王玲. 云南东南部立克次体病血清学调查与病原体分离. *云南医药* 1987; (1): 37-40.

114. 张鸿武. 恙虫病患者和流行区健康人的外斐氏反应结果分析. *临床检验杂志* 1991; (2): 98-9.

115. 刘国栋, 李黎, 蔡守仁, 曹俊伟. 獐子岛部分健康人群恙虫病自然感染状况调查. *解放军预防医学杂志* 1992; (2): 43-5.

116. Lu Z, Hu L, Chai Z. A new natural focus of scrub typhus found in Hunchun. *Zhong hua liu xing bing xue za zhi= Zhong hua liu xing bing xue za zhi* 1994; **15**(1): 31-3.

117. 王均利，李萍，段爱香，许运珍，姚允超，孟凡彬，刘玉华，王玉琴. 蒙阴县恙虫病调查报告. *临沂医专学报* 1996; (2): 123-5.

118. 胡玲美, 鲁志新, 蔡增林, 金显涛, 赵占林. 东北部分地区恙虫病血清学调查. *中华流行病学杂志* 1996; (1): 32.

119. Yang ZQ, Yu XM, Liu YX. [Studies on clinical epidemiology of Tsutsugamushi disease of the autumn-winter type in the easten suburbs of Jinan]. *Zhonghua liu xing bing xue za zhi = Zhonghua liuxingbingxue zazhi* 1997; **18**(4): 233-5.

120. 周剑平, 侯志华, 康素华, 于擎, 陈小彬. 莆田市98例恙虫病流行病学调查报告. *现代预防医学* 1997; (4): 118.

121. 郑乡占, 霍勤, 江志礼, 覃顺寿, 段林枝, 李清. 山西南部恙虫病流行病学调查. *解放军预防医学杂志* 1997; (3): 44-5.

122. 黄昭穗, 林晖, 王荔华, et al. 南日岛健康人群恙虫病血清学调查. *解放军预防医学杂志* 1998; (3): 40-1.

123. 鲁志新, 温青莉, 解志刚, et al. 东北三省部分地区人血清恙虫病立克次体抗体调查. *中国人兽共患病杂志* 1999; (4): 107+60.

124. 刘运喜, 吴钦永, 杨占清, et al. 山东费县秋冬型恙虫病疫源地调查研究. *中国公共卫生* 1999; (2): 27-8.

125. Wang S, Jiang P, Huang J, et al. [The recognition of the epidemic area of tsutsugamushi disease on Nan Peng Lie Island in China and the strategy of prevention]. *Zhonghualiuxingbingxuezazhi=Zhong hua liu xing bing xue za zhi* 2000; **21**(3): 212-5.

126. 陈香蕊, 于强, 张永国, et al. 山西省恙虫病调查与病原学研究. *军事医学科学院院刊* 2000; (4): 275-7+81.

127. 唐天开, 詹道成, 陆振豸, 范传海, 彭雪军. 南海某珊瑚岛恙虫病流行病学调查. *解放军预防医学杂志* 2001; (5): 356-7.

128. 袁庆虹, 杨卫红, 米竹青, 黄文丽, 张海林. 云南大理地区发热病人恙虫病抗体调查. *地方病通报* 2001; (2): 31-2.

129. 陈素良, 李春明, 师鉴, et al. 河北某村恙虫病发病特点的流行病学研究. *中国媒介生物学及控制杂志* 2001; (2): 125-6.

130. 陈素良, 张亚利, 郭逸秀, et al. 河北省恙虫病的血清流行病学研究. *中国媒介生物学及控制杂志* 2001; (2): 122-4.

131. 林碧瑚, 孙晓娟, 詹志农, 林英姿, 李文广. 海南岛立克次体血清学和病原学调查报告. *海南医学院学报* 2003; (5): 257-60.

132. 袁高林, 陈文锦, 李翔英, 李方平. 宁德市恙虫病地理流行病学调查. *中国媒介生物学及控制杂志* 2003; (5): 372-4.

133. 操敏, 郭恒彬, 唐家琪, 郁兴明, 王柏仁, 杨文富. 福建省平潭岛春季恙虫病流行病学调查研究. *动物医学进展* 2004; (5): 107-9.

134. 吴志伟, 刘言训, 郝宗宇. 河南省立克次体流行病学调查: 山东大学; 2005.

135. Hengbin G, Min C, Kaihua T, Jiaqi T. The foci of scrub typhus and strategies of prevention in the Spring in Pingtan Island, Fujian Province. *Annals of the New York Academy of Sciences* 2006; **1078**: 188-96.

136. 周建荣, 刘惟优, 赖庆文. 106例感染性疾病致不明原因长期发热病因分析. *赣南医学院学报* 2007; (4): 602.

137. 尹骏, 展海燕. 靖江市人群恙虫病感染情况调查. *江苏卫生保健* 2007; (1): 17.

138. 张倩, 刘运喜, 曹务春. 内蒙古、新疆地区恙虫病流行病学调查研究: 中国人民解放军军事医学科学院; 2007.

139. 吴中发, 邓海智, 朱丰秀, 温江鸿, 刘素华. 江西赣州市恙虫病的调查研究. *新医学* 2008; (6): 390-1.

140. 林光宇, 张守印, 谢克锦, et al. 福建省北部林区人群人粒细胞无形体血清流行病学检测. *中国人兽共患病学报* 2008; **24**(12): 1179-80.

141. 王珊珊, 黄佳亮, 苏建新, 彭桂福, 王研, 李珉珉. 广东省硇州岛恙虫病疫源地的证实. *热带医学杂志* 2008; (1): 58-60.

142. Chai C-l, Lu Q-y, Sun J-m, et al. [Sero-epidemiologic investigation on tick-borne diseases of humans and domestic animals in Zhejiang province]. *Zhonghua liu xing bing xue za zhi = Zhonghua liuxingbingxue zazhi* 2010; **31**(10): 1144-7.

143. 刘红, 曹明华, 张永根, 史永林, 王俊, 张丽娟. 安徽省不同地区人群和家畜恙虫病血清流行病学调查. *安徽预防医学杂志* 2010; **16**(1): 12-3.

144. 薛家芹, 杨会利, 陈勇. 环泰山区域恙虫病东方体感染状况调查及基因序列分析. *中国病原生物学杂志* 2010; **5**(7): 512-6.

145. 张颖, 张之伦, 阴杰莹, et al. 天津市农牧地区2007—2009年莫氏立克次体、横赛巴尔通体、恙虫病东方体血清流行病学调查. *中华流行病学杂志* 2011; (3): 256-9.

146. 苏静静, 杨占清. 济南战区（山东）部分驻地秋冬型恙虫病流行病学特征与预防研究: 第三军医大学; 2011.

147. 姜仁杰, 沈进进, 张盐姝, et al. 盐城市2006-2010年恙虫病流行病学研究. *医学动物防制* 2011; **27**(12): 1079-81.

148. 谭兆营, 李亮, 张丽娟. 江苏省几种主要类型人畜立克次体抗体阳性检出情况的现况调查. *苏州大学学报(医学版)* 2012; **32**(4): 445-9+593.

149. 常利涛, 刀志宏, 梁长威, et al. 云南省人和家畜立克次体病血清流行病学调查(英文). *中国人兽共患病学报* 2013; **29**(7): 724-9.

150. 张立芹, 田丽丽, 窦相峰, 薛慧, 刘志强, 屈宏宇. 北京市平谷区常住人口恙虫病感染状况调查. *中华流行病学杂志* 2013; **34**(12): 1265-6.

151. 田丽丽, 张立芹, 吕燕宁, et al. 北京市恙虫病病例流行病学特征及实际感染病例数估计. *职业与健康* 2013; **29**(21): 2765-8.

152. 邱尔臣, 党荣理, 贾继民, et al. 新疆部分地区人群恙虫病血清流行病学调查. *中国公共卫生管理* 2013; **29**(6): 791-2.

153. 李静, 童淑芬, 杨志芬, et al. 云南省某大学新生中莱姆病和恙虫病的血清流行病学调查. *昆明医科大学学报* 2014; **35**(5): 21-3.

154. 申安然, 罗启松, 姚留昌, 师敏, 田宗茂. 陇川县2009-2013年恙虫病流行现状分析. *医学动物防制* 2014; **30**(4): 393-5.

155. 谭文文, 常亮, 董美华, et al. 宜兴市一般人群立克次体病血清流行病学调查. *中华疾病控制杂志* 2015; **19**(1): 96-8.

156. 韩腾伟, 刘菁, 洪荣涛, et al. 福建省2010—2013年恙虫病流行特征分析. *海峡预防医学杂志* 2015; **21**(6): 8-10.

157. 吕燕宁, 陈丽娟, 窦相峰, et al. 北京市远郊区恙虫病血清流行病学调查. *中国媒介生物学及控制杂志* 2016; **27**(3): 257-9.

158. 张曙光, 田丽丽, 张丽琴, 万帝. 北京市延庆区恙虫病人群感染状况与危险因素研究. *中国预防医学杂志* 2016; **17**(7): 532-4.

159. 陈永亮, 杨育松, 贾丽丽, et al. 北京市北部山区恙虫病血清流行病学调查分析. *中国媒介生物学及控制杂志* 2016; **27**(6): 597-9.

160. 展海燕, 徐贞, 何义林. 江苏省靖江市2011-2015年恙虫病流行特征及影响因素. *中华疾病控制杂志* 2017; **21**(11): 1119-22.

161. 张欢, 张鲍欢, 谈琦琪, 周惠琼, 吴德, 彭志强. 广州市2015年流行性出血热抗体阴性样本病原谱分析. *华南预防医学* 2018; **44**(5): 453-5.

162. 张立芹, 田丽丽, 林晖, 梁慧杰, 薛慧, 刘志强. 北京市平谷区农村社区医院恙虫病筛查结果分析. *寄生虫与医学昆虫学报* 2018; **25**(4): 200-5.

163. 李飒, 王瑞琴, 蔡旭, et al. 北京市昌平区恙虫病血清流行病学调查及危险因素分析. *中国媒介生物学及控制杂志* 2019; **30**(3): 248-51.

164. Gu XL, Qi R, Li WQ, Jiao YJ, Yu H, Yu XJ. Misdiagnosis of scrub typhus as hemorrhagic fever with renal syndrome and potential coinfection of both diseases in patients in shandong province, china, 2013-2014. *PLoS Neglected Tropical Diseases* 2021; **15**(3): e0009270.

165. Cadigan FC, Jr., Andre RG, Bolton M, Gan E, Walker JS. The effect of habitat on the prevalence of human scrub typhus in Malaysia. *Transactions of the Royal Society of Tropical Medicine and Hygiene* 1972; **66**(4): 582-7.

166. Brown GW, Robinson DM, Huxsoll DL, Ng TS, Lim KJ. Scrub typhus: a common cause of illness in indigenous populations. *Transactions of the Royal Society of Tropical Medicine and Hygiene* 1976; **70**(5): 444-8.

167. Brown GW, Robinson DM, Huxsoll DL. Serological evidence for a high incidence of transmission of Rickettsia tsutsugamushi in two Orang Asli settlements in Peninsular Malaysia. *The American journal of tropical medicine and hygiene* 1978; **27**(1): 121-3.

168. Brown GW, Shirai A, Jegathesan M, et al. Febrile illness in Malaysia--an analysis of 1,629 hospitalized patients. *The American journal of tropical medicine and hygiene* 1984; **33**(2): 311-5.

169. Taylor A, Sivarajah A, Kelly DJ, Lewis Jr GE. An analysis of febrile illnesses among members of the Malaysian Police Field Force. *Military Medicine* 1986; **151**(8): 442-5.

170. Tee TS, Kamalanathan M, Suan KA, et al. Seroepidemiologic survey of Orientia tsutsugamushi, Rickettsia typhi, and TT118 spotted fever group rickettsiae in rubber estate workers in Malaysia. *The American journal of tropical medicine and hygiene* 1999; **61**(1): 73-7.

171. Sagin DD, Ismail G, Nasian LM, Jok JJ, Pang EK. Rickettsial infection in five remote Orang Ulu villages in upper Rejang River, Sarawak, Malaysia. *The Southeast Asian journal of tropical medicine and public health* 2000; **31**(4): 733-5.

172. Tay ST, Ho TM, Rohani MY, Devi S. Antibodies to Orientia tsutsugamushi, Rickettsia typhi and spotted fever group rickettsiae among febrile patients in rural areas of Malaysia. *Transactions of the Royal Society of Tropical Medicine and Hygiene* 2000; **94**(3): 280-4.

173. Tay ST, Kamalanathan M, Rohani MY. Antibody prevalence of Orientia tsutsugamushi, Rickettsia typhi and TT118 spotted fever group rickettsiae among Malaysian blood donors and febrile patients in the urban areas. *The Southeast Asian journal of tropical medicine and public health* 2003; **34**(1): 165-70.

174. Tay ST, Mohamed Zan HA, Lim YAL, Ngui R. Antibody prevalence and factors associated with exposure to Orientia tsutsugamushi in different aboriginal subgroups in West Malaysia. *PLoS neglected tropical diseases* 2013; **7**(8): e2341.

175. Grigg MJ, William T, Clemens EG, et al. Rickettsioses as Major Etiologies of Unrecognized Acute Febrile Illness, Sabah, East Malaysia. *Emerging infectious diseases* 2020; **26**(7).

176. Elders PND, Swe M, Phyo AP, et al. Serological evidence indicates widespread distribution of rickettsioses in Myanmar. *International journal of infectious diseases: IJID: official publication of the International Society for Infectious Diseases* 2021; **103**: 494-501.

177. Murdoch DR, Woods CW, Zimmerman MD, et al. The etiology of febrile illness in adults presenting to Patan hospital in Kathmandu, Nepal. *The American journal of tropical medicine and hygiene* 2004; **70**(6): 670-5.

178. Blacksell SD, Sharma NP, Phumratanaprapin W, et al. Serological and blood culture investigations of Nepalese fever patients. *Transactions of the Royal Society of Tropical Medicine and Hygiene* 2007; **101**(7): 686-90.

179. Sedhain A, Bhattarai GR. Renal Manifestation in Scrub Typhus during a Major Outbreak in Central Nepal. *Indian J Nephrol* 2017; **27**(6): 440-5.

180. Gautam R, Parajuli K, Sherchand JB. Epidemiology, Risk Factors and Seasonal Variation of Scrub Typhus Fever in Central Nepal. *Tropical medicine and infectious disease* 2019; **4**(1).

181. Sharma K, Neupane MS, Poudyal S, Subba HK. Prevalence and knowledge of scrub typhus in patients with acute febrile illness in teaching hospital, Chitwan, Nepal. 2019; **8**.

182. Thapa S, Hamal P, Chaudhary NK, Sapkota LB, Singh JP. Burden of scrub typhus among patients with acute febrile illness attending tertiary care hospital in Chitwan, Nepal. *BMJ open* 2020; **10**(9): e034727.

183. Pokhrel A, Rayamajhee B, Khadka S, et al. Seroprevalence and Clinical Features of Scrub Typhus among Febrile Patients Attending a Referral Hospital in Kathmandu, Nepal. *Tropical medicine and infectious disease* 2021; **6**(2).

184. Demma LJ, McQuiston JH, Nicholson WL, et al. Scrub typhus, Republic of Palau. *Emerging infectious diseases* 2006; **12**(2): 290-5.

185. Spicer PE, Taufa T, Benjamin AL. Scrub typhus (Orientia tsutsugamushi), spotted fever (Rickettsia australis)and dengue fever as possible causes of my sterious deaths in the Strickland Gorge area of Southern Highlands and West Sepik Provinces of Papua New Guinea. *Papua and New Guinea medical journal* 2007; **50**(3): 172-83.

186. Kocher C, Jiang J, Morrison AC, et al. Serologic evidence of scrub typhus in the peruvian Amazon. *Emerging Infectious Diseases* 2017; **23**(8): 1389-91.

187. Yen T-Y, Zhang Z, Chao C-C, et al. Serologic Evidence for Orientia Exposure in the Democratic Republic of Sao Tome and Principe. *Vector borne and zoonotic diseases (Larchmont, NY)* 2019; **19**(11): 821-7.

188. Muzaheed, J Fatani A, D Divakar D, Rathod S, S Aloahd M. Hospital based serological evidence of rickettsial diseases and assessment diagnostic tests of pyrexia of unknown origin. *Tropical doctor* 2020; **50**(2): 122-4.

189. Chang WH, Choi MS, Park KH, et al. Seroepidemiological survey of tsutsugamushi disease in Korea, 1987 and 1988. *Journal of the Korean Society for Microbiology* 1989; **24**(2): 185-95.

190. Chang WH, Choi MS, Kee SH, et al. Seroepidemiological survey of Tsutsugamushi disease in Korea, 1989. *Journal of the Korean Society for Microbiology* 1990; **25**(3): 227-35.

191. Jin-Won S, Luck-Ju B, Sang-Hyun KIM, Se-Yeun KIM, Yong-Ju LEE, Ki-Joon S. Seroepidemiologic Analysis of Acute Febrile Illness During 1994-1995 in Korea. *Korean Journal of Infectious Diseases* 1998: 385-91.

192. Jin-Won S, Luck-Ju B, Yong-Ju LEE, Ki-Joon S, Sung-Hee HAN. Seroepidemiologic Analysis of Acute Febrile Illness from Korea in 1996. *Journal of the Korean Society of Virology* 1998: 377-82.

193. Min-Ja KIM, Hee-Jin C, Jang-Wook S, et al. A Prospective Multicenter Study of the Etiological Analysis in Adults with Community-Acquired Pneumonia: Legionella, Leptospira, Hantaan virus and Orientia tsutsugamushi. *Korean Journal of Infectious Diseases* 2001: 24-31.

194. Song JW, Lee JE, Kim SH, et al. Seroepidemiologic analysis of acute febrile illness in Korea during 1997-1998. *Journal of Bacteriology and Virology* 2002; **32**(3): 263-7.

195. Jang W-J, Kim J-H, Choi Y-J, et al. First serologic evidence of human spotted fever group rickettsiosis in Korea. *Journal of clinical microbiology* 2004; **42**(5): 2310-3.

196. Jung H-C, Chon S-B, Oh WS, Lee D-H, Lee H-J. Etiologies of acute undifferentiated fever and clinical prediction of scrub typhus in a non-tropical endemic area. *The American journal of tropical medicine and hygiene* 2015; **92**(2): 256-61.

197. Dong-Seob KIM, Dilaram A, Seok-Ju YOO, Ji-Hyuk P, Kwan LEE. A Study on the Febrile Illness in Autumn among Farmers in Gyeongju-si: Scrub typhus, Leptospirosis, HFRS. *Journal of Agricultural Medicine & Community Health* 2019: 1-10.

198. Park J-H, Gill B, Acharya D, Yoo S-J, Lee KL, Jeongmin. Seroprevalence and Factors Associated with Scrub Typhus Infection among Forestry Workers in National Park Offices in South Korea. *International journal of environmental research and public health* 2021; **18**(6).

199. Um J, Nam Y, Lim JN, et al. Seroprevalence of scrub typhus, murine typhus and spotted fever groups in North Korean refugees. *International Journal of Infectious Diseases* 2021; **106**: 23-8.

200. Liyanapathirana VC, Thevanesam V. Seroepidemiology of rickettsioses in Sri Lanka: a patient based study. *BMC infectious diseases* 2011; **11**: 328.

201. Kularatne SAM, Rajapakse RPVJ, Wickramasinghe WMRS, et al. Rickettsioses in the central hills of Sri Lanka: serological evidence of increasing burden of spotted fever group. *International journal of infectious diseases : IJID : official publication of the International Society for Infectious Diseases* 2013; **17**(11): e988-92.

202. Premaratna R, Ariyaratna N, Attanayake C, Bandara W, Chandrasena N, de Silva HJ. Rickettsial infection among military personnel deployed in Northern Sri Lanka. *BMC infectious diseases* 2014; **14**: 3864.

203. Olson JG, Bourgeois AL. Rickettsia tsutsugamushi infection and scrub typhus incidence among Chinese military personnel in the Pescadores Islands. *American journal of epidemiology* 1977; **106**(2): 172-5.

204. Johnson DE, Crum JW, Hanchalay S, Saengruchi C. Sero-epidemiological survey of Rickettsia tsutsugamushi infection in a rural Thai village. *Transactions of the Royal Society of Tropical Medicine and Hygiene* 1982; **76**(1): 1-3.

205. Duffy PE, Le Guillouzic H, Gass RF, Innis BL. Murine typhus identified as a major cause of febrile illness in a camp for displaced Khmers in Thailand. *The American journal of tropical medicine and hygiene* 1990; **43**(5): 520-6.

206. Silpapojakul K, Chupuppakarn S, Yuthasompob S, et al. Scrub and murine typhus in children with obscure fever in the tropics. *The Pediatric infectious disease journal* 1991; **10**(3): 200-3.

207. Strickman D, Tanskul P, Eamsila C, Kelly DJ. Prevalence of antibodies to rickettsiae in the human population of suburban Bangkok. *The American journal of tropical medicine and hygiene* 1994; **51**(2): 149-53.

208. Eamsila C, Singsawat P, Duangvaraporn A, Strickman D. Antibodies to Orientia tsutsugamushi in Thai soldiers. *The American journal of tropical medicine and hygiene* 1996; **55**(5): 556-9.

209. Frances SP, Eamsila C, Strickman D. Antibodies to Orientia tsutsugamushi in soldiers in northeastern Thailand. *The Southeast Asian journal of tropical medicine and public health* 1997; **28**(3): 666-8.

210. Chanyasanha C, Kaeburong K, Chenchittikul M, Sujirarat D. Seroprevalence of scrub typhus infection in patients with pyrexia at some malaria clinics in three western provinces of Thailand. *Asian Pacific journal of allergy and immunology* 1998; **16**(2): 119-25.

211. Pradutkanchana J, Pradutkanchana S, Kemapanmanus M, Wuthipum N, Silpapojakul K. The etiology of acute pyrexia of unknown origin in children after a flood. *The Southeast Asian journal of tropical medicine and public health* 2003; **34**(1): 175-8.

212. Suputtamongkol Y, Suttinont C, Niwatayakul K, et al. Epidemiology and clinical aspects of rickettsioses in Thailand. *Annals of the New York Academy of Sciences* 2009; **1166**: 172-9.

213. Thitivichianlert S, Panichkul S, Bodhidatta D, et al. Incidence of rickettsial infection in patients with acute fever in provincial Thai army hospitals. *Journal of the Medical Association of Thailand = Chotmaihet thangphaet* 2009; **92**: S39-46.

214. Thipmontree W, Suputtamongkol Y, Tantibhedhyangkul W, Suttinont C, Wongswat E, Silpasakorn S. Human leptospirosis trends: northeast Thailand, 2001-2012. *International journal of environmental research and public health* 2014; **11**(8): 8542-51.

215. Thipmontree W, Tantibhedhyangkul W, Silpasakorn S, Wongsawat E, Waywa D, Suputtamongkol Y. Scrub Typhus in Northeastern Thailand: Eschar Distribution, Abnormal Electrocardiographic Findings, and Predictors of Fatal Outcome. *The American journal of tropical medicine and hygiene* 2016; **95**(4): 769-73.

216. Hinjoy S, Wacharapluesadee S, Iamsirithaworn S, Smithsuwan P, Padungtod P. Zoonotic and vector borne agents causing disease in adult patients hospitalized due to fever of unknown origin in Thailand. *Asian Pacific Journal of Tropical Disease* 2017; **7**(10): 577-81.

217. Rodkvamtook W, Kuttasingkee N, Linsuwanon P, et al. Scrub typhus outbreak in Chonburi Province, Central Thailand, 2013. *Emerging Infectious Diseases* 2018; **24**(2): 361-5.

218. Wangrangsimakul T, Althaus T, Mukaka M, et al. Causes of acute undifferentiated fever and the utility of biomarkers in Chiangrai, northern Thailand. *PLoS neglected tropical diseases* 2018; **12**(5): e0006477.

219. Chaisiri K, Tanganuchitcharnchai A, Kritiyakan A, et al. Risk factors analysis for neglected human rickettsioses in rural communities in Nan province, Thailand: A community-based observational study along a landscape gradient. *PLOS Neglected Tropical Diseases* 2022; **16**(3): e0010256.

220. Gonwong S, Mason CJ, Chuenchitra T, et al. Nationwide Seroprevalence of Scrub Typhus, Typhus, and Spotted Fever in Young Thai Men. *The American journal of tropical medicine and hygiene* 2022.

221. Nadjm B, Thuy P, Trang V, Ha LD, Kinh NV, Wertheim HF. Scrub typhus in the northern provinces of Vietnam: an observational study of admissions to a national referral hospital. *Transactions of the Royal Society of Tropical Medicine and Hygiene* 2014; **108**(11): 739-40.

222. Hamaguchi S, Cuong NC, Tra DT, et al. Clinical and Epidemiological Characteristics of Scrub Typhus and Murine Typhus among Hospitalized Patients with Acute Undifferentiated Fever in Northern Vietnam. *The American journal of tropical medicine and hygiene* 2015; **92**(5): 972-8.

223. Nguyen HLK, Pham HTT, Nguyen TV, et al. The genotypes of Orientia tsutsugamushi, identified in scrub typhus patients in northern Vietnam. *Transactions of the Royal Society of Tropical Medicine and Hygiene* 2017; **111**(3): 137-9.

224. Trung NV, Hoi LT, Thuong NTH, et al. Seroprevalence of Scrub Typhus, Typhus, and Spotted Fever Among Ruraland Urban Populations of Northern Vietnam. *The American journal of tropical medicine and hygiene* 2017; **96**(5): 1084-7.

225. Katoh S, Cuong NC, Hamaguchi S, et al. Challenges in diagnosing scrub typhus among hospitalized patients with undifferentiated fever at a national tertiary hospital in northern Vietnam. *PLoS neglected tropical diseases* 2019; **13**(12): e0007928.

226. Le-Viet N, Le V-N, Chung H, et al. Prospective case-control analysis of the aetiologies of acute undifferentiated fever in Vietnam. *Emerging microbes & infections* 2019; **8**(1): 339-52.

227. Derrick EH. The incidence and distribution of scrub typhus in North Queensland. *Australasian annals of medicine* 1961; **10**: 256-67.

228. Mackie TT. Observations on tsutsugamushi disease (scrub typhus) in Assam and Burma. *Trans R Soc Trop Med Hyg* 1946; **40**: 15-56.

229. Mathai E, Rolain JM, Verghese GM, et al. Outbreak of scrub typhus in southern India during the cooler months. *Annals of the New York Academy of Sciences* 2003; **990**: 359-64.

230. Mahajan SK, Rolain J-M, Sankhyan N, Kaushal RK, Raoult D. Pediatric scrub typhus in Indian Himalayas. *Indian journal of pediatrics* 2008; **75**(9): 947-9.

231. Chrispal A, Boorugu H, Gopinath KG, et al. Acute undifferentiated febrile illness in adult hospitalized patients: the disease spectrum and diagnostic predictors - an experience from a tertiary care hospital in South India. *Tropical doctor* 2010; **40**(4): 230-4.

232. Singh SI, Devi KP, Tilotama R, et al. An outbreak of scrub typhus in Bishnupur district of Manipur, India, 2007. *Tropical doctor* 2010; **40**(3): 169-70.

233. Palanivel S, Nedunchelian K, Poovazhagi V, Raghunadan R, Ramachandran P. Clinical profile of scrub typhus in children. *Indian journal of pediatrics* 2012; **79**(11): 1459-62.

234. Varghese GM, Janardhanan J, Trowbridge P, et al. Scrub typhus in South India: clinical and laboratory manifestations, genetic variability, and outcome. *International journal of infectious diseases : IJID : official publication of the International Society for Infectious Diseases* 2013; **17**(11): e981-7.

235. Singh SP, Singh R, Ahmad N. A study of complications of scrub typhus in a tertiary health care institute of Uttarakhand, India. 2014.

236. Jamil M, Lyngrah KG, Lyngdoh M, Hussain M. Clinical Manifestations and Complications of Scrub Typhus : A Hospital Based Study from North Eastern India. *The Journal of the Association of Physicians of India* 2014; **62**(12): 19-23.

237. Varghese GM, Trowbridge P, Janardhanan J, et al. Clinical profile and improving mortality trend of scrub typhus in South India. *International journal of infectious diseases: IJID: official publication of the International Society for Infectious Diseases* 2014; **23**: 39-43.

238. Subbalaxmi MVS, Madisetty MK, Prasad AK, et al. Outbreak of scrub typhus in Andhra Pradesh--experience at a tertiary care hospital. *The Journal of the Association of Physicians of India* 2014; **62**(6): 490-6.

239. Kumar Bhat N, Dhar M, Mittal G, et al. Scrub typhus in children at a tertiary hospital in north India: clinical profile and complications. *Iranian journal of pediatrics* 2014; **24**(4): 387-92.

240. Kumar V, Kumar V, Yadav AK, et al. Scrub typhus is an under-recognized cause of acute febrile illness with acute kidney injury in India. *PLoS neglected tropical diseases* 2014; **8**(1): e2605.

241. Bhat NK, Dhar M, Mittal G, Chandra H, Rawat A, Chandar V. Scrub typhus: A common rickettsial disease emerging in a new geographical region of north India. *Journal of Pediatric Infectious Diseases* 2014; **9**(2): 93-9.

242. Luthra M, Varma A, Gupta SK, Negi KS. A study of scrub typhus cases from a tertiary care hospital in Dehradun. *Indian Journal of Community Health* 2015; **27**(1): 150-4.

243. Jayanandan R, Manjunath R, Kulkarni P. Health care seeking behaviour and expenditure pattern among Scrub Typhus patients attending a tertiary care hospital in Mysore city. 2015.

244. Abhilash KPP, Gunasekaran K, Mitra S, et al. Scrub typhus meningitis: An under-recognized cause of aseptic meningitis in India. *Neurology India* 2015; **63**(2): 209-14.

245. Venkategowda PM, Rao SM, Mutkule DP, Rao MV, Taggu AN. Scrub typhus: Clinical spectrum and outcome. *Indian journal of critical care medicine : peer-reviewed, official publication of Indian Society of Critical Care Medicine* 2015; **19**(4): 208-13.

246. Sivarajan S, Shivalli S, Bhuyan D, Mawlong M, Barman R. Clinical and paraclinical profile, and predictors of outcome in 90 cases of scrub typhus, Meghalaya, India. *Infectious diseases of poverty* 2016; **5**(1): 91.

247. Gurunathan PS, Ravichandran T, Stalin S, Prabu V, Anandan H. Clinical Profile, Morbidity Pattern and Outcome of Children with Scrub Typhus. *International Journal of Scientific Study* 2016; **4**(2): 247-50.

248. Ramaganeshan D, Ramu P, Ramesh SV, Chaitanya N. Investigatory Profile and Treatment Outcome of Paediatric Scrub Typhus Patients: A Tertiary Care Hospital Experience in Southern India. *Journal of Evolution of Medical and Dental Sciences-Jemds* 2016; **5**(67): 4793-6.

249. Mahajan SK, Raina R, Singh B, et al. Pattern of Clinical Presentation, Laboratory Findings and Mortality Risk Among Patients of Scrub Typhus in Western Himalayas. *The Journal of the Association of Physicians of India* 2016; **64**(3): 26-30.

250. Krishnan R, Pillai RK, Elizabeth KE, Shanavas A, Bindusha S. Pediatric scrub typhus in Southern Kerala: An emerging public health problem. *Clinical Epidemiology and Global Health* 2016; **4**(2): 89-94.

251. Sharma N, Biswal M, Kumar A, Zaman K, Jain S, Bhalla A. Scrub Typhus in a Tertiary Care Hospital in North India. *The American journal of tropical medicine and hygiene* 2016; **95**(2): 447-51.

252. Bhat NK, Pandita N, Saini M, et al. Scrub Typhus: A Clinico-Laboratory Differentiation of Children with and without Meningitis. *Journal of tropical pediatrics* 2016; **62**(3): 194-9.

253. Rose W, Rajan RJ, Punnen A, Ghosh U. Distribution of Eschar in Pediatric Scrub Typhus. *Journal of tropical pediatrics* 2016; **62**(5): 415-20.

254. Kumar R, Thakur S, Bhawani R, Kanga A, Ranjan A. Clinical Profile and Complications of Scrub Typhus: Hospital-Based Study in Sub-Himalayan Region. *The Journal of the Association of Physicians of India* 2016; **64**(12): 30-4.

255. Takhar RP, Bunkar ML, Arya S, Mirdha N, Mohd A. Scrub typhus: A prospective, observational study during an outbreak in Rajasthan, India. *The National medical journal of India* 2017; **30**(2): 69-72.

256. Negi A, Singh Y, Singh M, Saxena SR. A Study of Acute Febrile Illness with Thrombocytopenia from Tertiary Care Centre of Uttarakhand. 2018; **4**.

257. Ganesh R, Suresh N, Pratyusha LL, Janakiraman L, Manickam M, Andal A. Clinical profile and outcome of children with scrub typhus from Chennai, South India. *European journal of pediatrics* 2018; **177**(6): 887-90.

258. Dhar SK, Kabi S, Das C, et al. Clinical spectrum of scrub typhus in a tertiary care hospital at eastern India. *Asian Journal of Pharmaceutical and Clinical Research* 2018; **11**(5): 351-4.

259. Nadda N, Chauhan N, Mahajan A, Chaudhary A, Katoch K, Chamotra S. A descriptive study to quantify severity in patients with severe scrub typhus in hilly city of Northern India. 2019; **6**.

260. Roy S, Majumdar SD, Chakrabartty S, Chakravarti S. A study on scrub typhus in children: Experience in a tertiary care hospital. *Sri Lanka Journal of Child Health* 2019; **49**(2): 155-9.

261. Sandhu A, Rao R, Kamath A, Bhat R. Assessment of Predictors for Severity in Scrub Typhus-A Case-Control Study. *International Journal of Medical Research & Health Sciences* 2019; **8**(12): 68-73.

262. Gaba S, Gupta M, Singla N, Singh R. Clinical outcome and predictors of severity in scrub typhus patients at a tertiary care hospital in Chandigarh, India. *Journal of vector borne diseases* 2019; **56**(4): 367-72.

263. Sharma R, Mahajan SK, Singh B, Raina R, Kanga A. Predictors of Severity in Scrub Typhus. *The Journal of the Association of Physicians of India* 2019; **67**(4): 35-8.

264. Saluja M, Vimlani H, Chittora S, et al. Scrub typhus: Epidemiology, clinical presentation, diagnostic approach, and outcomes. *Journal, Indian Academy of Clinical Medicine* 2019; **20**(1): 15-21.

265. Pathania M, Amisha, Malik P, Rathaur VK. Scrub typhus: Overview of demographic variables, clinical profile, and diagnostic issues in the sub-Himalayan region of India and its comparison to other Indian and Asian studies. *Journal of family medicine and primary care* 2019; **8**(3): 1189-95.

266. Jayaprakash V, Vamsikrishna M, Indhumathi E, Jayakumar M. Scrub typhus-associated acute kidney injury: A study from a South Indian Tertiary Care Hospital. *Saudi journal of kidney diseases and transplantation : an official publication of the Saudi Center for Organ Transplantation, Saudi Arabia* 2019; **30**(4): 883-90.

267. Nallasamy K, Gupta S, Bansal A, et al. Clinical Profile and Predictors of Intensive Care Unit Admission in Pediatric Scrub Typhus: A Retrospective Observational Study from North India. *Indian journal of critical care medicine : peer-reviewed, official publication of Indian Society of Critical Care Medicine* 2020; **24**(6): 445-50.

268. Kiruthika VM, Shruthi TK, Shuba S, Rajakumar PS, Krithika P, Balaji TK. Clinical Profile and Predictors of Outcome for Paediatric Scrub Typhus at a Tertiary Care Hospital in South India. *BMC Infectious Diseases* 2020; **20**.

269. Thakur CK, Chaudhry R, Gupta N, et al. Scrub typhus in patients with acute febrile illness: a 5-year study from India. *QJM : monthly journal of the Association of Physicians* 2020; **113**(6): 404-10.

270. Gaba S, Gaba N, Gupta M, Sharma S. Hepatic and Renal Profile of Scrub Typhus Patients at a Tertiary Care Center in India. *Cureus* 2020; **12**(5): e7925.

271. Pannu A, Saroch A, Sharda S, Debnath M, Biswal M, Sharma N. Circulatory and hepatic failure at admission predicts mortality of severe scrub typhus patients: A prospective cohort study. *Asian Pacific Journal of Tropical Medicine* 2021; **14**(5): 209-13.

272. Bansod YV, Aher AA, Bhole P, Rengaraj K, Jadhav P. Clinical Profile and Treatment Outcome in Scrub Typhus Patients in Central India. *J Assoc Physicians India* 2021; **69**(9): 11-2.

273. Barnabas R, Abhilash K, Varghese GM, Shubanker M, Ramya I, Prakash J. Prospective study to assess the treatment modalities and fever defervescence in patients with scrub typhus from a tertiary care centre in South India. *Journal of vector borne diseases* 2021; **58**(1): 33-8.

274. Roychowdhury S, Ghosh S, Majumder D, Mukhopadhyay P. A Menace without Specific Feature - Scrub Typhus a Reemerging Disease. *J AssocPhys India* 2021; **69**(12): 68-71.

275. Williams V, Menon N, Bhatia P, et al. Hyperferritinemia in children hospitalized with scrub typhus. *Tropical medicine and health* 2021; **49**(1): 15.

276. Muthukrishnan K, Tarikere S, Sivaraman RP, Sankaranarayanan S, Prabaharan K, Kothandam BT. Clinical profile and predictors of outcome for pediatric scrub typhus at a tertiary care hospital. *Archives of Pediatric Infectious Diseases* 2021; **9**(1): 1-6.

277. Pannu AK, Debnath MK, Sharma N, et al. Circulating cardiac biomarkers and echocardiographic abnormalities in patients with scrub typhus: A prospective cohort study from a tertiary care center in North India. *Journal of vector borne diseases* 2021; **58**(3): 193-8.

278. Loganathan SK, Jaybhaye A, Dash N, Punnen A, Ghosh U, Rose W. Acute respiratory distress syndrome in paediatric scrub typhus. *Tropical Doctor* 2021; **51**(4): 514-7.

279. Parasher V, Shaha S, Khatri R, Das S, Yadav S, Mittal U. Pattern of admission and clinical outcome of scrub typhus patients admitted in PICU in Southern Rajasthan. *International Journal of Pharmaceutical and Clinical Research* 2022; **14**(2): 223-8.

280. 王树声, 蔡日芬, 梁绍伶, et al. 广西桂林市郊恙虫病暴发流行的流行病学、病原学和血清学研究. *广西医学* 1989; (3): 153-7.

281. 张祥浩, 李顺波, 张呈芳. 莒县恙虫病流行病学调查报告. *临沂医专学报* 1991; (2): 137-8.

282. Zhang L-j, Li X-m, Zhang D-r, et al. Molecular epidemic survey on co-prevalence of scrub typhus and marine typhus in Yuxi city, Yunnan province of China. *Chinese medical journal* 2007; **120**(15): 1314-8.

283. 李剑萍, 蔡卫平, 王建, 洪文昕. 30例恙虫病临床特征分析. *传染病信息* 2009; **22**(1): 45-8.

284. 王晟. 500例恙虫病并发症临床分析. *中国热带医学* 2009; **9**(8): 1524.

285. 罗小松. 小儿恙虫病150例临床分析. *医学信息(中旬刊)* 2010; **5**(7): 1762-3.

286. 廖云珍, 叶晓光. 广州地区恙虫病流行病学和临床特征分析. *中国热带医学* 2013; **13**(6): 709-12.

287. 罗雷, 张競文, 景钦隆, et al. 广州一起公园恙虫病聚集性事件的现场调查与应急处置. *热带医学杂志* 2013; **13**(12): 1518-20.

288. 孙明, 汪东亮, 赵春云, 陈秋香. 宿迁地区32例恙虫病临床特征分析. *中国血吸虫病防治杂志* 2013; **25**(6): 639-41.

289. Wei Y, Luo L, Jing Q, et al. A city park as a potential epidemic site of scrub typhus: a case-control study of an outbreak in Guangzhou, China. *Parasites & vectors* 2014; **7**: 513.

290. 郑伟华, 陈海斌, 侯六生, 区利娟, 赵润梅, 赵双彪. 广东省中山地区恙虫病临床与流行病学特征及防治. *临床荟萃* 2015; **30**(5): 552-5.

291. 曾诚, 张剑锋, 黄英华. 广西恙虫病并多器官损害168例临床特征分析. *中华灾害救援医学* 2015; **3**(3): 133-5.

292. 杨松, 张耀亭, 邱跃灵. 58例恙虫病并发肺部感染的流行病学及临床分析. *临床肺科杂志* 2016; **21**(8): 1439-42.

293. 陆永姝, 吴超, 陆冬梅, 李秋臣, 周赛, 孙素娟. 24例重症恙虫病患者的临床观察与护理. *当代护士(上旬刊)* 2017; (3): 121-3.

294. Zhao D, Zhang Y, Yin Z, Zhao J, Yang D, Zhou Q. Clinical Predictors of Multiple Organ Dysfunction Syndromes in Pediatric patients with Scrub Typhus. *Journal of tropical pediatrics* 2017; **63**(3): 167-73.

295. 吴超, 姚成州, 王改, 孙明. 恙虫病误诊病例临床诊治剖析. *临床误诊误治* 2017; **30**(3): 51-4.

296. Wang Y, Li M, Xu W, et al. [Analysis of the clinical characteristics of severe tsutsugamushi disease in Yunnan Province from 2017 to 2018]. *Zhonghua wei zhong bing ji jiu yi xue* 2019; **31**(8): 1018-23.

297. 何道兴. 南平地区260例恙虫病患者的临床分析及流行病特征. *中国现代药物应用* 2021; **15**(24): 240-3.

298. Jensenius M, Han PV, Schlagenhauf P, et al. Acute and potentially life-threatening tropical diseases in western travelers--a GeoSentinel multicenter study, 1996-2011. *The American journal of tropical medicine and hygiene* 2013; **88**(2): 397-404.

299. Sedhain A, Bhattarai GR. Renal Manifestation in Scrub Typhus during a Major Outbreak in Central Nepal. *Indian journal of nephrology* 2017; **27**(6): 440-5.

300. Adhikari S, Poudel RS, Shrestha S, Lamichhane P. Predictors of Mortality in Scrub Typhus Infection Requiring Intensive Care Admission in Tertiary Healthcare Centre of Nepal. *Interdisciplinary perspectives on infectious diseases* 2018; **2018**: 4867958.

301. Pathak S, Chaudhary N, Dhakal P, et al. Clinical profile, complications and outcome of scrub typhus in children: A hospital based observational study in central Nepal. *PloS one* 2019; **14**(8): e0220905.

302. Adhikari S, Poudel RS, Shrestha S, Bhandari N. Outcome Predictors in Scrub Typhus Requiring Ventilator and Vasopressor Support. *Journal of Nepal Health Research Council* 2020; **17**(4): 485-90.

303. Marks M, Joshua C, Longbottom J, et al. An outbreak investigation of scrub typhus in Western Province, Solomon Islands, 2014. *Western Pacific surveillance and response journal : WPSAR* 2016; **7**(1): 6-9.

304. Lee C-S, Hwang J-H, Lee H-B, Kwon K-S. Risk factors leading to fatal outcome in scrub typhus patients. *The American journal of tropical medicine and hygiene* 2009; **81**(3): 484-8.

305. Moon KM, Han MS, Rim CaB, et al. Risk Factors for Mechanical Ventilation in Patients with Scrub Typhus Admitted to Intensive Care Unit at a University Hospital. *Tuberculosis and respiratory diseases* 2016; **79**(1): 31-6.

306. Hwang K, Jang HN, Lee TW, et al. Incidence, risk factors and clinical outcomes of acute kidney injury associated with scrub typhus: a retrospective study of 510 consecutive patients in South Korea (2001-2013). *BMJ open* 2017; **7**(3): e013882.

307. Yang S-H, Ho Y-H, Chu C-H, Chu S-Y. Childhood scrub typhus in eastern Taiwan: ten-year experience from a medical center. *Acta paediatrica Taiwanica = Taiwan er ke yi xue hui za zhi* 2007; **48**(6): 332-6.

308. Jim W-T, Chiu N-C, Chan W-T, et al. Clinical manifestations, laboratory findings and complications of pediatric scrub typhus in eastern Taiwan. *Pediatrics and neonatology* 2009; **50**(3): 96-101.

309. Watt G, Jongsakul K, Chouriyagune C, Paris R. Differentiating dengue virus infection from scrub typhus in Thai adults with fever. *American Journal of Tropical Medicine and Hygiene* 2003; **68**(5): 536-8.

310. Leelarasamee A, Chupaprawan C, Chenchittikul M, Udompanthurat S. Etiologies of acute undifferentiated febrile illness in Thailand. *Journal of the Medical Association of Thailand = Chotmaihet thangphaet* 2004; **87**(5): 464-72.

311. Silpapojakul K, Varachit B, Silpapojakul K. Paediatric scrub typhus in Thailand: a study of 73 confirmed cases. *Transactions of the Royal Society of Tropical Medicine and Hygiene* 2004; **98**(6): 354-9.

312. Charoensak A, Chawalparit O, Suttinont C, et al. Scrub typhus: chest radiographic and clinical findings in 130 Thai patients. *Journal of the Medical Association of Thailand = Chotmaihet thangphaet* 2006; **89**(5): 600-7.

313. Trung NV, Hoi LT, Dien VM, et al. Clinical Manifestations and Molecular Diagnosis of Scrub Typhus and Murine Typhus, Vietnam, 2015-2017. *Emerging infectious diseases* 2019; **25**(4).

314. Sharma PK, Ramakrishnan R, Hutin YJF, et al. Scrub typhus in Darjeeling, India: opportunities for simple, practical prevention measures. *Transactions of the Royal Society of Tropical Medicine and Hygiene* 2009; **103**(11): 1153-8.

315. Varghese GR, DeepaFrancis,MarkR.Sarkar,RajivTrowbridge,PaulMuliyil,Jayaprakash. Epidemiology & risk factors of scrub typhus in south India. *The Indian journal of medical research* 2016; **144**(1): 76-81.

316. Thangaraj JWV, Vasanthapuram R, Machado L, et al. Risk Factors for Acquiring Scrub Typhus among Children in Deoria and Gorakhpur Districts, Uttar Pradesh, India, 2017. *Emerging infectious diseases* 2018; **24**(12): 2364-7.

317. George T, Rajan SJ, Peter JV, et al. Risk Factors for Acquiring Scrub Typhus among the Adults. *Journal of global infectious diseases* 2018; **10**(3): 147-51.

318. Rose W, Kang G, Verghese VP, et al. Risk factors for acquisition of scrub typhus in children admitted to a tertiary centre and its surrounding districts in South India: a case control study. *BMC infectious diseases* 2019; **19**(1): 665.

319. Roberts T, Parker DM, Bulterys PL, et al. A spatio-temporal analysis of scrub typhus and murine typhus in Laos; implications from changing landscapes and climate. *PLOS Neglected Tropical Diseases* 2021; **15**(8): e0009685.

320. 季红燕, 张燕波, 刘劫. 68例少儿恙虫病1∶2匹配病例对照研究. *衡阳医学院学报* 1998; (4): 38-9+45.

321. 吴钦永, 刘运喜, 杨占清, 苏明, 秦德太, 于仁芝. 山东省恙虫病危险因素病例的对照研究. *医学动物防制* 1998; (4): 19-20.

322. 查震球, 李群, 叶冬青. 安徽省阜阳市恙虫病疫源地调查研究: 安徽医科大学; 2011.

323. 丁磊, 王显军, 赵仲堂. 秋冬型恙虫病流行特征及影响因素研究: 山东大学; 2012.

324. 方益荣朱保平叶冬青. 安徽省阜阳市恙虫病疫源地调查研究: 安徽医科大学; 2012.

325. 刘隽, 陈邦华, 吴德, et al. 广东省一起恙虫病暴发疫情流行病学调查. *中华流行病学杂志* 2013; **34**(9): 946-7.

326. 张立芹, 窦相峰, 田丽丽, 薛慧, 刘志强, 王全意. 北京市平谷区恙虫病危险因素的病例对照研究. *职业与健康* 2013; **29**(11): 1355-6.

327. 何义林, 戴文军, 移钱华, et al. 2012年靖江市恙虫病危险因素的病例对照研究. *江苏预防医学* 2014; **25**(4): 16-7.

328. Hu J, Tan Z, Ren D, et al. Clinical characteristics and risk factors of an outbreak with scrub typhus in previously unrecognized areas, Jiangsu province, China 2013. *PloS one* 2015; **10**(5): e0125999.

329. 李孟磊, 其木格, 王若琳, 李懿, 苏佳, 黄学勇. 河南省一起恙虫病暴发疫情调查分析. *现代预防医学* 2016; **43**(23): 4384-7+401.

330. 郭志南, 林勇, 陈国伟. 厦门市2013-2015年恙虫病流行特征和感染危险因素分析. *中华卫生杀虫药械* 2017; **23**(4): 337-9.

331. 魏跃红, 李晓宁, 吴新伟, 陶霞, 陈守义, 杨智聪. 广州市恙虫病危险因素的病例对照研究. *中华疾病控制杂志* 2017; **21**(2): 171-4.

332. 廖勇, 黄仁发, 胡晓军, 李建华, 黄鸿山, 徐建民. 江西省赣州市恙虫病危险因素调查. *中国公共卫生* 2018; **34**(12): 1665-7.

333. 李大鹏, 魏跃红, 杨智聪. 广州市恙虫病危险因素与Ot-Sta56基因分型研究: 广东药科大学; 2019.

334. 李鹏宾, 邱宗耀, 陈玉红, et al. 佛山市南海区恙虫病危险因素病例对照研究. *现代预防医学* 2019; **46**(15): 2870-5.

335. Musa TH, Li W, He Y, et al. Factors associated with Scrub Typhus infection: A case-control study from Luhe, China. *The Medical journal of Malaysia* 2021; **76**(4): 474-9.

336. Kim DM, Kim KY, Nam HS, Kweon SS, Park MY, Ryu SY. Risk-factors for human infection with Orientia tsutsugamushi: a case-control study in Korea. *Clinical microbiology and infection : the official publication of the European Society of Clinical Microbiology and Infectious Diseases* 2008; **14**(2): 174-7.

337. Kweon S-S, Choi J-S, Lim H-S, et al. A community-based case-control study of behavioral factors associated with scrub typhus during the autumn epidemic season in South Korea. *The American journal of tropical medicine and hygiene* 2009; **80**(3): 442-6.

338. Kim D-S, Acharya D, Lee K, Yoo S-J, Park J-H, Lim H-S. Awareness and Work-Related Factors Associated with Scrub Typhus: A Case-Control Study from South Korea. *International journal of environmental research and public health* 2018; **15**(6).

339. Chang Y-C, Sun W, Lin J-N, Chen Y-H, Lai C-H, Lee C-H. Epidemiology and risk factors of scrub typhus in Taiwan: A nationwide database study from 1996 to 2014. *Zoonoses and public health* 2021; **68**(8): 876-83.

340. Tran HTD, Hattendorf J, Do HM, et al. Ecological and behavioural risk factors of scrub typhus in central Vietnam: a case-control study. *Infectious diseases of poverty* 2021; **10**(1): 110.

341. Song P, Fang Z, Wang H, et al. Global and regional prevalence, burden, and risk factors for carotid atherosclerosis: a systematic review, meta-analysis, and modelling study. *Lancet Glob Health* 2020; **8**(5): e721-e9.
